# Copper(I)-catalyzed asymmetric 1,6-conjugate allylation

**DOI:** 10.1038/s41467-020-19293-9

**Published:** 2020-10-30

**Authors:** Chang-Yun Shi, Zhi-Zhou Pan, Ping Tian, Liang Yin

**Affiliations:** 1 The Research Center of Chiral Drugs, Innovation Research Institute of Traditional Chinese Medicine and China-Thailand Joint Research Institute of Natural Medicine, Shanghai University of Traditional Chinese Medicine, 1200 Cailun Road, 201203 Shanghai, China; 2 CAS Key Laboratory of Synthetic Chemistry of Natural Substances, Center for Excellence in Molecular Synthesis, Shanghai Institute of Organic Chemistry, University of Chinese Academy of Sciences, Chinese Academy of Sciences, 345 Lingling Road, 200032 Shanghai, China

**Keywords:** Asymmetric catalysis, Homogeneous catalysis, Synthetic chemistry methodology

## Abstract

Catalytic asymmetric conjugate allylation of unsaturated carbonyl compounds is usually difficult to achieve, as 1,2-addition proceeds dominantly and high asymmetric induction is a challenging task. Herein, we disclose a copper(I)-NHC complex catalyzed asymmetric 1,6-conjugate allylation of 2,2-dimethyl-6-alkenyl-4*H*-1,3-dioxin-4-ones. The phenolic hydroxyl group in NHC ligands is found to be pivotal to obtain the desired products. Both aryl group and alkyl group at δ-position are well tolerated with the corresponding products generated in moderate to high yields and high enantioselectivity. Moreover, both 2-substituted and 3-substituted allylboronates serve as acceptable allylation reagents. At last, the synthetic utility of the products is demonstrated in several transformations by means of the versatile terminal olefin and dioxinone groups.

## Abstract (web summary)

Catalytic asymmetric conjugate allylation of unsaturated carbonyl compounds is usually difficult to achieve due to competing 1,2-addition. Here, the authors report a copper(I)-catalyzed asymmetric 1,6-conjugate allylation of 2,2-dimethyl-6-alkenyl-4H-1,3-dioxin-4-ones with good scope and high enantioselectivity.

## Introduction

Catalytic asymmetric conjugate addition of various metal reagents to unsaturated compounds is identified as one of the most important tools in the construction of carbon-carbon bonds in organic synthesis^1–3^. Among the various carbon-based metal reagents, allyl metal reagents exhibit advantages over other alkyl metal reagents as the olefin moiety is more synthetically versatile. Non-enantioselective methods based on different allyl metal (such as, Si, B, Zn, and Sn) species have been disclosed in the past several decades^4^. However, the catalytic asymmetric conjugate addition with allyl metal reagents is still in its infancy as such a reaction is not easy to achieve due to the competitive 1,2-addition and the difficulty in the asymmetric induction.

**Fig. 1:**
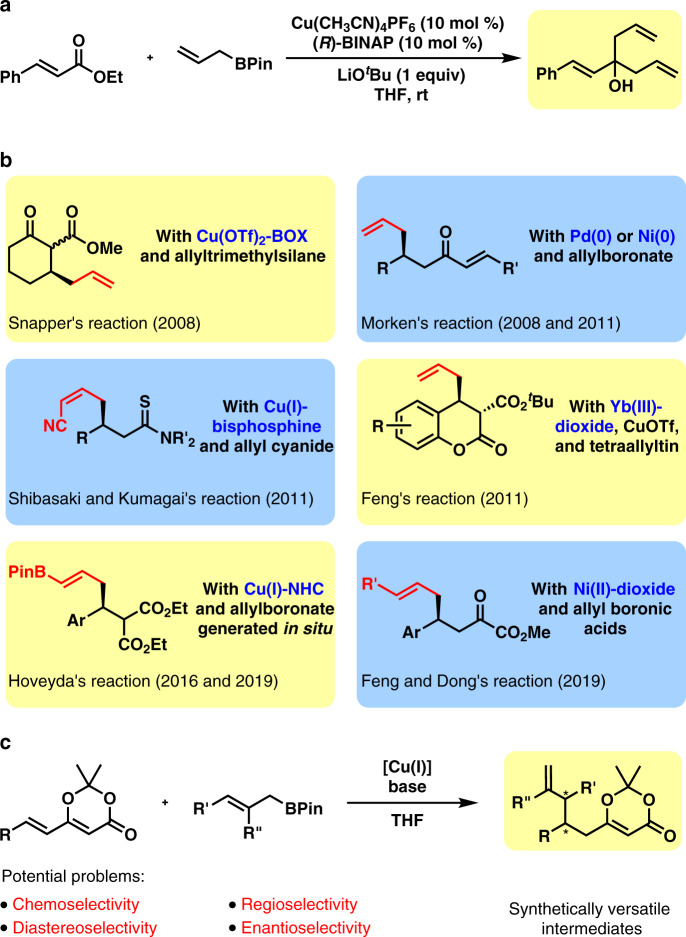
Prior arts in catalytic asymmetric conjugate allylation and our work. **a** Failed copper(I)-catalyzed asymmetric conjugate allylation. **b** Reported catalytic asymmetric conjugate allylation. **c** This work: copper(I)-catalyzed asymmetric conjugate 1,6-allylation.

Indeed, catalytic asymmetric allylation of aldehyde, ketone, and imine receives significant research efforts from the chemical community, which leads to the identification of efficient catalytic systems based on Cu^5–16^, Zn^17,18^, Ag^19–22^, and In^23,24^. The proposed six-membered ring transition state formed by the coordination of the allyl metal species to carbonyl group allows excellent asymmetric induction. Especially, copper(I)-catalysts serve as powerful weapons to enable the highly enantioselective allylation of carbonyl groups and imines^5–16^. However, the affinity of the highly nucleophilic allylcopper(I) species to carbonyl group set up an obstacle on the conjugate allylation. For example, in the presence of 10 mol % copper(I)-(*R*)-BINAP, 2 equiv allylboronate, and 1 equiv LiO^*t*^Bu, the allylation of α,β-unsaturated ester produced tertiary alcohol only and the 1,4-conjugate allylation product was not obtained (Fig. 1a). Moreover, without the assistance of the six-membered ring transition state, there is a concern about the enantioselectivity in the conjugate allylation.

Several research group made their contributions in the challenging catalytic asymmetric conjugate allylation (Fig. 1b)^25–33^. Snapper reported a Cu(II)-BOX-catalyzed asymmetric conjugate allylation of unsaturated cyclic β-keto-esters with allylsilane^25^. In 2007, Morken and co-workers disclosed an impressive Ni-catalyzed regioselective conjugate allylation of α,β-α′,β′-di-unsaturated ketones^26^. Later, the Morken group uncovered two efficient methods to carry out the catalytic asymmetric version in excellent control of the regioselectivity with either a palladium catalyst or a nickel catalyst^27,28^. The Hoveyda group achieved a three-component reaction of 1,3-butadiene, B_2_Pin_2_ and alkylidenemalonates in high yields with excellent enantioselectivity^29^. However, aliphatic substituents were not well tolerated at the β-position. The same group also succeeded in the catalytic enantioselective 1,6-conjugate allylation of α,β,γ,δ-unsaturated diesters with B_2_Pin_2_ and allenes^30,31^. In 2011, the Feng group achieved an asymmetric conjugate allylation of activated unsaturated lactones with a bimetallic catalytic system^32^. In 2019, the same group reported a formal catalytic asymmetric 1,4-allylation of β,γ-unsaturated α-ketoesters^4^. In fact, the formal conjugate allylation was enabled by the allylation of the ketone group and the following oxy-Cope rearrangement. Unfortunately, alkyl was not well tolerated at the γ-position as only moderate enantioselectivity was observed. Moreover, Shibasaki and Kumagai uncovered a catalytic asymmetric conjugate allylation of α,β-unsaturated thioamides with allyl cyanide under proton-transfer conditions^33^. In view of the above achievements, we are interested in developing a catalytic asymmetric conjugate 1,6-allylation with more general substrate structure and broader substrate scope.

Copper(I)-catalyzed asymmetric 1,6-addition with alkyl metal reagents (such as organozinc reagent and Grignard reagent) has been reported as a powerful tool to regio-selectively construct carbon-carbon bonds^34–46^. Herein, we disclose an asymmetric 1,6-conjugate allylation of 2,2-dimethyl-6-alkenyl-4*H*-1,3-dioxin-4-one with a copper(I)-NHC catalyst (Fig. 1c). The 2,2-dimethyl-4*H*-1,3-dioxin-4-one moiety is an equivalent of the synthetically versatile β-keto-ester group and the product containing both an allyl group and a dioxinone group allows further structure elaboration. Furthermore, in view of the bulky steric hindrance around the carbonyl group and the relative stability of the lactone moiety, it is envisioned that the highly nucleophilic allylcopper(I) species would not touch the carbonyl group in the dioxinone and thus would attack the less hindered conjugate carbon-carbon double bond to give the desired 1,6-allylation.

## Results

### Conditions optimization

First of all, the catalytic asymmetric conjugate allylation of (*E*)-2,2-dimethyl-6-(4-phenylbut-1-en-1-yl)-4*H*-1,3-dioxin-4-one (**1a**) with bench-stable allylboronate **2** was studied in the presence of 5 mol % Cu(CH_3_CN)_4_PF_6_, 6 mol % (*R*)-BINAP, and 1 equiv LiO^*t*^Bu (Table 1, entry 1). The conjugate allylation proceeded smoothly to afford product **3a** in 25% yield with 9% ee. Then, screening of commercially available bisphosphine ligands was performed and proved to be fruitless (entries 2-6). Especially, (*R*,*R*)-Ph-BPE, the previously reported best ligand for the copper(I)-catalyzed allylation of ketones^9,11,13^, only led to 38% ee (entry 3). Moreover, ferrocene-embedded bisphosphine ligands, such as (*R*,*R*_*P*_)-TANIAPHOS and (*R*)-(*S*)-JOSIPHOS, were not effective either (entries 5-6). Obviously, copper(I)-bisphosphine catalyst did not suit this conjugate 1,6-allylation.

**Table 1:** Optimization of reaction conditions^a^.


Entry	Ligand	T	Yield^b^	ee (%)^c^
1	(*R*)-BINAP	rt	25	9
2	(*R*)-DTBM-SEGPHOS	rt	44	20
3	(*R*,*R*)-Ph-BPE	rt	58	38
4	(*R*,*R*)-QUINOXP*	rt	trace	–
5	(*R*,*Rp*)-TANIAPHOS	rt	20	2
6	(*R*)-(*S*)-JOSIPHOS	rt	46	23
7	NHC-L1	rt	14	0
8	NHC-L2	rt	12	8
9	NHC-L3	rt	10	64
10	NHC-L4	rt	31	90
11	NHC-L5	rt	54	82
12^d^	NHC-L4	rt	50	90
13^d,e^	NHC-L4	rt	85	89
14^d,e^	NHC-L4	−20	53	95
15^d,f^	NHC-L4	−20	84	94

^a^**1a**: 0.1 mmol, **2**: 0.2 mmol.

^b^Determined by ^1^H NMR analysis of reaction crude mixture using mesitylene as an internal standard.

^c^Determined by chiral-stationary-phase HPLC analysis.

^d^10 mol % Cu(CH_3_CN)_4_PF_6_ and 12 mol % NHC-L4 were used.

^e^2 equiv LiO^*t*^Bu were employed.

^f^3 equiv **2** and 3 equiv LiO^*t*^Bu were employed.

Then, we turned our attention to NHC ligands (Table 1). Five NHC ligands were synthesized according to literature methods^47–49^. NHC-L1 was completely ineffective to get asymmetric induction in this 1,6-conjugate allylation (entry 7). A small but promising 8% ee was obtained in the reaction with NHC-L2 (entry 8). Since phenol-containing NHCs (including NHC-L3-L5) were identified as powerful ligands in some copper(I)-catalyzed enantioselective reactions by the Sawamura Group^49–52^, NHC-L3 was tried in our reaction, which provided **3a** in 10% yield with 64% ee (entry 9). To our joy, 90% ee was observed with NHC-L4 (entry 10). However, decreased enantioselectivity (82% ee) was obtained in the reaction with bulkier NHC-L5 (entry 11). The yield was increased to 50% by using 10 mol % copper(I) salt and 12 mol % NHC-L4 (entry 12). By increasing the amount of LiO^*t*^Bu from 1 equiv to 2 equiv, the yield was further increased to 85% with 89% ee (entry 13). Performing the reaction at −20 °C resulted in increased enantioselectivity (95% ee) but with decreased yield (53%) (entry 14). The yield was enhanced to 84% yield with 94% ee at −20 °C by using 3 equiv allylboronate and 3 equiv LiO^*t*^Bu (entry 15).

### Substrate scope

**Fig. 2:**
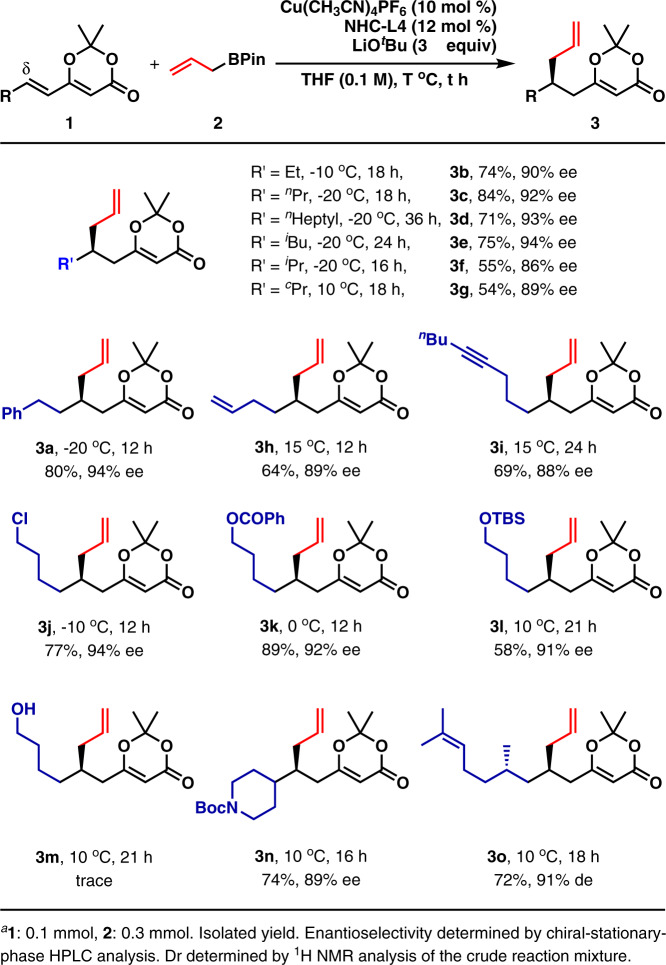
Substrate scope of (*E*)-2,2-dimethyl-6-alkyl-4*H*-1,3-dioxin-4-ones in the 1,6-conjugate allylation^a^. Various aliphatic substituents at δ-position are studied.

With the optimized reaction conditions in hand, the substrate scope of (*E*)-2,2-dimethyl-6-alkyl-4*H*-1,3-dioxin-4-ones was studied (Fig. 2). Linear alkyls, including ethyl (**3b**), ^*n*^propyl (**3c**), and ^*n*^heptyl (**3d**), were well tolerated and the corresponding products were isolated in good yields with high enantioselectivity. β-Branched alkyl (^*i*^butyl) (**3e**) was also accepted at the δ-position. The substrates bearing a α-branched alkyl with bigger steric hindrance (**3f** and **3g**), afforded the allylated products in moderate yields and slightly decreased enantioselectivity. Then, substrates with an alkyl containing a functional group, such as benzyl (**3a**), terminal alkene (**3h**), internal alkyne (**3i**), alkyl chloride (**3j**), ester (**3k**), TBS-ether (**3l**), and *N*-Boc (**3n**) were examined. To our joy, the products were obtained in moderate to high yields and high enantioselectivity. Notably, alkyl chloride and ester group were not touched by the nucleophilic allylcopper-NHC species, demonstrating that allylcopper-NHC species was less nucleophilic than allylcopper-bisphosphine species. Unfortunately, the substrate containing a free alcohol (**3m**) was not tolerated. A substrate with a preexisting chiral center (**3o**) was also studied. The allylated product was generated in 72% yield with 91% de, indicating that the asymmetric induction was mainly controlled by the copper(I) catalyst. It should be noted that in some cases, the reaction temperature was increased to get good yields.

**Fig. 3:**
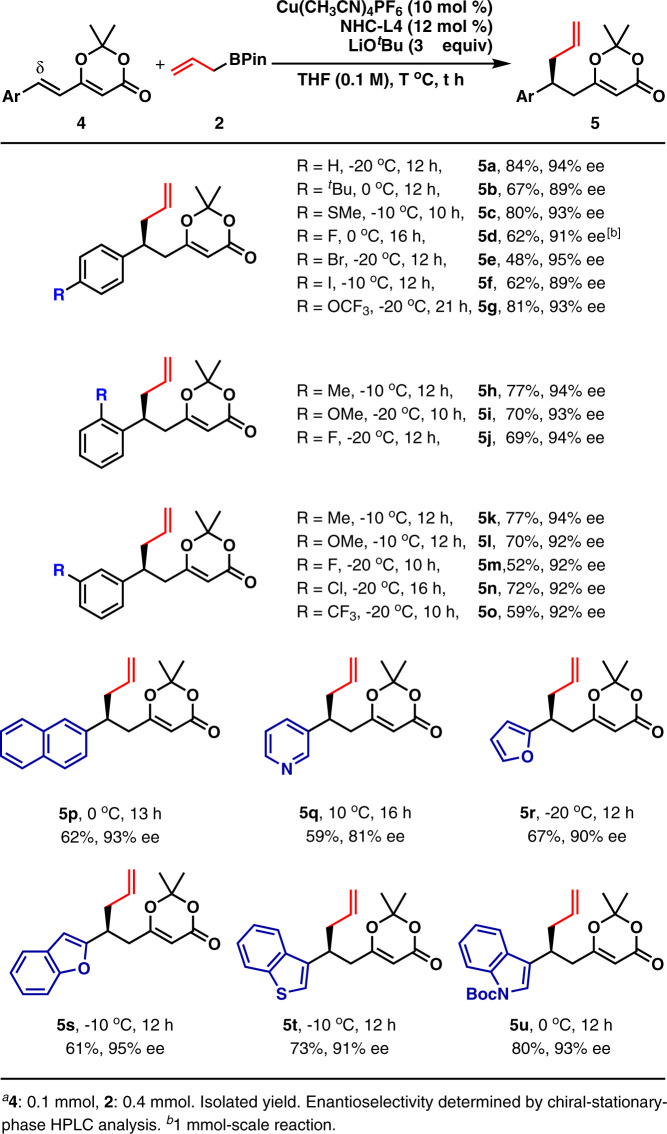
Substrate scope of (*E*)-2,2-dimethyl-6-aryl-4*H*-1,3-dioxin-4-ones in the 1,6-conjugate allylation^a^. Various aromatic substituents at δ-position are studied.

The reaction conditions were applied to the catalytic asymmetric allylation of (*E*)-2,2-dimethyl-6-aryl-4*H*-1,3-dioxin-4-ones with 4 equiv allylboronate (**2**) as 3 equiv **2** generally resulted in inferior yields (Fig. 3). The reaction was not very sensitive to the position of a substituent on the phenyl ring. As the allylated products containing a *para*-substituent, *ortho*-substituent, or *meta*-substituent were isolated in moderate to high yields with uniformly high enantioselectivity (**5a**–**5o**). It was noted that substrates with electron-withdrawing groups led to lower yields but with maintained enantioselectivity (**5d**–**5f**, **5** **m**, and **5o**). Moreover, substrates with electron-donating groups served as competent substrates as the corresponding products were furnished in good yields with high enantioselectivity (**5b**–**5c**, **5h**–**5i**, and **5k**–**5l**).

The phenyl group at δ-position was successfully changed to 2-naphthyl group without affecting both yield and enantioselectivity significantly (**5p**). Moreover, several heteroaryl groups, including 3-pyridyl (**5q**), 2-furanyl (**5r**), 2-benzofuranyl (**5s**), 3-benzothienyl (**5t**), and 3-*N*-Boc-indolyl (**5u**), were successfully tolerated at the δ-position. The corresponding products were furnished in moderate yields with uniformly high enantioselectivity. It should be pointed out that the reaction temperature varied in order to get good yields. The absolute configuration of **5a** was determined to be *S* by its transformation to a known compound (for the details, see SI). The absolute configurations of other products (**3** and **5**) were deduced by analogy.

**Fig. 4:**
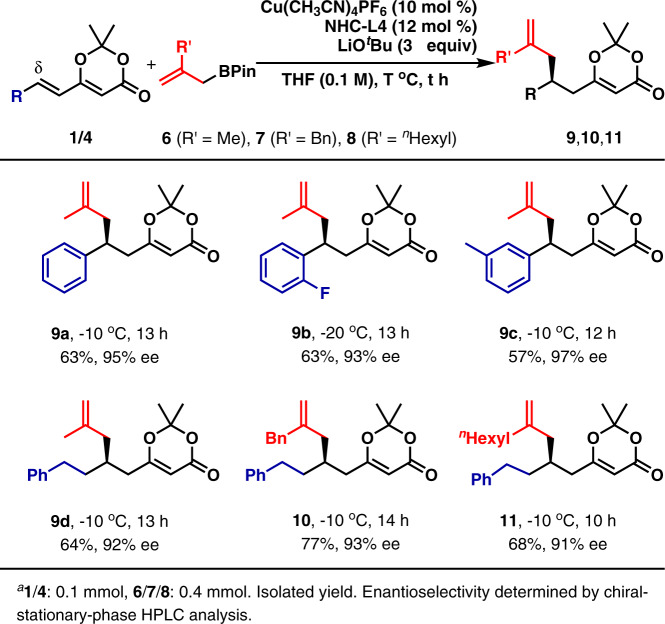
Preliminary investigation of 1,6-conjugate allylation with 2-substituted allylboronates (6–8)^a^. Both aromatic and aliphatic substituents at δ-position are tried.

**Fig. 5:**
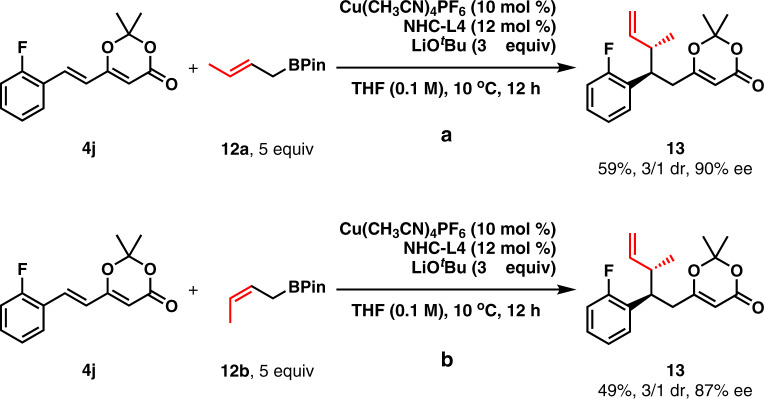
Diastereoselective 1,6-conjugate allylation. **a** The 1.6-conjugate allylation with (*E*)-allylBPin. **b** The 1,6-conjugate allylation with (*Z*)-allylBPin.

Then, the 1,6-conjugate allylation with 2-substituted allylboronates (**6**–**8**) was investigated as shown in Fig. 4. Several aryl groups, including phenyl, 2-F-phenyl, and 3-methyl-phenyl, were well tolerated at the δ-position in the reaction with **6**. The corresponding products (**9a**–**9c**) were obtained in 57%-63% yield with 93%-97% ee. An alkyl group, such as 2-phenyl-ethyl, was also acceptable at the δ-position (**9d**). 2-Methyl group in allylboronate **6** was successfully extended to 2-benzyl and ^*n*^hexyl without eroding both yields and enantioselectivity (**10**–**11**). Moreover, the reactions of both 3-methyl-(*E*)-allylboronate (**12a**) and 3-methyl-(*Z*)-allylboronate (**12b**) were studied as shown in Fig. 5. The diastereoselective allylation of **4j** and **12a** proceeded smoothly to afford **13** in moderate yield with moderate diastereoselectivity and excellent enantioselectivity. Surprisingly, the reaction with **12b** also furnished **13** as the product in decreased yield and slightly decreased enantioselectivity. At this stage, it is difficult to understand such experimental results. However, it is speculated that the addition of the (Z)-allylcopper(I) is kinetically unfavored and the isomerization of (Z)-allylcopper(I) species to (*E*)-allylcopper(I) might occur through 1,3-translocation in the present reaction conditions^53^. The absolute configurations of **9**, **10**, and **11** were deduced analogically based on the stereochemical structure of **5a**. Moreover, the absolute configurations of the two stereogenic carbon centers in **13** were determined by its transformation (For the details, see SI). In addition, the present catalytic system was extended to the asymmetric additions with PhMgBr and EtMgBr. However, only racemic products were obtained^54^.

### Demonstration of the importance of the phenol group in NHC ligands

**Fig. 6:**
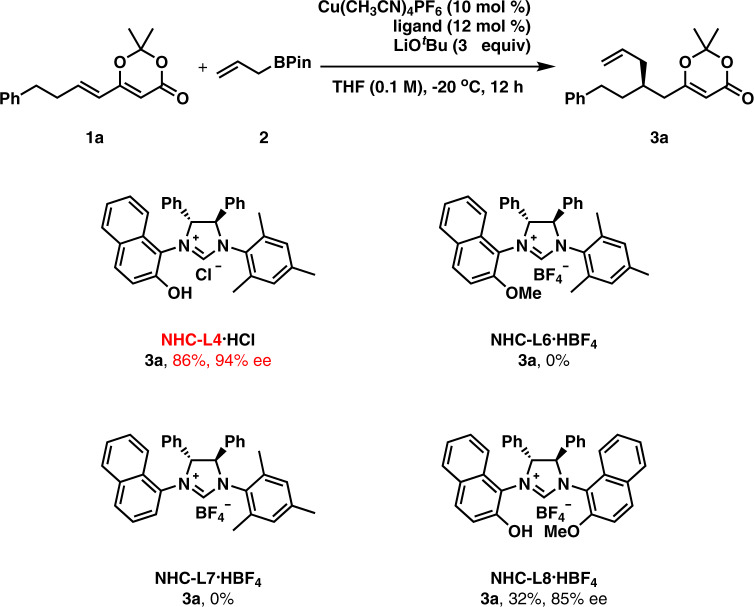
Control experiments with different NHC-ligands. The importance of the presence of a phenol group in NHC-ligands is demonstrated.

Several ligand variants of NHC-L4 were prepared to investigate the importance of the naphthol group (Fig. 6). The allylation with NHC-L6 containing a protected naphthol group did not afford the product **3a** at all. Moreover, the reaction using NHC-L7 without the naphthol moiety was fruitless. Interestingly, NHC-L8 bearing a naphthol group and a protected naphthol group was found as a good ligand as product **3a** was generated in 32% yield with 85% ee. These control experiments demonstrate that a free naphthol moiety is indispensable for this reaction to proceed. Furthermore, the steric hindrances on the both aryls are responsible for the asymmetric induction. Our finding of the essentiality of the free phenol or naphthol in this type of NHC ligands in asymmetric catalysis with copper(I) is in accordance with Sawamura’s original findings^49–52^.

### Transformation

**Fig. 7:**
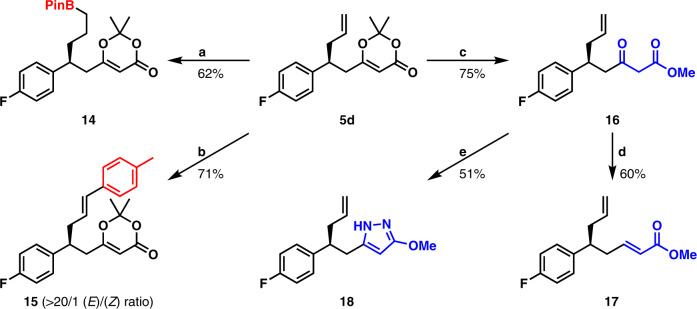
Transformations of product 5d. **a** Ir-catalyzed hydroboration. [Ir(COD)Cl]_2_ (5 mol %), dppm (Ph_2_PCH_2_PPh_2_) (10 mol %), HBPin (2 equiv), CH_2_Cl_2_, rt. **b** Olefin Metathesis. Hoveyda-Grubbs II catalyst (10 mol %), 4-methylstyrene (2 equiv), CH_2_Cl_2_, 40 °C. **c** Transformation to β-keto-ester. MeOH, K_2_CO_3_, rt. **d** Transformation to α,β-unsaturated ester. (1) NaBH_4_, MeOH, 0 °C; (2) MsCl, Et_3_N, CH_2_Cl_2_, 0 °C to rt. **e** Transformation to pyrazole. NH_2_NH_2_•HCl (2 equiv), MeOH, reflux.

At last, transformations of **5d** were performed as described in Fig. 7. An Ir-catalyzed hydroborylation of terminal olefin moiety in **5d** afforded boronate **14** in 62% yield^55^. The olefin-metathesis between **5d** and 4-methylstyrene with 10 mol % Hoveyda-Grubbs catalyst 2nd generation produced (*E*)-olefin **15** in 71% yield at 40 °C (>20/1 (*E*) form/(*Z*) form ratio). Removal of the propylidene group in **5d** was accomplished in MeOH to give β-keto-ester **16** in 75% yield. Then, a synthetic sequence, including the reduction of ketone unit, the formation of a mesylate, and the subsequent elimination furnished α,β-unsaturated ester **17** in 60% total yield. It should be noted that **16** serves as a formal 1,4-conjugate allylation product of α,β-unsaturated ketone and **17** serves as a formal 1,6-conjugate allylation product of α,β,γ,δ-unsaturated ester, which are difficult to access by known methods. Moreover, the preparation of pyrazole **18** was achieved by means of the β-keto-ester motif through a reported procedure^56^.

## Discussion

In summary, a catalytic asymmetric 1,6-conjugate allylation was achieved in moderate to high yields with high enantioselectivity. NHC ligands containing a phenolic hydroxyl group were found to be indispensable to enable this reaction. Both 2,2-dimethyl-6-alkenyl-4*H*-1,3-dioxin-4-one and allylboronate enjoyed broad substrate scopes. Several functional groups, especially alkyl halide and ester, were well tolerated in this reaction. The allyl group in the product allowed facile both hydroboration and olefin metathesis to give synthetically useful products. Moreover, the versatile dioxinone group was easily transformed to β-keto-ester moiety and α,β-unsaturated ester moiety, which generated a formal 1,4-conjugate allylation product of α,β-unsaturated ketone and a formal 1,6-conjugate allylation product of α,β,γ,δ-unsaturated ester. Detailed investigations of the mechanism are currently undertaken in our laboratory.

## Methods

### A general procedure for the catalytic asymmetric 1,6-conjugate allylation

A dried 25 ml Schlenk tube equipped with a magnetic stirring bar was charged with CuPF_6_(CH_3_CN)_4_ (3.7 mg, 0.01 mmol, 0.1 equiv), NHC-L4 (6.2 mg, 0.012 mmol, 0.12 equiv) and LiO^*t*^Bu (24.0 mg, 0.3 mmol, 3 equiv) in a glove box under Ar atmosphere. Anhydrous THF (1 ml, 0.1 M) was added to the tube via a syringe. The resulting mixture was stirred under room temperature for 7 min. Then **1** (0.1 mmol, 1 equiv) was added to the reaction mixture. It was cooled down to the stated temperature before adding **2** (50.4 mg, 0.3 mmol, 3 equiv) by a syringe. This mixture was stirred for 12–36 h at that temperature. Then the reaction was quenched by adding silica gel and the mixture was purified by flash silica gel column chromatography to give product **3**.

## Supplementary information

Supplementary Information

## Data Availability

The data supporting the findings of this study are available within the article and its Supplementary Information file. Any further relevant data are available from the authors on request.
