# Supplementary material for: Copper(I)-catalyzed asymmetric 1,6-conjugate allylation
Source: Nat Commun. 2020 Oct 30;11:5480. doi: 10.1038/s41467-020-19293-9 (PMC7603333; doi:10.1038/s41467-020-19293-9)
Supplement: Supplementary file 1 — Supplementary Information [file 41467_2020_19293_MOESM1_ESM.pdf]

Supplementary Information

Copper(I)-Catalyzed Asymmetric 1,6-Conjugate Allylation

Shi et al.

Supplementary Methods

General Information

All reagents were obtained commercially unless otherwise noted. Nuclear Magnetic Resonance (NMR) spectra were acquired on an Agilent 400 or Bruker 400 or Bruker 500 instrument. Agilent 400 and Bruker 400 are operating at 400, 101, 376 and 128 MHz for ^{1}H, ^{13}C ^{19}F and ^{11}B, respectively. Bruker 500 is operating at 500, 151, 565 and 193 MHz for ^{1}H, ^{13}C ^{19}F and ^{11}B, respectively. For ^{1}H NMR, chemical shifts were reported in δ ppm referenced to an internal standard ($SiMe_4$: 0.00 ppm). For ^{13}C NMR, chemical shifts were reported in the scale relative to NMR solvent ($CDCl_3$: 77.00 ppm) as an internal reference. For ^{19}F NMR, chemical shifts were reported in δ ppm referenced to an external standard ($CFCl_3$: 0.00 ppm). For ^{11}B NMR, chemical shifts were reported in δ ppm referenced to an external standard (BF_3-Et_2O: 0.00 ppm). Multiplicities are reported using the following abbreviations: s = singlet, d = doublet, t = triplet, q = quartet, m = multiplet, br = broad signal. Mass spectra (ESI) were measured on Agilent Technologies 1100 Series LC-MS. High-resolution mass spectra (ESI) were measured on Thermo Scientific LTQ FT Ultra FT-MS. Infrared (IR) spectra were recorded on Thermo Scientific Nicolet iS5 FT-IR. Optical rotation was measured using a 1 mL cell with a 1.0 dm path length on a JASCO P-1030 polarimeter. HPLC analysis was conducted on a Shimadzu HPLC system equipped with Daicel chiral-stationary-phase columns (ϕ 4.6 mm × 250 mm).

General procedure A (GPA)

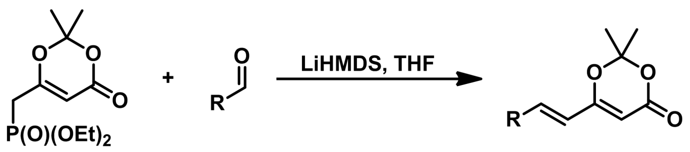

To a solution of LiHMDS (1.8 M in THF, 1.8 mmol, 1.0 equiv) in anhydrous THF (10 ml) was added (2,2-dimethyl-4-oxo-4*H*-1,3-dioxin-6-yl)methyl diethyl phosphite (500 mg in 4 ml THF, 1.8 mmol, 1.0 equiv) at 0 °C by a syringe. After being stirred for 40 minutes at 0 °C, it was cooled down to -78 °C. Then aldehyde (2 mmol in 3 ml THF, 1.1 equiv) was added slowly to the mixture. The solution was stirred for 10 h at -78 °C and then the reaction temperature was allowed to rise to 25 °C. the reaciton mixture was filtered on Celite. The solvent in filtrate was removed to give the crude, which was purified by silica gel chromatography to give the pure product[1] (PE:EA = 10:1).

General procedure B (GPB)

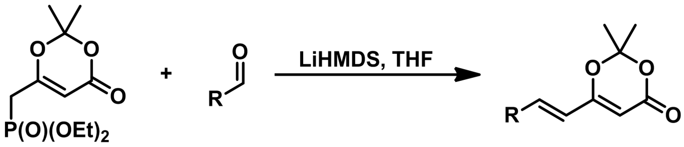

To a solution of LiHMDS (1.8 M in THF, 1.8 mmol, 1.0 equiv) in anhydrous THF (10 ml) was added (2,2-dimethyl-4-oxo-4*H*-1,3-dioxin-6-yl)methyl diethyl phosphite (500 mg in 4 ml THF, 1.8 mmol, 1.0 equiv) at 0 °C by a syringe. After being stirred for 40 minutes at 0 °C, it was cooled down to -60 °C.Then aldehyde (2 mmol in 3 ml THF, 1.1 equiv) was added slowly to the mixture. The solution was stirred for 10 h at -60 °C and then the reaction temperature was allowed to rise to 25 °C. The mixture was filtered on Celite. The solvent in filtrate was removed to give the crude, which was purified by silica gel chromatography to give the pure product (PE:EA = 10:1).

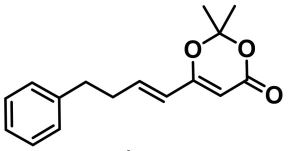

1a

(*E*)-2,2-dimethyl-6-(4-phenylbut-1-en-1-yl)-4*H*-1,3-dioxin-4-one (**1a**)
According to GPB, (9.36 mmol scale) 1.3g, white solid, 54% yield.
^{1}H NMR (400 MHz, CDCl$_3$) 7.35-7.15 (m, 5H), 6.65-6.53 (m, 1H), 5.91 (d, *J* = 15.6 Hz, 1H), 5.23 (s, 1H), 2.83-2.73 (m, 2H), 2.57-2.49 (m, 2H), 1.70 (s, 6H) ppm.
^{13}C NMR (101 MHz, CDCl$_3$) δ 163.13, 161.88, 141.17, 140.64, 128.38, 128.21, 126.10, 122.94, 106.19, 93.47, 93.42, 34.59, 34.34, 24.91 ppm.
HRMS (ESI) m/z [M+H]$^+$: calcd. 259.1329, found. 259.1328.
IR (film): ν_{max} (cm^{-1}) 3434, 3086, 3026, 1725, 1654, 1592, 1390, 1204, 1020, 969, 902, 749, 700, 512.

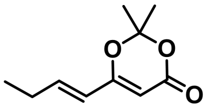

1b

(*E*)-6-(but-1-en-1-yl)-2,2-dimethyl-4*H*-1,3-dioxin-4-one (**1b**)
According to GPB, 200 mg, colourless oil, 61% yield.
^{1}H NMR (400 MHz, CDCl$_3$) 6.72-6.52 (m, 1H), 5.99-5.79 (m, 1H), 5.27 (d, *J* = 15.5 Hz, 1H), 2.32-2.18 (m, 2H), 1.71 (s, 6H), 1.08 (t, *J* = 7.4 Hz, 3H) ppm.
^{13}C NMR (101 MHz, CDCl$_3$) δ 163.49, 162.07, 143.88, 121.50, 106.17, 93.17, 25.71, 24.97, 12.41 ppm.
HRMS (ESI) m/z [M+H]$^+$: calcd. 183.1016, found. 183.1017.

IR (film): ν_{max} (cm^{-1}) 2998, 2968, 1728, 1654, 1593, 1438, 1390, 1274, 1013, 902, 854, 598.

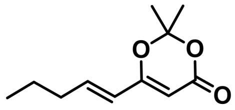

1c

(*E*)-2,2-dimethyl-6-(pent-1-en-1-yl)-4*H*-1,3-dioxin-4-one (**1c**)
According to GPB, 222 mg, yellow oil, 63% yield.
^{1}H NMR (400 MHz, CDCl$_3$) 6.63-6.50 (m, 1H), 5.90 (d, *J* = 15.6 Hz, 1H), 5.24 (s, 1H), 2.24-2.14 (m, 2H), 1.72 (s, 6H), 1.55-1.45 (m, 2H), 0.95 (t, *J* = 7.4 Hz, 3H) ppm.
^{13}C NMR (101 MHz, CDCl$_3$) δ 163.39, 162.07, 142.42, 122.53, 106.19, 93.17, 34.69, 24.97, 21.55, 13.67 ppm.
HRMS (ESI) m/z [M+H]$^+$: calcd. 197.1172, found. 197.1174.
IR (film): ν_{max} (cm^{-1}) 2961, 1728, 1654, 1390, 1274, 1206, 1018, 970, 902.

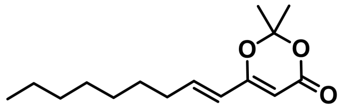

1d

(*E*)-2,2-dimethyl-6-(non-1-en-1-yl)-4*H*-1,3-dioxin-4-one (**1d**)
According to GPB, 300 mg, colourless oil, 66% yield.
^{1}H NMR (400 MHz, CDCl$_3$) 6.67-6.45 (m, 1H), 5.90 (d, *J* = 15.5 Hz, 1H), 5.24 (s, 1H), 2.25-2.14 (m, 2H), 1.71 (s, 6H), 1.49-1.41 (m, 2H), 1.30 (d, *J* = 5.6 Hz, 8H), 0.89 (t, *J* = 5.8 Hz, 3H) ppm.
^{13}C NMR (101 MHz, CDCl$_3$) δ 163.41, 162.07, 142.72, 122.31, 106.16, 93.11, 32.71, 31.66, 29.11, 29.00, 28.28, 24.95, 22.57, 14.02 ppm.
HRMS (ESI) m/z [M+H]$^+$: calcd. 253.1798, found. 253.1801.
IR (film): ν_{max} (cm^{-1}) 3507, 2998, 2927, 2856, 1728, 1651, 1594, 1462, 1390, 1206, 1019, 901, 799, 601.

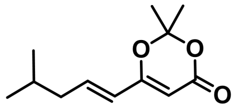

1e

(*E*)-2,2-dimethyl-6-(4-methylpent-1-en-1-yl)-4*H*-1,3-dioxin-4-one (**1e**)
According to GPB, 300 mg, colourless oil, 79% yield.
^{1}H NMR (400 MHz, CDCl$_3$) 6.59-6.49 (m, 1H), 5.89 (d, *J* = 15.5 Hz, 1H), 5.24 (s, 1H), 2.13-2.06 (m, 2H), 1.80-1.73 (m, 1H), 1.71 (s, 6H), 0.93 (d, *J* = 6.7 Hz, 6H) ppm.
^{13}C NMR (101 MHz, CDCl$_3$) δ 163.30, 162.11, 141.49, 123.46, 106.23, 93.25, 42.02, 28.09, 25.00, 22.38 ppm.
HRMS (ESI) m/z [M+H]$^+$: calcd. 211.1329, found. 211.1331.
IR (film): ν_{max} (cm^{-1}) 3517, 2998, 2957, 1731, 1652, 1593, 1273, 1019, 902, 812.

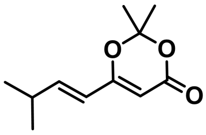

1f

(*E*)-2,2-dimethyl-6-(3-methylbut-1-en-1-yl)-4*H*-1,3-dioxin-4-one (**1f**)
According to GPB, 211 mg, pale yellow oil, 60% yield.
^{1}H NMR (400 MHz, CDCl$_3$) 6.54 (dd, *J* = 15.6, 6.8 Hz, 1H), 5.85 (d, *J* = 15.6 Hz, 1H), 5.26 (s, 1H), 2.54-2.40 (m, 1H), 1.72 (s, 6H), 1.08 (d, *J* = 6.8 Hz, 6H) ppm.
^{13}C NMR (101 MHz, CDCl$_3$) δ 163.66, 162.03, 148.67, 119.80, 106.18, 93.33, 31.29, 24.97, 21.45 ppm.
HRMS (ESI) m/z [M+H]$^+$: calcd. 197.1172, found. 197.1172.
IR (film): v_{max} (cm^{-1}) 3435, 2963, 1731, 1651, 1592, 1391, 1272, 1018, 801, 598.

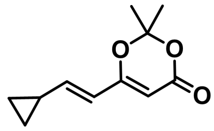

1g

(*E*)-6-(2-cyclopropylvinyl)-2,2-dimethyl-4*H*-1,3-dioxin-4-one (**1g**)
According to GPB, 170 mg, colourless oil, 49% yield.
^{1}H NMR (400 MHz, CDCl$_3$) 6.07-5.94 (m, 2H), 5.19 (s, 1H), 1.69 (s, 6H), 1.61-1.52 (m, 1H), 1.00-0.94 (m, 2H), 0.67-0.59 (m, 2H) ppm.
^{13}C NMR (101 MHz, CDCl$_3$) δ 163.22, 162.23, 147.12, 119.55, 106.08, 92.14, 24.98, 15.07, 8.85 ppm.
HRMS (ESI) m/z [M+H]$^+$: calcd. 195.1016, found. 195.1016.
IR (film): v_{max} (cm^{-1}) 2996, 2939, 1772, 1633, 1591, 1392, 1272, 1205, 1016, 721.

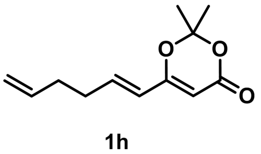

1h

(*E*)-6-(hexa-1,5-dien-1-yl)-2,2-dimethyl-4H-1,3-dioxin-4-one (**1h**)
According to GPB, 122 mg, colourless oil, 33% yield.
^{1}H NMR (400 MHz, CDCl$_3$) δ 6.62-6.51 (m, 1H), 5.92 (d, *J* = 15.6 Hz, 1H), 5.87-5.73 (m, 1H), 5.25 (s, 1H), 5.11-4.99 (m, 2H), 2.37-2.28 (m, 2H), 2.27-2.18 (m, 2H), 1.71 (s, 6H) ppm.
^{13}C NMR (101 MHz, CDCl$_3$) δ163.20, 162.02, 141.46, 137.00, 122.80, 115.52, 106.22, 93.39, 32.28, 31.93, 24.95 ppm.
HRMS (ESI) m/z [M+H]$^+$: calcd. 209.1172, found. 209.1173.
IR (film): v_{max} (cm^{-1}) 3078, 2999, 1729, 1653, 1437, 1275, 1021, 907.

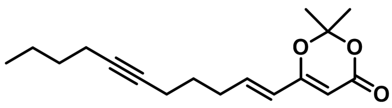

1i

(*E*)-2,2-dimethyl-6-(undec-1-en-6-yn-1-yl)-4*H*-1,3-dioxin-4-one (**1i**)
According to GPB, 214 mg, yellow oil, 42% yield.
^{1}H NMR (400 MHz, CDCl$_3$) δ 6.64-6.46 (m, 1H), 5.94 (d, *J* = 15.6 Hz, 1H), 5.25 (s, 1H), 2.36-2.29 (m, 2H), 2.21-2.14 (m, 4H), 1.71 (s, 6H), 1.66-1.63 (m, 1H), 1.48-1.38 (m, 5H), 0.92 (t, *J* = 7.1 Hz, 3H) ppm.
^{13}C NMR (101 MHz, CDCl$_3$) δ 163.13, 161.93, 141.53, 122.81, 106.12, 93.24, 81.01, 78.84, 31.53, 31.02, 27.54, 24.85, 21.78, 18.25, 18.14, 13.49 ppm.
HRMS (ESI) m/z [M+H]$^+$: calcd. 277.1798, found. 277.1799.
IR (film): ν_{max} (cm^{-1}) 2998, 2933, 1731, 1654, 1390, 1249, 1205, 1019, 902.

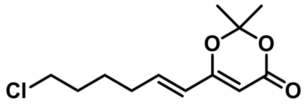

1j

(*E*)-6-(6-chlorohex-1-en-1-yl)-2,2-dimethyl-4*H*-1,3-dioxin-4-one (**1j**)
According to GPB, 233 mg, colourless oil, 52% yield.
^{1}H NMR (400 MHz, CDCl$_3$) δ 6.60-6.49 (m, 1H), 5.93 (dt, *J* = 15.5, 1.3 Hz, 1H), 5.25 (s, 1H), 3.56 (t, *J* = 6.5 Hz, 2H), 2.32-2.20 (m, 2H), 1.86-1.77 (m, 2H), 1.71 (s, 6H), 1.67-1.60 (m, 2H) ppm.
^{13}C NMR (101 MHz, CDCl$_3$) δ 163.03, 161.86, 141.37, 122.82, 106.17, 76.68, 44.53, 31.81, 31.75, 25.44, 24.88 ppm.
HRMS (ESI) m/z [M+H]$^+$: calcd. 245.0939, found. 245.0941.
IR (film): ν_{max} (cm^{-1}) 3093, 2998, 2865, 1727, 1391, 1019, 860.

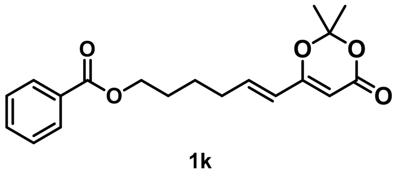

1k

(*E*)-6-(2,2-dimethyl-4-oxo-4*H*-1,3-dioxin-6-yl)hex-5-en-1-yl benzoate (**1k**)
According to GPB, 148 mg, pale yellow oil, 25% yield.
^{1}H NMR (400 MHz, CDCl$_3$) δ 8.04 (d, *J* = 7.6 Hz, 2H), 7.62-7.52 (m, 1H), 7.50-7.40 (m, 2H), 6.63-6.50 (m, 1H), 5.93 (d, *J* = 15.5 Hz, 1H), 5.25 (s, 1H), 4.35 (t, *J* = 6.3 Hz, 2H), 2.36-2.23 (m, 2H), 1.89-1.76 (m, 2H), 1.71 (s, 6H), 1.67-1.59 (m, 2H) ppm.
^{13}C NMR (101 MHz, CDCl$_3$) δ 166.52, 163.11, 161.99, 141.57, 132.90, 130.16, 129.43, 128.31, 122.86, 106.23, 93.43, 64.45, 32.20, 28.23, 24.94, 24.82 ppm.
HRMS (ESI) m/z [M+H]$^+$: calcd. 331.1540, found. 331.1540.
IR (film): ν_{max} (cm^{-1}) 3002, 2941, 2868, 1717, 1653, 1388, 1273, 1113, 1018, 970, 901, 712.

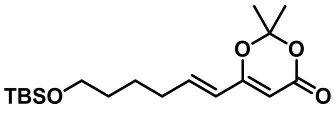

1l

(*E*)-6-(6-((tert-butyldimethylsilyl)oxy)hex-1-en-1-yl)-2,2-dimethyl-4*H*-1,3-dioxin-4-one (**1l**)
According to GPB, 478 mg, pale yellow oil, 78% yield.
^{1}H NMR (400 MHz, $CDCl_3$) δ 6.61-6.49 (m, 1H), 5.91 (d, *J* = 15.5 Hz, 1H), 5.24 (s, 1H), 3.66-3.58 (m, 2H), 2.30-2.18 (m, 2H), 1.71 (s, 6H), 1.58-1.47 (m, 4H), 0.90 (s, 9H), 0.11-0.02 (s, 6H).
^{13}C NMR (101 MHz, $CDCl_3$) δ 163.35, 162.10, 142.39, 122.56, 106.22, 93.25, 62.75, 32.44, 32.21, 25.93, 25.00, 24.66, 18.33, -5.32 ppm.
HRMS (ESI) m/z [M+H]$^+$: calcd. 341.2143, found. 341.2143.
IR (film): ν_{max} (cm^{-1}) 2929, 2857, 1731, 1654, 1389, 1273, 1099, 836.

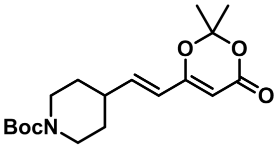

1n

(*E*)-tert-butyl-4-(2-(2,2-dimethyl-4-oxo-4*H*-1,3-dioxin-6-yl)vinyl)piperidine-1-carboxylate (**1n**)
According to GPB, 310 mg, colourless oil, 51% yield.
^{1}H NMR (400 MHz, $CDCl_3$) δ 6.47 (dd, *J* = 15.6, 6.7 Hz, 1H), 5.89 (d, *J* = 15.7 Hz, 1H), 5.27 (s, 1H), 4.14 (s, 2H), 2.86-2.67 (m, 2H), 2.36-2.24 (m, 1H), 1.80-1.65 (m, 8H), 1.46 (s, 9H), 1.41-1.26 (m, 2H).
^{13}C NMR (101 MHz, $CDCl_3$) δ 163.14, 161.87, 154.67, 144.83, 121.21, 106.34, 93.94, 79.55, 39.08, 30.88, 28.41, 25.00. ppm.
HRMS (ESI) m/z [M+Na]$^+$: calcd. 360.1781, found. 360.1779.
IR (film): ν_{max} (cm^{-1}) 2928, 1731, 1692, 1424, 1391, 1250, 1019, 867.

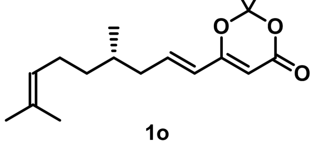

1o

(*S*,*E*)-6-(4,8-dimethylnona-1,7-dien-1-yl)-2,2-dimethyl-4*H*-1,3-dioxin-4-one (**1o**)
According to GPB, 344 mg, colourless oil, 76% yield.
^{1}H NMR (400 MHz, $CDCl_3$) 6.53 (dt, *J* = 15.5, 7.5 Hz, 1H), 5.90 (d, *J* = 15.5 Hz, 1H), 5.24 (s, 1H), 5.09 (t, *J* = 6.8 Hz, 1H), 2.28-2.17 (m, 1H), 2.08-1.95 (m, 3H), 1.71 (s, 6H), 1.69 (s, 3H), 1.61 (s, 3H), 1.45-1.10 (m, 3H), 0.91 (d, *J* = 6.7 Hz, 3H) ppm.
^{13}C NMR (101 MHz, $CDCl_3$) δ 163.26, 162.05, 141.39, 131.49, 124.32, 123.55, 106.21, 93.20, 40.21, 36.71, 32.37, 25.68, 25.46, 25.00, 24.98, 19.51, 17.62 ppm.
HRMS (ESI) m/z [M+H]$^+$: calcd. 279.1955, found. 279.1957.

IR (film): ν_{max} (cm^{-1}) 2972, 2916, 1731, 1651, 1594, 1455, 1390, 1273, 865, 803.
Optical rotation: $[\alpha]_D^{25}$ = 4.89 (c = 1.00, $CHCl_3$)

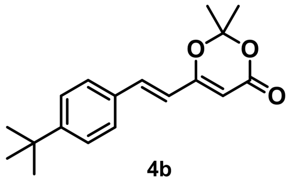

4b

(*E*)-6-(4-(*tert*-butyl)styryl)-2,2-dimethyl-4*H*-1,3-dioxin-4-one (**4b**)
According to GPA, 271 mg, yellow solid, 53% yield.
^{1}H NMR (400 MHz, $CDCl_3$) δ 7.47-7.39 (m, 4H), 7.28 (d, J = 16.7 Hz, 1H), 6.51 (d, J = 15.9 Hz, 1H), 5.42 (s, 1H), 1.77 (s, 6H), 1.33 (s, 9H) ppm.
^{13}C NMR (101 MHz, $CDCl_3$) δ 163.55, 161.96, 153.55, 137.67, 131.99, 127.56, 125.92, 118.82, 106.40, 94.57, 34.87, 31.14, 25.10 ppm.
HRMS (ESI) m/z $[M+H]^+$: calcd. 287.1642, found. 287.1642.
IR (film): ν_{max} (cm^{-1}) 2965, 1715, 1634, 1588, 1389, 1374, 1274, 1020, 981, 831.

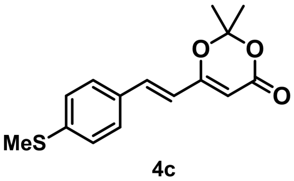

4c

(*E*)-2,2-dimethyl-6-(4-(methylthio)styryl)-4*H*-1,3-dioxin-4-one (**4c**)
According to GPA, 180 mg, pale yellow solid, 36% yield.
^{1}H NMR (400 MHz, $CDCl_3$) δ 7.42 (d, J = 8.4 Hz, 2H), 7.28-7.21 (m, 3H), 6.49 (d, J = 15.9 Hz, 1H), 5.41 (s, 1H), 2.51 (s, 3H), 1.77 (s, 6H) ppm.
^{13}C NMR (101 MHz, $CDCl_3$) δ 163.38, 161.91, 141.56, 137.15, 131.25, 128.05, 126.00, 118.66, 106.41, 94.64, 25.10, 15.14 ppm.
HRMS (ESI) m/z $[M+H]^+$: calcd. 277.0893, found. 277.0893.
IR (film): ν_{max} (cm^{-1}) 3055, 2999, 2924, 1715, 1632, 1583, 1330, 1093, 976, 903, 815, 739, 518.

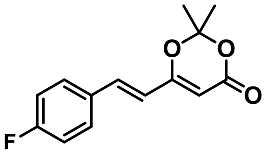

4d

(*E*)-6-(4-fluorostyryl)-2,2-dimethyl-4*H*-1,3-dioxin-4-one (**4d**)
According to GPA, 200 mg, white solid, 45% yield.
^{1}H NMR (400 MHz, $CDCl_3$) δ 7.54-7.46 (m, 2H), 7.31-7.22 (m, 1H), 7.12-7.05 (m, 2H), 6.47 (d, J = 15.9 Hz, 1H), 5.43 (s, 1H), 1.77 (s, 6H) ppm.
^{13}C NMR (101 MHz, $CDCl_3$) δ 164.87, 162.44 (d, J = 135.3 Hz), 162.37, 136.40, 131.06 (d, J = 3.4 Hz), 129.52 (d, J = 8.5 Hz), 119.45 (d, J = 2.4 Hz), 116.09 (d, J = 22.0 Hz), 106.49, 95.00, 25.08 ppm.
^{19}F NMR (376 MHz, $CDCl_3$) δ -109.95 ppm.

HRMS (ESI) m/z [M+H]$^+$: calcd. 249.0921, found. 249.0922.
IR (film): ν_{max} (cm^{-1}) 3094, 3031, 1712, 1639, 1595, 1386, 1159, 829, 646, 484.

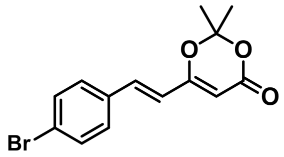

4e

(*E*)-6-(4-bromostyryl)-2,2-dimethyl-4*H*-1,3-dioxin-4-one (**4e**)
According to GPA, 210 mg, white solid, 38% yield.
^{1}H NMR (400 MHz, CDCl$_3$) δ 7.50 (d, *J* = 8.2 Hz, 2H), 7.36 (d, *J* = 8.2 Hz, 2H), 7.23 (d, *J* = 15.5 Hz, 1H), 6.52 (d, *J* = 15.9 Hz, 1H), 5.43 (s, 1H), 1.76 (s, 6H) ppm.
^{13}C NMR (101 MHz, CDCl$_3$) δ 162.84, 161.68, 136.23, 133.46, 131.95, 128.96, 123.91, 120.12, 106.41, 95.16, 24.89 ppm.
HRMS (ESI) m/z [M+H]$^+$: calcd. 309.0121, found. 309.0121.
IR (film): ν_{max} (cm^{-1}) 3063, 2994, 1717, 1635, 1388, 1250, 1019, 1009, 817.

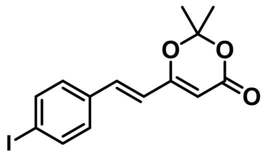

4f

(*E*)-6-(4-iodostyryl)-2,2-dimethyl-4*H*-1,3-dioxin-4-one (**4f**)
According to GPA, 300 mg, yellow solid, 47% yield.
^{1}H NMR (400 MHz, CDCl$_3$) δ 7.73 (d, *J* = 8.3 Hz, 2H), 7.27-7.18 (m, 3H), 6.55 (d, *J* = 15.9 Hz, 1H), 5.45 (s, 1H), 1.77 (s, 6H) ppm.
^{13}C NMR (101 MHz, CDCl$_3$) δ 162.82, 161.63, 138.06, 136.43, 134.15, 129.12, 120.36, 106.51, 96.00, 95.41, 25.05 ppm.
HRMS (ESI) m/z [M+H]$^+$: calcd. 356.9982, found. 356.9983.
IR (film): ν_{max} (cm^{-1}) 2996, 1720, 1635, 1579, 1389, 1202, 1059, 1019, 815.

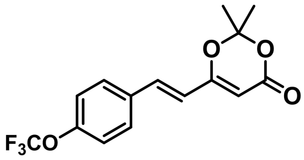

4g

(*E*)-2,2-dimethyl-6-(4-(trifluoromethoxy)styryl)-4*H*-1,3-dioxin-4-one (**4g**)
According to GPA, 250 mg, white solid, 44% yield.
^{1}H NMR (400 MHz, CDCl$_3$) δ 7.55 (d, *J* = 8.2 Hz, 2H), 7.33-7.20 (m, 3H), 6.54 (d, *J* = 15.9 Hz, 1H), 5.47 (s, 1H), 1.78 (s, 6H) ppm.
^{13}C NMR (101 MHz, CDCl$_3$) δ 162.79, 161.58, 149.90, 135.82, 133.29, 129.01, 121.06, 120.51, 120.22 (q, *J* = 257.9 Hz), 106.48, 95.37, 24.90 ppm.
^{19}F NMR (376 MHz, CDCl$_3$) δ -57.80.
HRMS (ESI) m/z [M+H]$^+$: calcd. 315.0839, found. 315.0839.
IR (film): ν_{max} (cm^{-1}) 3097, 2989, 1717, 1636, 1507, 1390, 1257, 1213, 1167, 1018.

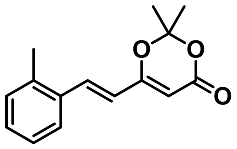

4h

(E)-2,2-dimethyl-6-(2-methylstyryl)-4H-1,3-dioxin-4-one (**4h**)
According to GPA, 260 mg, yellow solid, 59% yield.
^{1}H NMR (400 MHz, $CDCl_3$) δ 7.59-7.50 (m, 2H), 7.32-7.16 (m, 3H), 6.46 (d, J = 15.8 Hz, 1H), 5.44 (s, 1H), 2.43 (s, 3H), 1.78 (s, 6H) ppm.
^{13}C NMR (101 MHz, $CDCl_3$) δ 163.39, 161.78, 137.18, 135.22, 133.64, 130.75, 129.66, 126.35, 125.93, 120.71, 106.41, 94.84, 25.04, 19.67 ppm.
HRMS (ESI) m/z [M+H]$^+$: calcd. 245.1172, found. 245.1172.
IR (film): ν_{max} (cm^{-1}) 2997, 1724, 1633, 1389, 1274, 1019, 903, 967, 804, 755.

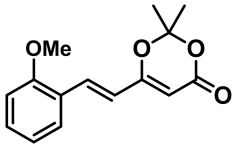

4i

(E)-6-(2-methoxystyryl)-2,2-dimethyl-4H-1,3-dioxin-4-one (**4i**)
According to GPA, 295 mg, yellow solid, 63% yield.
^{1}H NMR (400 MHz, $CDCl_3$) δ 7.59 (d, J = 16.1 Hz, 1H), 7.54-7.46 (m, 1H), 7.40-7.30 (m, 1H), 7.04-6.89 (m, 2H), 6.65 (d, J = 16.1 Hz, 1H), 5.41 (s, 1H), 3.91 (s, 3H), 1.77 (s, 6H). ppm.
^{13}C NMR (101 MHz, $CDCl_3$) δ 163.97, 162.07, 157.99, 133.13, 131.14, 128.41, 123.61, 120.76, 120.23, 111.10, 106.32, 94.42, 55.45, 25.05 ppm.
HRMS (ESI) m/z [M+H]$^+$: calcd. 261.1121, found. 261.1123.
IR (film): ν_{max} (cm^{-1}) 2998, 1719, 1630, 1438, 1247, 1050, 1019, 753.

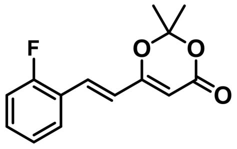

4j

(E)-6-(2-fluorostyryl)-2,2-dimethyl-4H-1,3-dioxin-4-one (**4j**)
According to GPA, 230 mg, white solid, 51% yield.
^{1}H NMR (400 MHz, $CDCl_3$) δ 7.59-7.50 (m, 1H), 7.42 (d, J = 16.0 Hz, 1H), 7.37-7.28 (m, 1H), 7.21-7.04 (m, 2H), 6.65 (d, J = 16.0 Hz, 1H), 5.45 (s, 1H), 1.76 (d, J = 1.7 Hz, 6H) ppm.
^{13}C NMR (101 MHz, $CDCl_3$) δ 162.71, 161.33, 160.66 (d, J = 253.1 Hz), 131.00 (d, J = 8.7 Hz), 129.74, 128.13 (d, J = 2.7 Hz), 124.18 (d, J = 3.4 Hz), 122.34 ((d, J = 11.5 Hz)), 121.65 (d, J = 6.5 Hz), 115.71 (d, J = 21.9 Hz), 115.61, 106.17, 95.17, 24.57 ppm.
^{19}F NMR (376 MHz, $CDCl_3$) δ -120.40

HRMS (ESI) m/z [M+H]+: calcd. 249.0921, found. 249.0922.
IR (film): v_{max} (cm^{-1}) 3102, 2993, 1721, 1636, 1388, 1272, 1203, 1018.

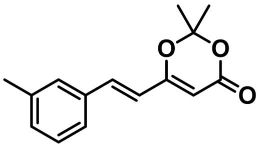

4k

(*E*)-2,2-dimethyl-6-(3-methylstyryl)-4*H*-1,3-dioxin-4-one (**4k**)
According to GPA, 200 mg, pale yellow solid, 46% yield.
^{1}H NMR (400 MHz, CDCl$_3$) 7.37-7.24 (m, 4H), 7.18 (d, *J* = 7.1 Hz, 1H), 6.53 (d, *J* = 15.9 Hz, 1H), 5.42 (s, 1H), 2.38 (s, 3H), 1.77 (s, 6H) ppm.
^{13}C NMR (101 MHz, CDCl$_3$) δ 163.40, 161.90, 138.60, 137.96, 134.67, 130.81, 128.82, 128.35, 124.97, 119.45, 106.43, 94.81, 25.09, 21.31 ppm.
HRMS (ESI) m/z [M+H]+: calcd. 245.1172, found. 245.1174.
IR (film): v_{max} (cm^{-1}) 2998, 1727, 1634, 1593, 1390, 1273, 1204, 1019, 967, 804, 696.

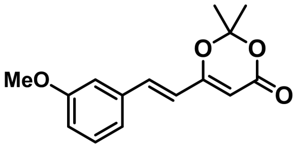

4l

(*E*)-6-(3-methoxystyryl)-2,2-dimethyl-4*H*-1,3-dioxin-4-one (**4l**)
According to GPA, 270 mg, yellow solid, 58% yield.
^{1}H NMR (400 MHz, CDCl$_3$) δ 7.37-7.20 (m, 2H), 7.10 (d, *J* = 7.7 Hz, 1H), 7.02 (d, *J* = 1.9 Hz, 1H), 6.92 (dd, *J* = 8.2, 2.3 Hz, 1H), 6.53 (d, *J* = 15.9 Hz, 1H), 5.43 (s, 1H), 3.84 (s, 3H), 1.77 (s, 6H) ppm.
^{13}C NMR (101 MHz, CDCl$_3$) δ 163.17, 161.77, 159.87, 137.62, 136.03, 129.87, 120.34, 119.92, 115.70, 112.62, 106.43, 95.01, 55.24, 25.02 ppm.
HRMS (ESI) m/z [M+H]+: calcd. 261.1121, found. 261.1121.
IR (film): v_{max} (cm^{-1}) 2998, 2942, 1722, 1636, 1597, 1390, 1274, 1204, 1019, 903, 781.

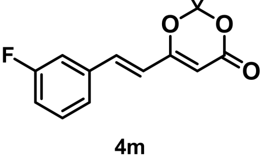

4m

(*E*)-6-(3-fluorostyryl)-2,2-dimethyl-4*H*-1,3-dioxin-4-one (**4m**)
According to GPA, 200 mg, yellow solid, 45% yield.
^{1}H NMR (400 MHz, CDCl$_3$) δ 7.41-7.33 (m, 1H), 7.30-7.23 (m, 2H), 7.23-7.17 (m, 1H), 7.11-7.03 (m, 1H), 6.54 (d, *J* = 15.9 Hz, 1H), 5.46 (s, 1H), 1.77 (s, 6H) ppm.
^{13}C NMR (101 MHz, CDCl$_3$) δ 163.04 (d, *J* = 247.1 Hz), 162.76, 161.68, 136.32 (d, *J* = 2.9 Hz), 130.48 (d, *J* = 8.4 Hz), 123.64 (d, *J* = 2.8 Hz), 121.03, 116.79 (d, *J* = 21.5

Hz), 114.00 (d, J = 22.1 Hz), 113.89, 106.62, 103.45, 95.69, 25.10 ppm.
^{19}F NMR (101 MHz, $CDCl_3$) δ -112.49.
HRMS (ESI) m/z [M+H]$^+$: calcd. 249.0921, found. 249.0922.
IR (film): ν_{max} (cm^{-1}) 3105, 3067, 2994, 1734, 1717, 1653, 1559, 1374, 1272, 1015, 968.

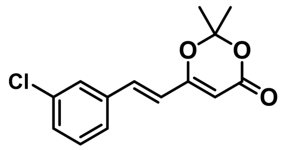

4n

(*E*)-6-(3-chlorostyryl)-2,2-dimethyl-4*H*-1,3-dioxin-4-one (**4n**)
According to GPA, 238 mg, yellow solid, 50% yield.
^{1}H NMR (400 MHz, $CDCl_3$) δ 7.47 (s, 1H), 7.42-7.29 (m, 3H), 7.22 (d, J = 15.8 Hz, 1H), 6.54 (d, J = 15.9 Hz, 1H), 5.46 (s, 1H), 1.76 (s, 6H) ppm.
^{13}C NMR (101 MHz, $CDCl_3$) δ 162.51, 161.35, 136.27, 135.77, 134.51, 129.90, 129.39, 127.15, 125.65, 120.77, 106.29, 95.36, 24.73 ppm.
HRMS (ESI) m/z [M+H]$^+$: calcd. 265.0626, found. 265.0626.
IR (film): ν_{max} (cm^{-1}) 3105, 2998, 2946, 1711, 1634, 1589, 1376, 1208, 1020, 819.

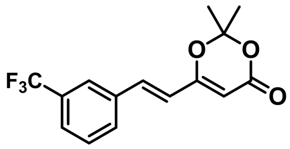

4o

(*E*)-2,2-dimethyl-6-(3-(trifluoromethyl)styryl)-4*H*-1,3-dioxin-4-one (**4o**)
According to GPA, 225 mg, yellow solid, 42% yield.
^{1}H NMR (400 MHz, $CDCl_3$) δ 7.79-7.65 (m, 2H), 7.62-7.48 (m, 2H), 7.34 (d, J = 15.9 Hz, 1H), 6.65 (d, J = 15.9 Hz, 1H), 5.51 (s, 1H), 1.78 (s, 6H) ppm.
^{13}C NMR (101 MHz, $CDCl_3$) δ 162.50, 161.42, 135.64, 135.30, 130.94 (q, J = 32.5 Hz), 130.39, 129.23, 125.85 (q, J = 3.6 Hz), 124.10 (q, J = 3.7 Hz), 123.58 (q, J = 272.5 Hz), 121.24, 106.41, 95.59, 24.62 ppm.
^{19}F NMR (376 MHz, $CDCl_3$) δ -67.91 ppm.
HRMS (ESI) m/z [M+H]$^+$: calcd. 299.0890, found. 299.0891.
IR (film): ν_{max} (cm^{-1}) 3063, 1998, 2946, 1724, 1639, 1392, 1335, 1274, 1126, 1020, 967, 903, 808, 696.

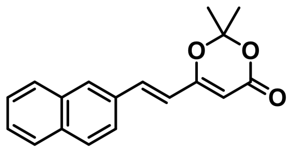

4p

(*E*)-2,2-dimethyl-6-(2-(naphthalen-2-yl)vinyl)-4*H*-1,3-dioxin-4-one (**4p**)
According to GPA, 284 mg, yellow solid, 56% yield.

^{1}H NMR (400 MHz, CDCl$_3$) δ 7.91 (s, 1H), 7.87-7.79 (m, 3H), 7.66 (dd, *J* = 8.7, 1.5 Hz, 1H), 7.57-7.42 (m, 3H), 6.66 (d, *J* = 15.9 Hz, 1H), 5.47 (s, 1H), 1.80 (s, 6H) ppm.
^{13}C NMR (101 MHz, CDCl$_3$) δ 163.33, 161.91, 137.84, 133.98, 133.31, 132.20, 129.49, 128.75, 128.42, 127.78, 127.18, 126.76, 123.21, 119.82, 106.49, 94.98, 25.12 ppm.
HRMS (ESI) m/z [M+H]$^+$: calcd. 281.1172, found. 281.1172.
IR (film): v_{max} (cm^{-1}) 3054, 3006, 2933, 1716, 1634, 1387, 1274, 1203, 1019, 815.

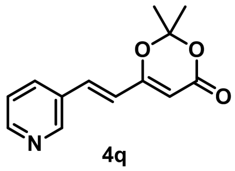

4q

(*E*)-2,2-dimethyl-6-(2-(pyridin-3-yl)vinyl)-4*H*-1,3-dioxin-4-one (**4q**)
According to GPA, 266 mg, white solid, 64% yield.
^{1}H NMR (400 MHz, CDCl$_3$) δ 8.73 (s, 1H), 8.59 (s, 1H), 7.85 (d, *J* = 7.6 Hz, 1H), 7.45-7.24 (m, 2H), 6.62 (d, *J* = 15.9 Hz, 1H), 5.49 (s, 1H), 1.79 (s, 6H) ppm.
^{13}C NMR (101 MHz, CDCl$_3$) δ 162.41, 161.52, 150.55, 149.54, 133.88, 133.59, 121.75, 106.70, 95.95, 25.08 ppm.
HRMS (ESI) m/z [M+H]$^+$: calcd. 232.0968, found. 232.0969.
IR (film): v_{max} (cm^{-1}) 3098, 1725, 1639, 1389, 1250, 1199, 1020, 826.

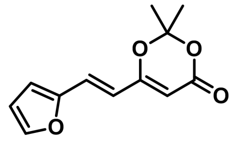

4r

(*E*)-6-(2-(furan-2-yl)vinyl)-2,2-dimethyl-4*H*-1,3-dioxin-4-one (**4r**)
According to GPA, 200 mg, yellow solid, 50% yield.
^{1}H NMR (400 MHz, CDCl$_3$) 7.49 (s, 1H), 7.06 (d, *J* = 15.6 Hz, 1H), 6.57 (d, *J* = 3.3 Hz, 1H), 6.50-6.42 (m, 2H), 5.41 (s, 1H), 1.75 (s, 6H) ppm.
^{13}C NMR (101 MHz, CDCl$_3$) δ 163.09, 161.83, 151.29, 144.59, 124.28, 117.54, 114.05, 112.42, 106.35, 94.82, 25.06 ppm.
HRMS (ESI) m/z [M+H]$^+$: calcd. 221.0808, found. 221.0809.
IR (film): v_{max} (cm^{-1}) 3115, 2995, 1708, 1629, 1606, 1379, 1201, 1018, 757, 518.

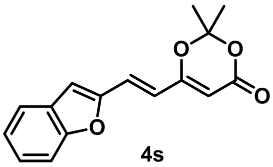

4s

(*E*)-6-(2-(benzofuran-2-yl)vinyl)-2,2-dimethyl-4*H*-1,3-dioxin-4-one (**4s**)
According to GPA, 250 mg, yellow solid, 51% yield.
^{1}H NMR (400 MHz, CDCl$_3$) δ 7.58 (d, *J* = 7.7 Hz, 1H), 7.47 (d, *J* = 8.3 Hz, 1H), 7.40-7.28 (m, 1H), 7.28-7.21 (m, 1H), 7.17 (d, *J* = 15.5 Hz, 1H), 6.88 (s, 1H), 6.70 (d,

J = 15.5 Hz, 1H), 5.48 (s, 1H), 1.76 (s, 6H) ppm.
^{13}C NMR (101 MHz, CDCl$_3$) δ 162.54, 161.64, 155.42, 152.62, 128.41, 126.32, 124.39, 123.32, 121.63, 120.40, 111.19, 110.39, 106.49, 95.78, 25.04 ppm.
HRMS (ESI) m/z [M+H]$^+$: calcd. 271.0965, found. 271.0966.
IR (film): ν_{max} (cm^{-1}) 3067, 2933, 1717, 1628, 1387, 1258, 1200, 1019, 750.

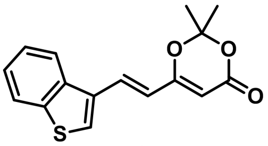

4t

(*E*)-6-(2-(benzo[*b*]thiophen-3-yl)vinyl)-2,2-dimethyl-4*H*-1,3-dioxin-4-one (**4t**)
According to GPA, 299 mg, yellow solid, 58% yield.
^{1}H NMR (400 MHz, CDCl$_3$) 7.98 (d, *J* = 8.0 Hz, 1H), 7.89 (d, *J* = 7.9 Hz, 1H), 7.72 (s, 1H), 7.56 (d, *J* = 15.9 Hz, 1H), 7.50-7.38 (m, 2H), 6.62 (d, *J* = 15.9 Hz, 1H), 5.46 (s, 1H), 1.80 (s, 6H) ppm.
^{13}C NMR (101 MHz, CDCl$_3$) δ 163.22, 161.79, 140.41, 136.98, 131.91, 129.27, 126.91, 125.05, 124.83, 123.03, 121.76, 120.33, 106.49, 94.85, 25.09 ppm.
HRMS (ESI) m/z [M+H]$^+$: calcd. 287.0736, found. 287.0739.
IR (film): ν_{max} (cm^{-1}) 2999, 1716, 1631, 1374, 1268, 1019, 780, 601.

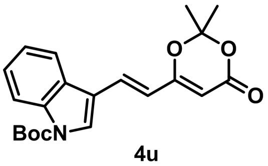

4u

(*E*)-tert-butyl-3-(2-(2,2-dimethyl-4-oxo-4*H*-1,3-dioxin-6-yl)vinyl)-1*H*-indole-1-carboxylate (**4u**)
According to GPA, 360 mg, yellow solid, 54% yield.
^{1}H NMR (400 MHz, CDCl$_3$) 8.20 (d, *J* = 8.0 Hz, 1H), 7.89-7.79 (m, 2H), 7.49-7.30 (m, 3H), 6.63 (d, *J* = 16.0 Hz, 1H), 5.43 (s, 1H), 1.79 (s, 6H), 1.69 (s, 9H) ppm.
^{13}C NMR (101 MHz, CDCl$_3$) δ 163.58, 161.98, 148.91, 136.04, 129.62, 127.91, 127.59, 125.19, 123.44, 119.89, 118.80, 116.97, 115.49, 106.26, 93.83, 84.57, 27.99, 24.99 ppm.
HRMS (ESI) m/z [M+H]$^+$: calcd. 370.1649, found. 370.1648.
IR (film): ν_{max} (cm^{-1}) 3050, 2989, 1718, 1632, 1539, 1372, 1236, 1274, 1153, 1096, 760.

General Procedure C

A dried 25 ml schlenk tube equipped with a magnetic stirring bar were charged with $CuPF_6(CH_3CN)_4$ (3.7 mg, 0.01 mmol, 0.1 equiv), (*R*)-BINAP (6.2 mg, 0.01 mmol, 0.1 equiv) and LiO^{t}Bu (8.0 mg, 0.1 mmol, 1 equiv) in a glove box under Ar atmosphere. Anhydrous THF (1 ml, 0.1M) was added to the tube via a syringe. The resulting mixture was stirred under room temperature for 15 min. Then ethyl cinnamate or (*E*)-chalcone (0.1 mmol, 1 equiv) and allylboronate (33.6 mg, 0.2 mmol, 2 equiv) was added by a syringe. This mixture was stirred for 12 h at room temperature. The yield was determined by ^{1}H NMR analysis using mesitylene as an internal standard.

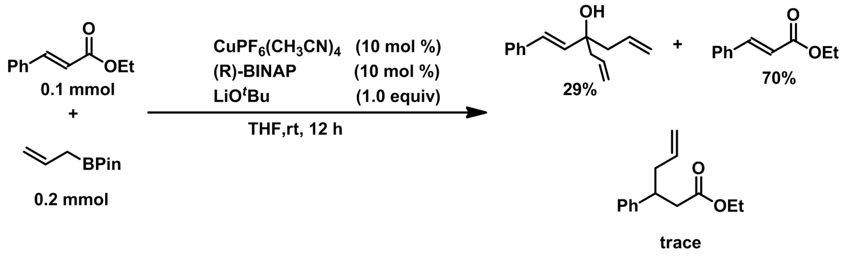

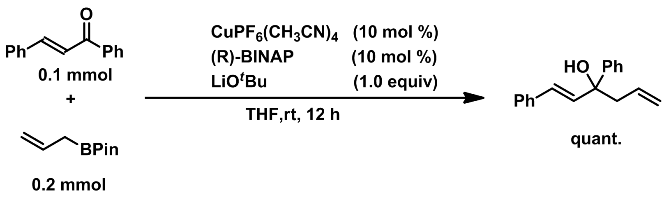

General Procedure for Copper(I)-Catalyzed Asymmetric 1,6-Conjugate Allylation

General Procedure D (GPD)

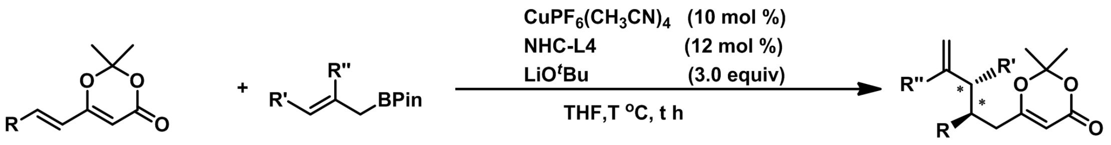

1 or 4 2,6,7 or 8 3,5,9,10,11 or 13

A dried 25 ml schlenk tube equipped with a magnetic stirring bar was charged with $CuPF_6(CH_3CN)_4$ (3.7 mg, 0.01 mmol, 0.1 equiv), NHC-L4 (6.2 mg, 0.012 mmol, 0.12 equiv) and LiO^{t}Bu (24.0 mg, 0.3 mmol, 3 equiv) in a glove box under Ar atmosphere. Anhydrous THF (1 ml, 0.1M) was added to the tube via a syringe. The resulting mixture was stirred under room temperature for 7 min. Then **1** (0.1 mmol, 1 equiv) was added to the reaction mixture. It was cooled down to the stated temperature before adding **2** (50.4 mg, 0.3 mmol, 3 equiv) by a syringe.This mixture was stirred for 12-36 h at that temperature. Then the reaction was quenched by adding silica gel and the mixture was purified by flash silica gel column chromatography to give product **3**. (petroleum ether/ethyl acetate = 7/1 with 0.5% Et_3N for **3j** and **3k**, petroleum ether/ethyl acetate = 10/1 with 0.5% Et_3N for the others).

General Procedure E (GPE)

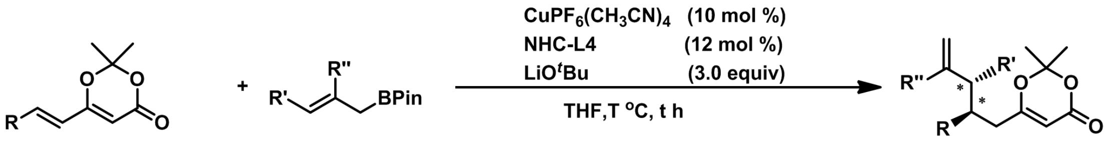

1 or 4 2,6,7 or 8 3,5,9,10,11 or 13

A dried 25 ml schlenk tube equipped with a magnetic stirring bar was charged with $CuPF_6(CH_3CN)_4$ (3.7 mg, 0.01 mmol, 0.1 equiv), NHC-L4 (6.2 mg, 0.012 mmol, 0.12 equiv) and LiO^{t}Bu (24.0 mg, 0.3 mmol, 3 equiv) in a glove box under Ar atmosphere. Anhydrous THF (1 ml, 0.1M) was added to the tube via a syringe. The resulting mixture was stirred under room temperature for 7 min. Then **4/1** (0.1 mmol, 1 equiv) was added to the reaction mixture. It was cooled down to the stated temperature before adding **2/6/7/8** (0.4 mmol, 4 equiv) by a syringe.This mixture was stirred for 10-16 h at that temperature. Then the reaction was quenched by adding silica gel and

the mixture was purified by flash silica gel column chromatography to give product **5**. (petroleum ether/ethyl acetate = 7/1 with 0.5% Et_3N).

General Procedure F (GPF)

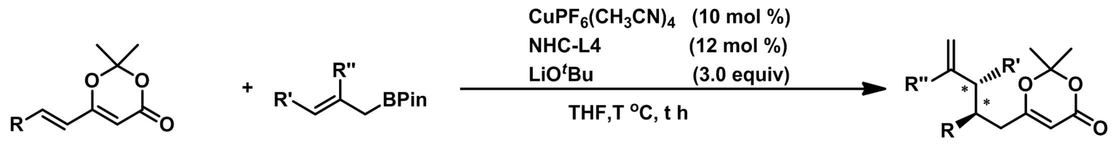

A dried 25 ml schlenk tube equipped with magnetic stirring bar was charged with $CuPF_6(CH_3CN)_4$ (3.7 mg, 0.01 mmol, 0.1 equiv), NHC-L5 (6.2 mg, 0.012 mmol, 0.12 equiv) and LiO^{t}Bu (24.0 mg, 0.3 mmol, 3 equiv) in a glove box under Ar atmosphere. Anhydrous THF (1 ml, 0.1M) was added to the tube via a syringe. The resulting mixture was stirred under room temperature for 7 min. Then **1** (0.1 mmol, 1 equiv) was added to the reaction mixture. It was cooled down to stated temperature before adding **12** (91.0 mg, 0.5 mmol, 5 equiv) by a syringe.This mixture was stirred for 12 h at 10 °C. Then the reaction was quenched by adding silica gel and the mixture was purified by flash silica gel column chromatography to give product **13**. (petroleum ether/ethyl acetate = 7/1 with 0.5% Et_3N).

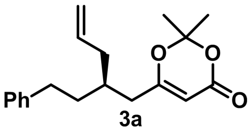

3a

(*S*)-2,2-dimethyl-6-(2-phenethylpent-4-en-1-yl)-4*H*-1,3-dioxin-4-one (**3a**)
According to GPD, 24 mg, pale yellow oil, 80% yield, 94% ee.
^{1}H NMR (400 MHz, $CDCl_3$) δ 7.33-7.25 (m, 2H), 7.22-7.11 (m, 3H), 5.80-5.68 (m, 1H), 5.22 (s, 1H), 5.14-5.01 (m, 2H), 2.72-2.56 (m, 2H), 2.32-2.07 (m, 4H), 1.89-1.79 (m, 1H), 1.73-1.58 (m, 8H) ppm.
^{13}C NMR(101 MHz, $CDCl_3$) δ 170.90, 161.11, 141.82, 135.36, 128.40, 128.26, 125.90, 117.43, 106.28, 94.47, 37.77, 37.47, 34.98, 34.53, 32.80, 25.06, 25.02 ppm.
HRMS (ESI) m/z $[M+H]^+$: calcd. 301.1798, found. 301.1798.
IR (film): ν_{max} (cm^{-1}) 3026, 2924, 2856, 1730, 1389, 1252, 1204, 1014, 803, 700
Optical rotation: $[\alpha]_D^{25}$ = 2.43 (*c* = 1.00, $CHCl_3$).
HPLC: DAICEL CHIRALPAK OD-H, hexane/*i*-PrOH = 9/1, flow rate: 0.5 mL/min, λ = 254 nm, t_R(minor) = 15.2 min, t_R(major) = 15.9 min, ee = 94%.

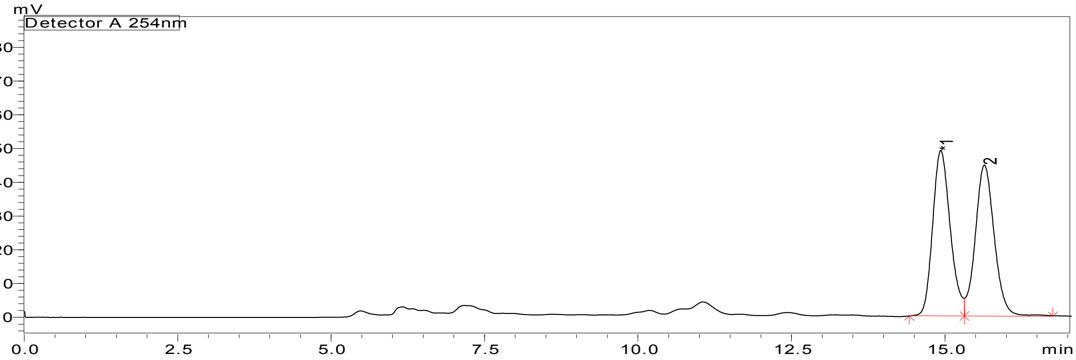

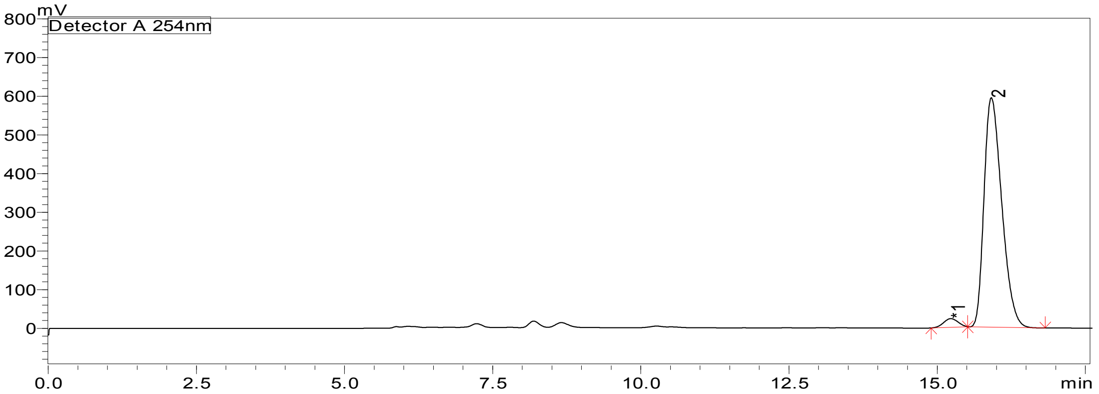

Peak#	Ret. Time	Area%
1	14.931	50.401
2	15.641	49.599

Peak#	Ret. Time	Area%
1	15.231	2.970
2	15.914	97.030

Supplementary Figure 1. HPLC chromatogram for compound **3a**

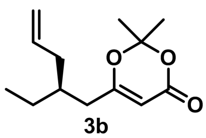

3b

(S)-6-(2-ethylpent-4-en-1-yl)-2,2-dimethyl-4H-1,3-dioxin-4-one (**3b**)
According to GPD, 16.5 mg, pale yellow oil, 74% yield, 90% ee.
^{1}H NMR (400 MHz, CDCl$_3$) δ 5.80-5.67 (m, 1H), 5.23 (s, 1H), 5.10-4.99 (m, 2H), 2.23-2.01 (m, 4H), 1.77-1.67 (m, 7H), 1.41-1.32 (m, 2H), 0.90 (t, J = 7.4 Hz, 3H) ppm.
^{13}C NMR (101 MHz, CDCl$_3$) δ 171.33, 161.26, 135.76, 117.08, 106.25, 94.34, 37.54, 37.14, 36.56, 25.67, 25.08, 25.06, 10.78 ppm.
HRMS (ESI) m/z [M+H]$^+$: calcd. 225.1485, found. 225.1484.
IR (film): ν_{max} (cm^{-1}) 3077, 2961, 1734, 1389, 1270, 1204, 1015, 801, 700.
Optical rotation: $[\alpha]_D^{25}$ = -11.46 (c = 0.25, CHCl$_3$).
HPLC: DAICEL CHIRALPAK OX-3, hexane/i-PrOH = 99/1, flow rate: 1 mL/min, λ = 254 nm, t_R(minor) = 46.5 min, t_R(major) = 48.0 min, ee = 90%.

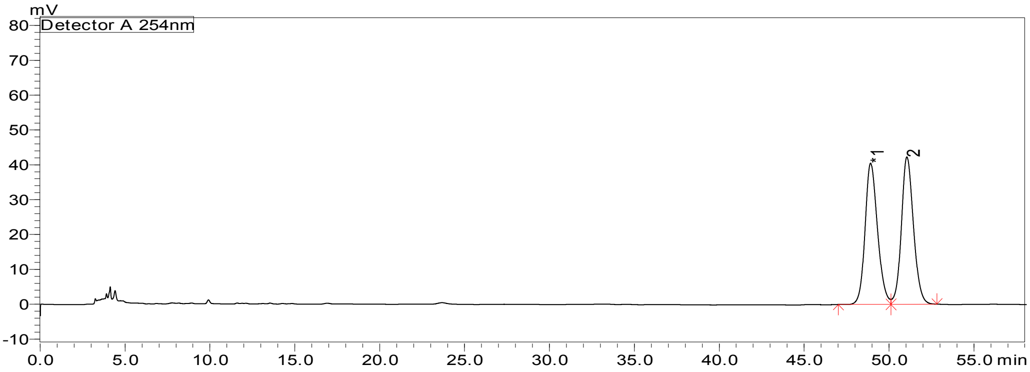

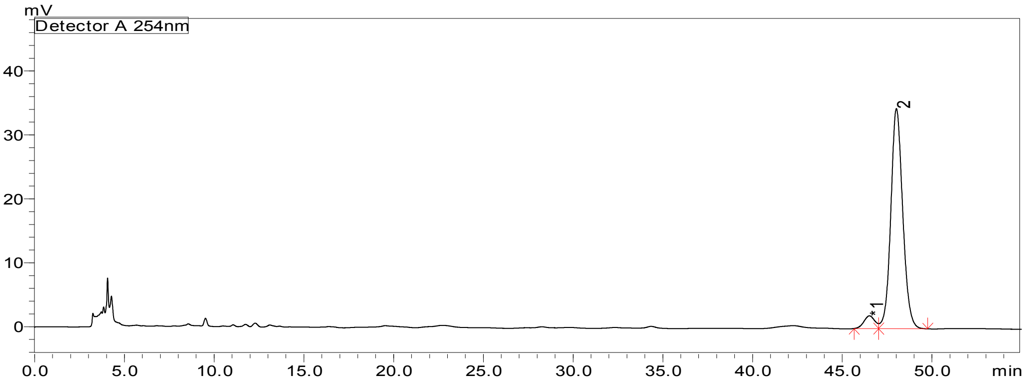

Peak#	Ret. Time	Area%
1	48.916	49.663
2	51.054	50.337

Peak#	Ret. Time	Area%
1	46.511	5.087
2	48.016	94.913

Supplementary Figure 2. HPLC chromatogram for compound **3b**

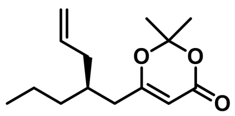

3c

(S)-2,2-dimethyl-6-(2-propylpent-4-en-1-yl)-4H-1,3-dioxin-4-one (**3c**)
According to GPD, 20 mg, pale yellow oil, 84% yield, 92% ee.
^{1}H NMR (400 MHz, CDCl$_3$) δ 5.81-5.67 (m, 1H), 5.23 (s, 1H), 5.13-4.97 (m, 2H), 2.26-1.99 (m, 4H), 1.86-1.76 (m, 1H), 1.69 (s, 6H), 1.39-1.22 (m, 4H), 0.90 (t, J = 6.7 Hz, 3H) ppm.
^{13}C NMR (101 MHz, CDCl$_3$) δ 171.32, 161.23, 135.74, 117.10, 106.23, 94.33, 37.92, 37.61, 36.40, 35.39, 34.84, 25.08, 19.62, 14.14 ppm.
HRMS (ESI) m/z [M+H]$^+$: calcd. 239.1642, found. 239.1642.
IR (film): v_{max} (cm^{-1}) 2958, 2927, 1734, 1389, 1251, 1204, 1014, 802.
Optical rotation: $[\alpha]_D^{25}$ = -8.79 (c = 1.00, CHCl$_3$).
HPLC: DAICEL CHIRALPAK OX-3, hexane/i-PrOH = 99/1, flow rate: 1 mL/min, λ = 254 nm, t_R(minor) = 39.9 min, t_R(major) = 42.1 min, ee = 92%.

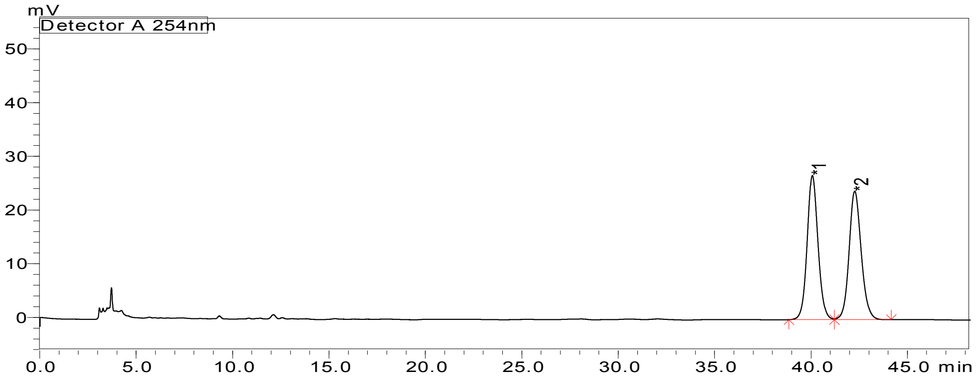

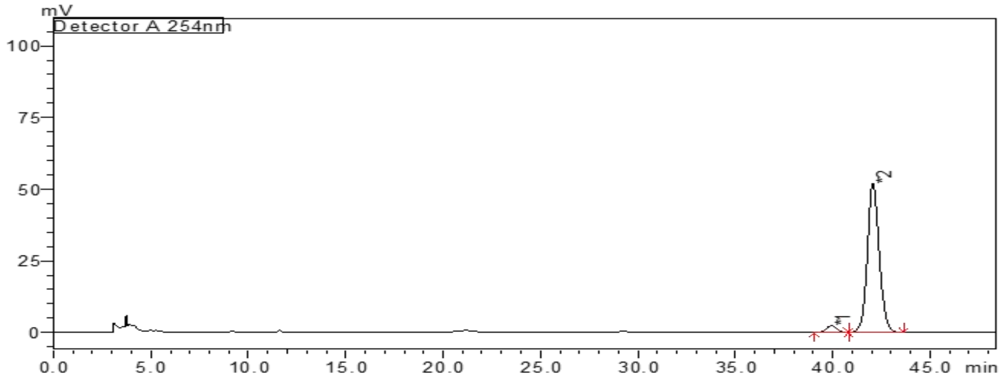

Peak#	Ret. Time	Area%
1	40.068	50.210
2	42.268	49.790

Peak#	Ret. Time	Area%
1	39.927	3.923
2	42.061	96.077

Supplementary Figure 3. HPLC chromatogram for compound **3c**

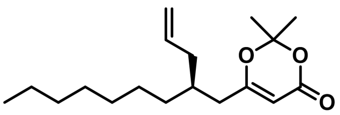

3d

(*S*)-6-(2-allylnonyl)-2,2-dimethyl-4*H*-1,3-dioxin-4-one (**3d**)
According to GPD, 23 mg, pale yellow oil, 71% yield, 93% ee.
^{1}H NMR (400 MHz, CDCl$_3$) δ 5.79-5.68 (m, 1H), 5.22 (s, 1H), 5.10-5.01 (m, 2H), 2.24-1.98 (m, 5H), 1.90-1.82 (m, 1H), 1.74-1.64 (m, 9H), 1.23-1.06 (m, 2H), 1.04-0.76 (m, 9H) ppm.
^{13}C NMR (101 MHz, CDCl$_3$) δ 171.36, 161.26, 135.76, 117.10, 106.23, 94.33, 37.92, 37.61, 35.10, 33.11, 31.79, 29.69, 29.21, 26.47, 25.08, 24.99, 22.62, 14.07 ppm.
HRMS (ESI) m/z [M+H]$^+$: calcd. 295.2268, found. 295.2268.
IR (film): ν_{max} (cm^{-1}) 2926, 2855, 1735, 1839, 1270,1204, 1014, 901,802.
Optical rotation: $[\alpha]_D^{25}$ = 0.18 (*c* = 1.00, CHCl$_3$).
HPLC: DAICEL CHIRALPAK OX-3, hexane/*i*-PrOH = 47/3, flow rate: 0.5 mL/min, λ = 254 nm, t_R(major) = 19.7 min, t_R(minor) = 19.0 min, ee = 93%.

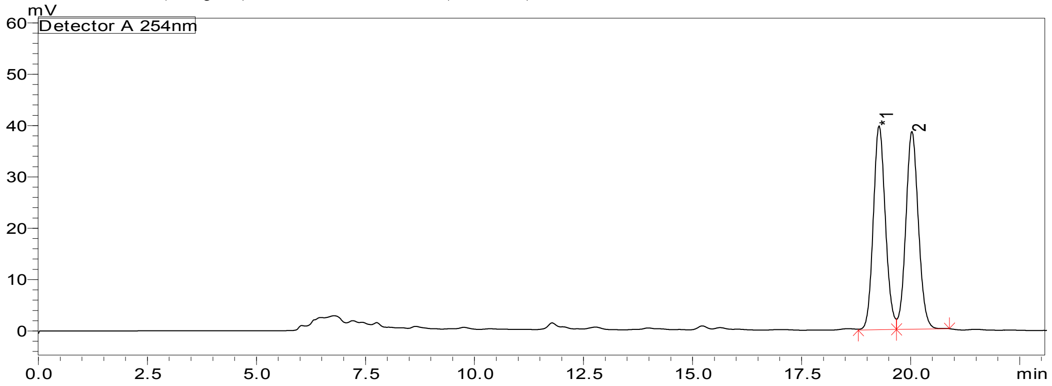

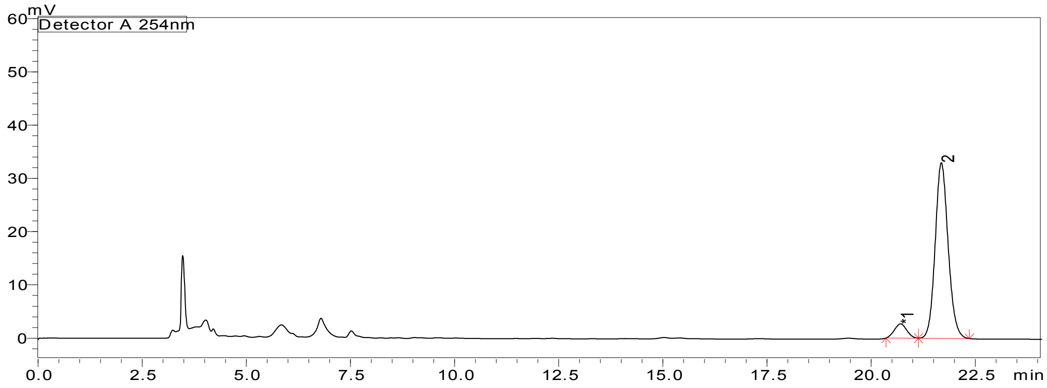

Peak#	Ret. Time	Area%
1	19.276	49.794
2	20.028	50.206

Peak#	Ret. Time	Area%
1	19.032	3.462
2	19.749	96.538

Supplementary Figure 4. HPLC chromatogram for compound **3d**

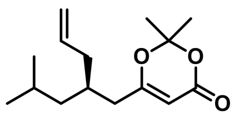

3e

(*S*)-6-(2-isobutylpent-4-en-1-yl)-2,2-dimethyl-4*H*-1,3-dioxin-4-one (**3e**)

According to GPD, 19 mg, pale yellow oil, 75% yield, 94% ee

^{1}H NMR (400 MHz, CDCl$_3$) δ 5.80-5.66 (m, 1H), 5.22 (s, 1H), 5.11-4.99 (m, 2H), 2.25-2.17 (m, 1H), 2.16-1.97 (m, 3H), 1.91-1.81 (m, 1H), 1.72-1.66 (m, 7H), 1.24-1.06 (m, 2H), 0.88 (dd, *J* = 6.5, 4.2 Hz, 6H) ppm.

^{13}C NMR (101 MHz, CDCl$_3$) δ 171.25, 161.22, 135.58, 117.25, 106.22, 94.37, 42.77, 38.15, 37.74, 32.71, 25.17, 25.05, 24.99, 22.93, 22.34 ppm.

HRMS (ESI) m/z [M+H]$^+$: calcd. 253.1798, found. 253.1797.

IR (film): ν_{max} (cm^{-1}) 2956, 2927, 1735, 1398, 1251, 1204, 901, 801.

Optical rotation: $[\alpha]_D^{25}$ = -2.47 (*c* = 1.00, CHCl$_3$).

HPLC: DAICEL CHIRALPAK OX-3, hexane/*i*-PrOH = 47/1, flow rate: 0.5 mL/min, λ = 254 nm, t_R(minor) = 23.1 min, t_R(major) = 24.2min, ee = 94%.

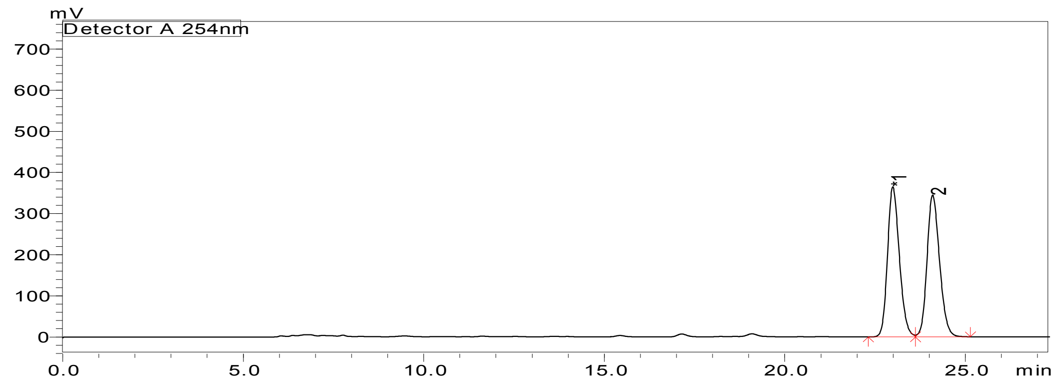

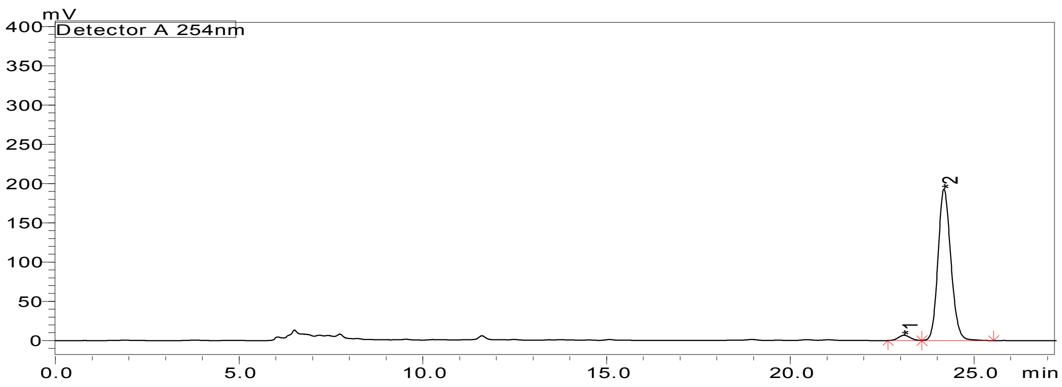

Peak#	Ret. Time	Area%
1	22.991	49.999
2	24.095	50.001

Peak#	Ret. Time	Area%
1	23.094	3.130
2	24.180	96.870

Supplementary Figure 5. HPLC chromatogram for compound **3e**

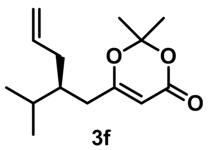

3f

(*S*)-6-(2-isopropylpent-4-en-1-yl)-2,2-dimethyl-4*H*-1,3-dioxin-4-one (**3f**)

According to GPD, 13 mg, pale yellow oil, 55% yield, 86% ee.

^{1}H NMR (400 MHz, $CDCl_3$) 5.79-5.67 (m, 1H), 5.24 (s, 1H), 5.08-5.00 (m, 2H), 2.23-2.08 (m, 3H), 2.01-1.91 (m, 1H), 1.82-1.73 (m, 1H), 1.71-1.63 (m, 7H), 0.89 (d, J = 6.8 Hz, 6H) ppm.

^{13}C NMR (101 MHz, $CDCl_3$) δ 171.83, 161.27, 136.64, 116.77, 106.22, 94.28, 40.83, 34.87, 34.57, 28.91, 25.09, 24.99, 18.91, 18.71 ppm.

HRMS (ESI) m/z [M+H]$^+$: calcd. 239.1642, found. 239.1643.

IR (film): ν_{max} (cm^{-1}) 2960, 2927, 1735, 1398, 1252, 1026, 1015, 901.

Optical rotation: $[\alpha]_D^{25}$ = -25.35 (c = 0.50, $CHCl_3$).

HPLC: DAICEL CHIRALPAK OX-3, hexane/*i*-PrOH = 97/3, flow rate: 1 mL/min, λ = 254 nm, t_R(major) = 21.7 min, t_R(minor) = 20.7 min, ee = 86%.

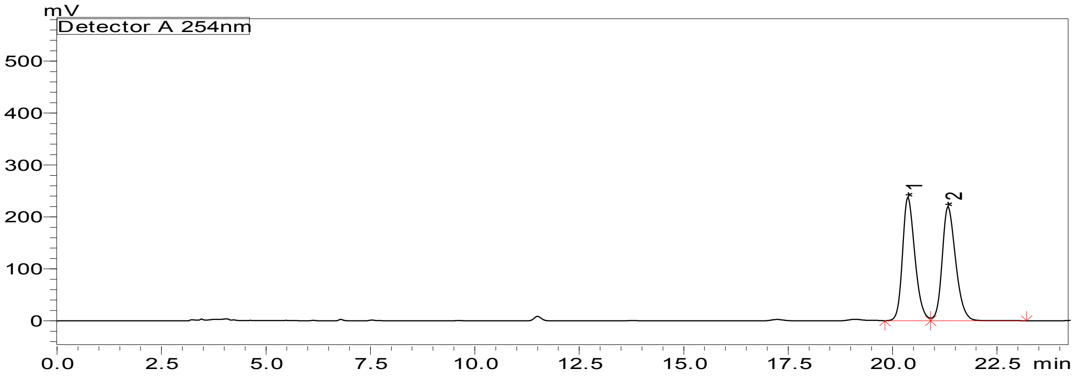

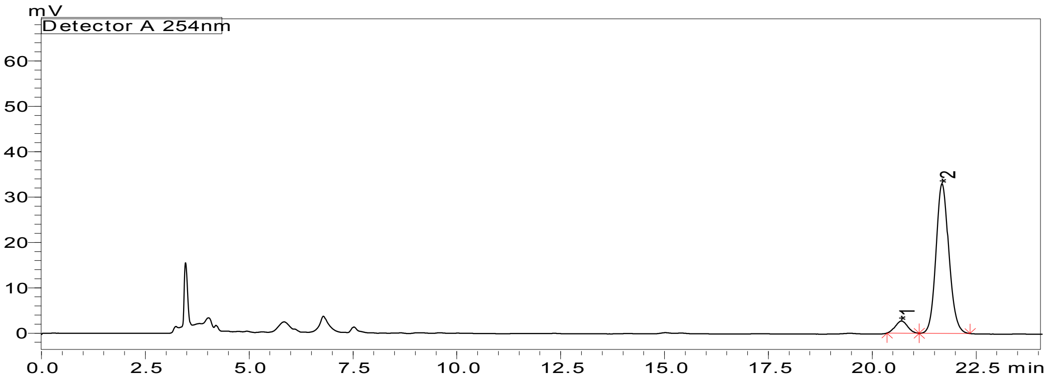

Peak#	Ret. Time	Area%
1	20.364	49.727
2	21.322	50.273

Peak#	Ret. Time	Area%
1	20.704	7.093
2	21.683	92.907

Supplementary Figure 6. HPLC chromatogram for compound **3f**

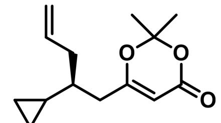

3g

(*S*)-6-(2-cyclopropylpent-4-en-1-yl)-2,2-dimethyl-4*H*-1,3-dioxin-4-one (**3g**)

According to GPD, 10.5 mg, pale yellow oil, 54% yield, 89% ee.

^{1}H NMR (400 MHz, CDCl$_3$) 5.92-5.78 (m, 1H), 5.25 (s, 1H), 5.12-5.00 (m, 2H), 2.35-2.25 (m, 2H), 2.24-2.14 (m, 2H), 1.68 (d, J = 3.1 Hz, 6H), 1.09-0.99 (m, 1H), 0.66-0.55 (m, 1H), 0.54-0.43 (m, 2H), 0.19-0.09 (m, 2H) ppm.

^{13}C NMR (101 MHz, CDCl$_3$) δ 171.09, 161.33, 135.84, 116.91, 106.22, 94.34, 41.47, 38.84, 25.53, 24.72, 15.79, 4.35, 4.32 ppm.

HRMS (ESI) m/z [M+H]$^+$: calcd. 237.1485, found. 237.1486.

IR (film): ν_{max} (cm^{-1}) 3077, 2999,,2920,1735,1374,1204,1015,900,803

Optical rotation: $[\alpha]_D^{25}$ = 1.54 (c = 0.50, CHCl$_3$).

HPLC: DAICEL CHIRALPAK OX-3, hexane/*i*-PrOH = 99/1, flow rate: 1 mL/min, λ = 254 nm, t_R(major) = 53.6 min, t_R(minor) = 52.0 min, ee = 89%.

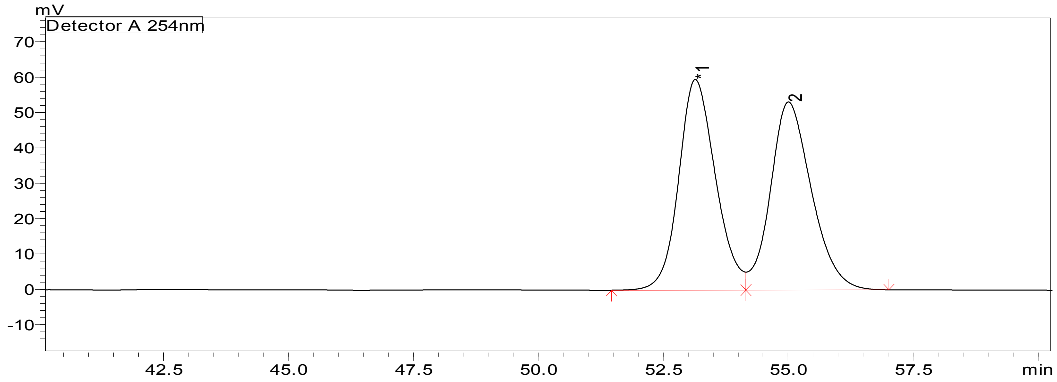

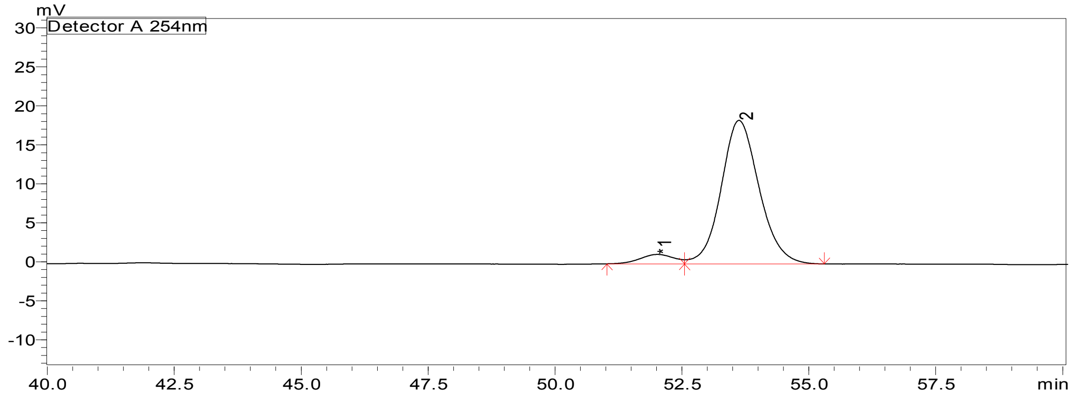

Peak#	Ret. Time	Area%
1	53.143	50.187
2	55.006	49.813

Peak#	Ret. Time	Area%
1	52.013	5.573
2	53.624	94.427

Supplementary Figure 7. HPLC chromatogram for compound **3g**

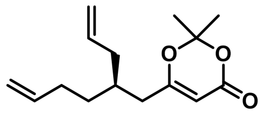

3h

(S)-6-(2-allylhex-5-en-1-yl)-2,2-dimethyl-4H-1,3-dioxin-4-one (**3h**)
According to GPD, 16 mg, colourless oil, 64% yield, 89% ee.
^{1}H NMR (400 MHz, $CDCl_3$) δ 5.84-5.67 (m, 2H), 5.23 (s, 1H), 5.11-4.92 (m, 4H), 2.26-2.00 (m, 6H), 1.88-1.77 (m, 1H), 1.69 (s, 6H), 1.45-1.36 (m, 2H) ppm.
^{13}C NMR (101 MHz, $CDCl_3$) δ 171.06, 161.19, 138.08, 135.46, 117.35, 114.91, 106.28, 94.43, 37.77, 37.43, 34.49, 32.28, 30.73, 25.10, 25.08 ppm.
HRMS (ESI) m/z $[M+H]^+$: calcd. 251.1642, found. 251.1642.
IR (film): ν_{max} (cm^{-1}) 2998, 2977, 1733, 1390, 1251, 1204, 1014, 902, 803.
Optical rotation: $[\alpha]_D^{25}$ = -6.71 (c = 1.00, $CHCl_3$).
HPLC: DAICEL CHIRALPAK OX-3, hexane/i-PrOH = 98/2, flow rate: 1 mL/min, λ = 254 nm, t_R(minor) = 27.3 min, t_R(major) = 28.2 min, ee = 89%.

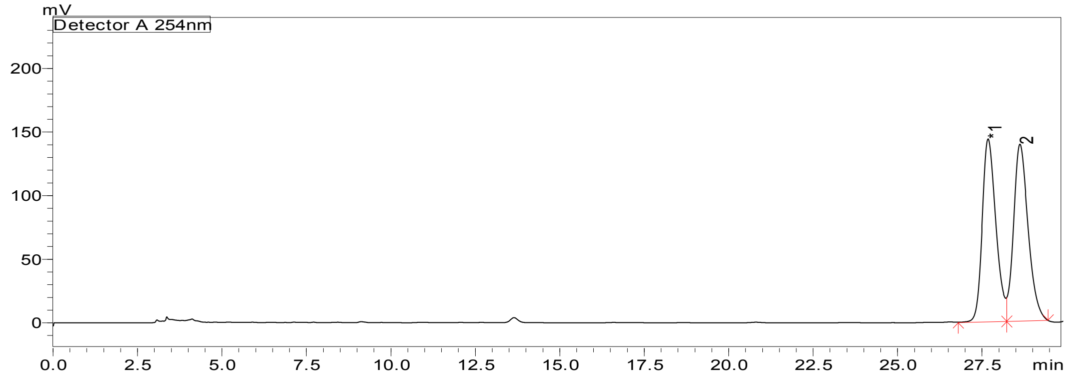

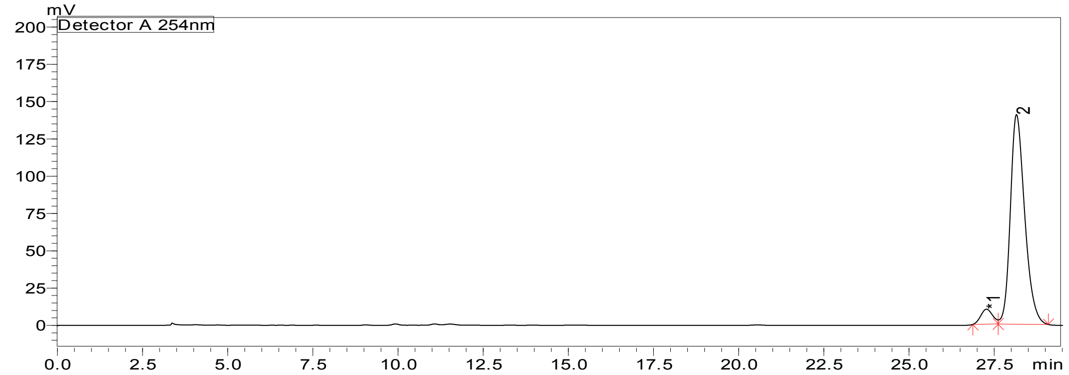

Peak#	Ret. Time	Area%
1	27.680	49.353
2	28.619	50.647

Peak#	Ret. Time	Area%
1	27.275	5.572
2	28.155	94.428

Supplementary Figure 8. HPLC chromatogram for compound **3h**

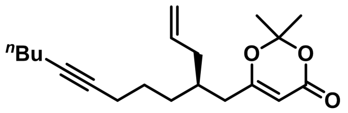

3i

(*S*)-6-(2-allylundec-6-yn-1-yl)-2,2-dimethyl-4*H*-1,3-dioxin-4-one (**3i**)
According to GPD, 22 mg, colourless oil, 69% yield, 88% ee.
^{1}H NMR (400 MHz, CDCl$_3$) 5.80-5.66 (m, 1H), 5.24 (s, 1H), 5.12-4.99 (m, 2H), 2.25-2.05 (m, 8H), 1.87-1.77 (m, 1H), 1.69 (s, 6H), 1.50-1.37 (m, 8H), 0.91 (t, J = 7.1 Hz, 3H) ppm.
^{13}C NMR (101 MHz, CDCl$_3$) δ 171.04, 161.18, 135.52, 117.31, 106.27, 94.46, 80.70, 79.51, 37.88, 37.55, 34.77, 32.28, 31.18, 26.11, 25.12, 25.07, 21.93, 18.89, 18.38, 13.62 ppm.
HRMS (ESI) m/z [M+H]$^+$: calcd. 319.2268, found. 319.2270.
IR (film): ν_{max} (cm^{-1}) 2997, 2932, 1733, 1390, 1252, 1204, 1014, 901, 803.
Optical rotation: $[\alpha]_D^{25}$ = -5.08 (c = 1.00, CHCl$_3$).
HPLC: DAICEL CHIRALPAK IC-3, hexane/*i*-PrOH = 64/1, flow rate: 1.3 mL/min, λ = 254 nm, t_R(major) = 63.1 min, t_R(minor) = 65.3 min, ee = 88%.

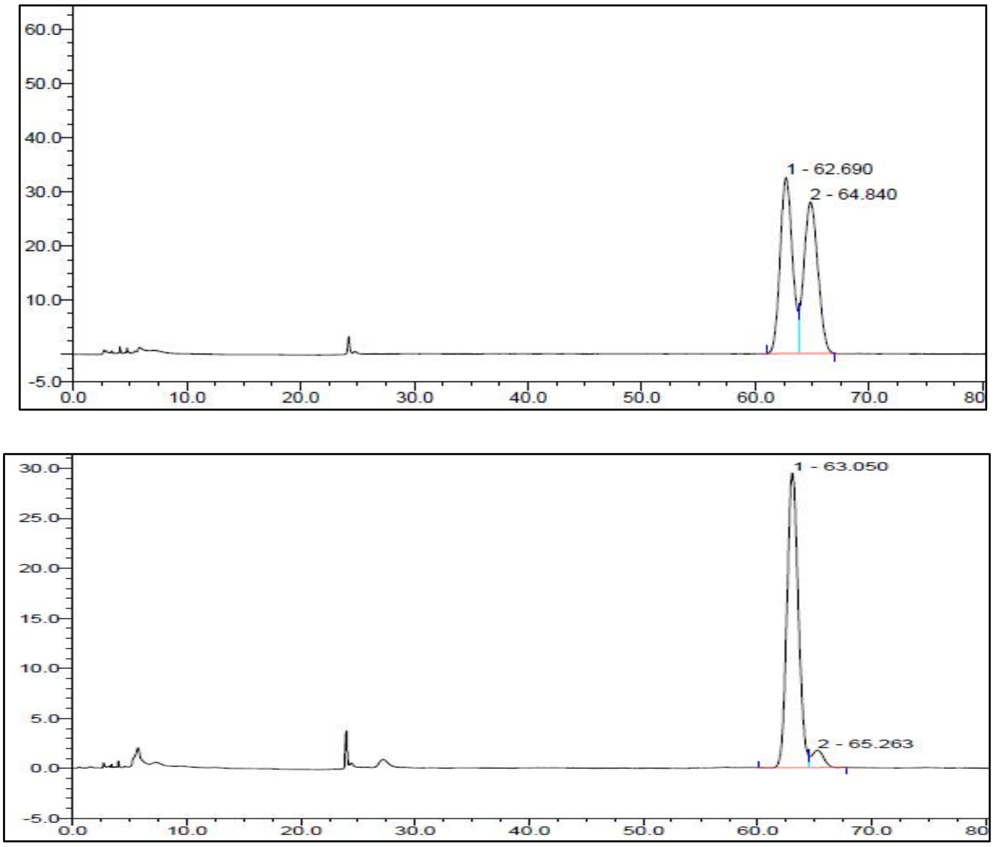

Peak#	Ret. Time	Area%
1	62.69	50.19
2	64.84	49.81

Peak#	Ret. Time	Area%
1	63.05	94.03
2	65.26	5.97

Supplementary Figure 9. HPLC chromatogram for compound **3i**

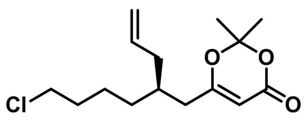

3j

(*S*)-6-(2-allyl-6-chlorohexyl)-2,2-dimethyl-4*H*-1,3-dioxin-4-one (**3j**)
According to GPD, 22 mg, pale yellow oil, 77% yield, 94% ee.
^{1}H NMR (400 MHz, $CDCl_3$) 5.81-5.65 (m, 1H), 5.24 (s, 1H), 5.13-5.01 (m, 2H), 3.54 (t, J = 6.5 Hz, 2H), 2.26-2.02 (m, 4H), 1.87-1.73 (m, 3H), 1.69 (s, 6H), 1.53-1.41 (m, 2H), 1.38-1.28 (m, 2H) ppm.
^{13}C NMR (101 MHz, $CDCl_3$) δ 170.93, 161.09, 135.40, 117.37, 106.28, 94.45, 44.79, 37.83, 37.48, 34.97, 32.52, 32.28, 25.11, 25.03, 23.71 ppm.
HRMS (ESI) m/z [M+H]$^{+}$: calcd. 287.1408, found. 287.1410.
IR (film): ν_{max} (cm^{-1}) 2998, 2938, 1731, 1390, 1252, 1204, 1015, 902, 803.
Optical rotation: $[\alpha]_D^{25}$ = -4.13 (c = 1.00, $CHCl_3$).
HPLC: DAICEL CHIRALPAK OX-3, hexane/*i*-PrOH = 99/1, flow rate: 1 mL/min, λ = 254 nm, t_R(major) = 58.7 min, t_R(minor) = 60.8 min, ee = 94%.

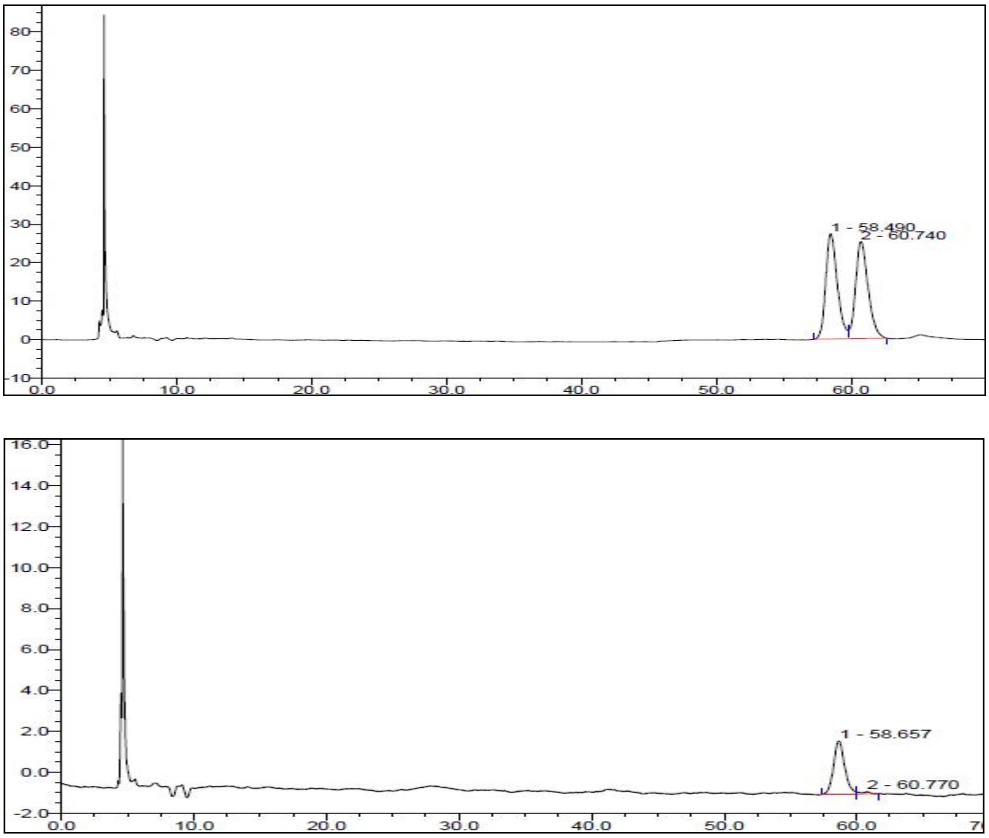

Peak#	Ret. Time	Area%
1	58.49	49.99
2	60.74	50.01

Peak#	Ret. Time	Area%
1	58.66	96.98
2	60.77	3.02

Supplementary Figure 10. HPLC chromatogram for compound **3j**

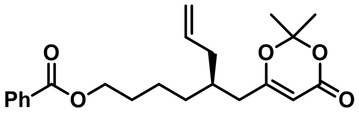

3k

(*S*)-5-((2,2-dimethyl-4-oxo-4*H*-1,3-dioxin-6-yl)methyl)oct-7-en-1-yl benzoate (**3k**)
According to GPD, 33 mg, colourless oil, 89% yield, 92% ee.
^{1}H NMR (400 MHz, $CDCl_3$) δ 8.08-8.00 (m, 2H), 7.57 (t, *J* = 7.4 Hz, 1H), 7.45 (t, *J* = 7.7 Hz, 2H), 5.80-5.66 (m, 1H), 5.23 (s, 1H), 5.11-4.99 (m, 2H), 4.32 (t, *J* = 6.5 Hz, 2H), 2.27-2.03 (m, 4H), 1.86-1.73 (m, 3H), 1.68 (s, 6H), 1.54-1.33 (m, 4H) ppm.
^{13}C NMR (151 MHz, $CDCl_3$) δ 170.99, 166.58, 161.11, 135.43, 132.88, 130.33, 129.47, 128.34, 117.36, 106.28, 94.45, 64.69, 37.86, 37.48, 34.99, 32.80, 28.86, 25.10, 25.03, 23.06 ppm.
HRMS (ESI) m/z [M+H]$^+$: calcd. 373.2010, found. 373.2009.
IR (film): ν_{max} (cm^{-1}) 3073, 2997, 2938, 1721, 1602,1451, 1390, 1270, 1203, 1014, 713, 688.
Optical rotation: $[\alpha]_D^{25}$ = -2.18 (*c* = 1.00, $CHCl_3$).
HPLC: DAICEL CHIRALPAK IBN-3, hexane/*i*-PrOH = 9/1, flow rate: 0.5 mL/min, λ = 254 nm, t_R(minor) = 25.2 min, t_R(major) = 24.0 min, ee = 92%.

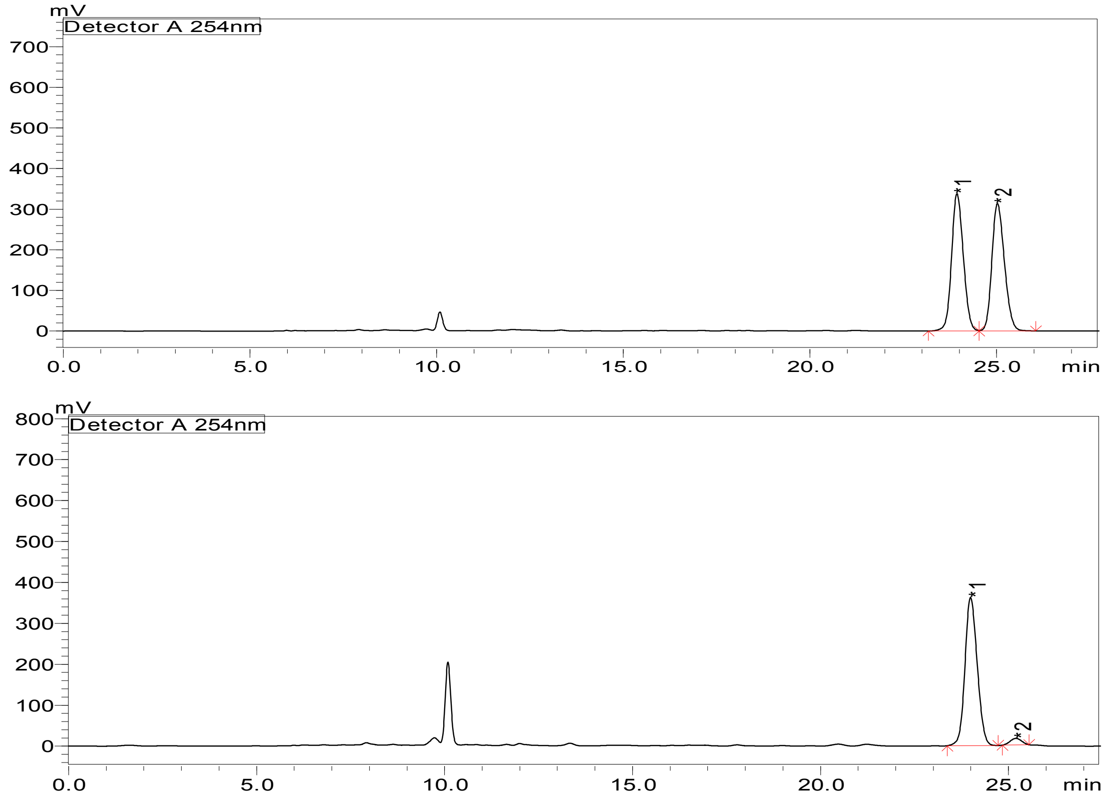

Peak#	Ret. Time	Area%
1	23.936	50.258
2	25.019	49.742

Peak#	Ret. Time	Area%
1	23.991	96.026
2	25.197	3.974

Supplementary Figure 11. HPLC chromatogram for compound **3k**

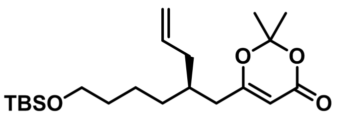

3l

(*S*)-6-(2-allyl-6-((tert-butyldimethylsilyl)oxy)hexyl)-2,2-dimethyl-4*H*-1,3-dioxin-4-one (**3l**)

According to GPD, 22 mg, colourless oil, 58% yield, 91% ee.

^{1}H NMR (400 MHz, $CDCl_3$) δ 5.80-5.67 (m, 1H), 5.23 (s, 1H), 5.12-4.97 (m, 2H), 3.60 (t, J = 6.2 Hz, 2H), 2.23-2.02 (m, 4H), 1.84-1.77 (m, 1H), 1.69 (s, 6H), 1.54-1.46 (m, 2H), 1.42-1.28 (m, 4H), 0.89 (s, 9H), 0.05 (s, 6H) ppm

^{13}C NMR (151 MHz, $CDCl_3$) δ 171.22, 161.20, 135.63, 117.19, 106.23, 94.37, 62.90, 37.88, 37.51, 35.05, 32.89, 25.92, 25.08, 25.07, 22.74, 18.31, -5.30 ppm.

HRMS (ESI) m/z $[M+H]^+$: calcd. 383.2612, found. 383.2612.

IR (film): ν_{max} (cm^{-1}) 2929, 2857, 1735, 1633, 1389, 1253, 1100, 1015, 836.

Optical rotation: $[\alpha]_D^{25}$ = -1.38 (c = 1.00, $CHCl_3$).

HPLC: DAICEL CHIRALPAK OX-3, hexane/*i*-PrOH = 99/1, flow rate: 1.0 mL/min, λ = 254 nm, t_R(minor) = 24.0 min, t_R(major) = 25.2 min, ee = 91%.

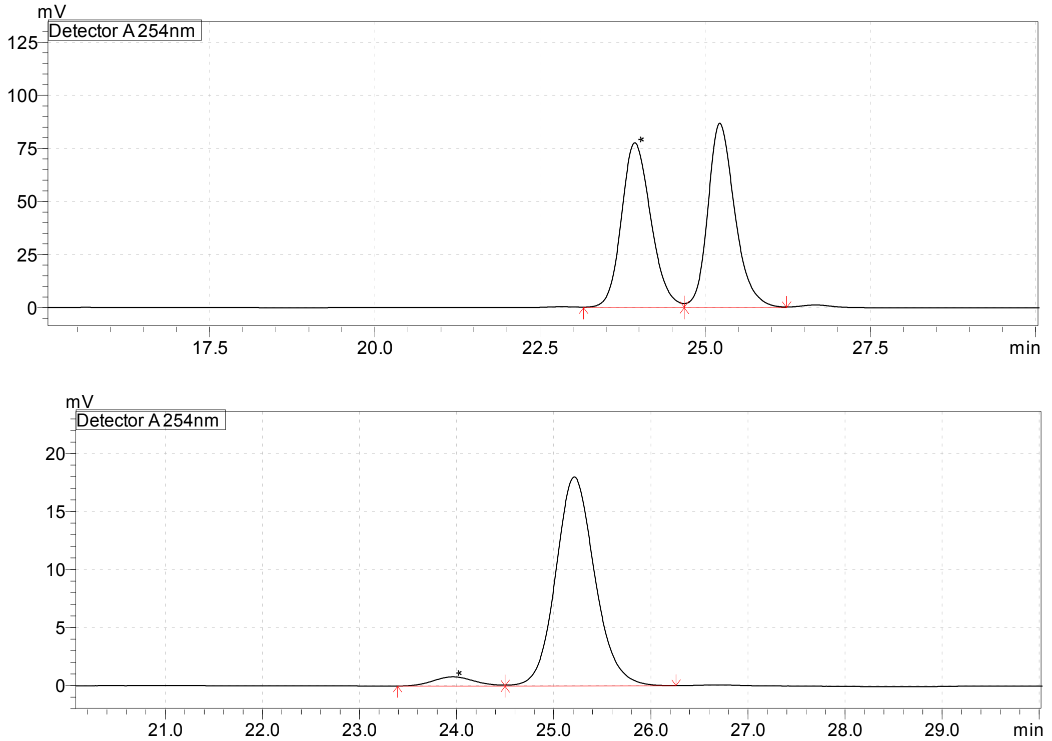

Peak#	Ret. Time	Area%
1	23.935	49.360
2	25.221	50.640

Peak#	Ret. Time	Area%
1	23.961	4.469
2	25.212	95.531

Supplementary Figure 12. HPLC chromatogram for compound **3l**

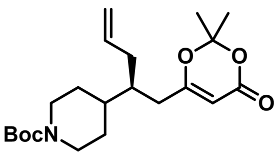

3n

(*S*)-tert-butyl-4-(1-(2,2-dimethyl-4-oxo-4*H*-1,3-dioxin-6-yl)pent-4-en-2-yl)piperidine-1-carboxylate (**3n**)

According to GPD, 28 mg, colourless oil, 74% yield, 89% ee.

^{1}H NMR (400 MHz, $CDCl_3$) δ 5.79-5.61 (m, 1H), 5.24 (s, 1H), 5.10-5.01 (m, 2H), 4.15 (s, 2H), 2.62 (s, 2H), 2.28-2.20 (m, 1H), 2.19-2.10 (m, 2H), 2.07-1.97 (m, 1H), 1.78-1.70 (m, 2H), 1.68 (s, 6H), 1.59-1.52 (m, 2H), 1.46 (s, 9H), 1.28-1.18 (m, 2H) ppm.

^{13}C NMR (151 MHz, $CDCl_3$) δ 171.16, 161.05, 154.68, 135.90, 117.33, 106.32, 94.48, 79.39, 39.66, 38.12, 34.92, 34.68, 28.41, 25.15, 24.98 ppm.

HRMS (ESI) m/z [M+Na]$^+$: calcd. 402.2251, found. 402.2249.

IR (film): ν_{max} (cm^{-1}) 2959, 2924, 1729, 1630, 1261, 1014, 802.

Optical rotation: $[\alpha]_D^{25}$ = -2.20 (*c* = 1.00, $CHCl_3$).

HPLC: DAICEL CHIRALPAK IBN-3, hexane/*i*-PrOH = 7/3, flow rate: 0.5 mL/min, λ = 254 nm, t_R(minor) = 11.1 min, t_R(major) = 12.7 min, ee = 89%.

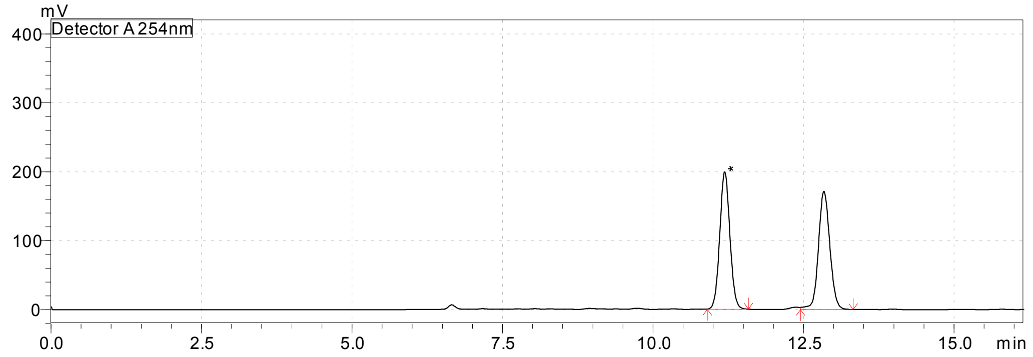

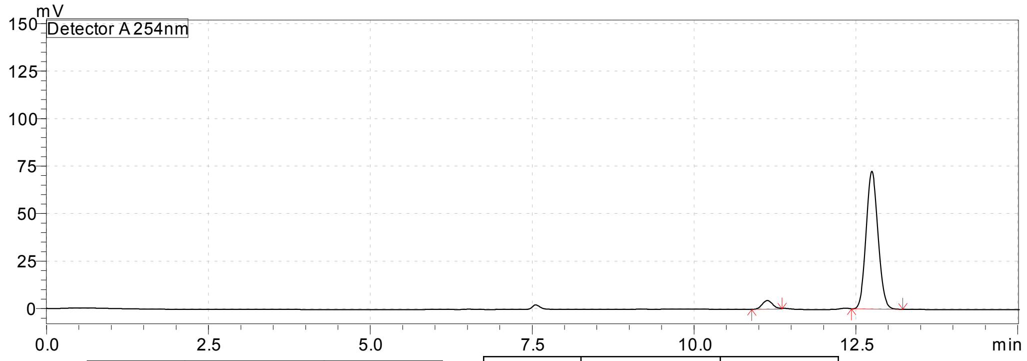

Peak#	Ret. Time	Area%
1	11.191	50.713
2	12.839	49.287

Peak#	Ret. Time	Area%
1	11.132	5.414
2	12.746	94.586

Supplementary Figure 13. HPLC chromatogram for compound **3n**

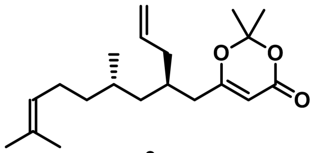

3o

6-((2*R*,4*S*)-2-allyl-4,8-dimethylnon-7-en-1-yl)-2,2-dimethyl-4*H*-1,3-dioxin-4-one (**3o**)
According to GPD, 20 mg, yellow brown oil, 72% yield, 15/1 dr.
^{1}H NMR (400 MHz, $CDCl_3$) δ 5.79-5.66 (m, 1H), 5.22 (s, 1H), 5.11-4.98 (m, 3H), 2.14 (d, J = 6.8 Hz, 2H), 2.06 (t, J = 6.6 Hz, 2H), 2.02-1.83 (m, 3H), 1.69 (s, 8H), 1.60 (s, 3H), 1.55-1.48 (m, 1H), 1.38-1.04 (m, 5H), 0.87 (d, J = 6.6 Hz, 3H) ppm.
^{13}C NMR (101 MHz, $CDCl_3$) δ 171.28, 161.22, 135.68, 131.36, 124.57, 117.21, 106.26, 94.42, 41.19, 38.22, 37.95, 36.96, 32.63, 29.50, 25.69, 25.38, 25.31, 24.93, 19.77, 17.67 ppm.
HRMS (ESI) m/z $[M+H]^+$: calcd. 321.2424, found. 321.2424.
IR (film): ν_{max} (cm^{-1}) 2923, 2854, 1733, 1633, 1456,1389,1204, 764, 750.
Optical rotation: $[\alpha]_D^{25}$ = -11.39 (c = 1.00, $CHCl_3$).
HPLC: DAICEL CHIRALPAK OX-3, hexane/*i*-PrOH = 47/3, flow rate: 0.5 mL/min, λ = 254 nm, t_R(minor) = 20.4 min, t_R(major) = 21.2 min, de = 91%.

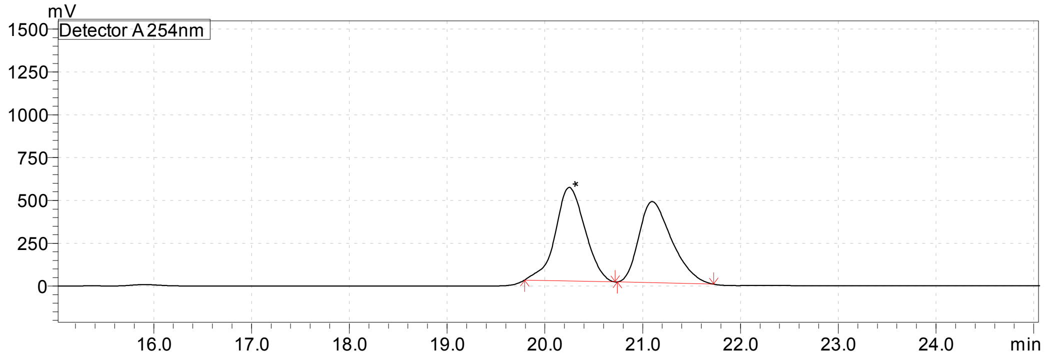

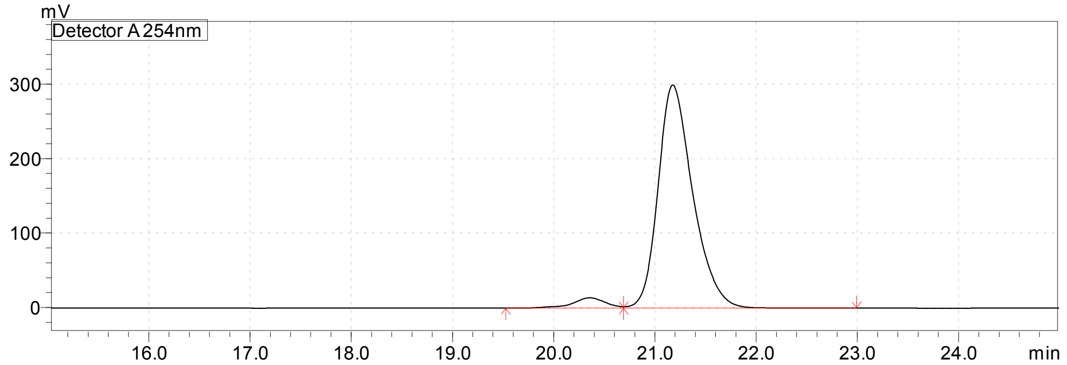

Peak#	Ret. Time	Area%
1	20.249	50.959
2	21.097	49.041

Peak#	Ret. Time	Area%
1	20.356	4.304
2	21.176	95.696

Supplementary Figure 14. HPLC chromatogram for compound **3o**

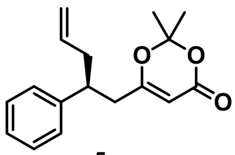

5a

(S)-2,2-dimethyl-6-(2-phenylpent-4-en-1-yl)-4H-1,3-dioxin-4-one (**5a**)
According to GPE, 23 mg, pale yellow oil, 84% yield, 94% ee.
^{1}H NMR (400 MHz, $CDCl_3$) δ 7.33-7.27 (m, 2H), 7.23-7.18 (m, 1H), 7.13 (d, J = 7.1 Hz, 2H), 5.70-5.59 (m, 1H), 5.08 (s, 1H), 5.05-4.96 (m, 2H), 3.05-2.95 (m, 1H), 2.70-2.61 (m, 1H), 2.52-2.35 (m, 3H), 1.56 (s, 3H), 1.44 (s, 3H) ppm.
^{13}C NMR (101 MHz, $CDCl_3$) δ 169.99, 161.06, 142.44, 135.48, 128.46, 127.41, 126.81, 117.12, 106.32, 94.61, 42.60, 41.15, 39.31, 25.22, 24.51 ppm.
HRMS (ESI) m/z $[M+H]^+$: calcd. 273.1485, found. 273.1485.
IR (film): ν_{max} (cm^{-1}) 3064, 3029, 2999, 1729, 1632, 1390, 125, 1204, 1014, 761, 643.
Optical rotation: $[\alpha]_D^{25}$ = 22.04 (c = 0.20, $CHCl_3$).
HPLC: DAICEL CHIRALPAK IF-3, hexane/i-PrOH = 24/1, flow rate: 0.5 mL/min, λ = 254 nm, t_R(minor) = 31.7 min, t_R(major) = 30.0 min, ee = 94%.

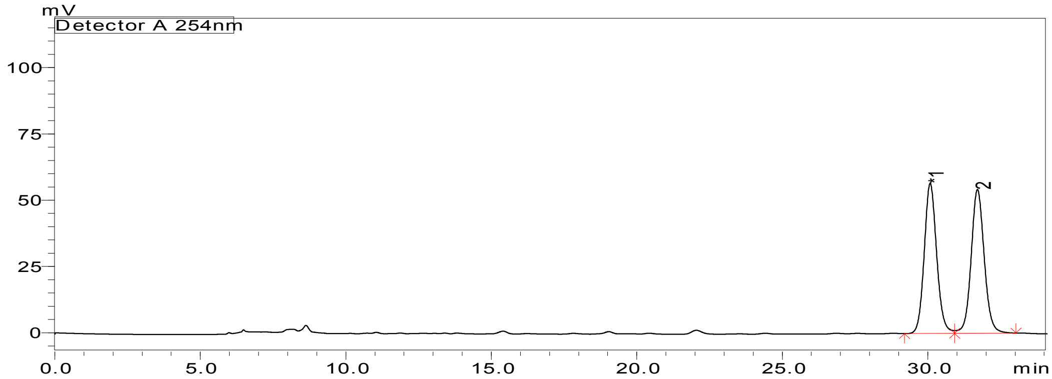

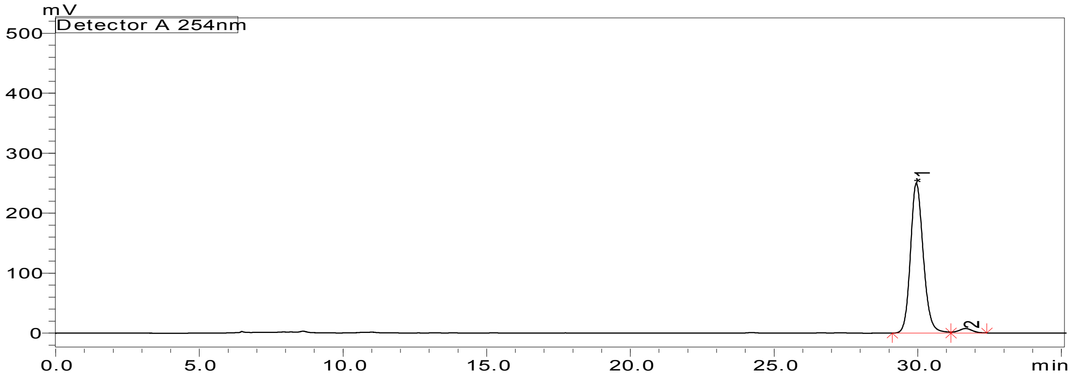

Peak#	Ret. Time	Area%
1	30.080	49.823
2	31.705	50.177

Peak#	Ret. Time	Area%
1	29.952	96.895
2	31.666	3.105

Supplementary Figure 15. HPLC chromatogram for compound **5a**

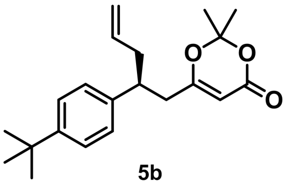

5b

(S)-6-(2-(4-(tert-butyl)phenyl)pent-4-en-1-yl)-2,2-dimethyl-4H-1,3-dioxin-4-one (**5b**)
According to GPE, 22 mg, colourless oil, 67% yield, 89% ee.
^{1}H NMR (400 MHz, CDCl$_3$) δ 7.29 (d, J = 8.3 Hz, 2H), 7.06 (d, J = 8.3 Hz, 2H), 5.72-5.59 (m, 1H), 5.10 (s, 1H), 5.08-4.93 (m, 2H), 3.04-2.90 (m, 1H), 2.71-2.59 (m, 1H), 2.52-2.29 (m, 3H), 1.54 (s, 3H), 1.42 (s, 3H), 1.29 (s, 9H) ppm.
^{13}C NMR (101 MHz, CDCl$_3$) δ 161.25, 149.59, 139.35, 135.72, 127.02, 125.28, 117.02, 106.32, 94.58, 42.03, 41.19, 39.22, 34.36, 31.30, 25.19, 24.41 ppm.
HRMS (ESI) m/z [M+H]$^+$: calcd. 329.2111, found. 329.2111.
IR (film): ν_{max} (cm^{-1}) 3055, 2998, 1728, 1632, 1390, 1251, 1203, 1014, 858, 749.
Optical rotation: $[\alpha]_D^{25}$ = 30.74 (c = 1.00, CHCl$_3$).
HPLC: DAICEL CHIRALPAK OX-3, hexane/i-PrOH = 9/1, flow rate: 0.5 mL/min, λ = 254 nm, t_R(minor) = 17.8 min, t_R(major) = 20.1 min, ee = 89%.

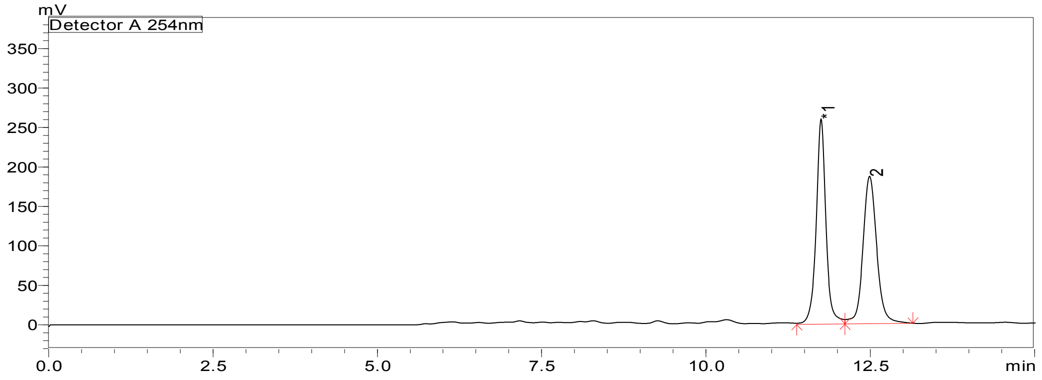

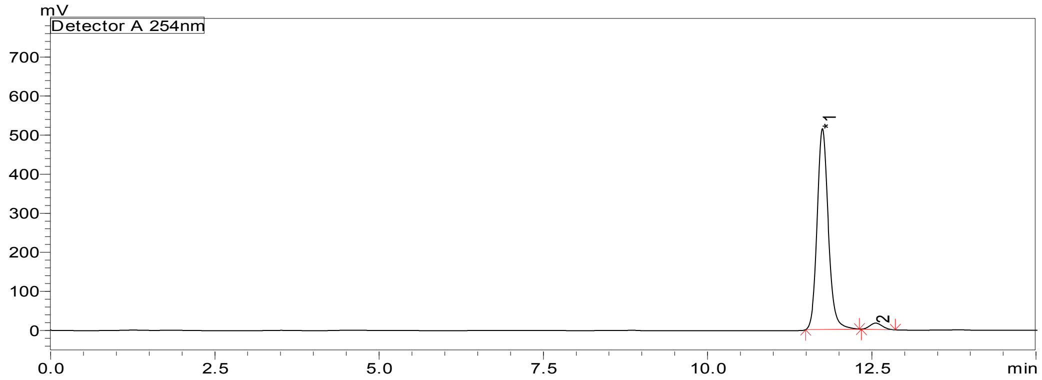

Peak#	Ret. Time	Area%
1	22.298	51.050
2	24.558	48.950

Peak#	Ret. Time	Area%
1	17.820	5.273
2	20.052	94.727

Supplementary Figure 16. HPLC chromatogram for compound **5b**

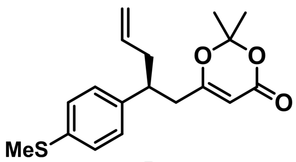

5c

(*S*)-2,2-dimethyl-6-(2-(4-(methylthio)phenyl)pent-4-en-1-yl)-4*H*-1,3-dioxin-4-one (**5c**)

According to GPE, 26 mg, pale yellow oil, 80% yield, 93% ee.

^{1}H NMR (400 MHz, $CDCl_3$) δ 7.19 (d, J = 8.3 Hz, 2H), 7.06 (d, J = 8.3 Hz, 2H), 5.71-5.53 (m, 1H), 5.08 (s, 1H), 5.05-4.95 (m, 2H), 3.03-2.90 (m, 1H), 2.68-2.58 (m, 1H), 2.46 (s, 4H), 2.40-2.32 (m, 2H), 1.57 (s, 3H), 1.46 (s, 3H) ppm.

^{13}C NMR (101 MHz, $CDCl_3$) δ 169.84, 161.01, 139.34, 136.65, 135.34, 127.90, 126.74, 117.25, 106.34, 94.64, 42.06, 41.07, 39.30, 25.24, 24.60, 15.87 ppm.

HRMS (ESI) m/z $[M+H]^+$: calcd. 319.1362, found. 319.1364.

IR (film): ν_{max} (cm^{-1}) 3075, 2996, 2921, 1724, 1630, 1460, 1389, 1270, 1202, 1013, 814.

Optical rotation: $[\alpha]_D^{25}$ = 51.01 (c = 1.00, $CHCl_3$).

HPLC: DAICEL CHIRALPAK IF-3, hexane/*i*-PrOH = 19/1, flow rate: 1 mL/min, λ = 254 nm, t_R(minor) = 17.7 min, t_R(major) = 16.8 min, ee = 93%.

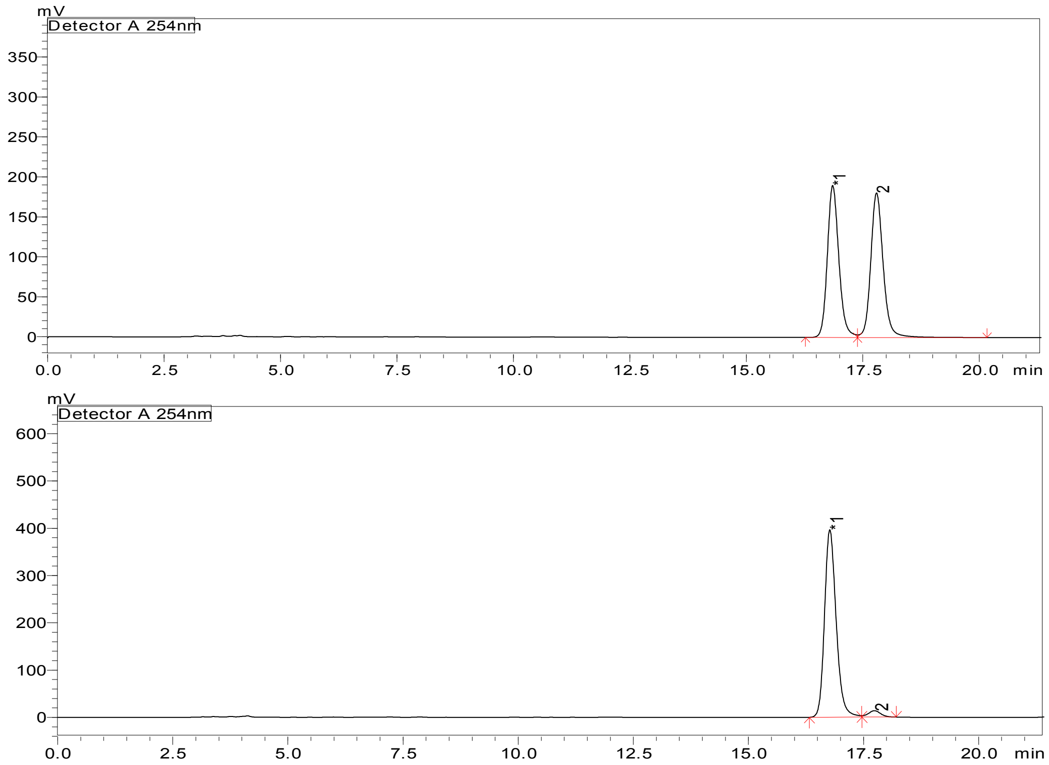

Peak#	Ret. Time	Area%
1	16.852	49.344
2	17.793	50.656

Peak#	Ret. Time	Area%
1	16.766	96.514
2	17.736	3.486

Supplementary Figure 17. HPLC chromatogram for compound **5c**

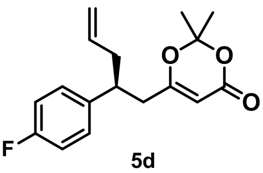

5d

(S)-6-(2-(4-fluorophenyl)pent-4-en-1-yl)-2,2-dimethyl-4H-1,3-dioxin-4-one (**5d**)
According to GPE, 179.8 mg, pale yellow oil, 62% yield, 91% ee.
^{1}H NMR (400 MHz, CDCl$_3$) δ 7.14-7.05 (m, 2H), 7.04-6.96 (m, 2H), 5.69-5.55 (m, 1H), 5.08 (s, 1H), 5.05-4.94 (m, 2H), 3.06-2.95 (m, 1H), 2.69-2.60 (m, 1H), 2.49-2.40 (m, 1H), 2.39-2.31 (m, 2H), 1.57 (s, 3H), 1.46 (s, 3H) ppm.
^{13}C NMR (151 MHz, CDCl$_3$) δ 169.71, 161.63 (d, J = 245.6 Hz), 160.97, 138.15 (d, J = 3.6 Hz), 135.21, 128.85 (d, J = 7.6 Hz), 117.42, 115.36 (d, J = 21.5 Hz), 106.39, 94.71, 41.95, 41.22, 39.52, 25.23, 24.64 ppm.
^{19}F NMR (376 MHz, CDCl$_3$) δ -116.15
HRMS (ESI) m/z [M+H]$^+$: calcd. 291.1391, found. 291.1392.
IR (film): ν_{max} (cm^{-1}) 3076, 2999, 2921, 1729, 1633, 1462, 1390, 1251, 1204, 1015, 835, 724.
Optical rotation: $[\alpha]_D^{25}$ = 34.53 (c = 1.00, CHCl$_3$).
HPLC: DAICEL CHIRALPAK OX-3, hexane/i-PrOH = 9/1, flow rate: 0.5 mL/min, λ = 254 nm, t_R(minor) = 43.4 min, t_R(major) = 40.4 min, ee = 91%.

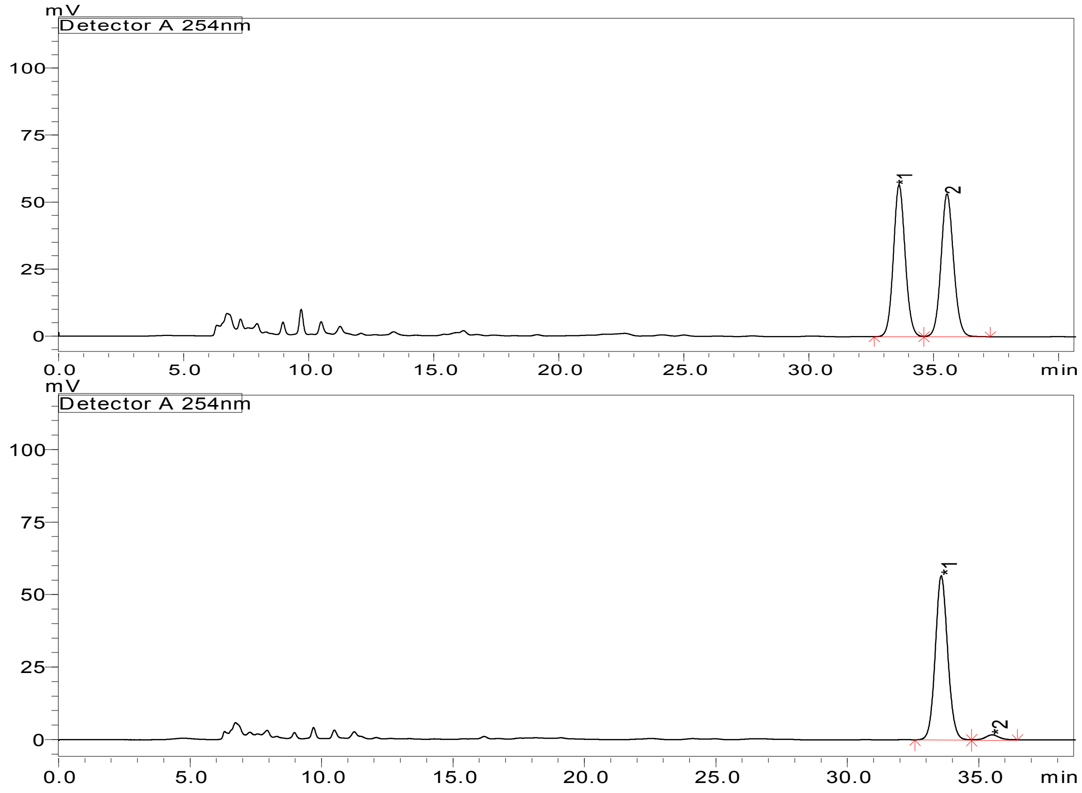

Peak#	Ret. Time	Area%
1	33.611	49.957
2	35.531	50.043

Peak#	Ret. Time	Area%
1	33.572	95.696
2	35.506	4.304

Supplementary Figure 18. HPLC chromatogram for compound **5d**

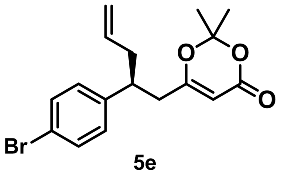

5e

(S)-6-(2-(4-bromophenyl)pent-4-en-1-yl)-2,2-dimethyl-4H-1,3-dioxin-4-one (**5e**)
According to GPE, 17 mg, colourless oil, 48% yield, 95% ee.
^{1}H NMR (400 MHz, $CDCl_3$) δ 7.42 (d, J = 8.2 Hz, 2H), 7.02 (d, J = 8.3 Hz, 2H), 5.67-5.55 (m, 1H), 5.09 (s, 1H), 5.05-4.96 (m, 2H), 3.04-2.93 (m, 1H), 2.68-2.59 (m, 1H), 2.50-2.41 (m, 1H), 2.39-2.28 (m, 2H), 1.58 (s, 3H), 1.47 (s, 3H) ppm.
^{13}C NMR (101 MHz, $CDCl_3$) δ 169.48, 160.90, 141.51, 135.01, 131.65, 129.18, 120.60, 117.59, 106.43, 94.76, 42.14, 40.94, 39.28, 29.70, 25.25, 24.71 ppm.
HRMS (ESI) m/z [M+H]$^+$: calcd. 351.0590, found. 351.0591.
IR (film): ν_{max} (cm^{-1}) 3082, 3000, 2930, 1724, 1631, 1388, 1247, 1201, 817.
Optical rotation: $[\alpha]_D^{25}$ = 25.93 (c = 2.00, $CHCl_3$).
HPLC: DAICEL CHIRALPAK OX-3, hexane/i-PrOH = 9/1, flow rate: 0.5 mL/min, λ = 254 nm, t_R(minor) = 36.8 min, t_R(major) = 34.6 min, ee = 95%.

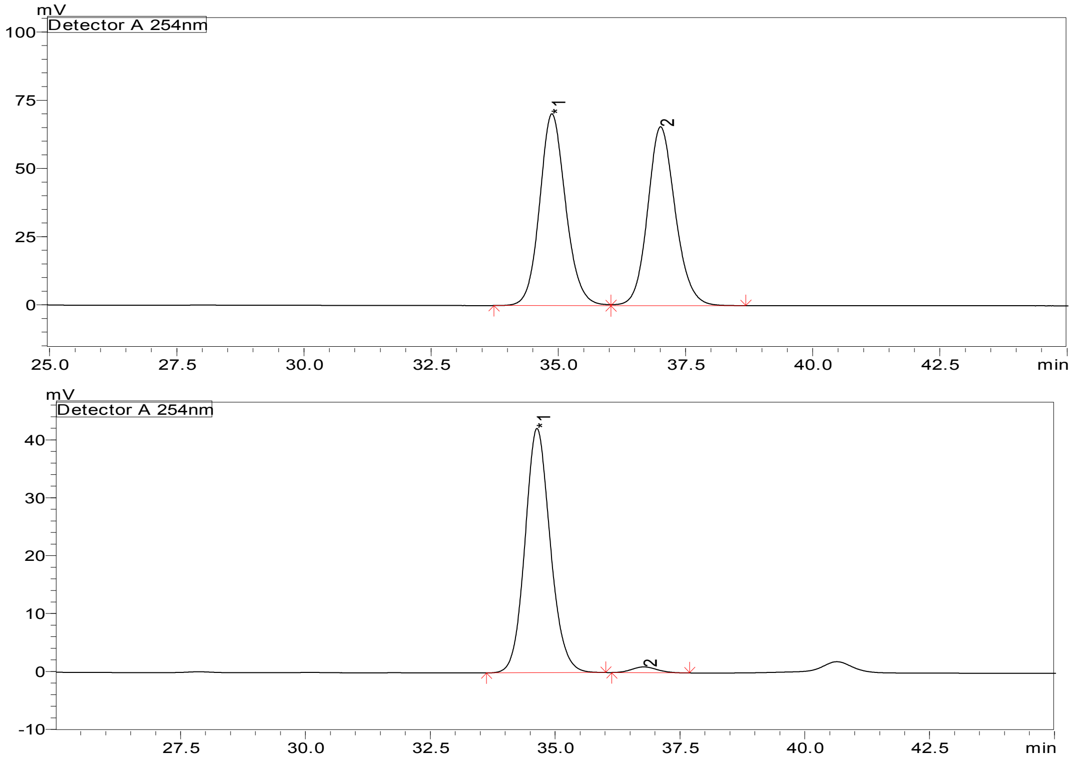

Peak#	Ret. Time	Area%
1	34.873	50.211
2	37.009	49.789

Peak#	Ret. Time	Area%
1	34.636	97.685
2	36.772	2.315

Supplementary Figure 19. HPLC chromatogram for compound **5e**

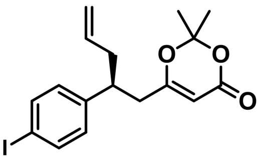

5f

(*S*)-6-(2-(4-iodophenyl)pent-4-en-1-yl)-2,2-dimethyl-4*H*-1,3-dioxin-4-one (**5f**)
According to GPE, 24 mg, colourless oil, 62% yield, 89% ee.
^{1}H NMR (400 MHz, CDCl$_3$) δ 7.62 (d, *J* = 8.3 Hz, 2H), 6.89 (d, *J* = 8.3 Hz, 2H), 5.67-5.54 (m, 1H), 5.09 (s, 1H), 5.05-4.96 (m, 2H), 3.01-2.91 (m, 1H), 2.67-2.58 (m, 1H), 2.49-2.40 (m, 1H), 2.39-2.29 (m, 2H), 1.58 (s, 3H), 1.47 (s, 3H) ppm.
^{13}C NMR (101 MHz, CDCl$_3$) δ 169.48, 160.92, 142.18, 137.59, 135.00, 129.48, 117.59, 106.43, 94.75, 91.99, 42.21, 40.89, 39.21, 25.24, 24.69 ppm.
HRMS (ESI) m/z [M+H]$^+$: calcd. 399.0452, found. 399.0451.
IR (film): ν_{max} (cm^{-1}) 2996, 2922, 1728, 1389,1250,1202,901,816.
Optical rotation: $[\alpha]_D^{25}$ = 29.25 (*c* = 1.00, CHCl$_3$).
HPLC: DAICEL CHIRALPAK AY-3, hexane/*i*-PrOH = 19/1, flow rate: 1 mL/min, λ = 254 nm, t_R(minor) = 17.3 min, t_R(major) = 18.4 min, ee = 89%.

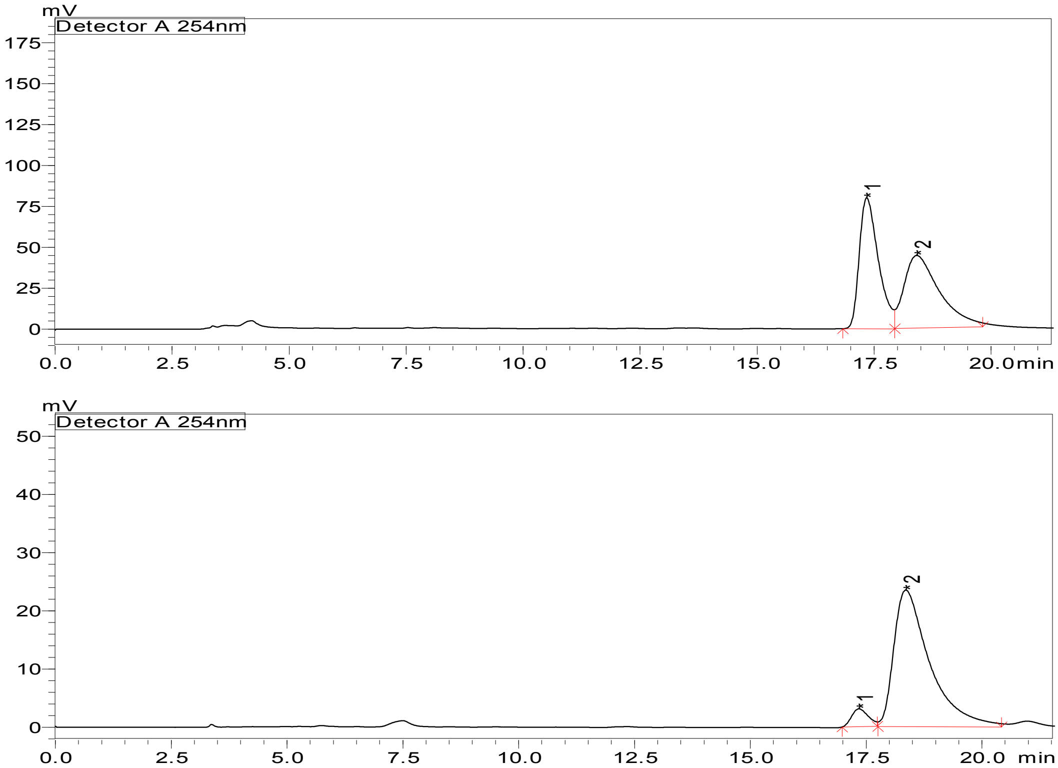

Peak#	Ret. Time	Area%
1	17.341	49.448
2	18.410	50.552

Peak#	Ret. Time	Area%
1	17.345	5.743
2	18.356	94.257

Supplementary Figure 20. HPLC chromatogram for compound **5f**

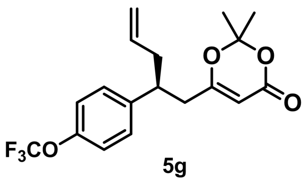

5g

(*S*)-2,2-dimethyl-6-(2-(4-(trifluoromethoxy)phenyl)pent-4-en-1-yl)-4*H*-1,3-dioxin-4-one (**5g**)

According to GPE, 28.5 mg, pale yellow oil, 81% yield, 93% ee.

^{1}H NMR (400 MHz, CDCl$_3$) δ 7.21-7.11 (m, 4H), 5.68-5.56 (m, 1H), 5.12 (s, 1H), 5.07-4.96 (m, 2H), 3.09-2.98 (m, 1H), 2.71-2.62 (m, 1H), 2.51-2.43 (m, 1H), 2.41-2.31 (m, 2H), 1.54 (s, 3H), 1.44 (s, 3H) ppm.

^{13}C NMR (101 MHz, CDCl$_3$) δ 169.51, 160.90, 147.97, 141.26, 128.77, 121.03, 120.42 (q, J = 257.0 Hz), 117.65, 106.43, 94.74, 42.09, 41.02, 39.28, 25.13, 24.56 ppm.

^{19}F NMR (376 MHz, CDCl$_3$) δ -57.98.

HRMS (ESI) m/z [M+H]$^+$: calcd. 357.1308, found. 357.1309.

IR (film): v_{max} (cm^{-1}) 2999, 1731, 1634, 1391, 1253, 1015, 850.

Optical rotation: $[\alpha]_D^{25}$ = 25.57 (c = 1.00, CHCl$_3$).

HPLC: DAICEL CHIRALPAK OX-3, hexane/*i*-PrOH = 24/1, flow rate: 1 mL/min, λ = 254 nm, t_R(minor) = 15.1 min, t_R(major) = 15.8 min, ee = 93%.

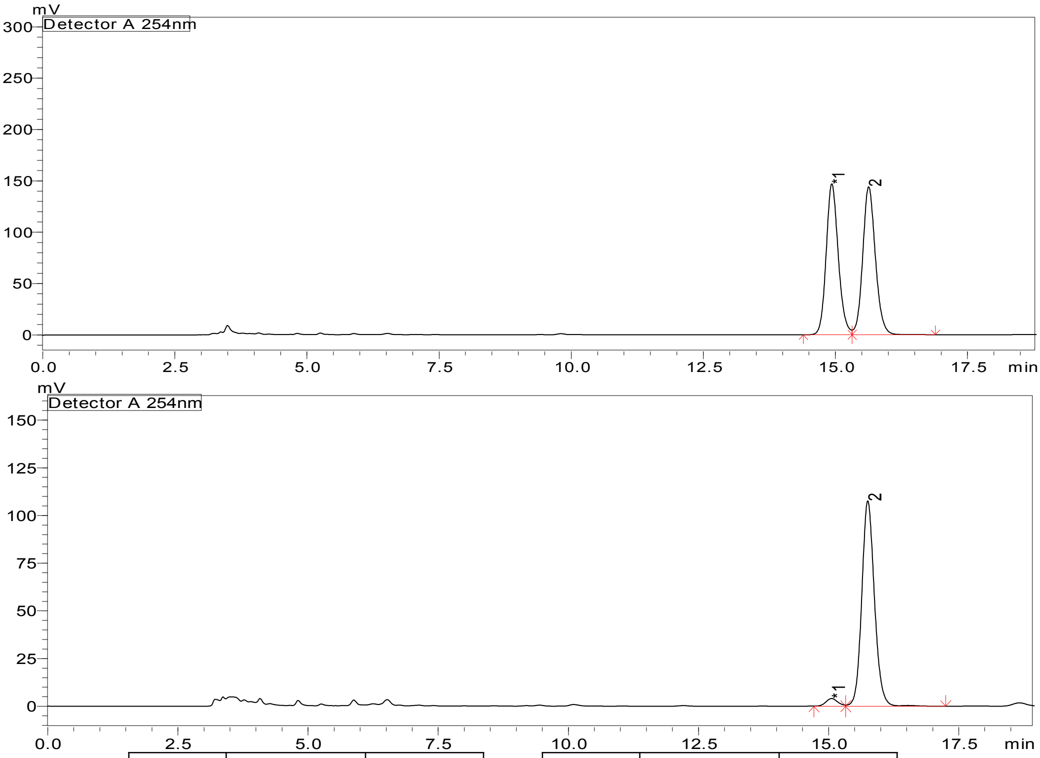

Peak#	Ret. Time	Area%
1	14.931	49.386
2	15.629	50.614

Peak#	Ret. Time	Area%
1	15.056	3.482
2	15.751	96.518

Supplementary Figure 21. HPLC chromatogram for compound **5g**

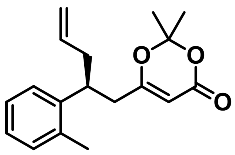

5h

(S)-2,2-dimethyl-6-(2-(o-tolyl)pent-4-en-1-yl)-4H-1,3-dioxin-4-one (**5h**)
According to GPE, 22 mg, pale yellow oil, 77% yield, 94% ee.
^{1}H NMR (400 MHz, $CDCl_3$) δ 7.20-7.06 (m, 4H), 5.73-5.59 (m, 1H), 5.12 (s, 1H), 5.06-4.96 (m, 2H), 3.42-3.29 (m, 1H), 2.73-2.62 (m, 1H), 2.58-2.46 (m, 1H), 2.41-2.27 (m, 5H), 1.55 (s, 3H), 1.33 (s, 3H) ppm.
^{13}C NMR (126 MHz, $CDCl_3$) δ 170.20, 161.11, 140.61, 135.60, 135.46, 130.48, 126.40, 126.24, 125.68, 117.18, 106.32, 94.55, 40.79, 38.73, 36.86, 25.17, 24.14, 19.66 ppm.
HRMS (ESI) m/z [M+H]$^{+}$: calcd. 287.1642 found. 287.1643.
IR (film): ν_{max} (cm^{-1}) 3074, 2998, 2919, 1729, 1632, 1491, 1399, 1251, 1203, 760, 727.
Optical rotation: $[\alpha]_D^{25}$ = 13.47 (c = 1.00, $CHCl_3$).
HPLC: DAICEL CHIRALPAK AY-3, hexane/i-PrOH = 9/1, flow rate: 0.5 mL/min, λ = 254 nm, t_R(minor) = 19.8 min, t_R(major) = 16.9 min, ee = 94%.

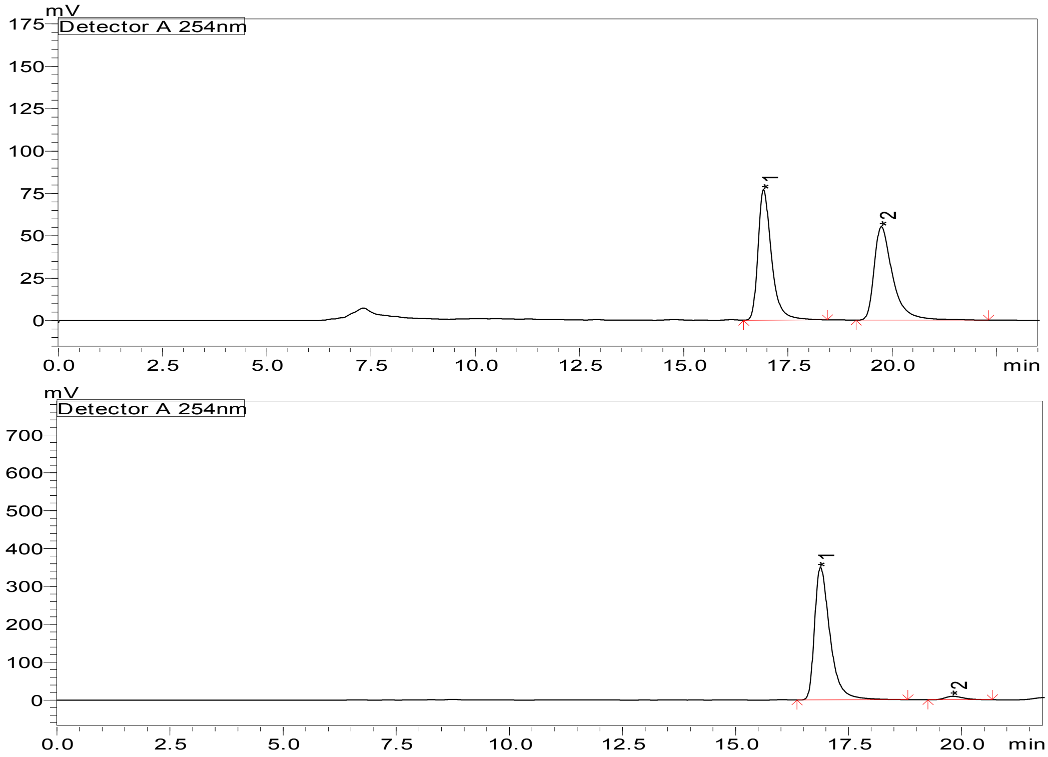

Peak#	Ret. Time	Area%
1	16.916	50.300
2	19.740	49.700

Peak#	Ret. Time	Area%
1	16.878	96.904
2	19.806	3.096

Supplementary Figure 22. HPLC chromatogram for compound **5h**

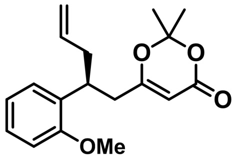

5i

(S)-6-(2-(2-methoxyphenyl)pent-4-en-1-yl)-2,2-dimethyl-4H-1,3-dioxin-4-one (**5i**)
According to GPE, 21 mg, pale yellow oil, 70% yield, 93% ee.
^{1}H NMR (400 MHz, $CDCl_3$) δ 7.22-7.14 (m, 1H), 7.06 (d, J = 7.5 Hz, 1H), 6.92-6.80 (m, 2H), 5.73-5.60 (m, 1H), 5.08 (s, 1H), 5.03-4.92 (m, 2H), 3.82 (s, 3H), 3.49-3.38 (m, 1H), 2.60 (d, J = 7.8 Hz, 2H), 2.49-2.29 (m, 2H), 1.55 (s, 3H), 1.41 (s, 3H) ppm.
^{13}C NMR (151 MHz, $CDCl_3$) δ 170.83, 161.37, 157.16, 136.16, 130.37, 128.16, 127.72, 120.45, 116.64, 110.62, 106.21, 94.19, 55.18, 39.45, 37.82, 36.44, 25.28, 24.31 ppm.
HRMS (ESI) m/z [M+H]$^{+}$: calcd. 303.1591, found. 303.1591.
IR (film): ν_{max} (cm^{-1}) 3074, 2998, 1728, 1632, 1463, 1390, 1204, 1015, 755.
Optical rotation: $[\alpha]_D^{25}$ = 24.14 (c = 1.00, $CHCl_3$).
HPLC: DAICEL CHIRALPAK AD-H, hexane/i-PrOH = 47/3, flow rate: 0.5 mL/min, λ = 254 nm, t_R(minor) = 16.6 min, t_R(major) = 13.5 min, ee = 93%.

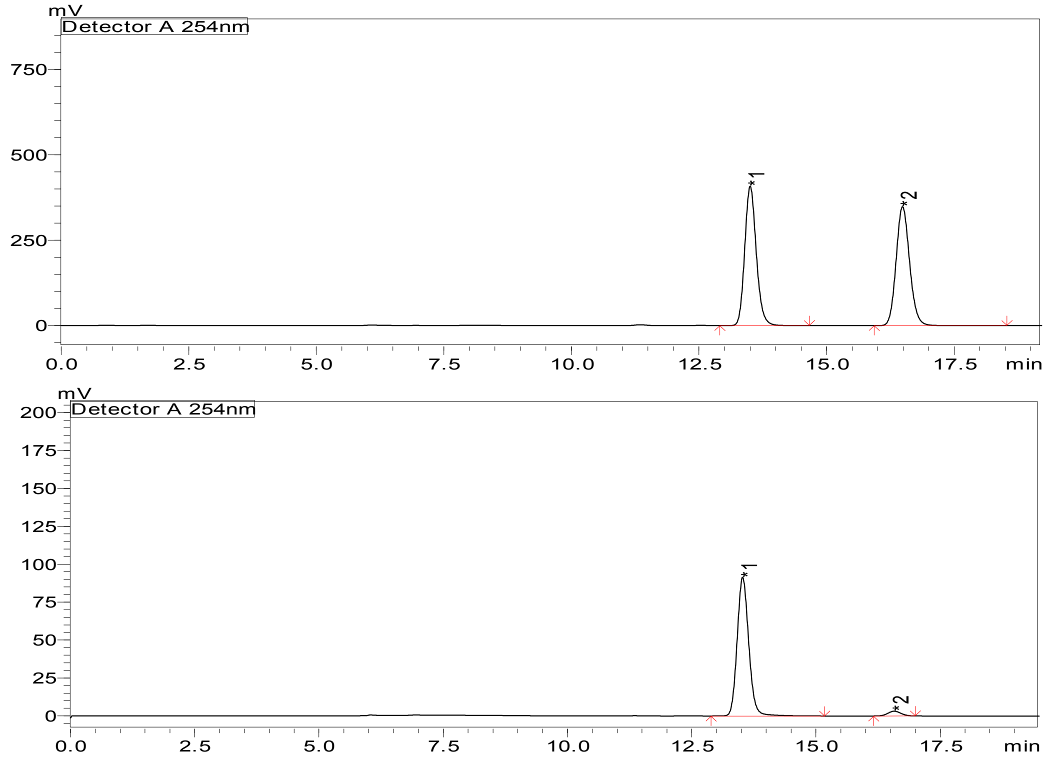

Peak#	Ret. Time	Area%
1	13.502	50.037
2	16.488	49.963

Peak#	Ret. Time	Area%
1	13.525	96.255
2	16.575	3.745

Supplementary Figure 23. HPLC chromatogram for compound **5i**

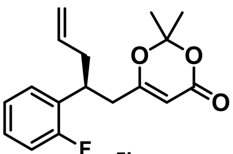

5j

(*S*)-6-(2-(2-fluorophenyl)pent-4-en-1-yl)-2,2-dimethyl-4*H*-1,3-dioxin-4-one (**5j**)
According to GPE, 20 mg, pale yellow oil, 69% yield, 94% ee.

^{1}H NMR (400 MHz, CDCl$_3$) δ 7.22-7.07 (m, 3H), 7.04-6.96 (m, 1H), 5.72-5.59 (m, 1H), 5.10 (s, 1H), 5.05-4.97 (m, 2H), 3.41-3.28 (m, 1H), 2.71-2.53 (m, 2H), 2.46-2.38 (m, 2H), 1.57 (s, 3H), 1.41 (s, 3H) ppm.

^{13}C NMR (151 MHz, CDCl$_3$) δ 169.72, 161.07, 160.83 (d, *J* = 245.5 Hz), 135.22, 129.10 (d, *J* = 14.4 Hz), 128.85 (d, *J* = 5.2 Hz), 128.38 (d, *J* = 9.1 Hz), 124.15 (d, *J* = 3.1 Hz), 117.38, 115.66 (d, *J* = 22.5 Hz), 106.42, 94.52, 39.85, 38.10, 36.28, 25.40, 24.16 ppm.

^{19}F NMR (565 MHz, CDCl$_3$) δ -117.74

HRMS (ESI) m/z [M+H]$^+$: calcd. 291.1391, found. 291.1393.

IR (film): ν_{max} (cm^{-1}) 3078, 2999, 1732, 1634, 1455, 1391,1252, 1204, 1015, 824, 759.

Optical rotation: $[\alpha]_D^{25}$ = 35.08 (*c* = 1.00, CHCl$_3$).

HPLC: DAICEL CHIRALPAK OX-3, hexane/*i*-PrOH = 9/1, flow rate: 0.5 mL/min, λ = 254 nm, t_R(minor) = 26.3 min, t_R(major) = 27.2 min, ee = 94%.

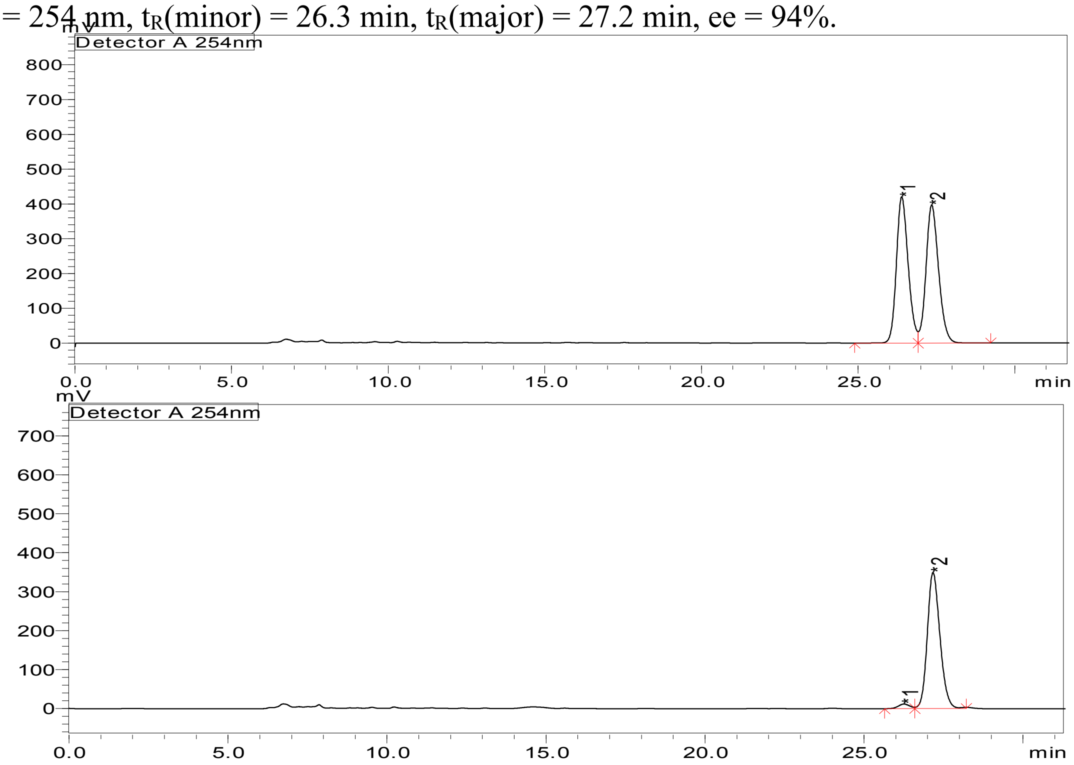

Peak#	Ret. Time	Area%
1	26.390	49.795
2	27.347	50.205

Peak#	Ret. Time	Area%
1	26.254	2.971
2	27.168	97.029

Supplementary Figure 24. HPLC chromatogram for compound **5j**

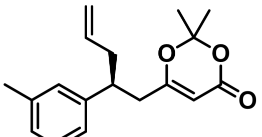

5k

(S)-2,2-dimethyl-6-(2-(m-tolyl)pent-4-en-1-yl)-4H-1,3-dioxin-4-one (**5k**)
According to GPE, 22 mg, pale yellow oil, 77% yield, 94% ee.
^{1}H NMR (400 MHz, CDCl$_3$) δ 7.21-7.13 (m, 1H), 7.02 (d, J = 7.6 Hz, 1H), 6.96-6.89 (m, 2H), 5.73-5.58 (m, 1H), 5.14-4.93 (m, 3H), 3.01-2.90 (m, 1H), 2.67-2.58 (m, 1H), 2.51-2.35 (m, 3H), 2.32 (s, 3H), 1.57 (s, 3H), 1.46 (s, 3H) ppm.
^{13}C NMR (101 MHz, CDCl$_3$) δ 170.14, 161.15, 142.45, 137.98, 135.66, 128.36, 128.20, 127.56, 124.39, 117.04, 106.33, 94.59, 42.53, 41.10, 39.41, 25.27, 24.56, 21.43 ppm.
HRMS (ESI) m/z [M+H]$^+$: calcd. 287.1642, found. 287.1643.
IR (film): ν_{max} (cm^{-1}) 2998, 2921, 1730,1634, 1458, 1390, 1251, 1204, 1014, 786, 107.
Optical rotation: $[\alpha]_D^{25}$ = 31.81 (c = 1.00, CHCl$_3$).
HPLC: DAICEL CHIRALPAK AY-3, hexane/i-PrOH = 48/1, flow rate: 1 mL/min, λ = 254 nm, t_R(minor) = 18.3 min, t_R(major) = 19.3 min, ee = 94%.

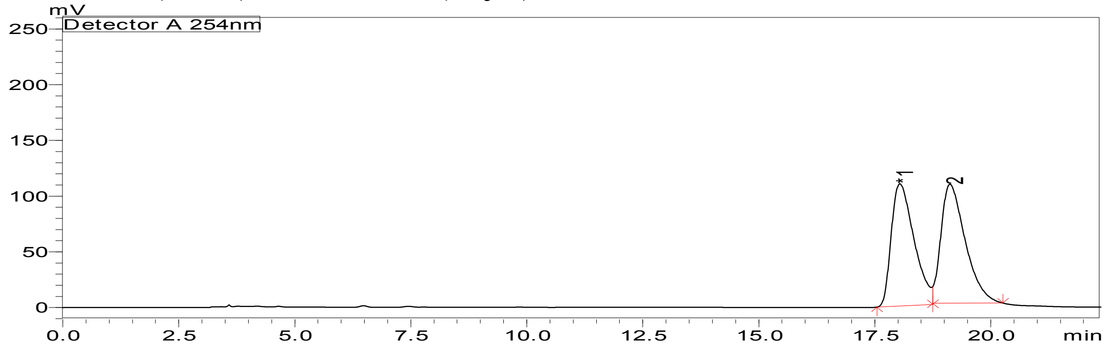

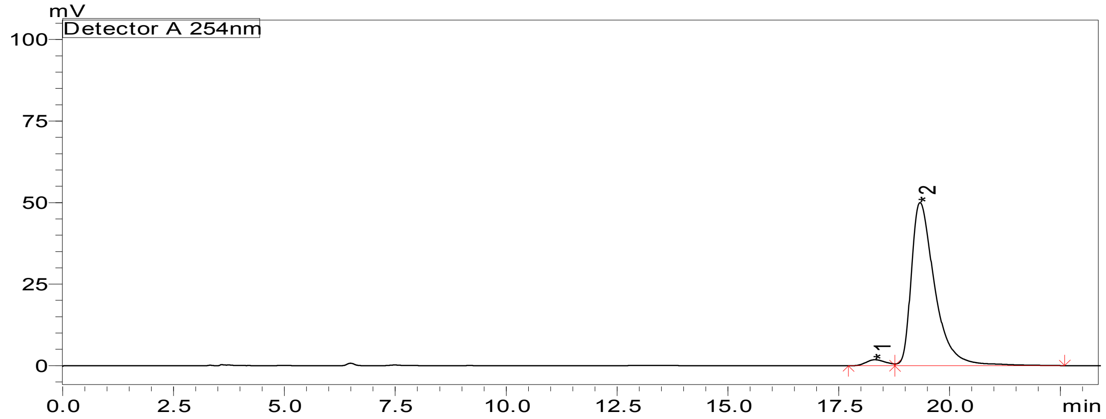

Peak#	Ret. Time	Area%
1	18.040	49.141
2	19.120	50.859

Peak#	Ret. Time	Area%
1	18.316	3.054
2	19.333	96.946

Supplementary Figure 25. HPLC chromatogram for compound **5k**

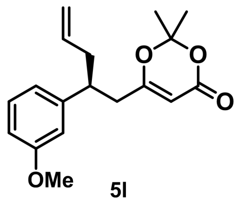

5l

(S)-6-(2-(3-methoxyphenyl)pent-4-en-1-yl)-2,2-dimethyl-4H-1,3-dioxin-4-one (**5l**)
According to GPE, 21 mg, pale yellow oil, 70% yield, 92% ee.
^{1}H NMR (400 MHz, $CDCl_3$) δ 7.24-7.18 (m, 1H), 6.78-6.66 (m, 3H), 5.72-5.58 (m, 1H), 5.09 (s, 1H), 5.06-4.96 (m, 2H), 3.79 (s, 3H), 3.02-2.91 (m, 1H), 2.68-2.58 (m, 1H), 2.50-2.31 (m, 3H), 1.58 (s, 3H), 1.47 (s, 3H) ppm.
^{13}C NMR (151 MHz, $CDCl_3$) δ 169.97, 161.12, 159.65, 144.18, 135.51, 129.50, 119.81, 117.16, 113.72, 111.53, 106.37, 94.66, 55.14, 42.60, 41.04, 39.35, 25.30, 24.58 ppm.
HRMS (ESI) m/z [M+H]$^+$: calcd. 303.1591, found. 303.1592.
IR (film): ν_{max} (cm^{-1}) 3451, 3075, 2998, 1728, 1632, 1455, 1390, 1204, 870, 785, 703.
Optical rotation: $[\alpha]_D^{25}$ = 35.89 (c = 1.00, $CHCl_3$).
HPLC: DAICEL CHIRALPAK IF-3, hexane/i-PrOH = 9/1, flow rate: 0.5 mL/min, λ = 254 nm, t_R(minor) = 21.0 min, t_R(major) = 19.8 min, ee = 92%.

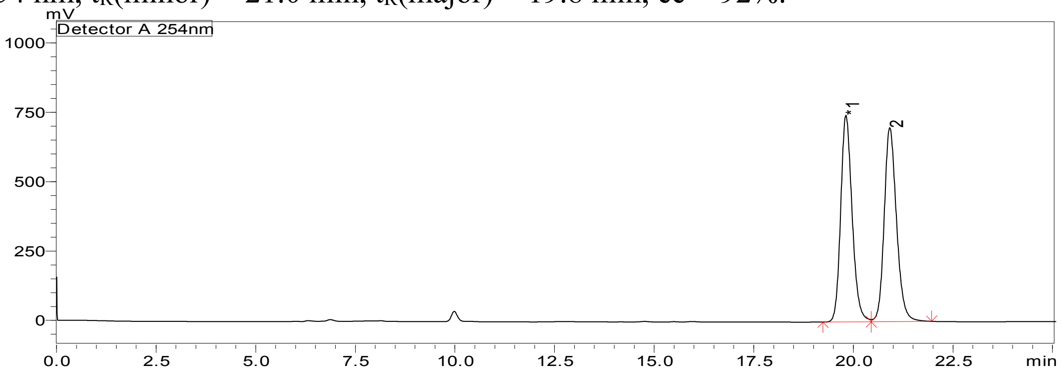

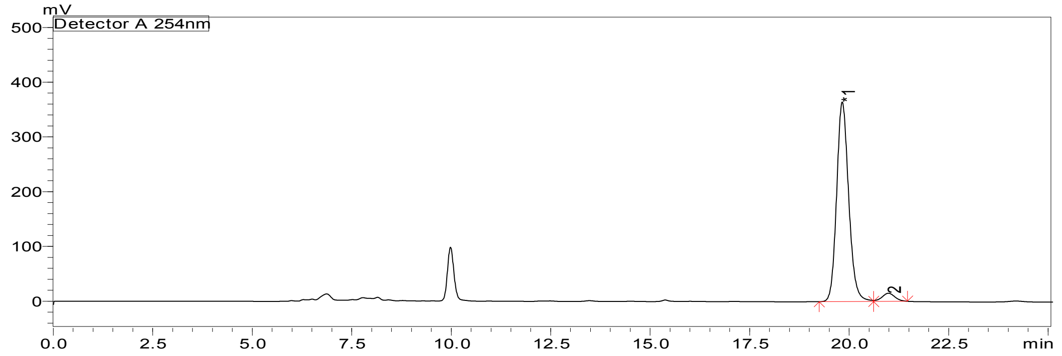

Peak#	Ret. Time	Area%
1	19.812	49.770
2	20.913	50.230

Peak#	Ret. Time	Area%
1	19.827	95.836
2	20.987	4.164

Supplementary Figure 26. HPLC chromatogram for compound **5l**

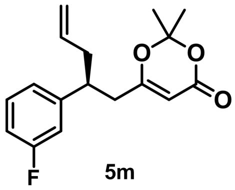

5m

(*S*)-6-(2-(3-fluorophenyl)pent-4-en-1-yl)-2,2-dimethyl-4*H*-1,3-dioxin-4-one (**5m**)
According to GPE, 16 mg, yellow oil, 52% yield, 92% ee.
^{1}H NMR (400 MHz, $CDCl_3$) δ 7.30-7.23 (m, 1H), 6.96-6.81 (m, 3H), 5.71-5.55 (m, 1H), 5.10 (s, 1H), 5.06-4.95 (m, 2H), 3.07-2.95 (m, 1H), 2.73-2.53 (m, 1H), 2.53-2.42 (m, 1H), 2.41-2.33 (m, 2H), 1.58 (s, 3H), 1.47 (s, 3H) ppm.
^{13}C NMR (101 MHz, $CDCl_3$) δ 169.50, 162.84 (d, J = 246.2 Hz), 160.98, 145.13 (d, J = 6.7 Hz), 135.00, 130.01 (d, J = 8.3 Hz), 123.18 (d, J = 2.7 Hz), 117.56, 114.26 (d, J = 21.2 Hz), 113.78 (d, J = 21.0 Hz), 106.42, 94.71, 42.35, 40.94, 39.23, 25.24, 24.58 ppm.
^{19}F NMR (376 MHz, $CDCl_3$) δ -113.15.
HRMS (ESI) m/z [M+H]$^+$: calcd. 291.1391, found. 291.1391.
IR (film): ν_{max} (cm^{-1}) 3854, 2924, 1728, 1636, 1389, 1202, 1014.
Optical rotation: $[\alpha]_D^{25}$ = 25.65 (c = 1.00, $CHCl_3$).
HPLC: DAICEL CHIRALPAK OX-3, hexane/*i*-PrOH = 9/1, flow rate: 0.5 mL/min, λ = 254 nm, t_R(minor) = 32.3 min, t_R(major) = 34.1 min, ee = 92%.

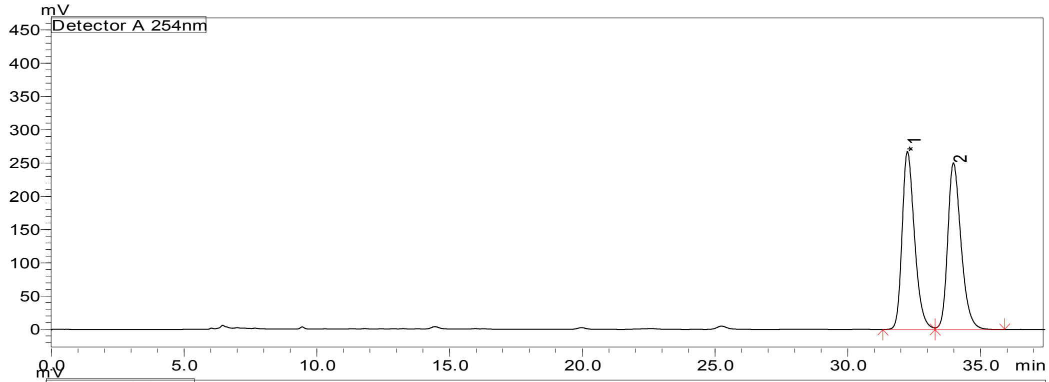

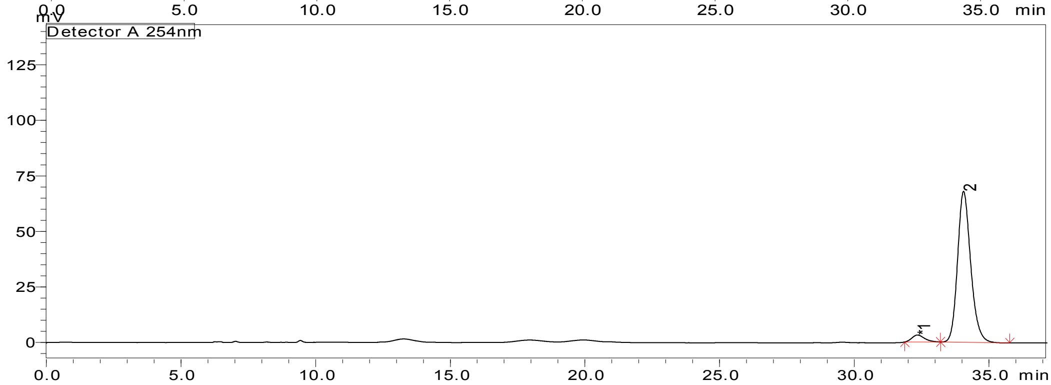

Peak#	Ret. Time	Area%
1	32.245	49.860
2	33.978	50.140

Peak#	Ret. Time	Area%
1	32.348	4.225
2	34.058	95.775

Supplementary Figure 27. HPLC chromatogram for compound **5m**

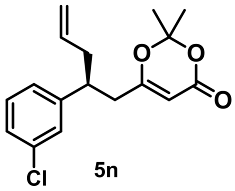

5n

(S)-6-(2-(3-chlorophenyl)pent-4-en-1-yl)-2,2-dimethyl-4H-1,3-dioxin-4-one (**5n**)
According to GPE, 22 mg, pale yellow oil, 72% yield, 92% ee.

^{1}H NMR (400 MHz, $CDCl_3$) δ 7.25-7.18 (m, 2H), 7.13 (s, 1H), 7.06-7.00 (m, 1H), 5.68-5.57 (m, 1H), 5.10 (s, 1H), 5.06-4.97 (m, 2H), 3.05-2.92 (m, 1H), 2.70-2.60 (m, 1H), 2.50-2.42 (m, 1H), 2.41-2.32 (m, 2H), 1.58 (s, 3H), 1.47 (s, 3H) ppm.

^{13}C NMR (101 MHz, $CDCl_3$) δ 169.44, 160.94, 144.61, 134.94, 134.30, 129.81, 127.67, 127.06, 125.63, 117.63, 106.45, 94.73, 42.37, 40.83, 39.23, 25.24, 24.61 ppm.

HRMS (ESI) m/z [M+H]$^+$: calcd. 307.1095, found. 307.1096.

IR (film): ν_{max} (cm^{-1}) 3076, 2998, 1732, 1636, 1462, 1390, 1252, 1203, 877, 786, 749.

Optical rotation: $[\alpha]_D^{25}$ = 33.36 (c = 1.00, $CHCl_3$).

HPLC: DAICEL CHIRALPAK OX-3, hexane/i-PrOH = 97/3, flow rate: 1 mL/min, λ = 254 nm, t_R(minor) = 34.1 min, t_R(major) = 34.8min, ee = 92%.

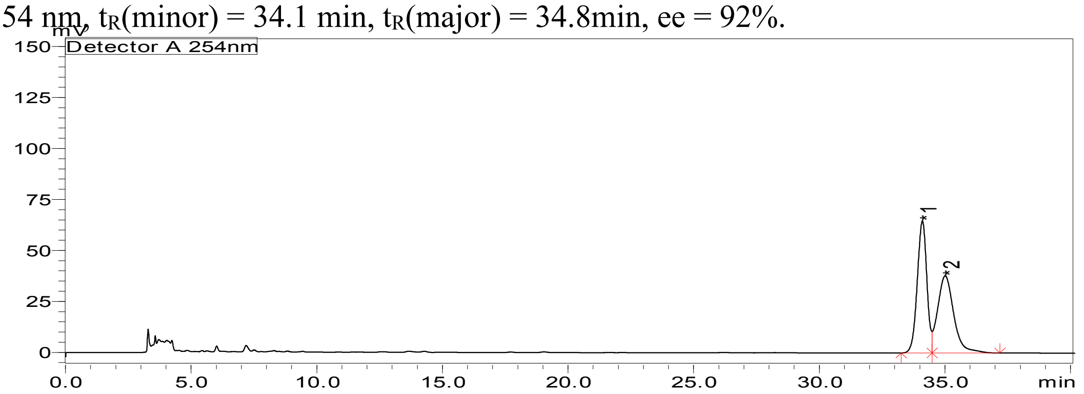

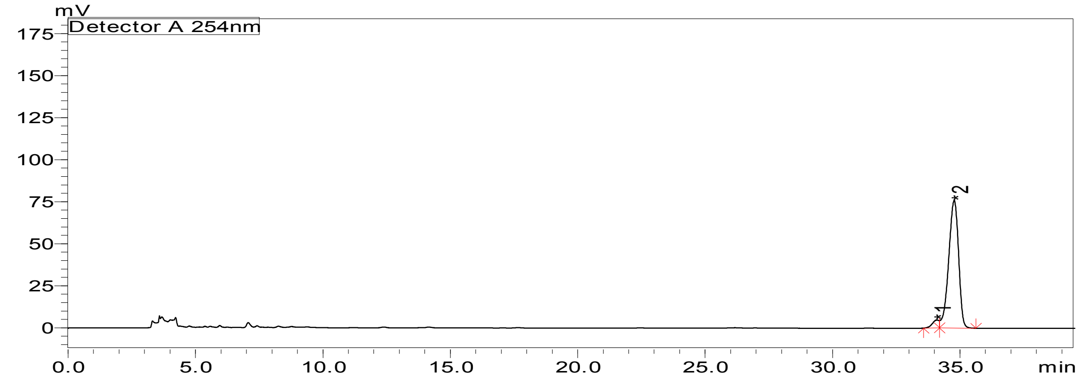

Peak#	Ret. Time	Area%
1	34.100	50.636
2	35.008	49.364

Peak#	Ret. Time	Area%
1	34.089	4.033
2	34.773	95.967

Supplementary Figure 28. HPLC chromatogram for compound **5n**

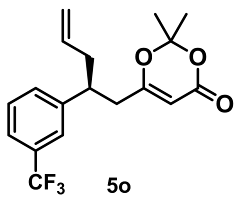

5o

(*S*)-2,2-dimethyl-6-(2-(3-(trifluoromethyl)phenyl)pent-4-en-1-yl)-4*H*-1,3-dioxin-4-one (**5o**)

According to GPE, 20 mg, pale yellow oil, 59% yield, 92% ee.

^{1}H NMR (400 MHz, $CDCl_3$) δ 7.52-7.31 (m, 4H), 5.70-5.55 (m, 1H), 5.10 (s, 1H), 5.05-4.96 (m, 2H), 3.15-3.04 (m, 1H), 2.74-2.64 (m, 1H), 2.54-2.45 (m, 1H), 2.44-2.34 (m, 2H), 1.55 (s, 3H), 1.44 (s, 3H) ppm.

^{13}C NMR (101 MHz, $CDCl_3$) δ 169.28, 160.88, 143.50, 134.73, 130.80 (q, J = 32.1 Hz), 130.73, 129.04, 124.38 (d, J = 3.7 Hz), 124.00 (q, J = 272.2 Hz), 123.78 (d, J = 3.8 Hz), 117.87, 106.47, 94.78, 42.52, 40.87, 39.18, 25.18, 24.54 ppm.

^{19}F NMR (376 MHz, $CDCl_3$) δ -62.77.

HRMS (ESI) m/z $[M+H]^+$: calcd. 341.1359, found. 341.1341.

IR (film): ν_{max} (cm^{-1}) 3086, 3000, 2927,1729, 1637, 1391, 1252, 1204, 1016, 920, 805, 705.

Optical rotation: $[\alpha]_D^{25}$ = 18.44 (c = 1.00, $CHCl_3$).

HPLC: DAICEL CHIRALPAK OX-3, hexane/*i*-PrOH = 9/1, flow rate: 0.5 mL/min, λ = 254 nm, t_R(minor) = 25.0 min, t_R(major) = 26.0 min, ee = 92%.

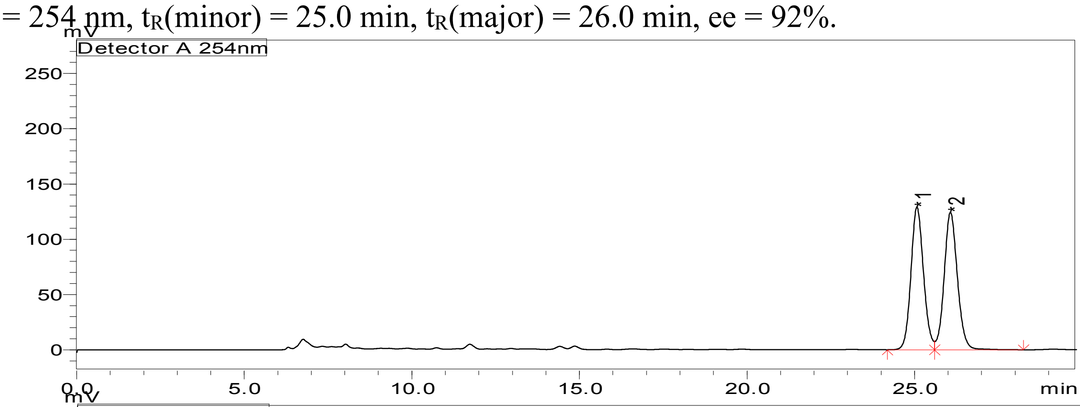

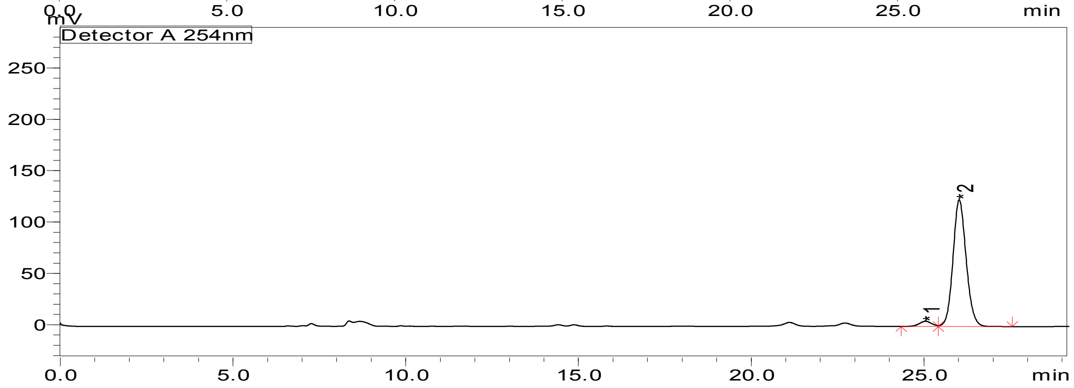

Peak#	Ret. Time	Area%
1	25.069	49.594
2	26.069	50.406

Peak#	Ret. Time	Area%
1	25.044	3.821
2	26.020	96.179

Supplementary Figure 29. HPLC chromatogram for compound **5o**

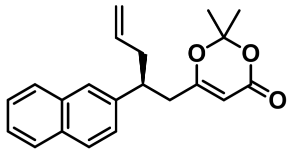

5p

(*S*)-2,2-dimethyl-6-(2-(naphthalen-2-yl)pent-4-en-1-yl)-4*H*-1,3-dioxin-4-one (**5p**)
According to GPE, 20 mg, pale yellow oil, 62% yield, 93% ee.
^{1}H NMR (400 MHz, CDCl$_3$) δ 7.84-7.74 (m, 3H), 7.57 (s, 1H), 7.51-7.41 (m, 2H), 7.29 (dd, *J* = 8.5, 1.5 Hz, 1H), 5.73-5.59 (m, 1H), 5.11 (s, 1H), 5.06-4.94 (m, 2H), 3.25-3.13 (m, 1H), 2.77-2.68 (m, 1H), 2.65-2.55 (m, 1H), 2.51-2.42 (m, 2H), 1.54 (s, 3H), 1.41 (s, 3H) ppm.
^{13}C NMR (101 MHz, CDCl$_3$) δ 169.93, 161.03, 139.91, 135.45, 133.32, 132.45, 128.33, 127.64, 127.49, 126.28, 126.19, 125.65, 125.34, 117.27, 106.38, 94.66, 42.77, 41.00, 39.41, 25.28, 24.60 ppm.
HRMS (ESI) m/z [M+H]$^+$: calcd. 323.1642, found. 323.1641.
IR (film): ν_{max} (cm^{-1}) 3055, 2998, 1727, 1631, 1390, 1251, 1203, 858.
Optical rotation: $[\alpha]_D^{25}$ = 41.67 (*c* = 1.00, CHCl$_3$).
HPLC: DAICEL CHIRALPAK AD-H, hexane/*i*-PrOH = 22/3, flow rate: 0.5 mL/min, λ = 254 nm, t_R(minor) = 12.6 min, t_R(major) = 11.7 min, ee = 93%.

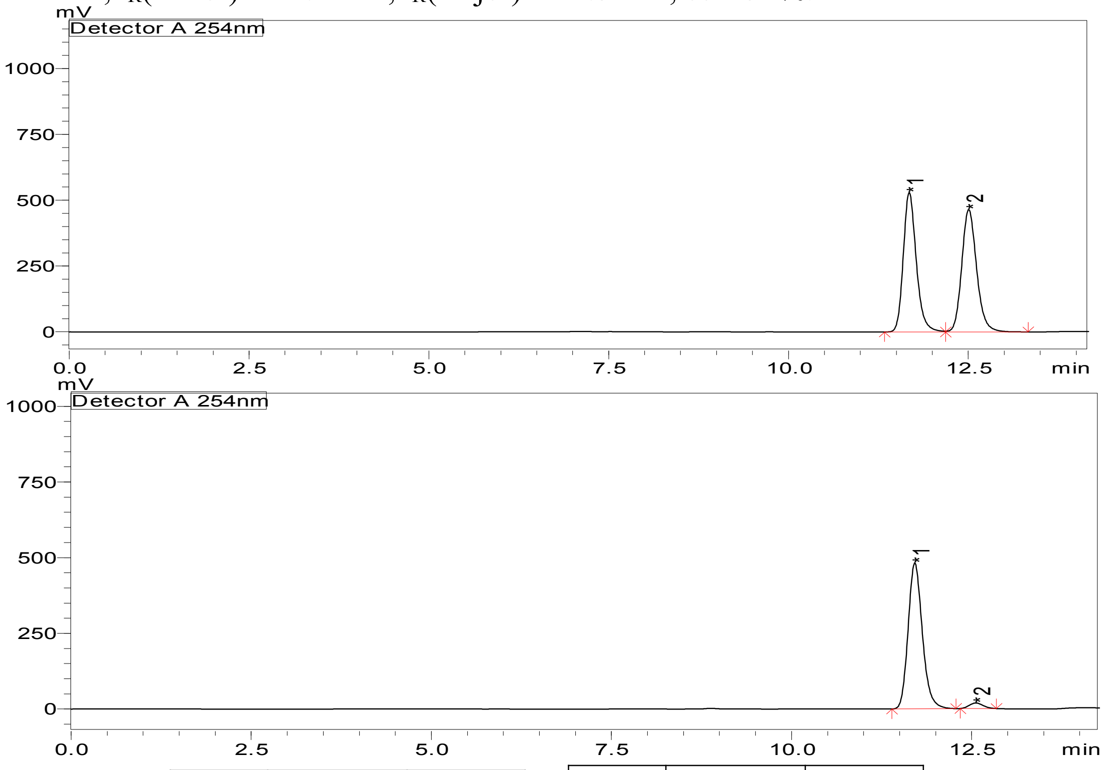

Peak#	Ret. Time	Area%
1	11.678	49.853
2	12.506	50.147

Peak#	Ret. Time	Area%
1	11.710	96.444
2	12.556	3.556

Supplementary Figure 30. HPLC chromatogram for compound **5p**

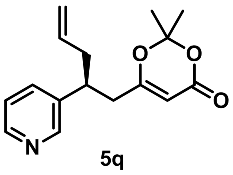

5q

(*S*)-2,2-dimethyl-6-(2-(pyridin-3-yl)pent-4-en-1-yl)-4*H*-1,3-dioxin-4-one (**5q**)
According to GPE, 16 mg, pale yellow oil, 59% yield, 81% ee.
^{1}H NMR (400 MHz, $CDCl_3$) δ 8.66 (s, 1H), 7.58-7.22 (m, 3H), 5.70-5.54 (m, 1H), 5.12 (s, 1H), 5.06-4.94 (m, 2H), 3.08 (s, 1H), 2.79-2.63 (m, 1H), 2.55-2.36 (m, 3H), 1.57 (s, 3H), 1.44 (s, 3H) ppm.
^{13}C NMR (101 MHz, $CDCl_3$) δ 169.06, 160.77, 134.53, 118.05, 106.52, 94.88, 40.78, 40.23, 39.02, 25.24, 24.60 ppm.
HRMS (ESI) m/z $[M+H]^+$: calcd. 274.1438, found. 274.1438.
IR (film): ν_{max} (cm^{-1}) 2959, 2924, 1729, 1631, 1390, 1261, 1014, 802.
Optical rotation: $[\alpha]_D^{25}$ = 37.2 (*c* = 0.25, $CHCl_3$).
HPLC: DAICEL CHIRALPAK OD-H, hexane/*i*-PrOH = 9/1, flow rate: 1.0 mL/min, λ = 254 nm, t_R(minor) = 23.4 min, t_R(major) = 24.9 min, ee = 81%.

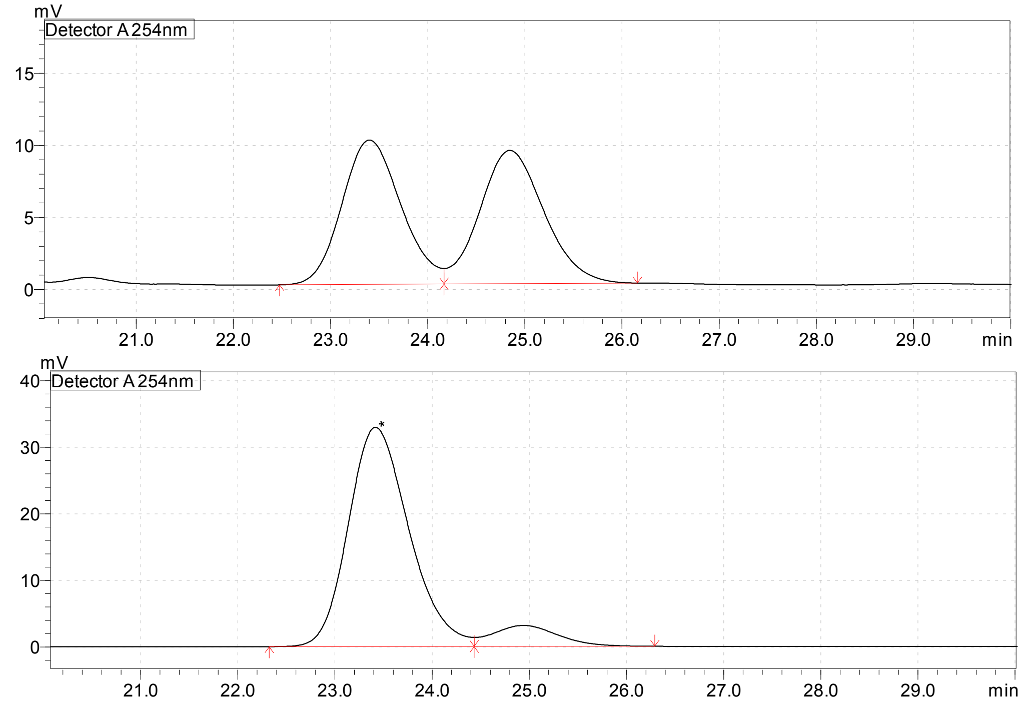

Peak#	Ret. Time	Area%
1	23.400	49.931
2	24.845	50.069

Peak#	Ret. Time	Area%
1	23.418	90.642
2	24.939	9.358

Supplementary Figure 31. HPLC chromatogram for compound **5q**

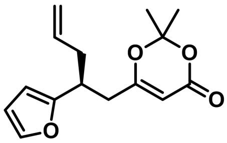

5r

(*S*)-6-(2-(furan-2-yl)pent-4-en-1-yl)-2,2-dimethyl-4*H*-1,3-dioxin-4-one (**5r**)
According to GPE, 17.6 mg, pale yellow oil, 67% yield, 90% ee.
^{1}H NMR (400 MHz, $CDCl_3$) δ 7.31 (d, *J* = 1.0 Hz, 1H), 6.27 (dd, *J* = 3.1, 1.8 Hz, 1H), 6.02 (d, *J* = 3.1 Hz, 1H), 5.75-5.62 (m, 1H), 5.14 (s, 1H), 5.09-5.01 (m, 2H), 3.20-3.10 (m, 1H), 2.60-2.53 (m, 2H), 2.48-2.32 (m, 2H), 1.64 (s, 3H), 1.57 (s, 3H) ppm.
^{13}C NMR (101 MHz, $CDCl_3$) δ 169.54, 161.04, 155.55, 141.29, 134.95, 117.42, 109.94, 106.39, 105.82, 94.56, 38.23, 37.29, 35.91, 25.36, 24.53 ppm.
HRMS (ESI) m/z [M+H]$^+$: calcd. 263.1278, found. 263.1279.
IR (film): v_{max} (cm^{-1}) 3116, 2999, 1734, 1635, 1390, 1253, 1204, 1014, 885,734.
Optical rotation: $[\alpha]_D^{25}$ = 37.50 (*c* = 1.00, $CHCl_3$).
HPLC: DAICEL CHIRALPAK OD-H, hexane/*i*-PrOH = 99/1, flow rate: 0.5 mL/min, λ = 254 nm, t_R(minor) = 15.6 min, t_R(major) = 16.4 min, ee = 90%.

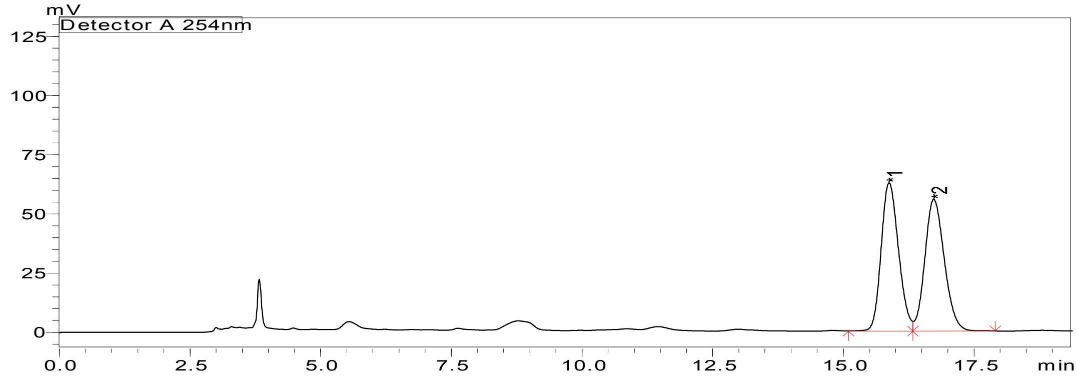

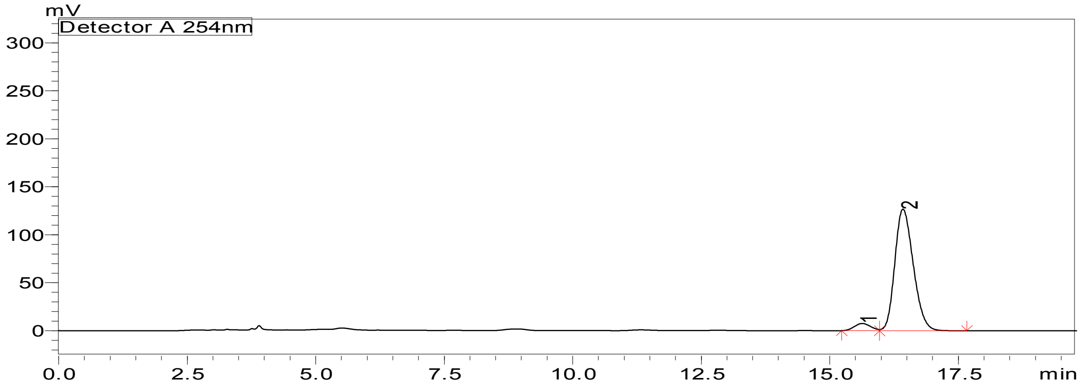

Peak#	Ret. Time	Area%
1	15.876	50.082
2	16.731	49.918

Peak#	Ret. Time	Area%
1	15.630	4.967
2	16.423	95.033

Supplementary Figure 32. HPLC chromatogram for compound **5r**

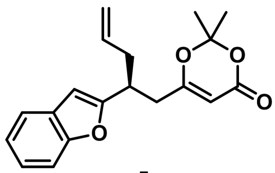

5s

(*S*)-6-(2-(benzofuran-2-yl)pent-4-en-1-yl)-2,2-dimethyl-4*H*-1,3-dioxin-4-one (**5s**)
According to GPE, 19 mg, pale yellow oil, 61% yield, 95% ee.

^{1}H NMR (400 MHz, CDCl$_3$) δ 7.52-7.45 (m, 1H), 7.41 (d, *J* = 8.0 Hz, 1H), 7.25-7.14 (m, 2H), 6.42 (s, 1H), 5.78-5.65 (m, 1H), 5.20 (s, 1H), 5.13-5.02 (m, 2H), 3.34-3.22 (m, 1H), 2.75-2.61 (m, 2H), 2.59-2.41 (m, 2H), 1.62 (s, 3H), 1.56 (s, 3H) ppm.

^{13}C NMR (101 MHz, CDCl$_3$) δ 169.17, 160.96, 158.54, 154.54, 134.63, 128.15, 123.74, 122.70, 120.58, 117.87, 110.83, 106.51, 103.00, 94.75, 37.85, 37.06, 36.38, 25.37, 24.66 ppm.

HRMS (ESI) m/z [M+H]$^+$: calcd. 313.1434, found. 313.1434.

IR (film): ν_{max} (cm^{-1}) 3076, 2998, 1729, 1635, 1390, 1252, 1204, 1015, 882, 752.

Optical rotation: $[\alpha]_D^{25}$ = 61.57 (*c* = 1.00, CHCl$_3$).

HPLC: DAICEL CHIRALPAK OX-3, hexane/*i*-PrOH = 97/3, flow rate: 1 mL/min, λ = 254 nm, t_R(minor) = 33.9 min, t_R(major) = 35.3 min, ee = 95 %.

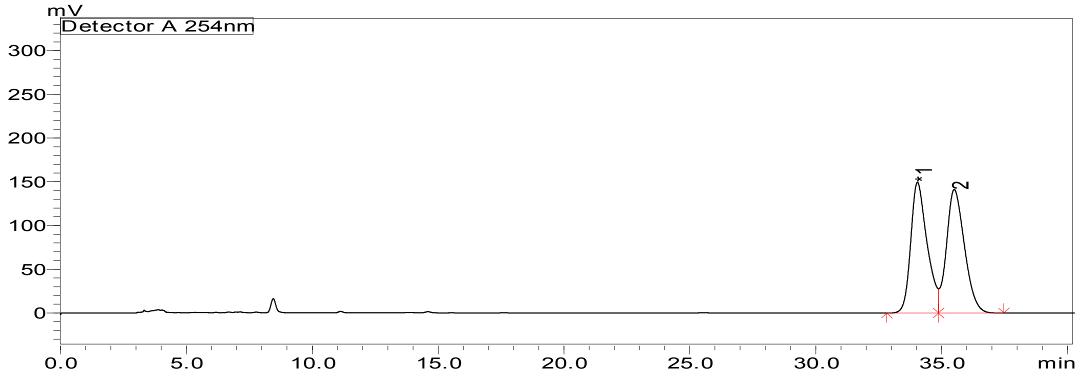

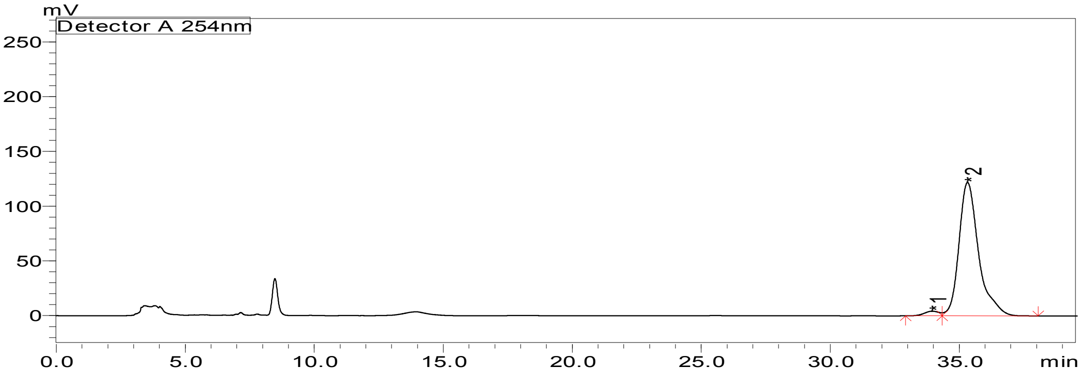

Peak#	Ret. Time	Area%
1	34.041	49.131
2	35.504	50.869

Peak#	Ret. Time	Area%
1	33.949	2.604
2	35.322	97.396

Supplementary Figure 33. HPLC chromatogram for compound **5s**

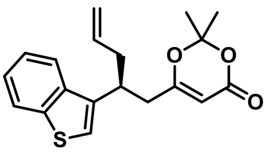

5t

(*S*)-6-(2-(benzo[*b*]thiophen-3-yl)pent-4-en-1-yl)-2,2-dimethyl-4*H*-1,3-dioxin-4-one **(5t)**

According to GPE, 24 mg, yellow oil, 73% yield, 91% ee.

^{1}H NMR (400 MHz, $CDCl_3$) δ 7.86 (d, *J* = 7.4 Hz, 1H), 7.77 (d, *J* = 7.5 Hz, 1H), 7.44-7.32 (m, 2H), 7.11 (s, 1H), 5.79-5.61 (m, 1H), 5.18 (s, 1H), 5.10-4.97 (m, 2H), 3.62-3.49 (m, 1H), 2.81-2.63 (m, 2H), 2.61-2.42 (m, 2H), 1.54 (s, 3H), 1.30 (s, 3H) ppm.

^{13}C NMR (151 MHz, $CDCl_3$) δ 169.77, 161.00, 140.49, 138.21, 137.35, 135.09, 124.46, 124.01, 123.05, 121.58, 121.32, 117.64, 106.45, 94.76, 39.49, 38.37, 35.30, 25.28, 24.18 ppm.

HRMS (ESI) m/z $[M+H]^+$: calcd. 329.1206, found. 329.1208.

IR (film): ν_{max} (cm^{-1}) 3075, 2997, 1725, 1632, 1390, 1252, 1203, 1015, 838, 763, 735.

Optical rotation: $[\alpha]_D^{25}$ = -5.51 (*c* = 1.00, $CHCl_3$).

HPLC: DAICEL CHIRALPAK OX-3, hexane/*i*-PrOH = 47/3, flow rate: 0.5 mL/min, λ = 254 nm, t_R(minor) = 30.8 min, t_R(major) = 34.0 min, ee = 91 %

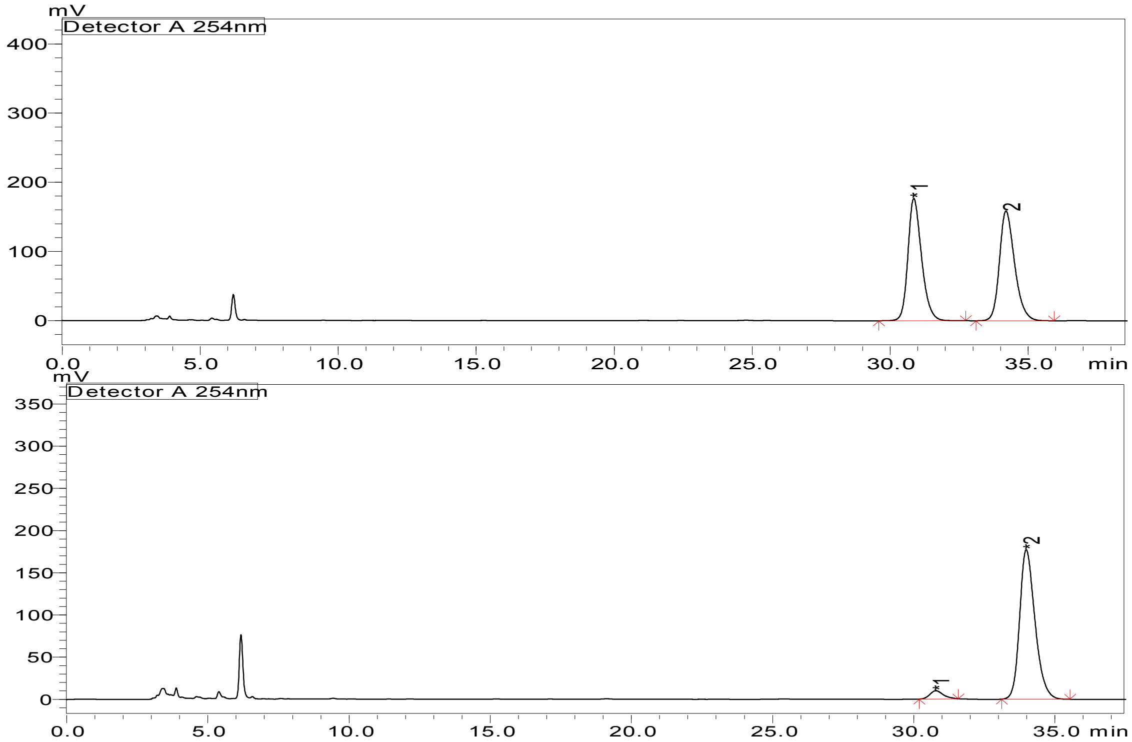

Peak#	Ret. Time	Area%
1	30.858	50.055
2	34.198	49.945

Peak#	Ret. Time	Area%
1	30.785	4.708
2	33.982	95.292

Supplementary Figure 34. HPLC chromatogram for compound **5t**

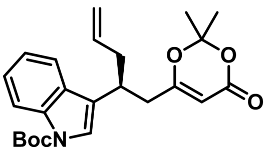

5u

(*S*)-tert-butyl-3-(1-(2,2-dimethyl-4-oxo-4*H*-1,3-dioxin-6-yl)pent-4-en-2-yl)-1*H*-indole-1-carboxylate (**5u**)

According to GPE, 33 mg, pale yellow oil, 80% yield, 93% ee.

^{1}H NMR (400 MHz, CDCl$_3$) δ 8.12 (s, 1H), 7.54 (d, *J* = 7.8 Hz, 1H), 7.40-7.28 (m, 2H), 7.26-7.20 (m, 1H), 5.80-5.64 (m, 1H), 5.15 (s, 1H), 5.11-5.00 (m, 2H), 3.37-3.26 (m, 1H), 2.74-2.60 (m, 2H), 2.60-2.43 (m, 2H), 1.68 (s, 9H), 1.61 (s, 3H), 1.48 (s, 3H) ppm.

^{13}C NMR (151 MHz, CDCl$_3$) δ 169.89, 161.05, 149.63, 135.37, 129.49, 124.54, 122.46, 122.42, 121.85, 118.94, 117.42, 115.49, 106.41, 94.67, 83.81, 39.22, 38.34, 33.35, 28.19, 25.19, 24.69 ppm.

HRMS (ESI) m/z [M+NH$_4$]$^+$: calcd. 429.2384, found. 429.2385.

IR (film): v_{max} (cm^{-1}) 2979, 2931, 1731, 1634, 1374, 1254, 1204,1015, 855, 762.

Optical rotation: $[\alpha]_D^{25}$ = 21.26 (*c* = 1.00, CHCl$_3$).

HPLC: DAICEL CHIRALPAK AD-H, hexane/*i*-PrOH = 47/3, flow rate: 0.5 mL/min, λ = 254 nm, t_R(major) = 12.3 min, t_R(minor) = 13.3 min, ee = 93 %

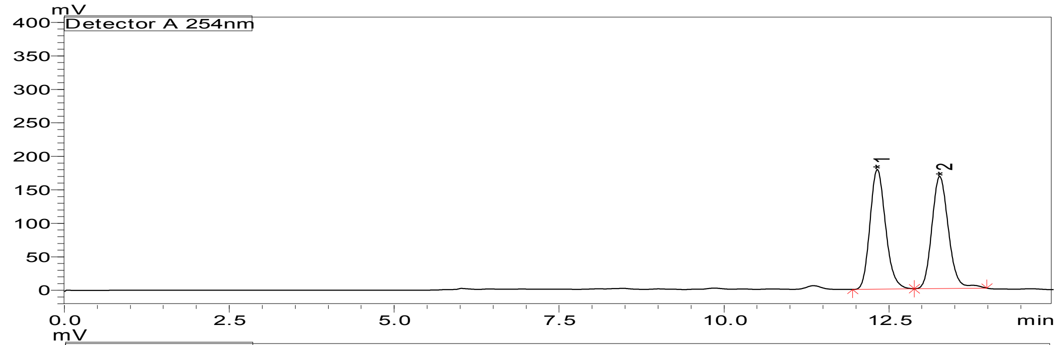

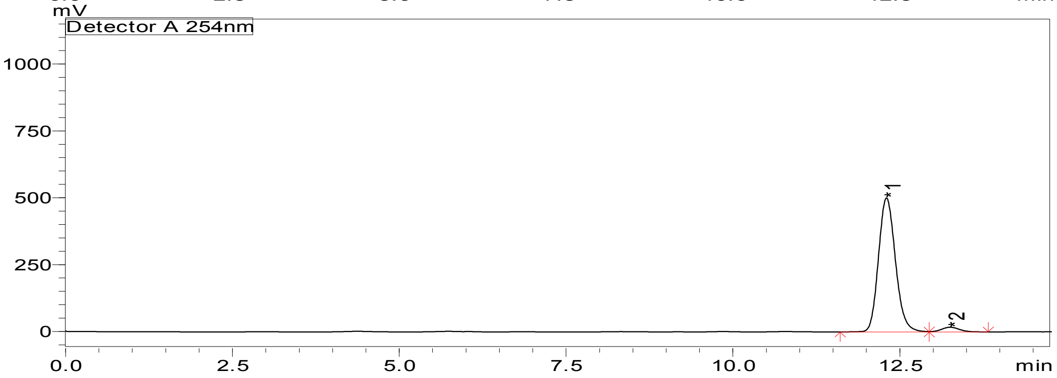

Peak#	Ret. Time	Area%
1	12.323	50.051
2	13.269	49.949

Peak#	Ret. Time	Area%
1	12.301	96.419
2	13.255	3.581

Supplementary Figure 35. HPLC chromatogram for compound **5u**

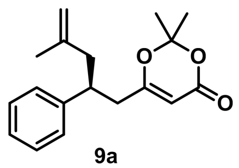

9a

(*S*)-2,2-dimethyl-6-(4-methyl-2-phenylpent-4-en-1-yl)-4*H*-1,3-dioxin-4-one (**9a**)
According to GPE, 18 mg, colourless oil, 63% yield, 95% ee.

^{1}H NMR (400 MHz, $CDCl_3$) δ 7.31-7.26 (m, 2H), 7.23-7.17 (m, 1H), 7.14 (d, *J* = 7.1 Hz, 2H), 5.06 (s, 1H), 4.74 (s, 1H), 4.63 (s, 1H), 3.17-3.01 (m, 1H), 2.71-2.55 (m, 1H), 2.48-2.39 (m, 1H), 2.33 (d, *J* = 7.6 Hz, 2H), 1.68 (s, 3H), 1.55 (s, 3H), 1.44 (s, 3H) ppm.

^{13}C NMR (101 MHz, $CDCl_3$) δ 170.15, 161.11, 142.80, 142.59, 128.47, 127.34, 126.80, 113.19, 106.33, 94.58, 45.50, 40.93, 39.55, 25.29, 24.54, 22.18 ppm.

HRMS (ESI) m/z [M+H]$^{+}$: calcd. 287.1642, found. 287.1641.

IR (film): ν_{max} (cm^{-1}) 3072, 2998, 1728, 1631, 1390, 1251, 1204, 1014, 760.

Optical rotation: $[\alpha]_D^{25}$ = 69.71 (*c* = 1.00, $CHCl_3$).

HPLC: DAICEL CHIRALPAK IF-3, hexane/*i*-PrOH = 24/1, flow rate: 0.5 mL/min, λ = 254 nm, t_R(major) = 27.2 min, t_R(minor) = 28.7 min, ee = 95%.

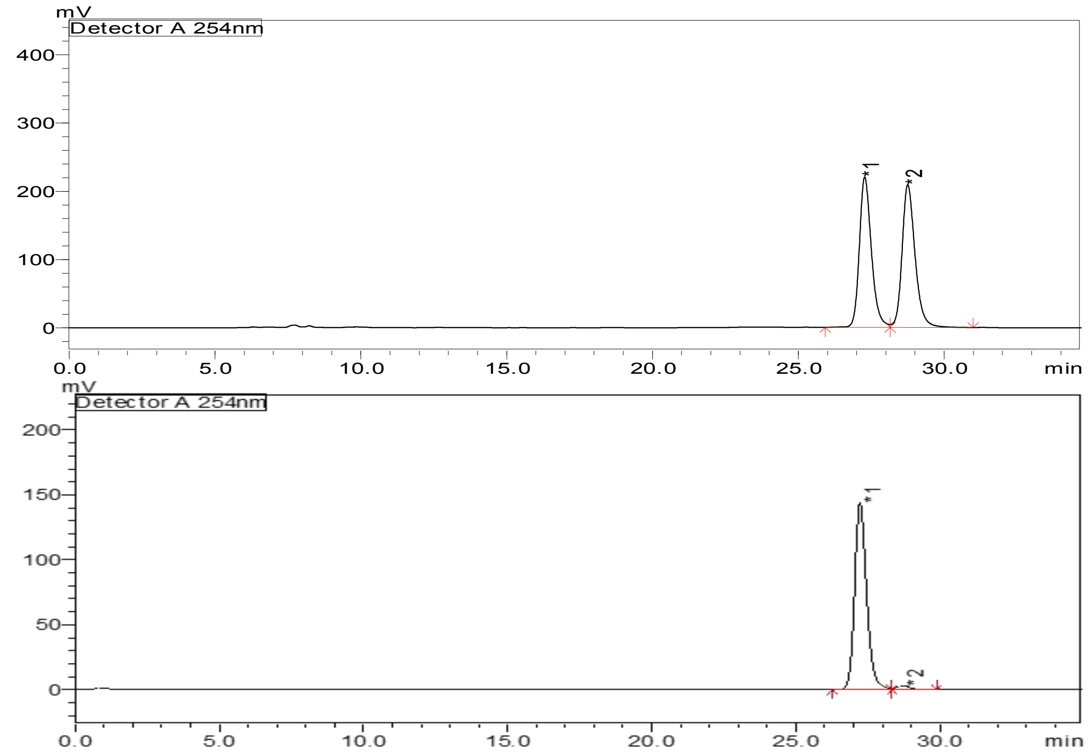

Peak#	Ret. Time	Area%
1	27.276	49.361
2	28.751	50.639

Peak#	Ret. Time	Area%
1	27.227	97.592
2	28.739	2.408

Supplementary Figure 36. HPLC chromatogram for compound **9a**

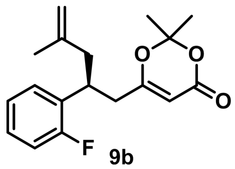

9b

(S)-6-(2-(2-fluorophenyl)-4-methylpent-4-en-1-yl)-2,2-dimethyl-4H-1,3-dioxin-4-one (**9b**)

According to GPE, 20 mg, colourless oil, 63% yield, 93% ee.

^{1}H NMR (400 MHz, CDCl$_3$) δ 7.23-7.11 (m, 2H), 7.11-7.04 (m, 1H), 7.03-6.95 (m, 1H), 5.09 (s, 1H), 4.73 (s, 1H), 4.62 (s, 1H), 3.53-3.41 (m, 1H), 2.70-2.61 (m, 1H), 2.59-2.48 (m, 1H), 2.37 (d, J = 7.6 Hz, 2H), 1.71 (s, 3H), 1.57 (s, 3H), 1.42 (s, 3H) ppm.

^{13}C NMR (151 MHz, CDCl$_3$) δ 169.85, 161.11, 160.88 (d, J = 244.4 Hz), 142.40, 129.37 (d, J = 13.7 Hz), 128.80 (d, J = 4.8 Hz), 128.34 (d, J = 8.1 Hz), 124.18, 115.66 (d, J = 23.4 Hz), 113.24, 106.43, 94.47, 44.17, 38.39, 34.64, 25.44, 24.18, 21.99 ppm.

^{19}F NMR (565 MHz, CDCl$_3$) δ -117.84.

HRMS (ESI) m/z [M+H]$^+$: calcd. 305.1547, found. 305.1548.

IR (film): ν_{max} (cm^{-1}) 2919, 1734, 1636, 1389, 1250, 1203, 1014, 900,804.

Optical rotation: $[\alpha]_D^{25}$ = 44.22 (c = 1.00, CHCl$_3$).

HPLC: DAICEL CHIRALPAK OX-3, hexane/i-PrOH = 9/1, flow rate: 0.5 mL/min, λ = 254 nm, t_R(major) = 28.7 min, t_R(minor) = 27.7 min, ee = 93%.

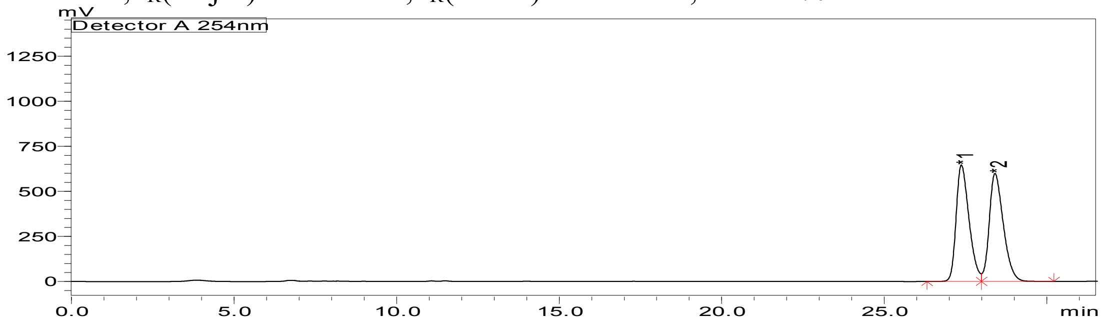

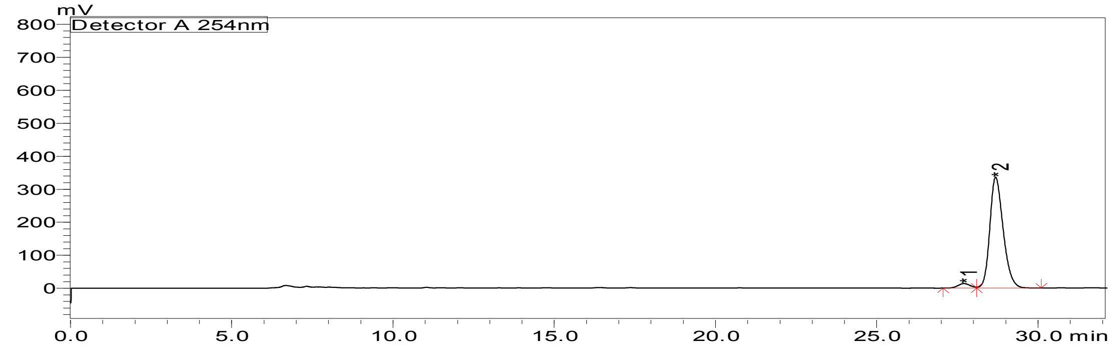

Peak#	Ret. Time	Area%
1	27.369	49.670
2	28.404	50.330

Peak#	Ret. Time	Area%
1	27.701	3.659
2	28.684	96.341

Supplementary Figure 37. HPLC chromatogram for compound **9b**

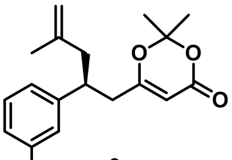

9c

(*S*)-2,2-dimethyl-6-(4-methyl-2-(m-tolyl)pent-4-en-1-yl)-4*H*-1,3-dioxin-4-one (**9c**)
According to GPE, 17 mg, colourless oil, 57% yield, 97% ee.
^{1}H NMR (400 MHz, $CDCl_3$) δ 7.20-7.13 (m, 1H), 7.01 (d, *J* = 7.4 Hz, 1H), 6.97-6.89 (m, 2H), 5.05 (s, 1H), 4.75 (s, 1H), 4.65 (s, 1H), 3.12-3.01 (m, 1H), 2.67-2.57 (m, 1H), 2.46-2.37 (m, 1H), 2.35-2.28 (m, 5H), 1.68 (s, 3H), 1.56 (s, 3H), 1.45 (s, 3H) ppm.
^{13}C NMR (151 MHz, $CDCl_3$) δ 170.30, 161.15, 142.84, 142.71, 137.95, 128.35, 128.13, 127.53, 124.31, 113.10, 106.32, 94.53, 45.42, 40.80, 39.55, 25.31, 24.56, 22.19, 21.42 ppm.
HRMS (ESI) m/z [M+H]$^+$: calcd. 301.1798, found. 301.1799.
IR (film): ν_{max} (cm^{-1}) 2923, 1730, 1635, 1389, 1250, 1203, 1014, 901, 804.
Optical rotation: $[\alpha]_D^{25}$ = 66.19 (*c* = 1.00, $CHCl_3$).
HPLC: DAICEL CHIRALPAK AY-3, hexane/*i*-PrOH = 99/1, flow rate: 1 mL/min, λ = 254 nm, t_R(major) = 27.2 min, t_R(minor) = 26.1 min, ee = 97%.

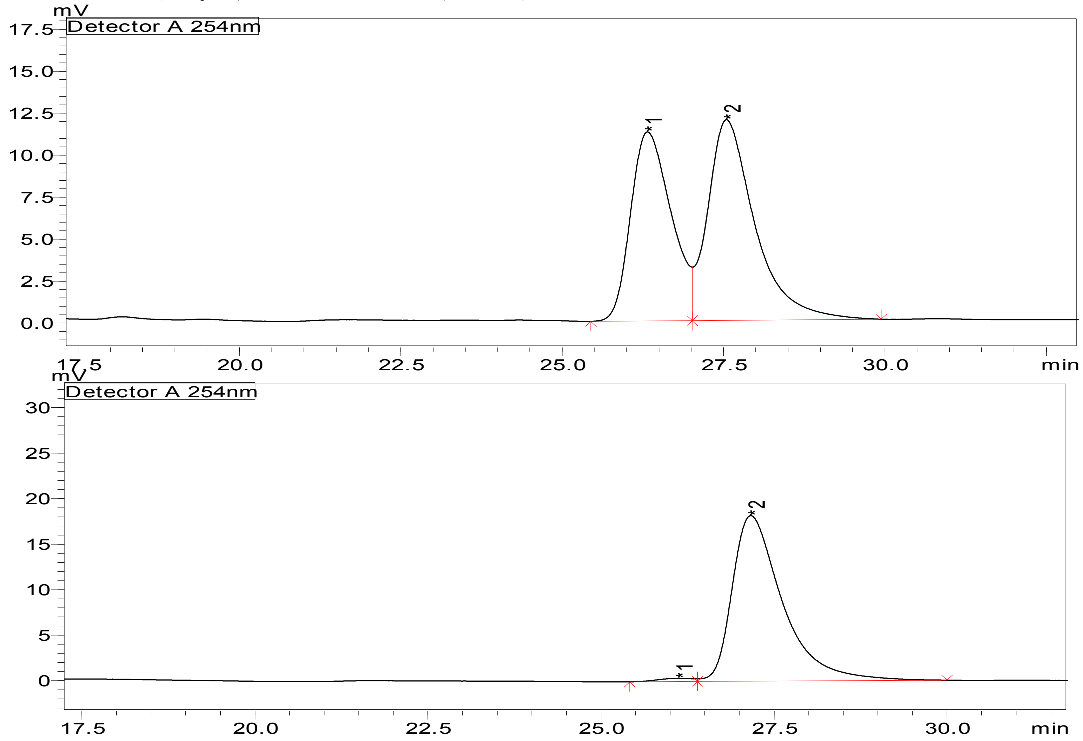

Peak#	Ret. Time	Area%
1	26.323	44.849
2	27.547	55.151

Peak#	Ret. Time	Area%
1	26.117	1.370
2	27.165	98.630

Supplementary Figure 38. HPLC chromatogram for compound **9c**

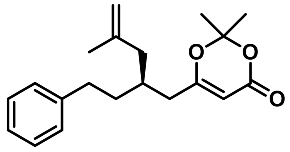

9d

(*S*)-2,2-dimethyl-6-(4-methyl-2-phenethylpent-4-en-1-yl)-4*H*-1,3-dioxin-4-one (**9d**)
According to GPE, 21 mg, colourless oil, 64% yield, 92% ee.
^{1}H NMR (400 MHz, $CDCl_3$) δ 7.33-7.26 (m, 2H), 7.21-7.12 (m, 3H), 5.22 (s, 1H), 4.82 (s, 1H), 4.71 (s, 1H), 2.69-2.58 (m, 2H), 2.30-2.20 (m, 1H), 2.20-2.09 (m, 2H), 2.03-1.85 (m, 2H), 1.67-1.59 (m, 11H) ppm.
^{13}C NMR (151 MHz, $CDCl_3$) δ 171.10, 161.12, 143.17, 141.85, 128.40, 128.32, 125.92, 112.92, 106.26, 94.43, 42.50, 37.87, 35.22, 32.61, 32.49, 25.14, 24.97, 22.03 ppm.
HRMS (ESI) m/z $[M+H]^+$: calcd. 315.1955, found. 315.1955.
IR (film): ν_{max} (cm^{-1}) 3026, 2998, 1732, 1631, 1389, 1253, 1204, 1014, 901, 802.
Optical rotation: $[\alpha]_D^{25}$ = -0.22 (*c* = 1.00, $CHCl_3$).
HPLC: DAICEL CHIRALPAK OD-H, hexane/*i*-PrOH = 49/1, flow rate: 1 mL/min, λ = 254 nm, t_R(minor) = 14.9 min, t_R(major) = 15.9 min, ee = 92%.

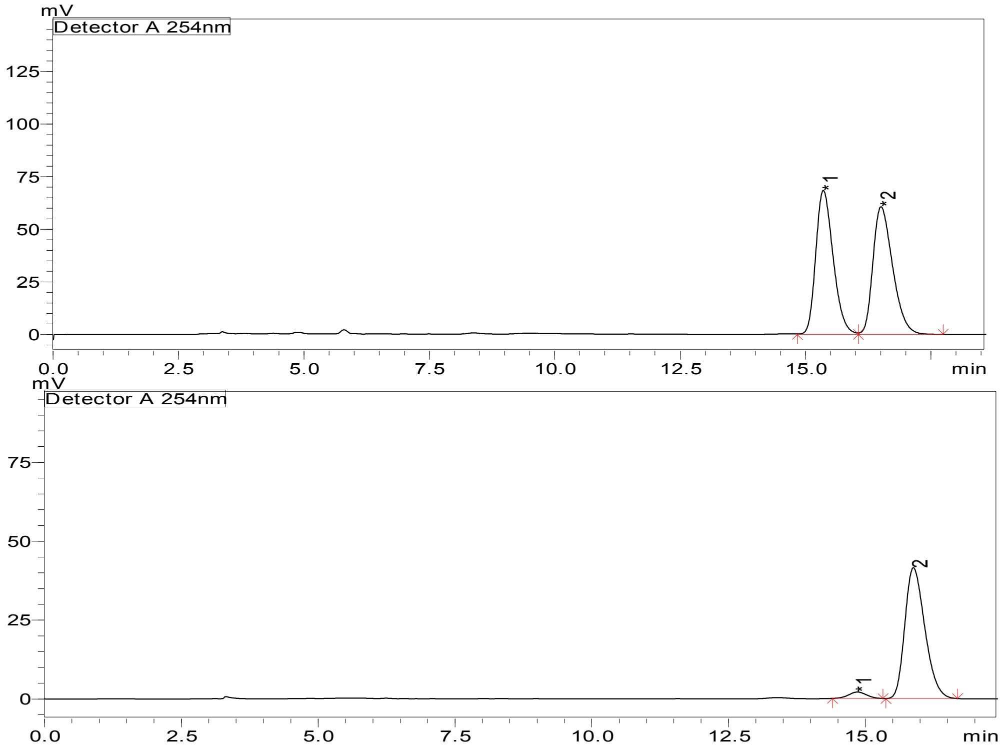

Peak#	Ret. Time	Area%
1	15.354	49.897
2	16.504	50.103

Peak#	Ret. Time	Area%
1	14.856	4.194
2	15.878	95.806

Supplementary Figure 39. HPLC chromatogram for compound **9d**

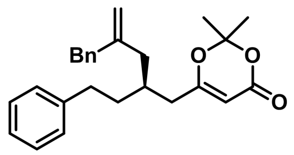

10

(*S*)-6-(4-benzyl-2-phenethylpent-4-en-1-yl)-2,2-dimethyl-4*H*-1,3-dioxin-4-one (**10**)
According to GPE, 30 mg, colourless oil, 77% yield, 93% ee.

^{1}H NMR (400 MHz, CDCl$_3$) δ 7.31-7.25 (m, 4H), 7.24-7.18 (m, 2H), 7.15-7.07 (m, 4H), 5.16 (s, 1H), 4.87 (s, 1H), 4.85 (s, 1H), 3.25 (s, 2H), 2.60-2.51 (m, 2H), 2.26-2.18 (m, 1H), 2.17-2.06 (m, 2H), 1.98-1.85 (m, 2H), 1.62-1.55 (m, 8H) ppm.

^{13}C NMR (151 MHz, CDCl$_3$) δ 170.98, 161.06, 146.23, 141.77, 139.08, 128.87, 128.40, 128.37, 128.34, 126.28, 125.93, 114.25, 106.25, 94.41, 42.49, 40.08, 37.91, 35.18, 32.55, 32.41, 25.09, 24.85 ppm.

HRMS (ESI) m/z [M+H]$^+$: calcd. 391.2268, found. 391.2269.

IR (film): ν_{max} (cm^{-1}) 3061, 3026, 2999, 1729, 1631, 1389, 1252, 1204, 1013, 740, 700.

Optical rotation: $[\alpha]_D^{25}$ = 1.76 (*c* = 1.00, CHCl$_3$).

HPLC: DAICEL CHIRALPAK IBN-3, hexane/*i*-PrOH = 9/1, flow rate: 0.5 mL/min, λ = 254 nm, t_R(major) = 19.8 min, t_R(minor) = 18.3 min, ee = 93%.

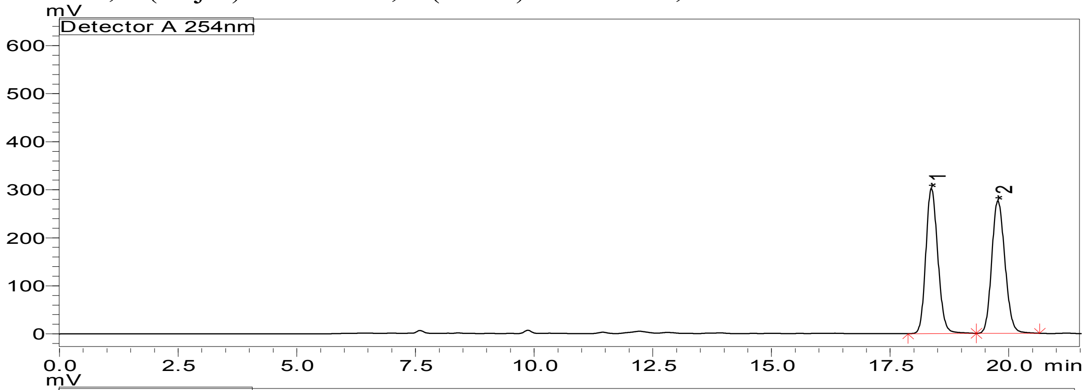

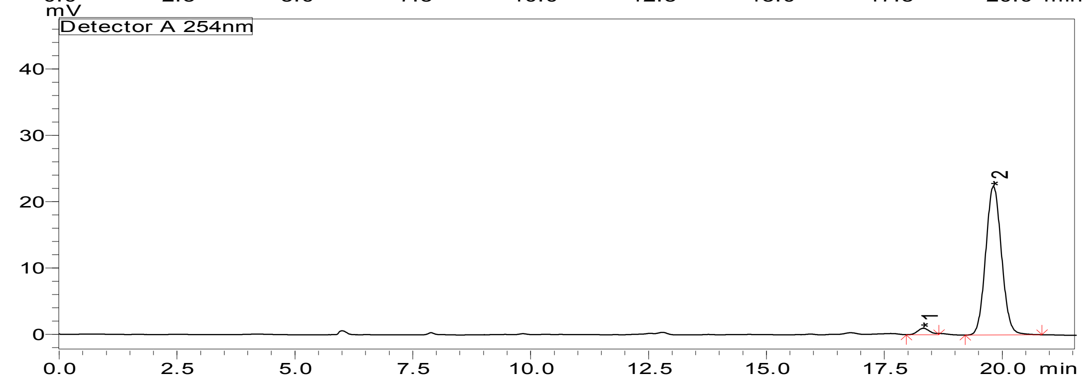

Peak#	Ret. Time	Area%
1	18.367	49.939
2	19.768	50.061

Peak#	Ret. Time	Area%
1	18.328	3.384
2	19.814	96.616

Supplementary Figure 40. HPLC chromatogram for compound **10**

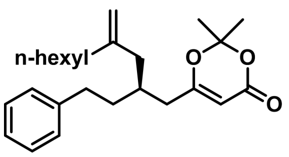

11

(S)-2,2-dimethyl-6-(4-methylene-2-phenethylundecyl)-4H-1,3-dioxin-4-one (**11**)
According to GPE, 26 mg, colourless oil, 68% yield, 92% ee.
^{1}H NMR (400 MHz, CDCl$_3$) δ 7.32-7.26 (m, 2H), 7.21-7.12 (m, 3H), 5.22 (s, 1H), 4.82 (s, 1H), 4.72 (s, 1H), 2.68-2.59 (m, 2H), 2.30-2.21 (m, 1H), 2.20-2.10 (m, 2H), 2.00-1.87 (m, 4H), 1.74-1.62 (m, 7H), 1.43-1.35 (m, 2H), 1.33-1.21 (m, 7H), 0.89 (t, J = 6.8 Hz, 3H) ppm.
^{13}C NMR (151 MHz, CDCl$_3$) δ 171.18, 161.12, 147.25, 141.85, 128.39, 128.32, 125.91, 111.61, 106.25, 94.41, 40.79, 37.97, 35.45, 35.28, 32.65, 32.60, 31.70, 29.03, 27.63, 25.18, 24.91, 22.61, 14.07 ppm.
HRMS (ESI) m/z [M+H]$^+$: calcd. 385.2737, found. 385.2737.
IR (film): ν_{max} (cm^{-1}) 3026, 2998, 1735, 1632, 1389, 1252, 1204, 1014, 746.
Optical rotation: $[\alpha]_D^{25}$ = 1.04 (c = 1.00, CHCl$_3$).
HPLC: DAICEL CHIRALPAK OD-H, hexane/i-PrOH = 49/1, flow rate: 1.0 mL/min, λ = 254 nm, t_R(minor) = 10.4 min, t_R(major) = 11.5 min, ee = 91%.

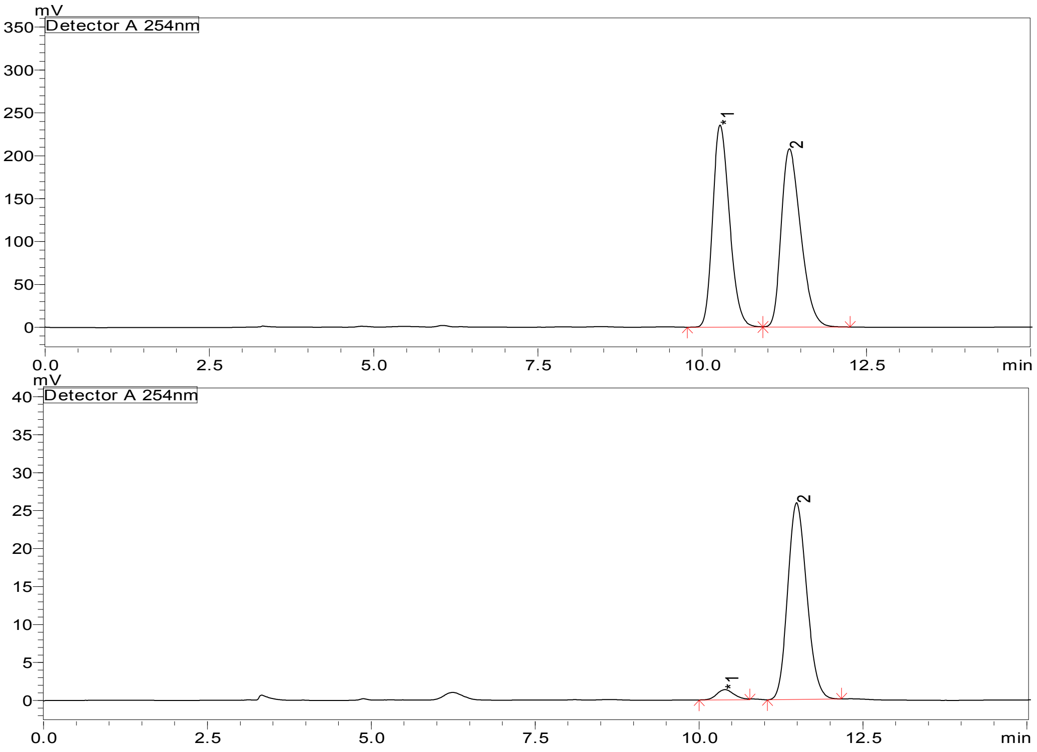

Peak#	Ret. Time	Area%
1	10.272	49.405
2	11.326	50.595

Peak#	Ret. Time	Area%
1	10.394	4.570
2	11.488	95.430

Supplementary Figure 41. HPLC chromatogram for compound **11**

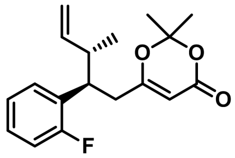

13

6-((2*R*,3*S*)-2-(2-fluorophenyl)-3-methylpent-4-en-1-yl)-2,2-dimethyl-4*H*-1,3-dioxin-4-one (**13**)

According to GPF, 17 mg, colourless oil, 59% yield, 90% ee, 3/1 dr.

^{1}H NMR (400 MHz, CDCl$_3$) δ 7.23-7.14 (m, 1H), 7.13-7.04 (m, 2H), 7.03-6.94 (m, 1H), 5.76-5.53 (m, 1H), 5.10-5.07 (m, 1H), 5.04-4.86 (m, 2H), 3.38-3.03 (m, 1H), 2.82-2.33 (m, 3H), 1.55-1.52 (m, 3H), 1.32-1.28 (m, 3H), 1.06-0.81 (m, 3H) ppm.

^{13}C NMR (101 MHz, CDCl$_3$) δ 170.33, 169.99, 161.01 (d, *J* = 244.6 Hz), 161.16, 161.14, 142.07, 140.44, 129.57 (d, *J* = 4.7 Hz), 129.28, 128.28, 127.24, 124.14, 123.73 (d, *J* = 3.4 Hz), 115.6, 115.28, 106.40, 106.33, 94.66, 94.29, 43.96, 42.67, 41.36, 40.05, 37.16, 35.90, 27.92, 25.66, 23.65, 23.55, 18.82, 17.64 ppm.

^{19}F NMR (376 MHz, CDCl$_3$) δ -95.16, -95.59.

HRMS (ESI) m/z [M+H]$^+$: calcd. 305.1547, found. 305.1548.

IR (film): ν_{max} (cm^{-1}) 3004, 2967, 1730, 1390, 1253, 1204, 759.

Optical rotation: $[\alpha]_D^{25}$ = 32.67 (*c* = 0.50, CHCl$_3$).

HPLC: DAICEL CHIRALPAK AD-H, hexane/*i*-PrOH = 9/1, flow rate: 0.5 mL/min, λ = 254 nm, t_R(minor) = 10.6 min, t_R(major) = 11.7 min, ee = 90%.

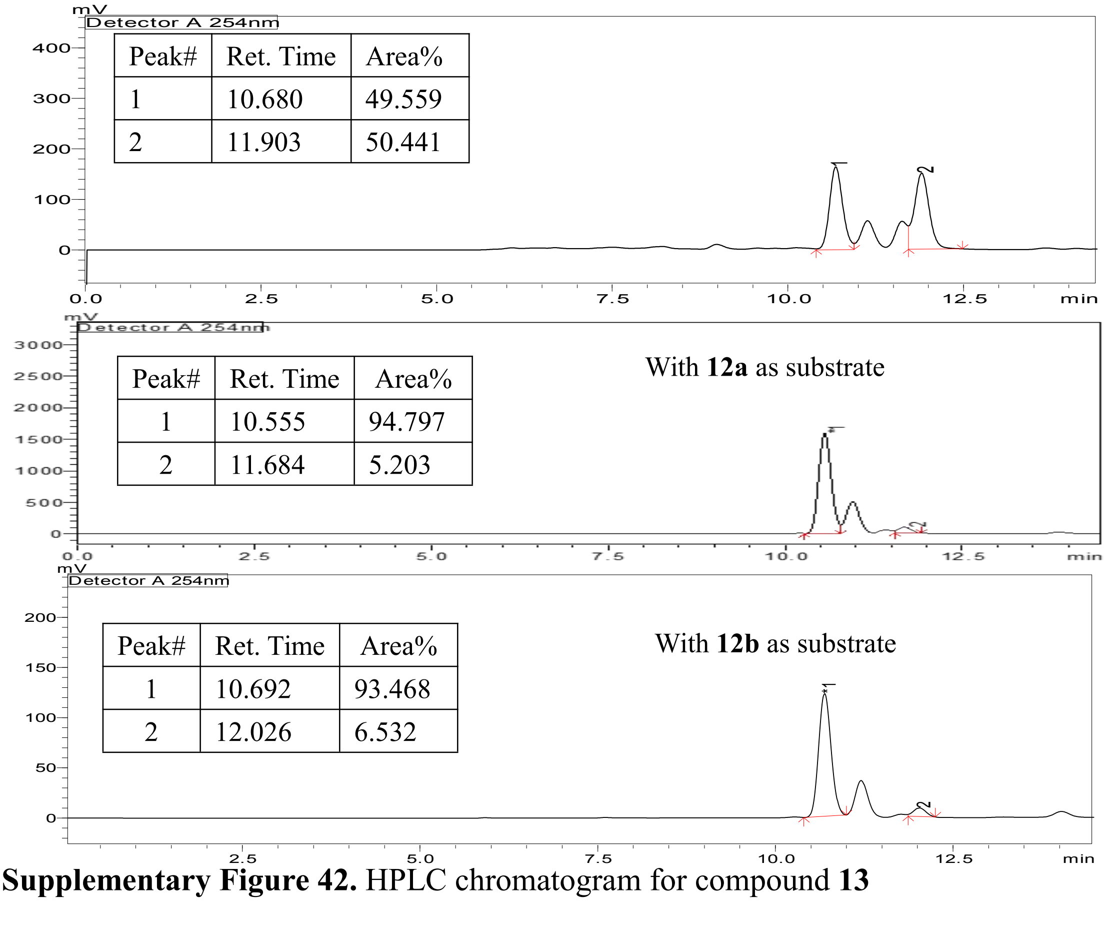

Peak#	Ret. Time	Area%
1	10.680	49.559
2	11.903	50.441

With **12a** as substrate

Peak#	Ret. Time	Area%
1	10.555	94.797
2	11.684	5.203

With **12b** as substrate

Peak#	Ret. Time	Area%
1	10.692	93.468
2	12.026	6.532

Supplementary Figure 42. HPLC chromatogram for compound **13**

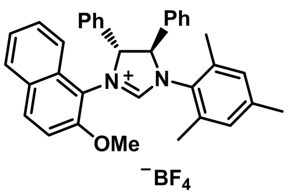

(4*R*,5*R*)-1-mesityl-3-(2-methoxynaphthalen-1-yl)-4,5-diphenyl-4,5-dihydro-1*H*-imidazol-3-ium tetrafluoroborate (NHC-L6•HBF_4)

Pale yellow solid.

^{1}H NMR (400 MHz, $CDCl_3$) δ 8.24 (s, 1H), 7.90 - 7.80 (m, 1H), 7.79 - 7.69 (m, 1H), 7.61 (s, 3H), 7.47 - 7.11 (m, 10H), 6.91 (s, 1H), 6.69 (s, 2H), 6.11 (d, J = 10.7 Hz, 1H), 5.95 (d, J = 10.7 Hz, 1H), 4.25 (s, 3H), 2.72 (s, 3H), 2.72 (s, 3H), 2.15 (s, 3H), 1.97 (s, 3H) ppm.

^{13}C NMR (101 MHz, $CDCl_3$) δ 159.62, 153.10, 140.13, 135.52, 134.41, 133.02, 132.60, 131.86, 130.55, 130.13, 129.91, 129.22, 128.84, 128.57, 128.37, 128.11, 127.77, 124.43, 120.69, 115.50, 112.41, 74.18, 56.87, 53.48, 20.74, 18.97, 17.71. ppm.

^{19}F NMR (376 MHz, $CDCl_3$) δ -152.62.

HRMS (ESI) m/z [M-BF_4]$^+$: calcd. 497.2587, found. 497.2587.

IR (film): ν_{max} (cm^{-1}) 2918, 1616, 1455, 1273, 1059, 757.

Optical rotation: $[\alpha]_D^{25}$ = 285.69 (c = 1.0, $CHCl_3$).

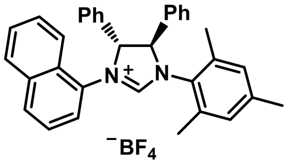

(4*R*,5*R*)-1-mesityl-3-(naphthalen-1-yl)-4,5-diphenyl-4,5-dihydro-1*H*-imidazol-3-ium tetrafluoroborate (NHC-L7•HBF_4)

Pale yellow solid.

^{1}H NMR (400 MHz, $CDCl_3$) δ 8.20 (s, 1H), 8.04 (d, J = 8.5 Hz, 1H), 7.88 (d, J = 7.4 Hz, 1H), 7.86 - 7.78 (m, 2H), 7.71 (d, J = 8.2 Hz, 1H), 7.55 - 7.43 (m, 5H), 7.42 - 7.32 (m, 3H), 7.30 - 7.17 (m, 4H), 6.84 (s, 1H), 6.68 (s, 1H), 6.40 (d, J = 11.2 Hz, 1H), 5.84 (d, J = 11.2 Hz, 1H), 2.67 (s, 3H), 2.10 (s, 3H), 1.95 (s, 3H) ppm.

^{13}C NMR (101 MHz, $CDCl_3$) δ 157.64, 140.10, 135.48, 134.71, 133.84, 133.33, 130.76, 130.49, 130.29, 130.17, 129.90, 129.70, 129.32, 129.27, 129.20, 128.95, 128.85, 128.57, 128.43, 128.26, 127.95, 126.85, 126.41, 125.38, 119.89, 114.09, 75.57, 74.06, 20.53, 18.69, 18.02 ppm.

^{19}F NMR (376 MHz, $CDCl_3$) δ -150.91.

HRMS (ESI) m/z [M-BF_4]$^+$: calcd. 467.2482, found. 467.2482.

IR (film): ν_{max} (cm^{-1}) 3062, 2926, 1616, 1456, 1269, 1056, 758.

Optical rotation: $[\alpha]_D^{25}$ = 322.21 (c = 1.0, $CHCl_3$).

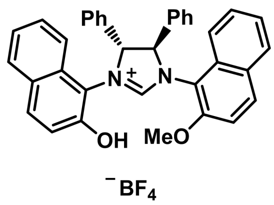

(4*R*,5*R*)-3-(2-hydroxynaphthalen-1-yl)-1-(2-methoxynaphthalen-1-yl)-4,5-diphenyl-4,5-dihydro-1*H*-imidazol-3-ium tetrafluoroborate (NHC-L8•HBF_4)

Pale brown solid.

^{1}H NMR (400 MHz, $CDCl_3$) δ 7.91 - 7.77 (m, 2H), 7.76 - 7.43 (m, 14H), 7.23 (s, 8H), 6.19 (d, J = 25.8 Hz, 2H), 4.36 (s, 3H). ppm.

^{13}C NMR (101 MHz, $CDCl_3$) δ 165.72, 162.95, 159.51, 157.31, 153.02, 152.85, 152.32, 151.48, 151.12, 133.77, 133.30, 133.04, 132.58, 131.98, 130.32, 130.07, 129.11, 128.75, 128.62, 128.29, 128.01, 127.83, 127.54, 126.55, 124.58, 124.01, 123.70, 123.50, 122.21, 119.77, 118.41, 114.56, 113.58, 112.41, 74.75, 56.95. ppm.

HRMS (ESI) m/z [M+H]$^+$: calcd. 521.2224, found. 521.2224.

IR (film): ν_{max} (cm^{-1}) 3349, 3066, 1618, 1512, 1276, 1061, 754, 733.

Optical rotation: $[\alpha]_D^{25}$ = 344.83 (c = 1.0, $CHCl_3$)

Supplementary Note 1. Product Derivatizations

Hydroborylation[2],[3]

A dried 25 mL Schlenk tube equipped with a magnetic stirring bar was charged with $[Ir(COD)Cl]_2$ (1.7 mg, 0.0025 mmol, 0.05 equiv) and bis(diphenylphosphanyl)methane (1.9 mg, 0.005 mmol, 0.1 equiv) in a glove box under Ar atmosphere. Anhydrous DCM (1 mL), **5d** (14.5 mg, 0.05 mmol, 1.0 equiv) and HBpin (12.8 mg, 0.1 mmol, 2.0 equiv) were added sequentially. The mixture was stirred for 24 hours at room temperature. Then, the crude mixture was purified by flash column chromatography (petroleum ether/ethyl acetate = 7/1) to afford the desired product **14**.

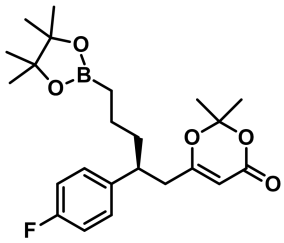

14

(*S*)-6-(2-(4-fluorophenyl)-5-(4,4,5,5-tetramethyl-1,3,2-dioxaborolan-2-yl)pentyl)-2,2-dimethyl-4*H*-1,3-dioxin-4-one (**14**)
13 mg, colourless oil, 62% yield.
^{1}H NMR (400 MHz, $CDCl_3$) δ 7.13-7.05 (m, 2H), 7.02-6.93 (m, 2H), 5.06 (s, 1H), 2.96-2.82 (m, 1H), 2.62-2.50 (m, 1H), 2.47-2.36 (m, 1H), 1.69-1.60 (m, 2H), 1.57 (s, 3H), 1.47 (s, 3H), 1.35-1.14 (m, 14H), 0.79-0.63 (m, 2H) ppm.
^{13}C NMR (151 MHz, $CDCl_3$) δ 169.97, 161.53 (d, J = 244.5 Hz), 161.07, 138.93, 128.82 (d, J = 8.0 Hz), 115.28 (d, J = 21.5 Hz), 106.32, 94.51, 82.98, 42.06, 40.71, 39.43, 25.22, 24.78, 24.71, 21.56 ppm.
^{19}F NMR (376 MHz, $CDCl_3$) δ -116.54.
^{11}B NMR (193 MHz, $CDCl_3$) δ 33.55.
HRMS (ESI) m/z $[M+H]^+$: calcd. 418.2436, found. 418.2438.
IR (film): ν_{max} (cm^{-1}) 2978, 2931, 1731, 1633, 1379, 1253, 1014, 839.
Optical rotation: $[\alpha]_D^{25}$ = 12.01 (c = 0.50, $CHCl_3$).

Metathesis

A 25mL flame dried Schlenk tube was charged with **5d** (14.5 mg, 0.05 mmol, 1.0 equiv), 4-methylstyrene (11.8 mg, 0.1 mmol, 2 equiv) and Hoveyda-Grubbs II catalyst (4.2 mg, 0.005 mmol, 0.1 equiv). Anhydrous CH_2Cl_2 (1 mL) was added by a syringe under N_2 atmosphere. The reaction mixture was stirred at 40 °C for 4 h. The crude reaction mixture was direct purified by flash column chromatography (petroleum ether/ethyl acetate = 7/1) to afford the desired product **15**.

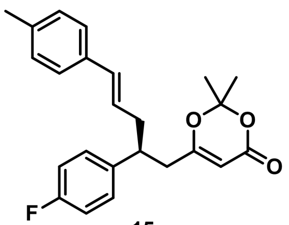

15

(*S*,*E*)-6-(2-(4-fluorophenyl)-5-(p-tolyl)pent-4-en-1-yl)-2,2-dimethyl-4*H*-1,3-dioxin-4-one (**15**)
13.5 mg, colourless oil, 71% yield.
^{1}H NMR (400 MHz, $CDCl_3$) δ 7.20-7.05 (m, 6H), 7.03-6.95 (m, 2H), 6.32 (d, *J* = 15.8 Hz, 1H), 6.05-5.89 (m, 1H), 5.09 (s, 1H), 3.14-3.02 (m, 1H), 2.75-2.64 (m, 1H), 2.55-2.43 (m, 3H), 2.32 (s, 3H), 1.57 (s, 3H), 1.45 (s, 3H) ppm.
^{13}C NMR (101 MHz, $CDCl_3$) δ 169.75, 161.62 (d, *J* = 245.5 Hz), 161.02, 138.15, 137.16, 134.25, 132.44, 129.21, 128.84 (d, *J* = 7.8 Hz), 125.91, 125.66, 115.42 (d, *J* = 21.2 Hz), 106.41, 94.72, 42.39, 40.56, 39.41, 29.69, 25.27, 24.59, 21.15 ppm.
^{19}F NMR (376 MHz, $CDCl_3$) δ -115.79.
HRMS (ESI) m/z $[M+H]^+$: calcd. 381.1860, found. 381.1862.
IR (film): v_{max} (cm^{-1}) 2998, 2958, 1729, 1390, 1251, 1015, 834, 806.
Optical rotation: $[\alpha]_D^{25}$ = 1.65 (*c* = 0.50, $CHCl_3$).

Alcoholysis[4]

To a 25mL flame dried Schlenk tube charged with **5d** (29 mg, 0.1 mmol, 1.0 equiv) and K_2CO_3 (41.5 mg, 0.3 mmol, 3.0 equiv) was added anhydrous MeOH (1 ml) by a syringe under N_2 atmosphere. The reaction mixture was stirred at room temperature overnight. The crude reaction mixture was direct purified by flash column chromatography (petroleum ether/ethyl acetate = 10/1) to afford the desired product **16**.

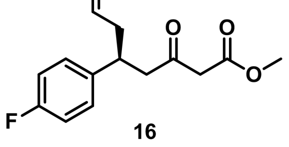

16

(*S*)-methyl 5-(4-fluorophenyl)-3-oxooct-7-enoate (**16**)
18.4 mg, pale yellow oil, 75% yield.
^{1}H NMR (400 MHz, $CDCl_3$) δ 7.19-7.09 (m, 2H), 7.03-6.93 (m, 2H), 5.68-5.56 (m, 1H), 5.05-4.92 (m, 2H), 3.68 (s, 3H), 3.36-3.21 (m, 3H), 2.93-2.76 (m, 2H), 2.38-2.29 (m, 2H) ppm.
^{13}C NMR (151 MHz, $CDCl_3$) δ 201.15, 167.31, 161.46, 139.26, 135.68, 128.85, 117.17, 115.28, 52.33, 49.60, 48.76, 40.69, 39.59 ppm.
^{19}F NMR (376 MHz, $CDCl_3$) δ -116.79.
HRMS (ESI) m/z $[M+NH_4]^+$: calcd. 282.1500, found. 282.1500.

IR (film): v_{max} (cm^{-1}) 2959, 2925, 1718, 1654, 1437, 1221, 1015, 833.
Optical rotation: $[\alpha]_D^{25}$ = 18.88 (c = 1.00, $CHCl_3$).

Dehydration

Step one: A 25mL flame dried Schlenk tube filled with N_2 was charged with **16** (80 mg, 0.27 mmol, 1.0 equiv) and MeOH (4 ml). The mixture was cooled down to 0 °C and $NaBH_4$ (20.8 mg, 0.55 mmol, 2.0 equiv) was added in one portion. The reaction mixture was stirred at 0 °C for 1h. The major part of solvent was removed under reduced pressure. Then H_2O (5 ml) was added. The crude reaction mixture was extracted with ethyl acetate (5 ml x 3) and dried by anhydrous Na_2SO_4. After removing of the solvent, the product (β-hydroxyl-ester) was directed used without further purification.
Step two: A 25mL flame dried Schlenk tube filled with N_2 was charged with β-hydroxyl-ester, DCM (3 ml) and triethylamine (210.5 mg, 2.16 mmol, 8 equiv) at 0 °C. To the mixture was added MsCl (75.6 mg, 0.66 mmol, 3.0 equiv) dropwise. The reaction mixture was warmed to room temperature and stirred at room temperature for 8 h. The crude reaction mixture was direct purified by flash column chromatography (petroleum ether/ethyl acetate = 10/1) to afford the desired product **17**.

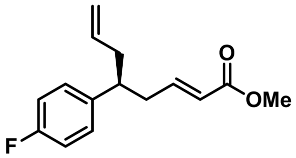

17

(S,E)-methyl 5-(4-fluorophenyl)octa-2,7-dienoate (**17**)
40 mg, colourless oil, 60% yield (two steps).
^{1}H NMR (400 MHz, $CDCl_3$) δ 7.14-7.06 (m, 2H), 7.02-6.94 (m, 2H), 6.85-6.74 (m, 1H), 5.75 (d, J = 15.6 Hz, 1H), 5.69-5.55 (m, 1H), 5.02-4.93 (m, 2H), 3.69 (s, 3H), 2.85-2.74 (m, 1H), 2.62-2.51 (m, 1H), 2.50-2.29 (m, 3H) ppm.
^{13}C NMR (101 MHz, $CDCl_3$) δ 166.74, 161.43 (d, J = 244.2 Hz), 146.92, 139.16, 135.82, 128.87 (d, J = 7.8 Hz), 122.53, 116.87, 115.25 (d, J = 21.2 Hz), 51.42, 44.11, 40.55, 38.56 ppm.
^{19}F NMR (376 MHz, $CDCl_3$) δ -116.94.
HRMS (ESI) m/z $[M+NH_4]^+$: calcd. 266.1551, found. 266.1552.
IR (film): v_{max} (cm^{-1}) 2951, 2925, 1724, 1456, 1270, 1223, 1015, 831.
Optical rotation: $[\alpha]_D^{25}$ = 15.52 (c = 0.50, $CHCl_3$).

Pyrazole synthesis

To a 25mL flame dried Schlenk tube charged with **15** (13 mg, 0.05 mmol, 1.0 equiv) and NH_2NH_2•HCl (6.4 mg, 0.10 mmol, 2.0 equiv) was added anhydrous MeOH (1 ml) by a syringe under N_2 atmosphere. The reaction mixture was refluxed for 8 h. After cooling down to room temperature, the crude reaction mixture was direct purified by flash column chromatography (petroleum ether/ethyl acetate = 1/1) to afford the desired product **18**.

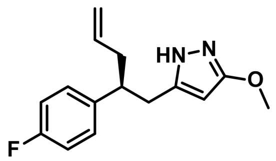

18

(*S*)-5-(2-(4-fluorophenyl)pent-4-en-1-yl)-3-methoxy-1H-pyrazole (**18**)
6.7 mg, colourless oil, 51% yield.
^{1}H NMR (400 MHz, $CDCl_3$) δ 7.13-7.05 (m, 2H), 7.03-6.93 (m, 2H), 5.70-5.57 (m, 1H), 5.35 (s, 1H), 5.05-4.92 (m, 2H), 3.82 (s, 3H), 2.97-2.86 (m, 2H), 2.84-2.73 (m, 1H), 2.47-2.33 (m, 2H) ppm.
^{13}C NMR (101 MHz, $CDCl_3$) δ 161.59 (d, *J* = 245.0 Hz), 143.43, 139.11 (d, *J* = 3.2 Hz), 135.75, 128.90 (d, *J* = 7.8 Hz), 117.11, 115.45 (d, *J* = 21.1 Hz), 89.76, 55.97, 44.79, 40.36, 32.89 ppm.
^{19}F NMR (376 MHz, $CDCl_3$) δ -116.15.
HRMS (ESI) m/z $[M+H]^+$: calcd. 261.1398, found. 261.1399.
IR (film): ν_{max} (cm^{-1}) 3192, 2924, 1578, 1460, 1410, 1039, 832.
Optical rotation: $[\alpha]_D^{25}$ = 9.36 (*c* = 0.50, $CHCl_3$).

Supplementary Note 2. Determination of the Absolute Configurations of 5a and 13.

The absolute configuration of **5a** is determined by converting **5a** to a literature compound **5a′** as below[5].

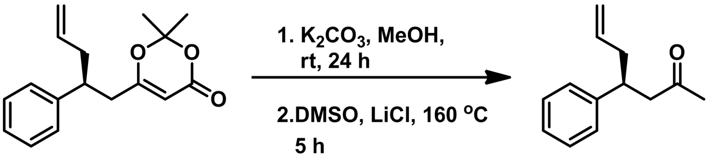

5a 5a'

The literature reported data of **5a′**: **Optical rotation:** $[\alpha]_D^{20}$ = +7.9 (*c* = 1.0, $CHCl_3$). **HPLC:** CHIRAL OJ-H column, hexane/*i*-PrOH = 95/5, flow rate: 1 mL/min, t_R (*S*)-(major) = 9.1 min, t_R (*R*)-(minor) = 10.4 min, ee = 93%.

Our data of **5a′: Optical rotation:** $[\alpha]_D^{20}$ = +34.2 (*c* = 1.0, $CHCl_3$). **HPLC:**CHIRAL OJ-H column, hexane/*i*-PrOH = 95/5, flow rate: 1 mL/min, t_R (*S*)-(major) = 8.2 min, t_R (*R*)-(minor) = 9.2min, ee = 94%.

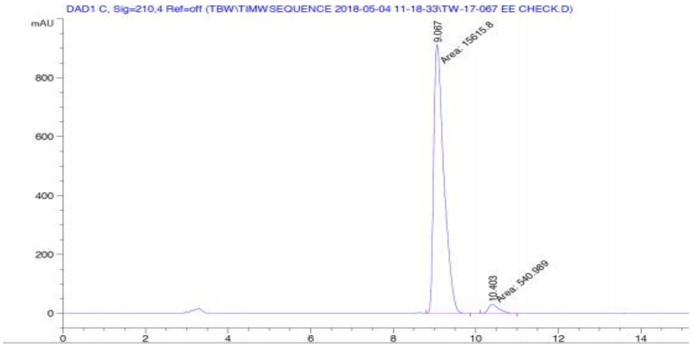

Peak#	Ret. Time	Area%
1	9.067	96.6516
2	10.403	3.3484

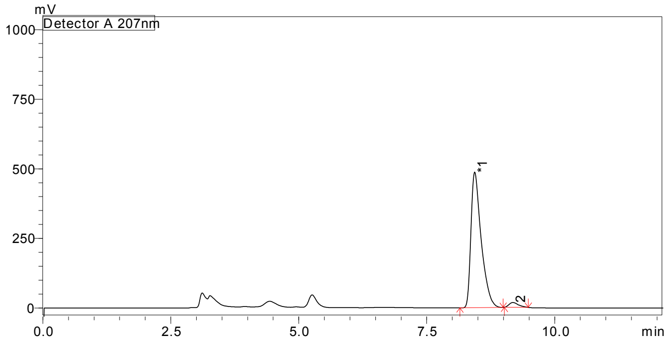

Peak#	Ret. Time	Area%
1	8.437	96.843
2	9.184	3.157

Supplementary Figure 43. HPLC chromatogram for compound **5a′**

The absolute configuration of **5a** is determined to be ***S*** and other products' configurations (**3** and **5**) were deduced by analogy.

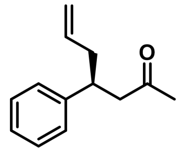

5a'

(*S*)-4-phenylhept-6-en-2-one (**5a′**)

16 mg, colourless oil, 62% yield (two steps).

^{1}H NMR (400 MHz, CDCl$_3$) δ 7.31-7.25 (m, 2H), 7.22-7.12 (m, 3H), 5.70-5.56 (m, 1H), 5.05-4.91 (m, 2H), 3.31-3.21 (m, 1H), 2.82-2.67 (m, 2H), 2.36 (t, *J* = 7.1 Hz, 2H), 2.02 (s, 3H) ppm.

^{13}C NMR (101 MHz, CDCl$_3$) δ 207.73, 144.01, 136.12, 128.44, 127.41, 126.42, 116.74, 49.47, 40.85, 40.67, 30.66 ppm.

HRMS (ESI) m/z [M+NH$_4$]$^+$: calcd. 206.1539, found. 206.1540.

IR (film): ν_{max} (cm^{-1}) 3063, 2925, 1717, 1653, 1494, 1455, 1261, 1087, 801, 700.

The absolute configuration of **13** is determined by comparing ^{1}H NMR data of **Me** in **13′** with a literature compound **13″** as below[6].

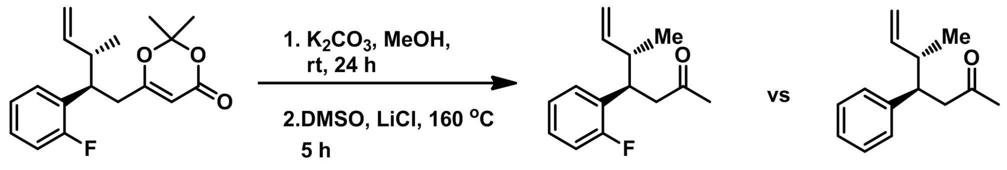

13 13' 13"

^{1}H NMR data of **Me** group in **13″**: (CCl$_4$) δ of *anti*-**13″** 0.95 (3H, d, *J* = 7.0 Hz); δ of *syn*-**13″** 0.79 (3H, d, *J* = 6.7 Hz).

^{1}H NMR data of **Me** group in **13′**: (CCl$_4$) δ of major isomer (*anti*-**13′**) 1.00 (3H, d, *J* = 6.0 Hz); δ of minor isomer (*syn*-**13′**) 0.84 (3H, d, *J* = 6.4 Hz).

^{1}H NMR data of **Me** group in **13′**: (CDCl$_3$) δ of major isomer (*anti*-**13′**) 0.99 (3H, d, *J* = 6.7 Hz); δ of minor isomer (*syn*-**13′**) 0.82 (3H, d, *J* = 6.7 Hz).

Therefore, the absolute configurations of two stereogenic carbon centers of **13** are determined to be (**2*R***, **3*S***).

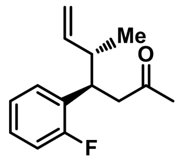

(4*R*,5*S*)-4-(2-fluorophenyl)-5-methylhept-6-en-2-one (**13′**)

13 mg, colourless oil, 59% yield (two steps).

^{1}H NMR of *anti*-13′ (400 MHz, $CDCl_3$) δ 7.19-6.96 (m, 4H), 5.67-5.52 (m, 1H), 4.96-4.83 (m, 2H), 3.56-3.48 (m, 1H), 2.84 (d, J = 7.3 Hz, 2H), 2.54-2.43 (m, 1H), 2.055 (s, 3H), 0.98 (d, J = 6.8 Hz, 3H) ppm.
^{1}H NMR of *syn*-13′ (400 MHz, $CDCl_3$) δ 7.19-6.96 (m, 4H), 5.72-5.62 (m, 1H), 5.11-4.99 (m, 2H), 3.32-3.23 (m, 1H), 2.84 (d, J = 7.3 Hz, 2H), 2.44-2.35 (m, 1H), 2.046 (s, 3H), 0.82 (d, J = 6.7 Hz, 3H) ppm.
^{13}C NMR (101 MHz, $CDCl_3$) δ 207.54, 160.95 (d, J = 244.8 Hz), 141.19, 129.7 (d, J = 5.0 Hz), 128.90, 127.85 (d, J = 8.4 Hz), 123.66 (d, J = 3.4 Hz), 115.52 (d, J = 23.3 Hz), 114.74, 45.89, 42.02, 38.92, 30.31, 17.57 ppm.
HRMS (ESI) m/z $[M+NH_4]^+$: calcd. 221.1336, found. 221.1336.
IR (film): ν_{max} (cm^{-1}) , 3055, 2925, 2853, 1716, 1491, 1265, 1227, 919, 737.
Optical rotation: $[\alpha]_D^{25}$ =14.32 (c = 0.50, $CHCl_3$).

Supplementary Figures of NMR Spectra

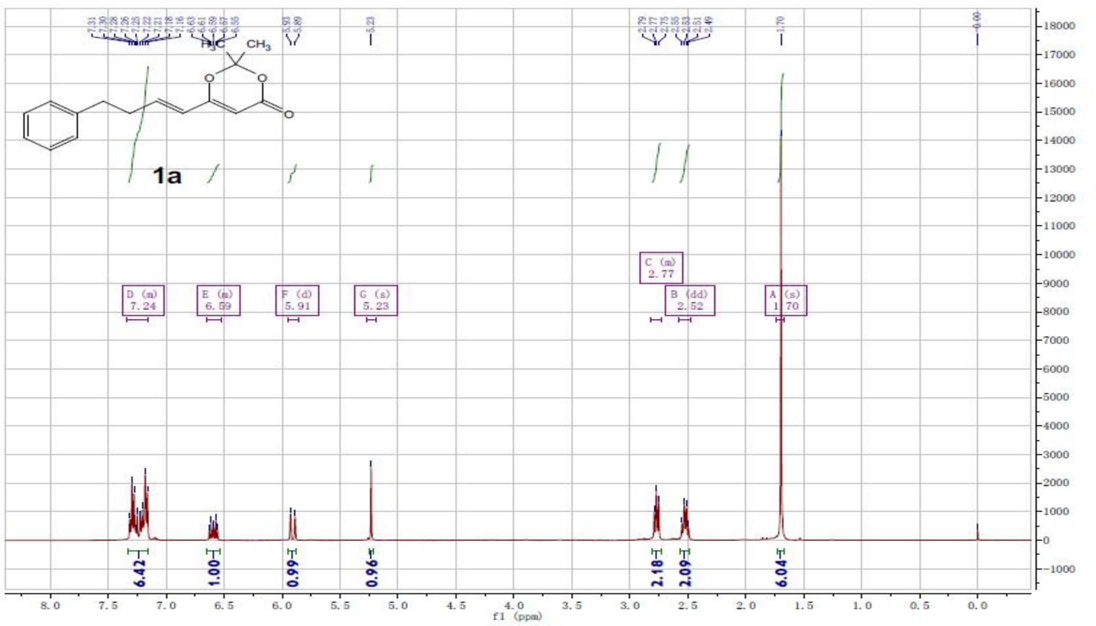

Supplementary Figure 44. ^{1}H NMR spectrum for compound **1a**

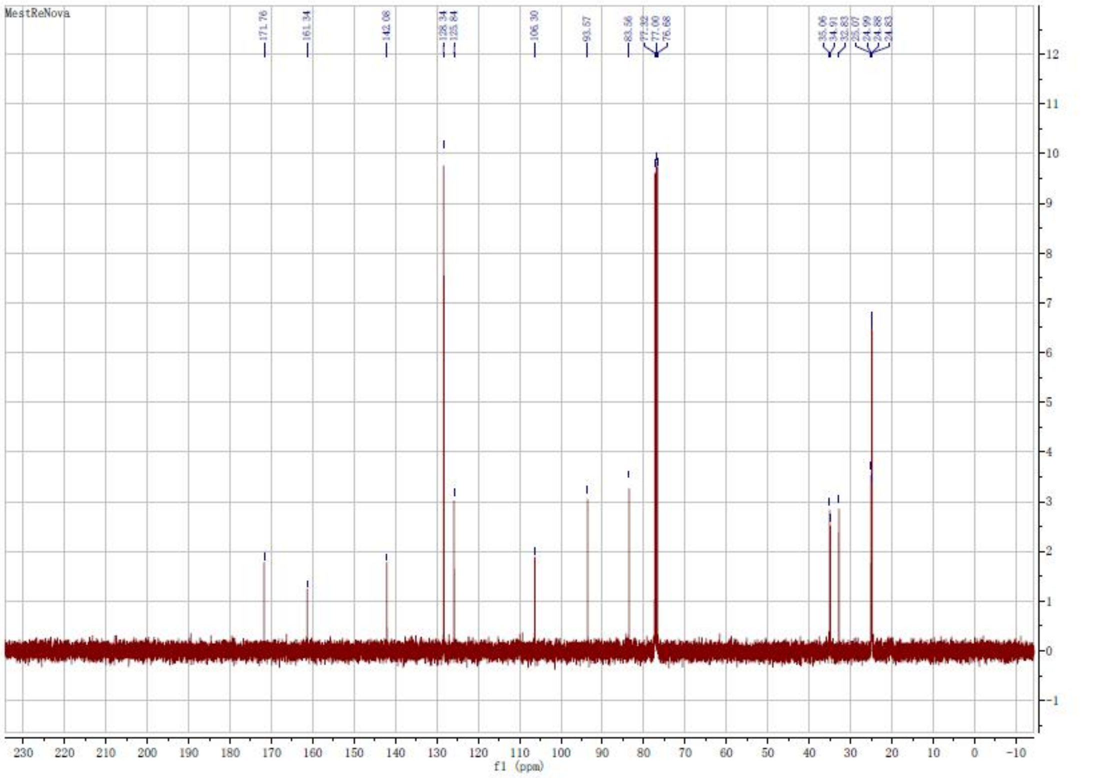

Supplementary Figure 45. ^{13}C NMR spectrum for compound **1a**

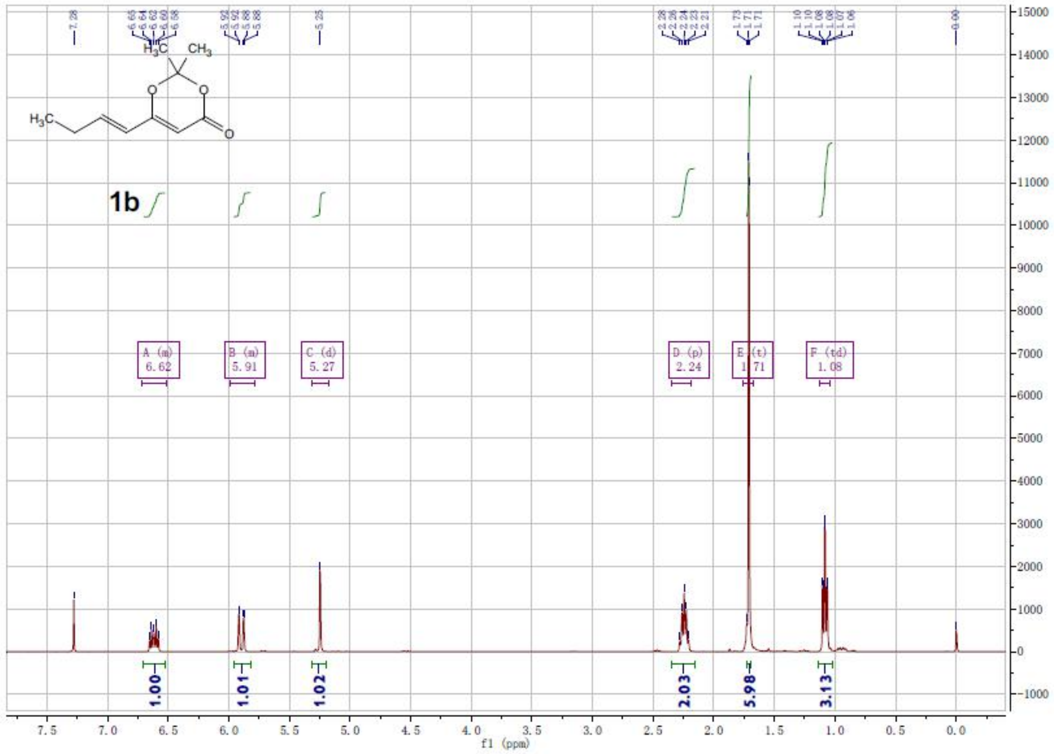

Supplementary Figure 46. ^{1}H NMR spectrum for compound **1b**

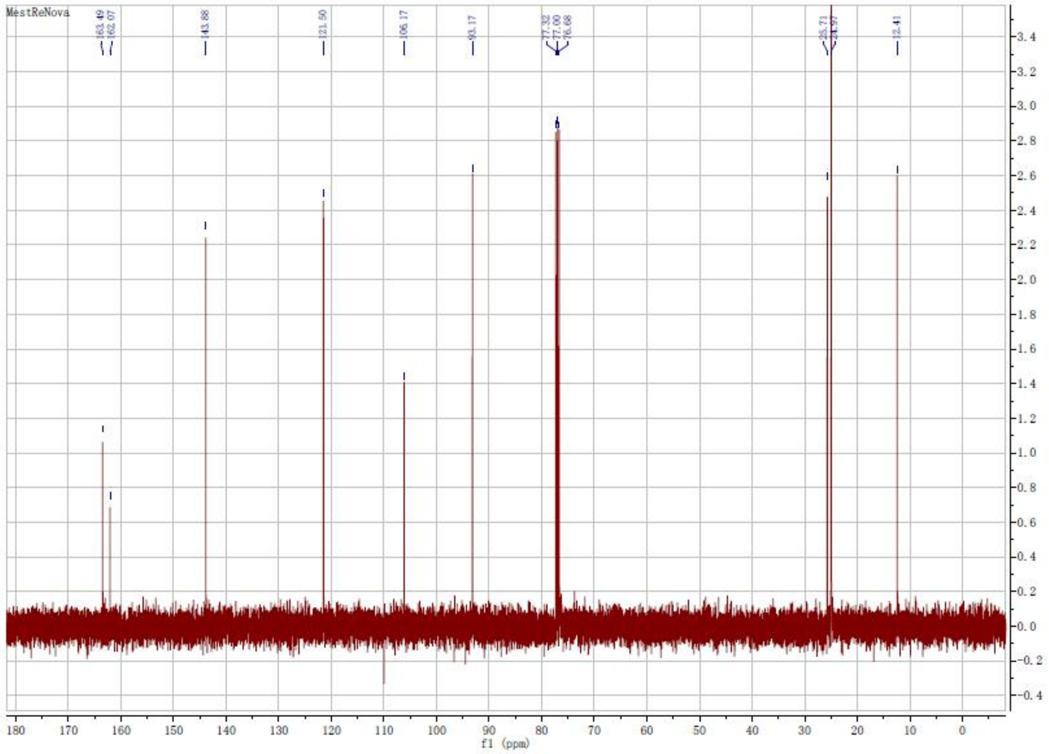

Supplementary Figure 47. ^{13}C NMR spectrum for compound **1b**

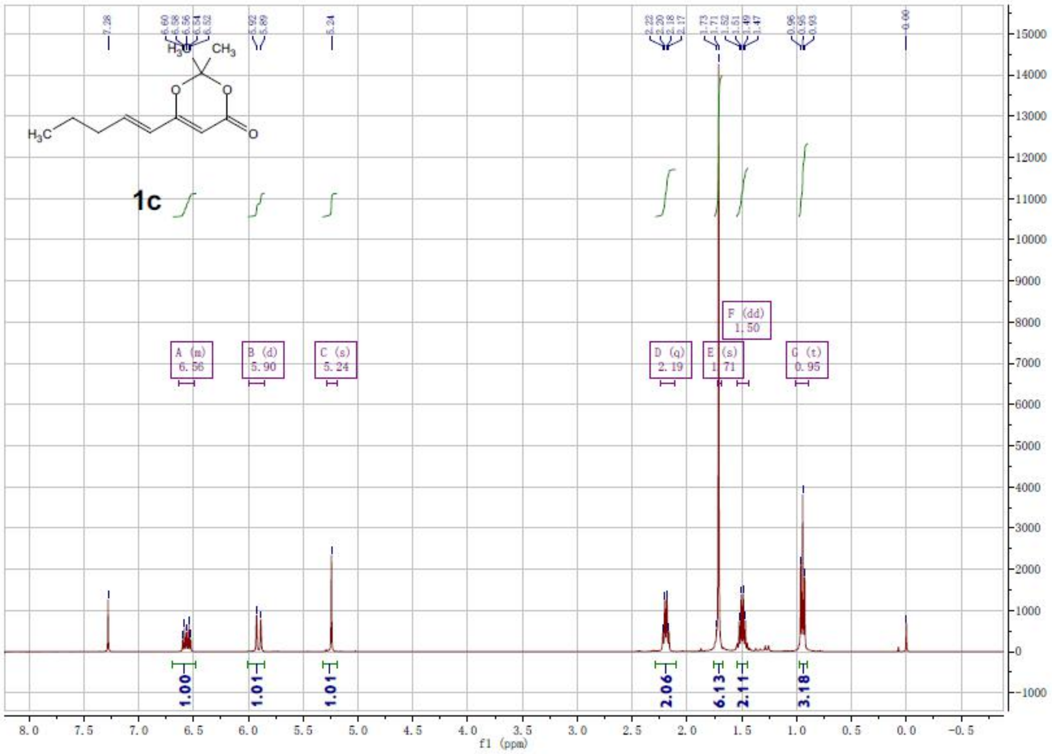

Supplementary Figure 48. ^{1}H NMR spectrum for compound **1c**

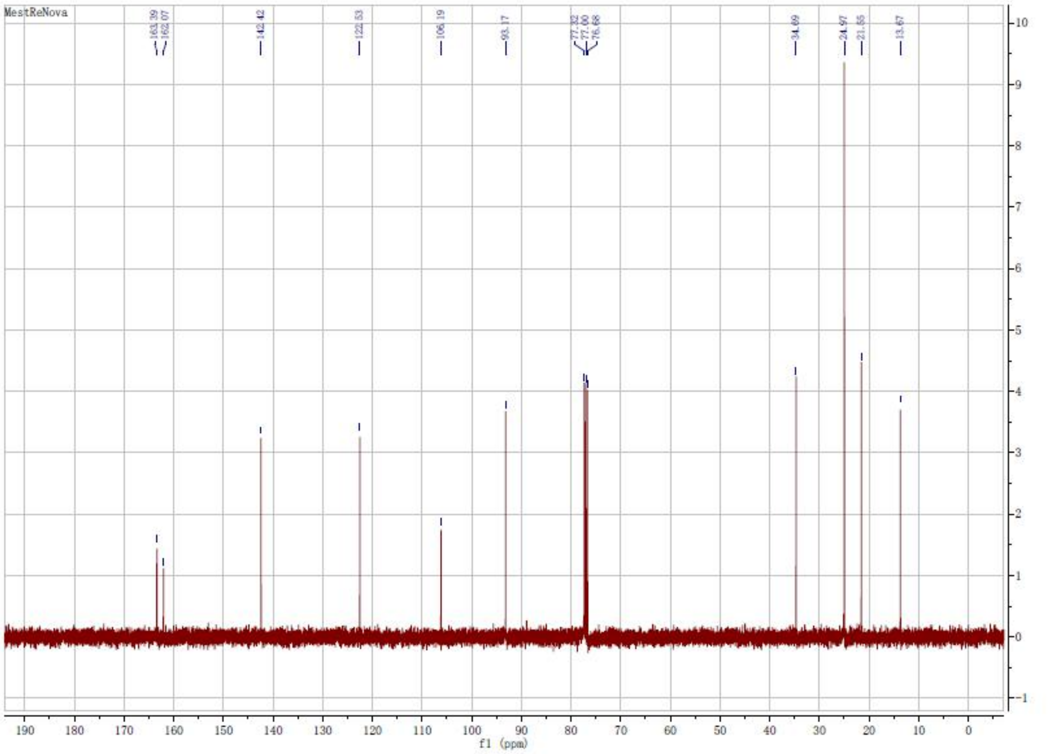

Supplementary Figure 49. ^{13}C NMR spectrum for compound **1c**

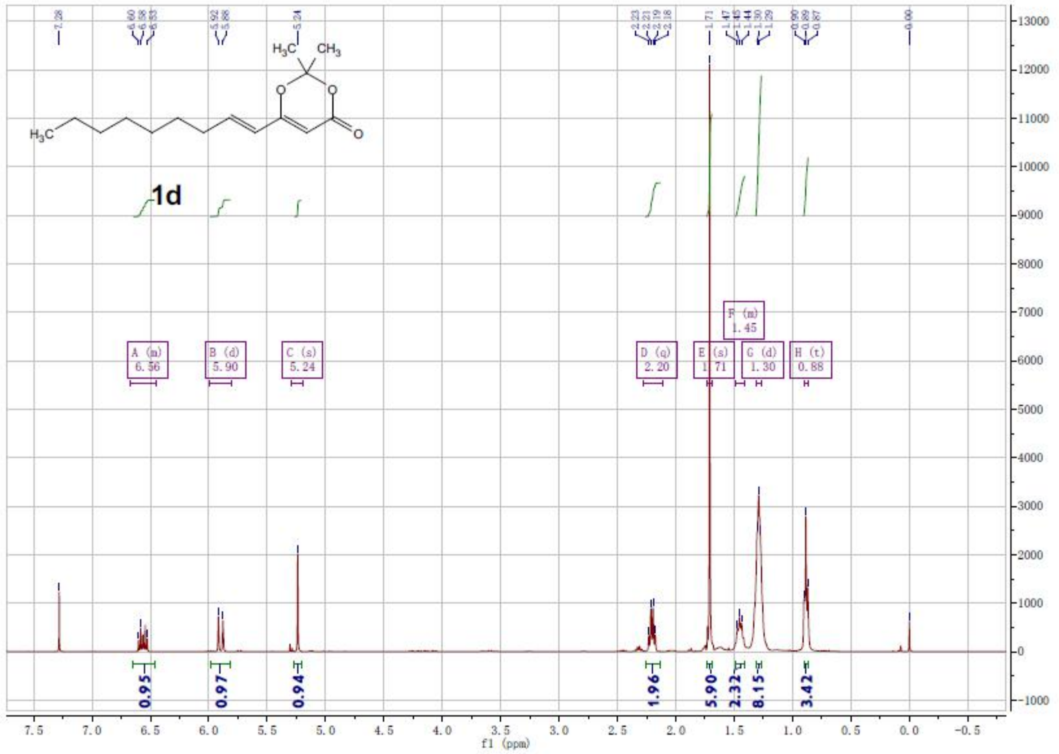

Supplementary Figure 50. ^{1}H NMR spectrum for compound **1d**

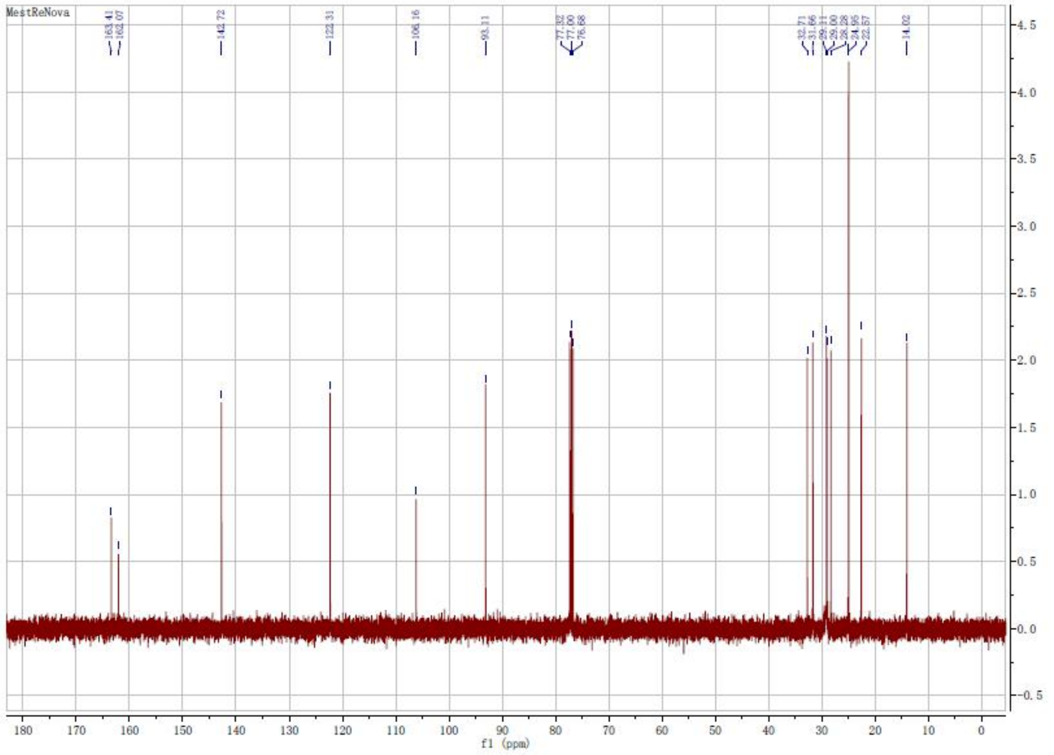

Supplementary Figure 51. ^{13}C NMR spectrum for compound **1d**

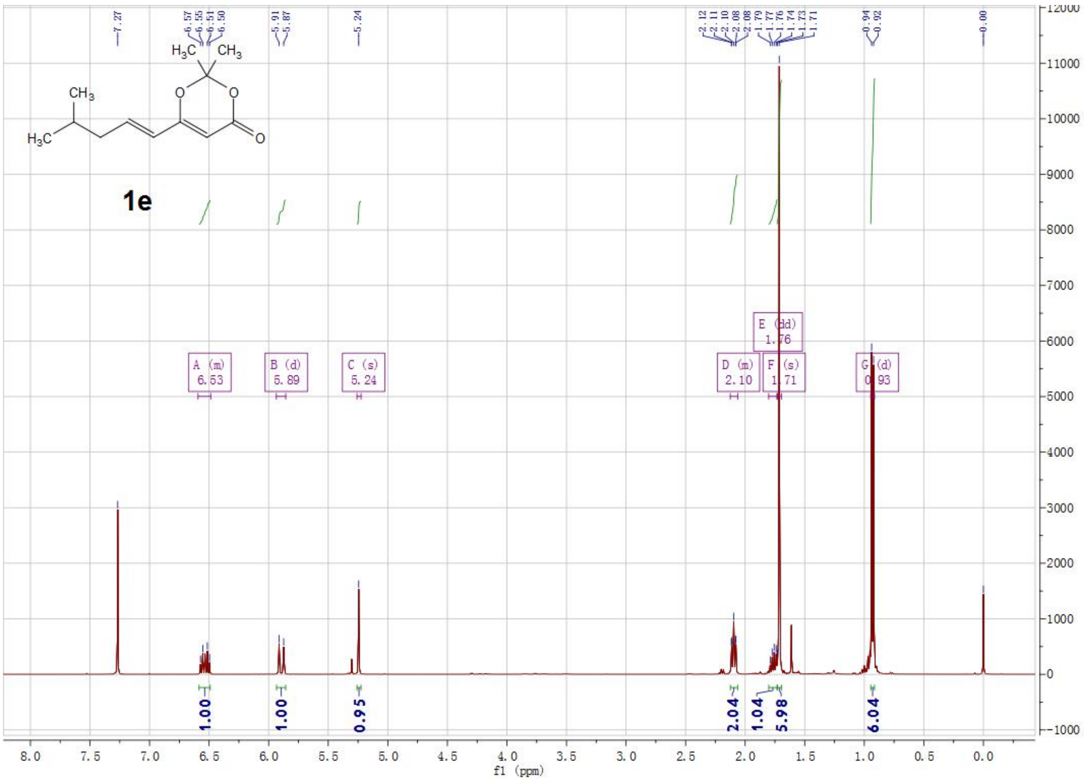

Supplementary Figure 52. ^{1}H NMR spectrum for compound **1e**

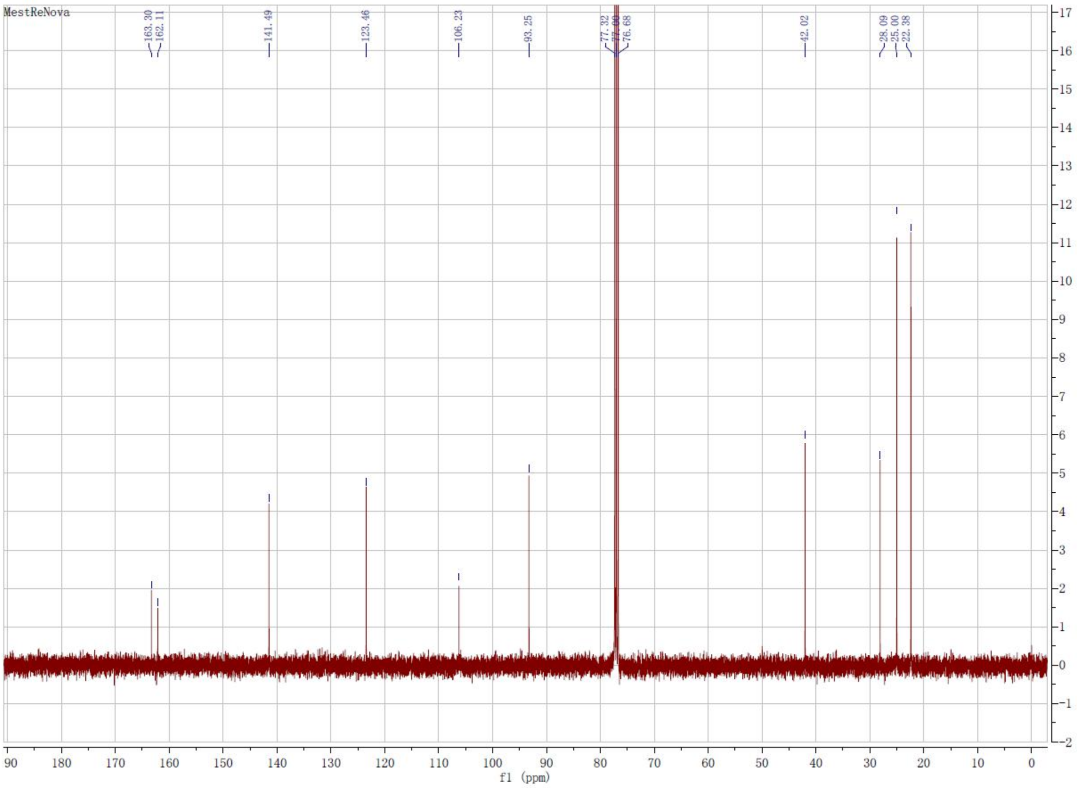

Supplementary Figure 53. ^{13}C NMR spectrum for compound **1e**

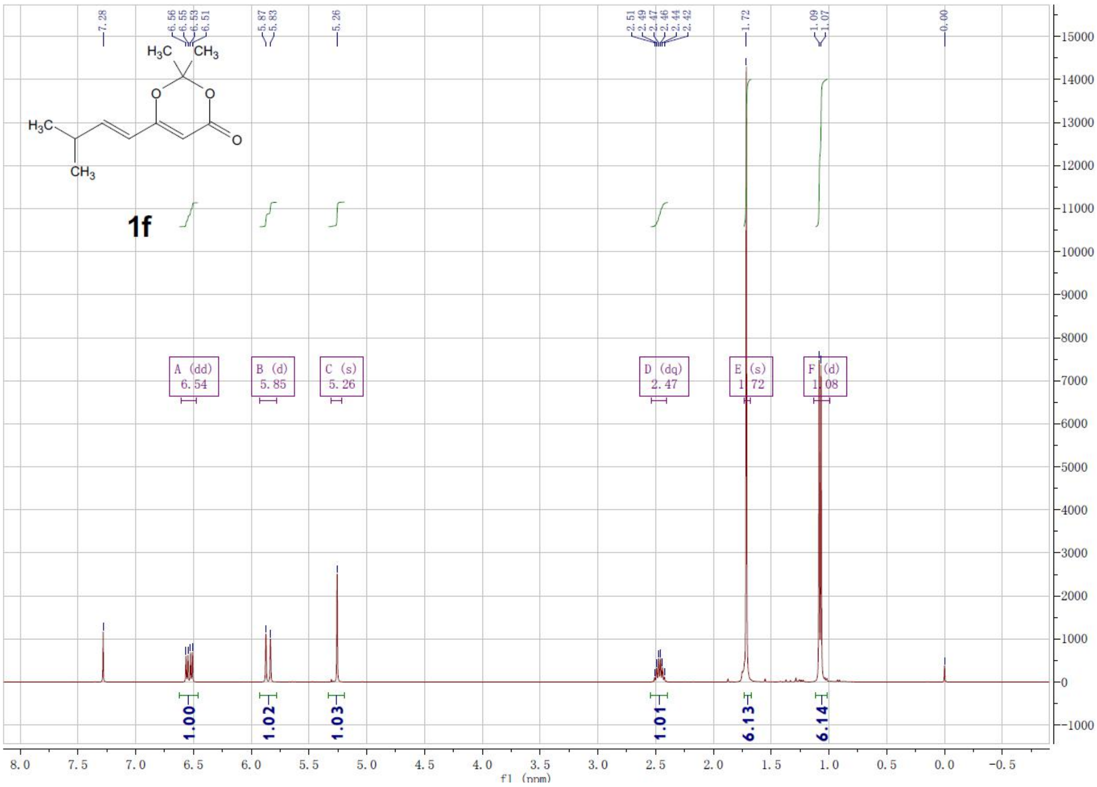

Supplementary Figure 54. ^{1}H NMR spectrum for compound **1f**

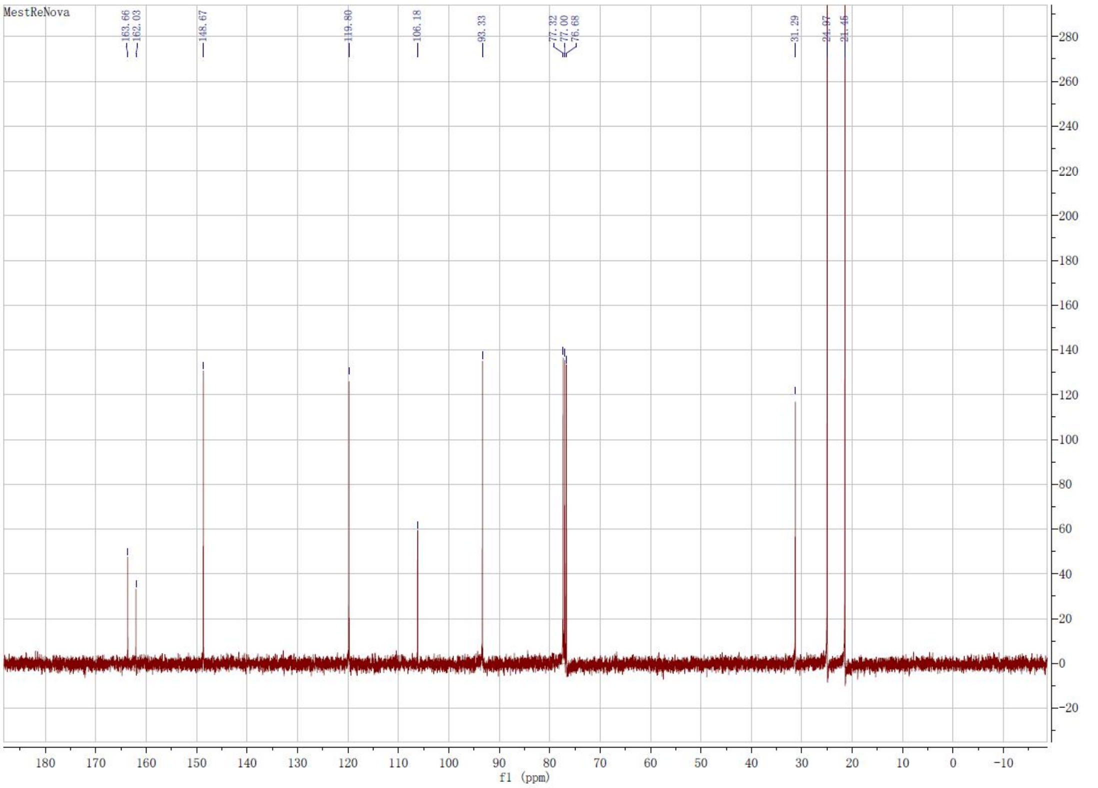

Supplementary Figure 55. ^{13}C NMR spectrum for compound **1f**

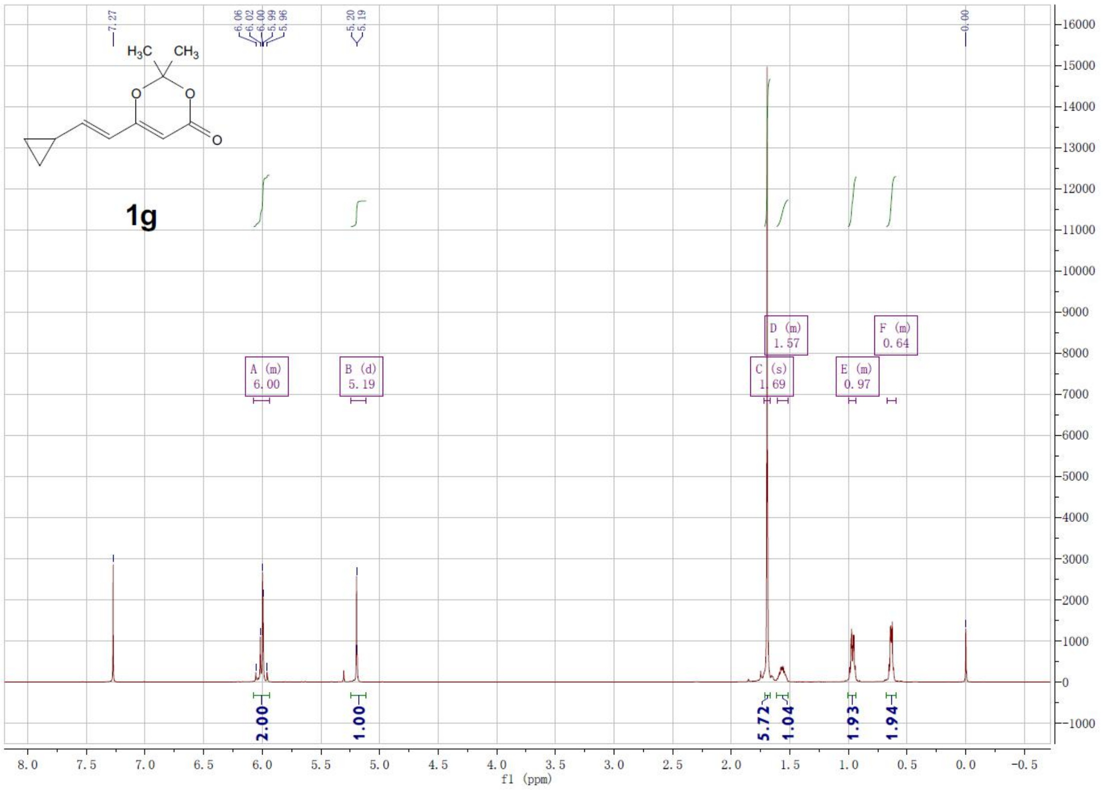

Supplementary Figure 56. ^{1}H NMR spectrum for compound **1g**

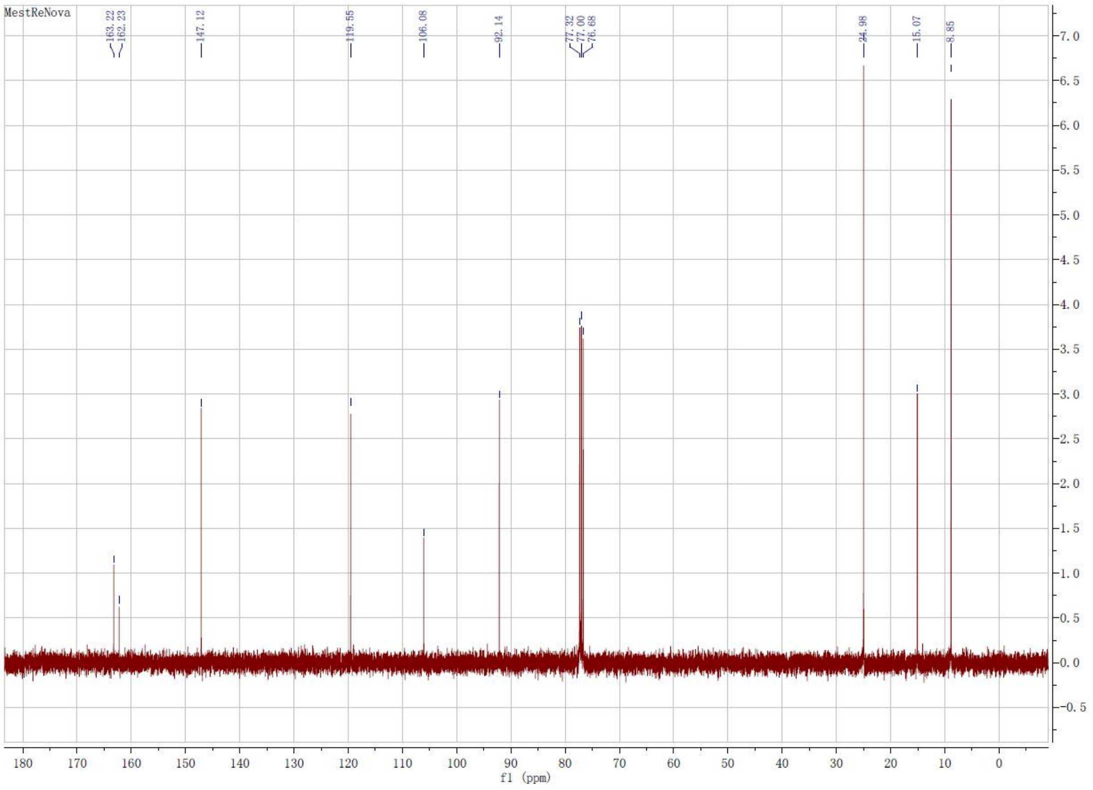

Supplementary Figure 57. ^{13}C NMR spectrum for compound **1g**

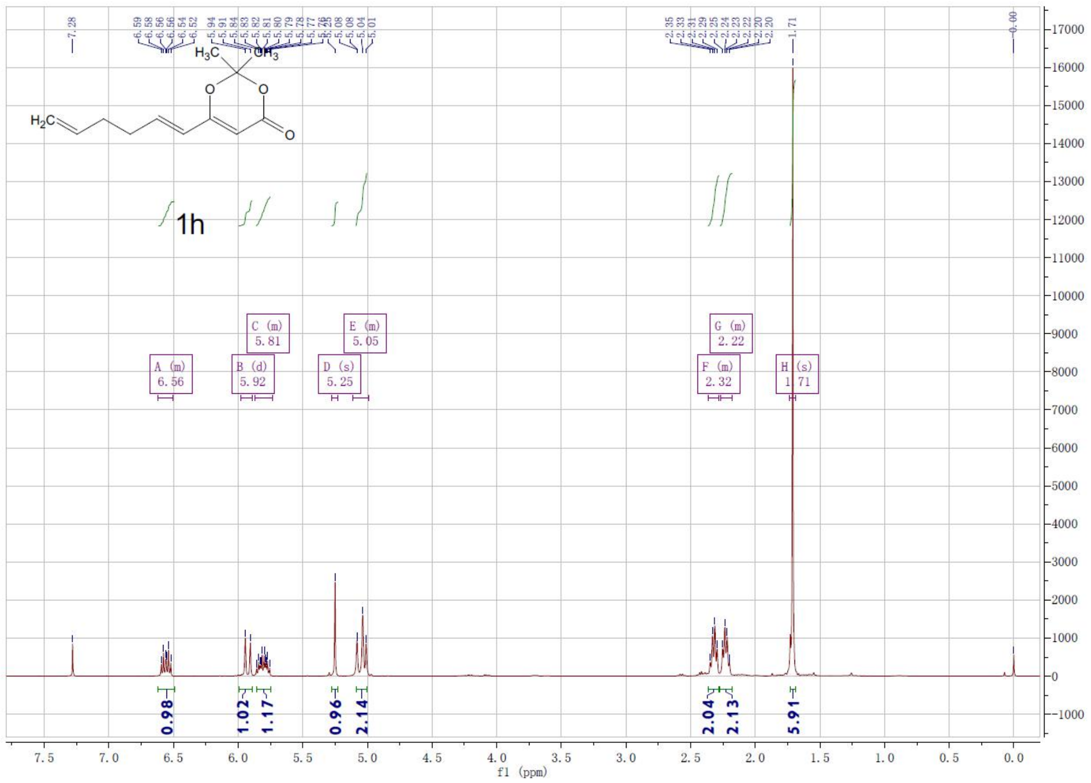

Supplementary Figure 58. ^{1}H NMR spectrum for compound **1h**

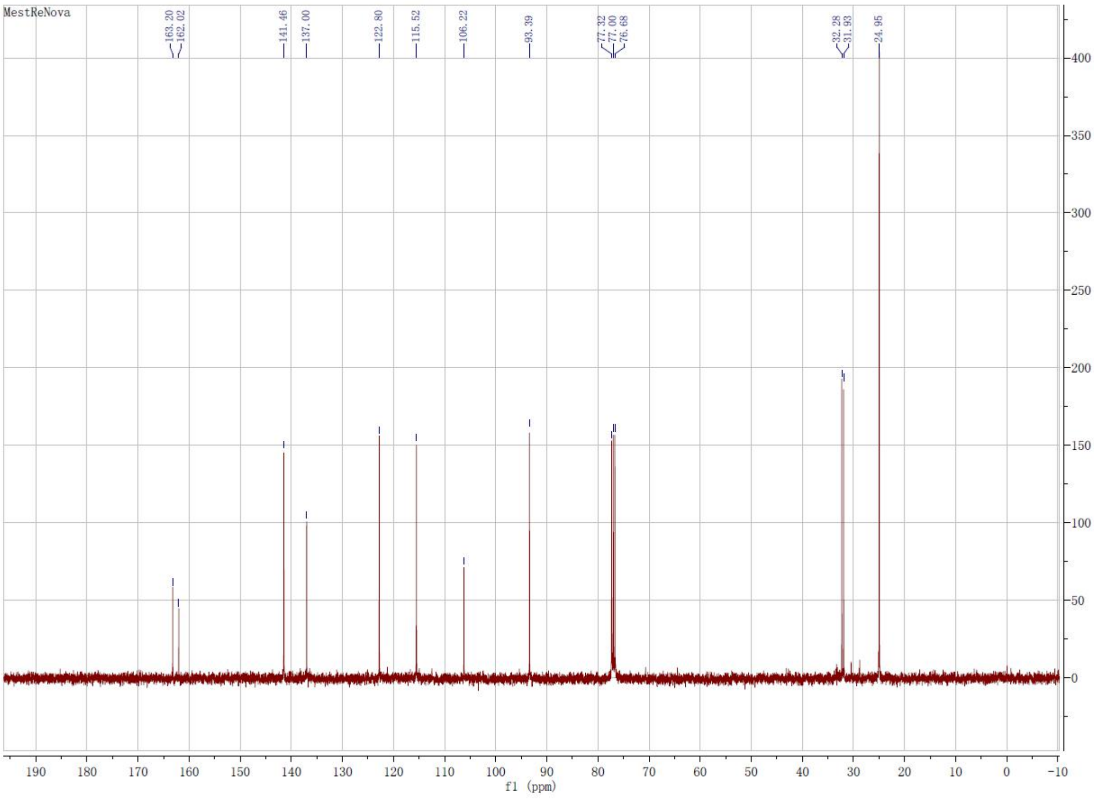

Supplementary Figure 59. ^{1}H NMR spectrum for compound **1h**

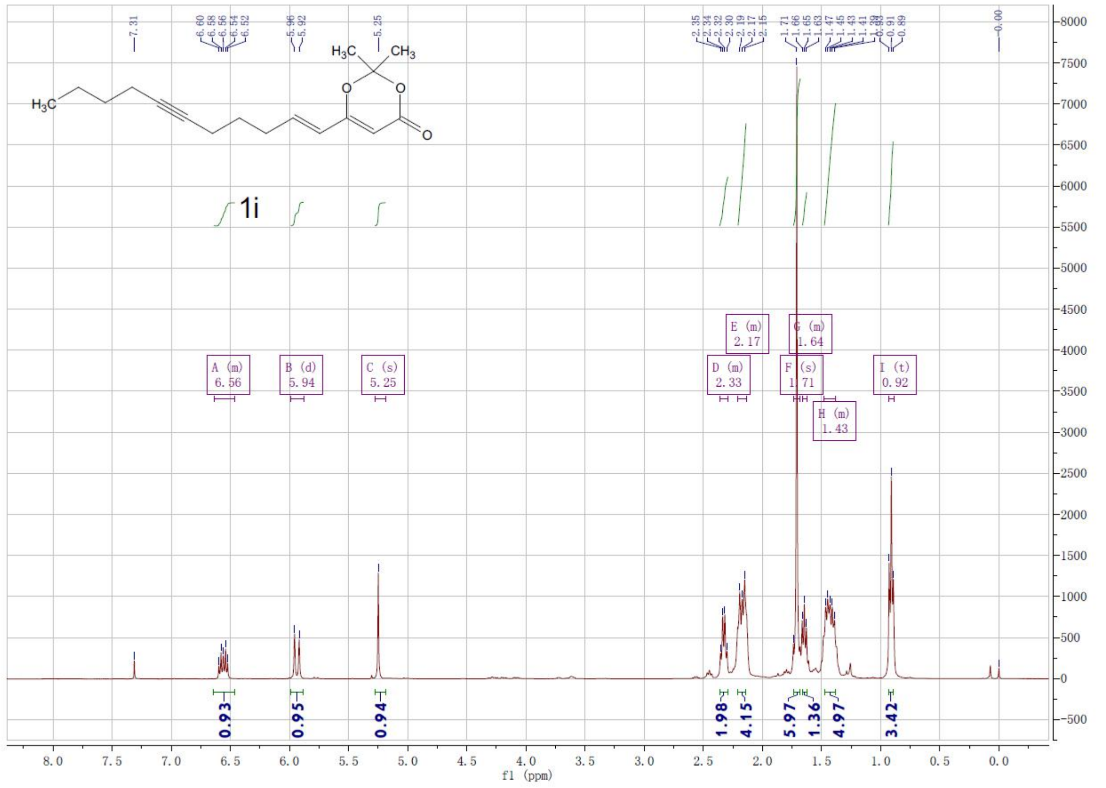

Supplementary Figure 60. ^{1}H NMR spectrum for compound **1i**

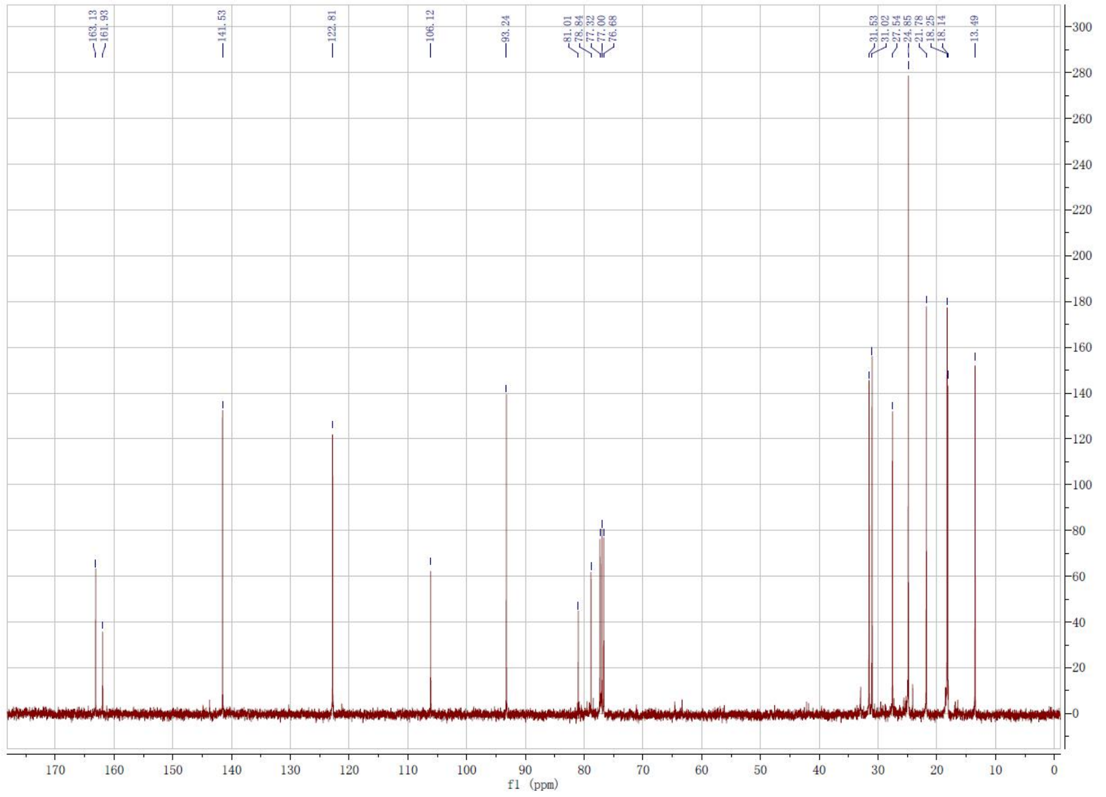

Supplementary Figure 61. ^{13}C NMR spectrum for compound **1i**

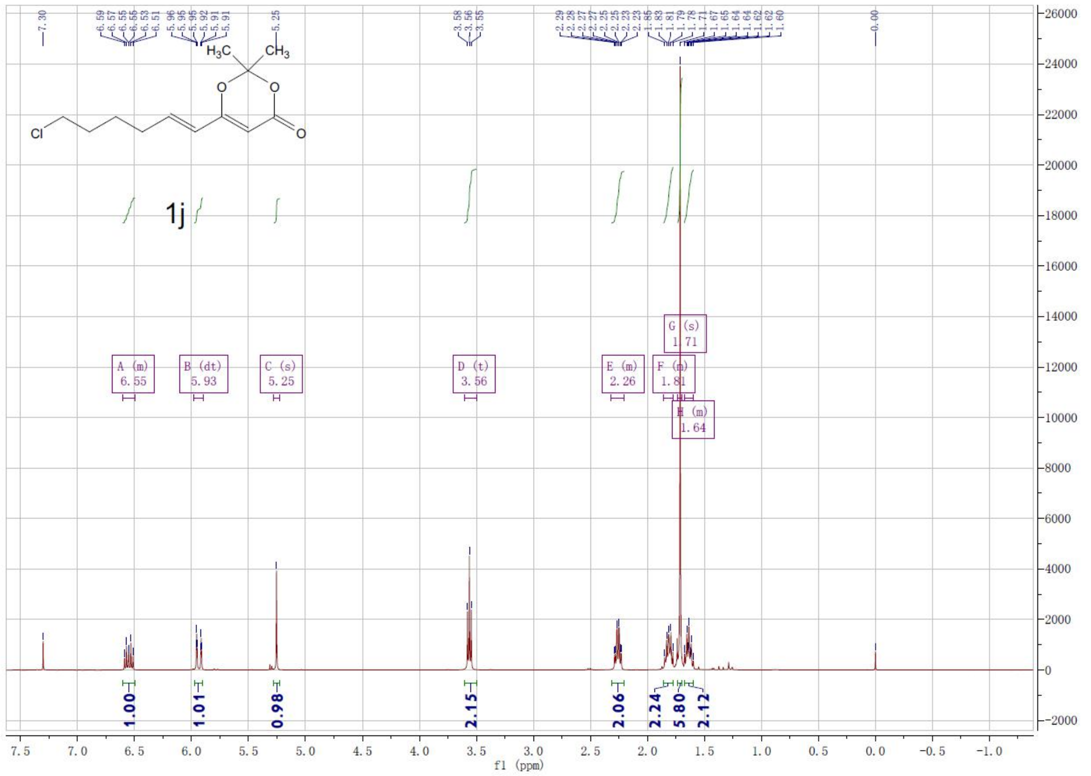

Supplementary Figure 62. ^{1}H NMR spectrum for compound **1j**

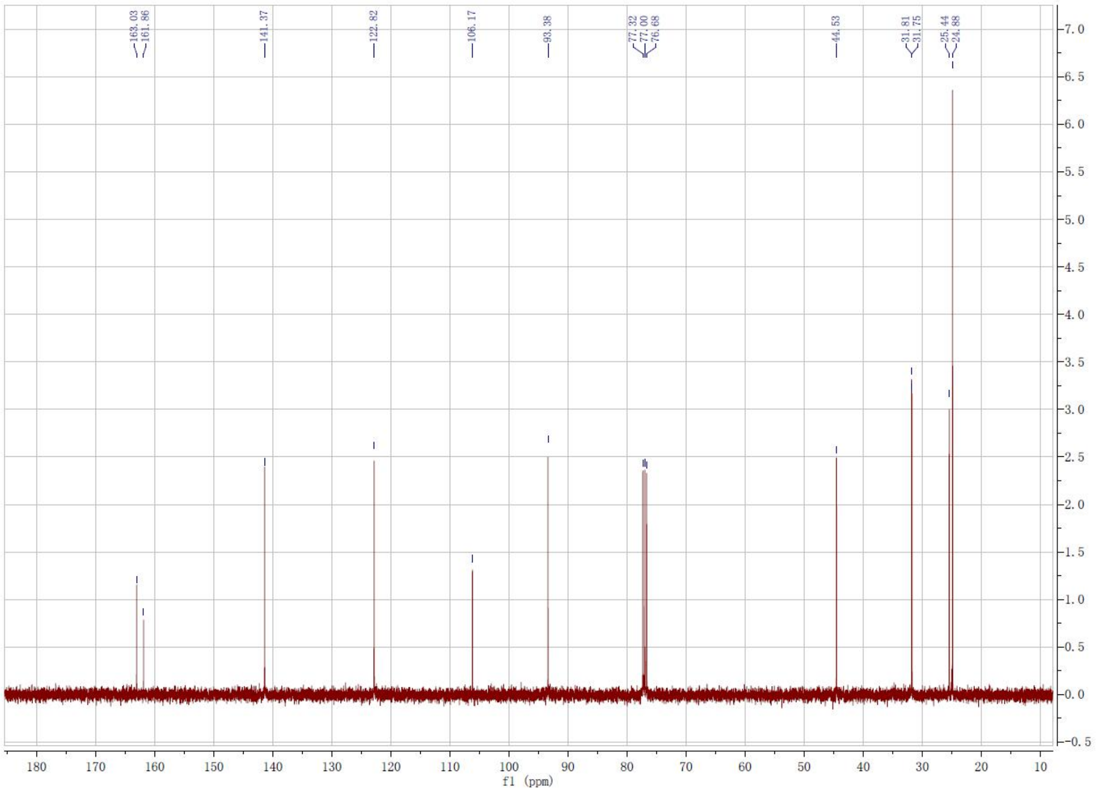

Supplementary Figure 63. ^{13}C NMR spectrum for compound **1j**

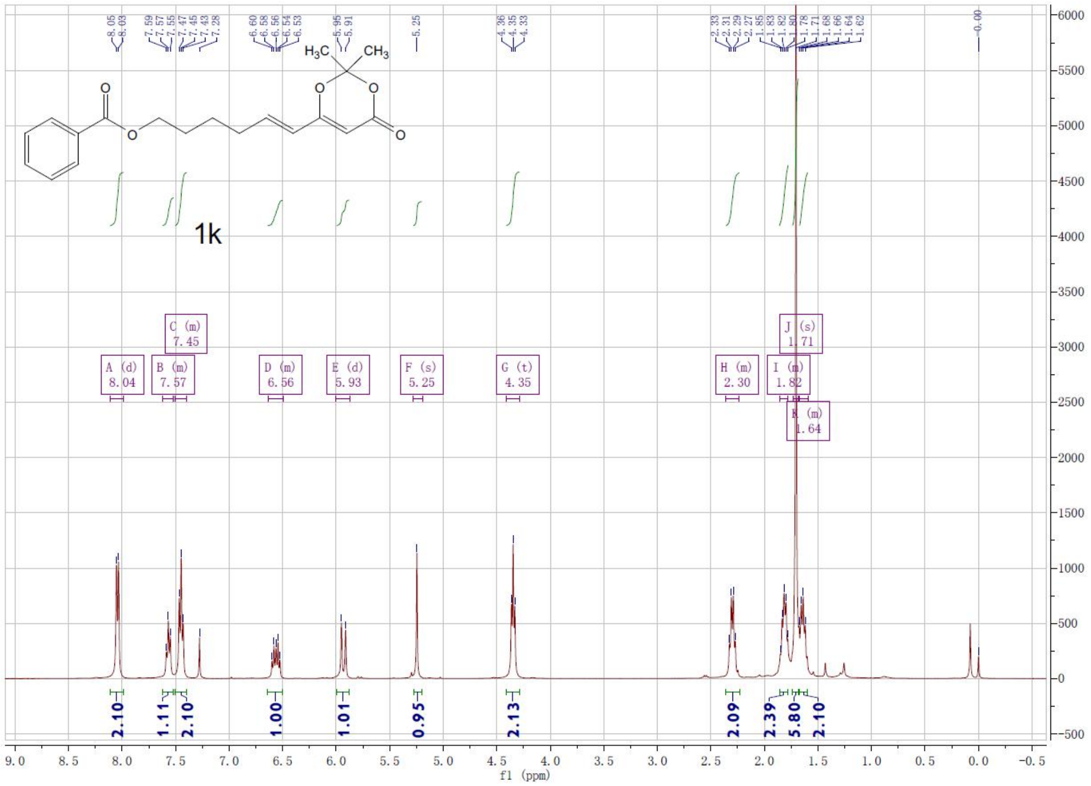

Supplementary Figure 64. ^{1}H NMR spectrum for compound **1k**

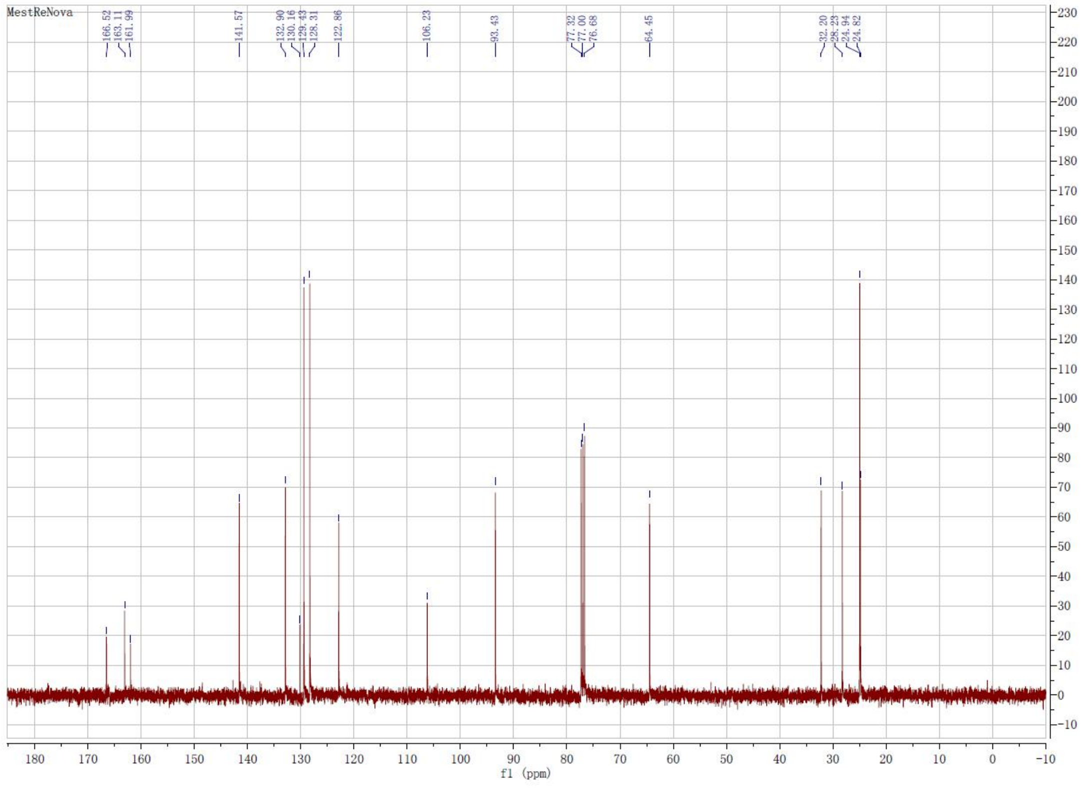

Supplementary Figure 65. ^{13}C NMR spectrum for compound **1k**

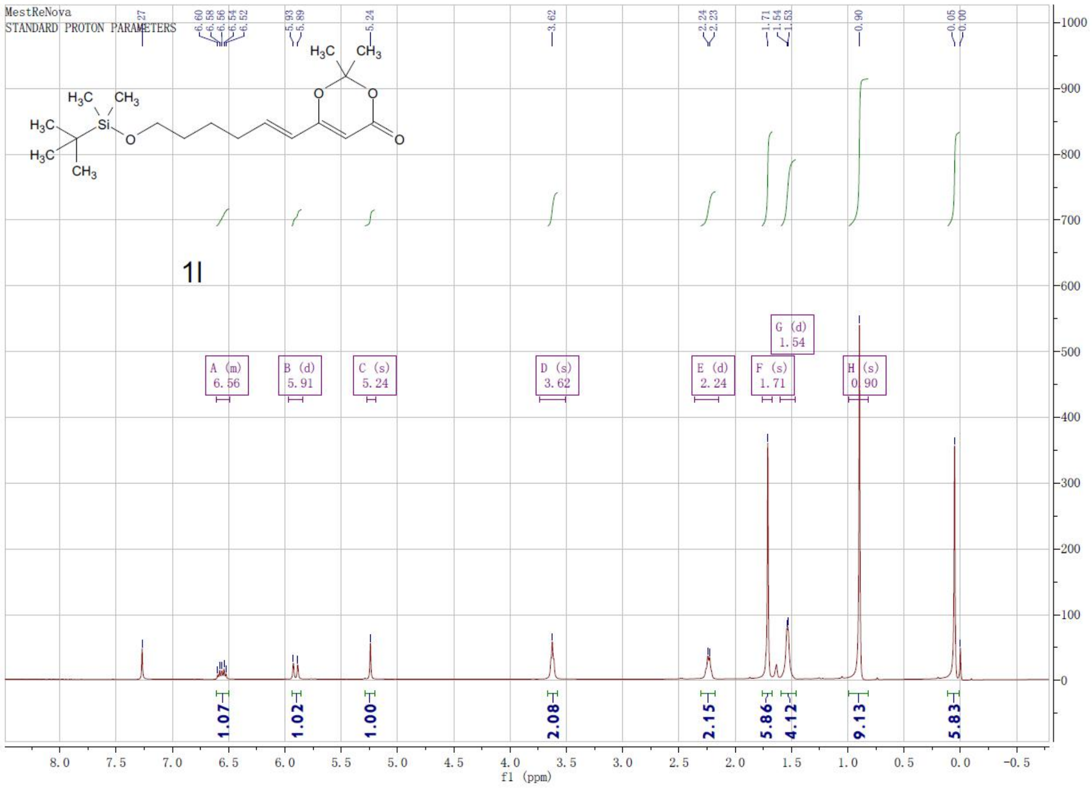

Supplementary Figure 66. ^{1}H NMR spectrum for compound **1l**

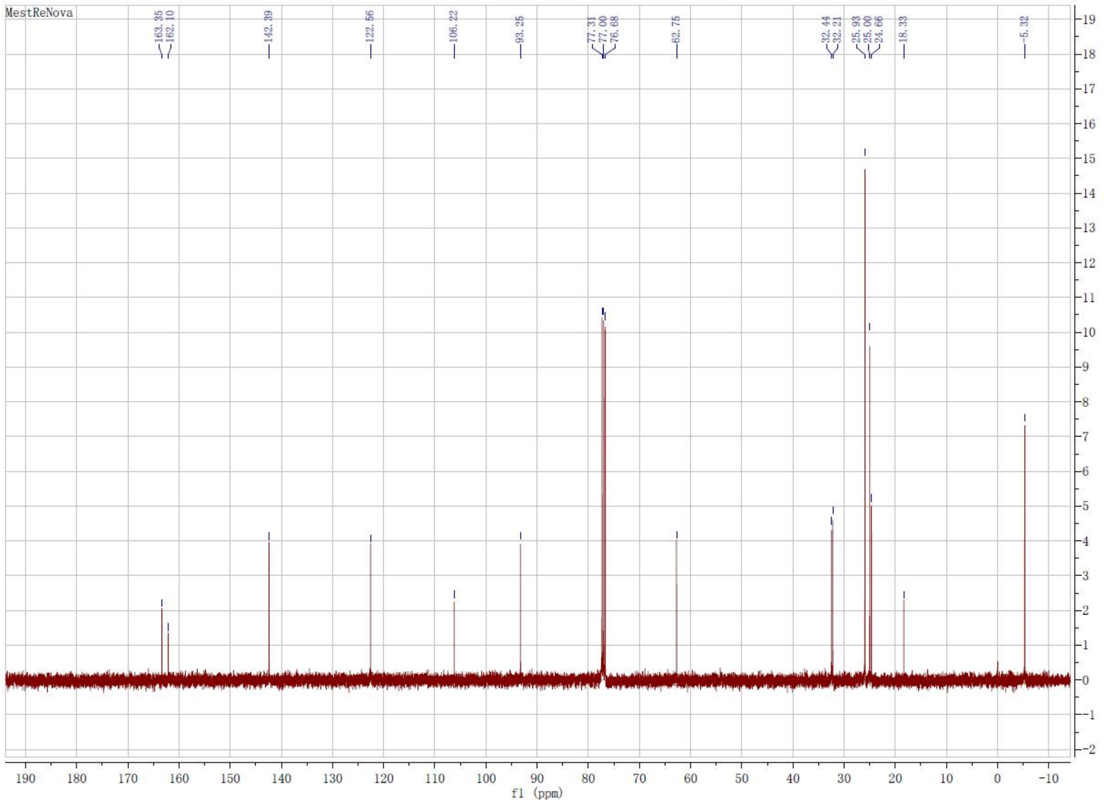

Supplementary Figure 67. ^{13}C NMR spectrum for compound **1l**

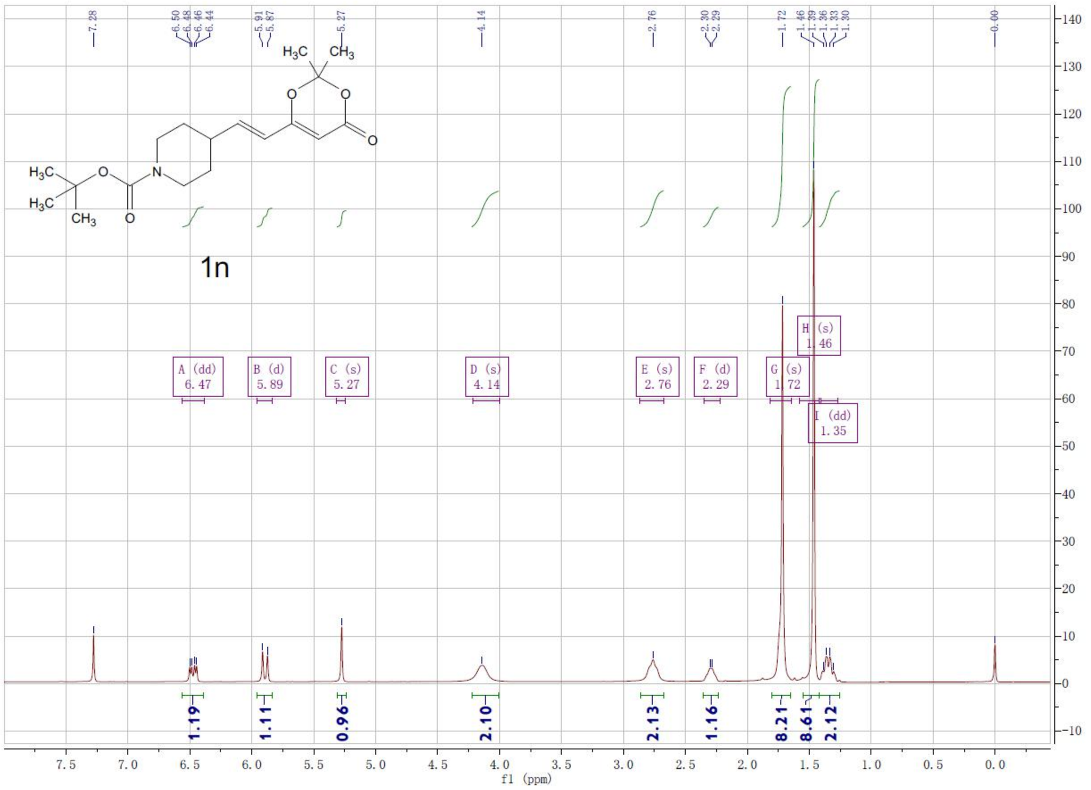

Supplementary Figure 68. ^{1}H NMR spectrum for compound **1n**

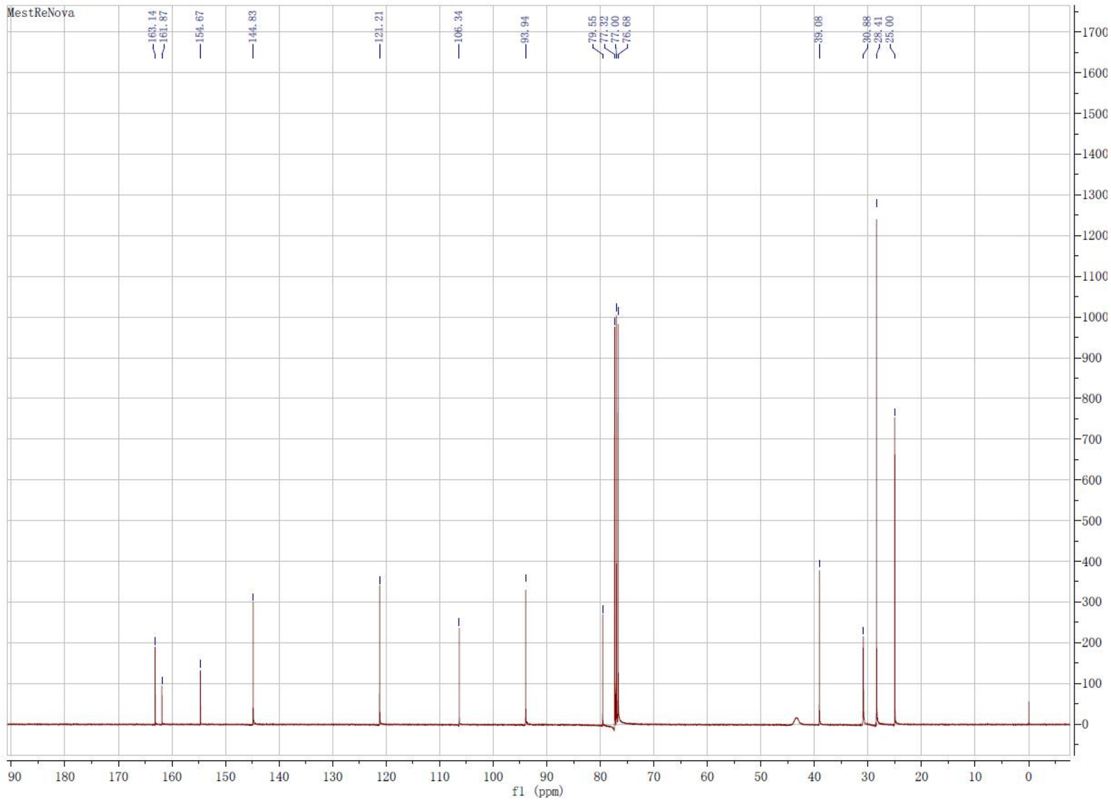

Supplementary Figure 69. ^{13}C NMR spectrum for compound **1n**

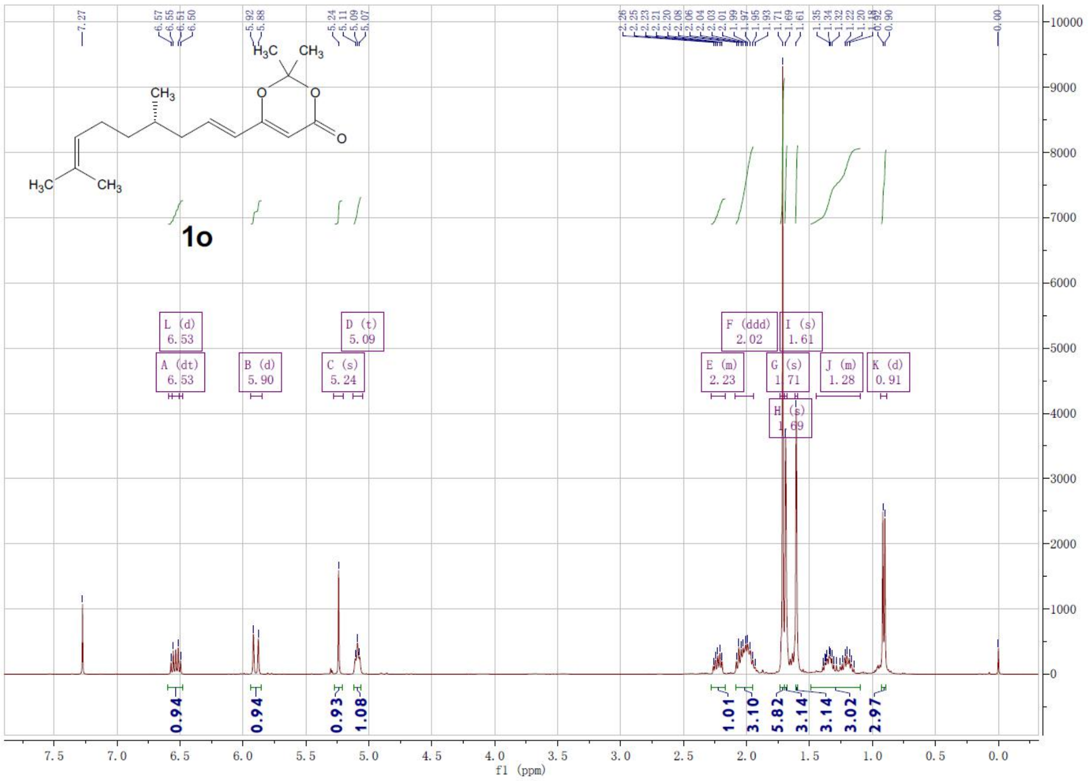

Supplementary Figure 70. ^{1}H NMR spectrum for compound **1o**

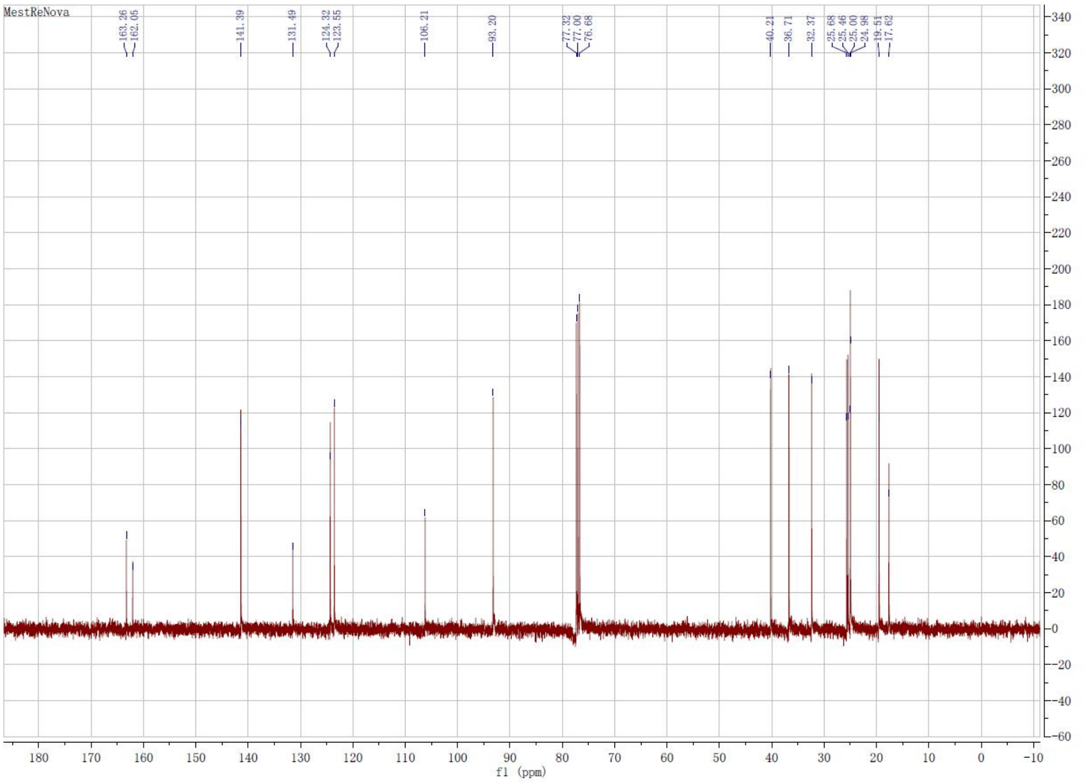

Supplementary Figure 71. ^{13}C NMR spectrum for compound **1o**

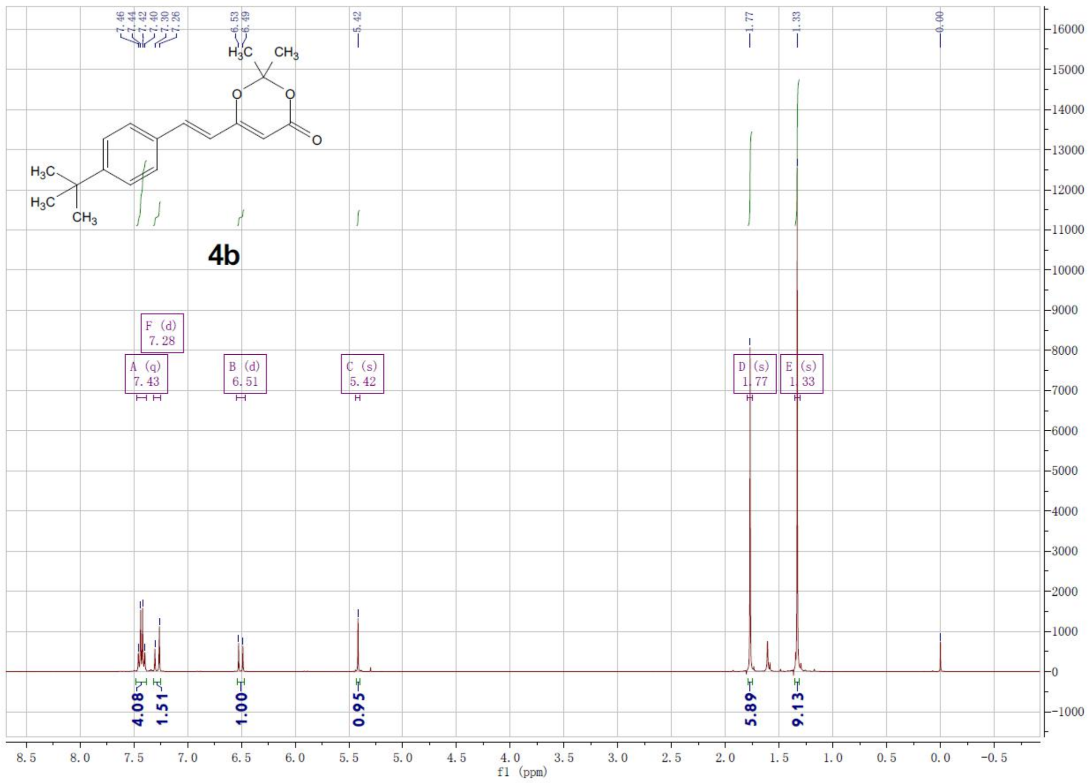

Supplementary Figure 72. ^{1}H NMR spectrum for compound **4b**

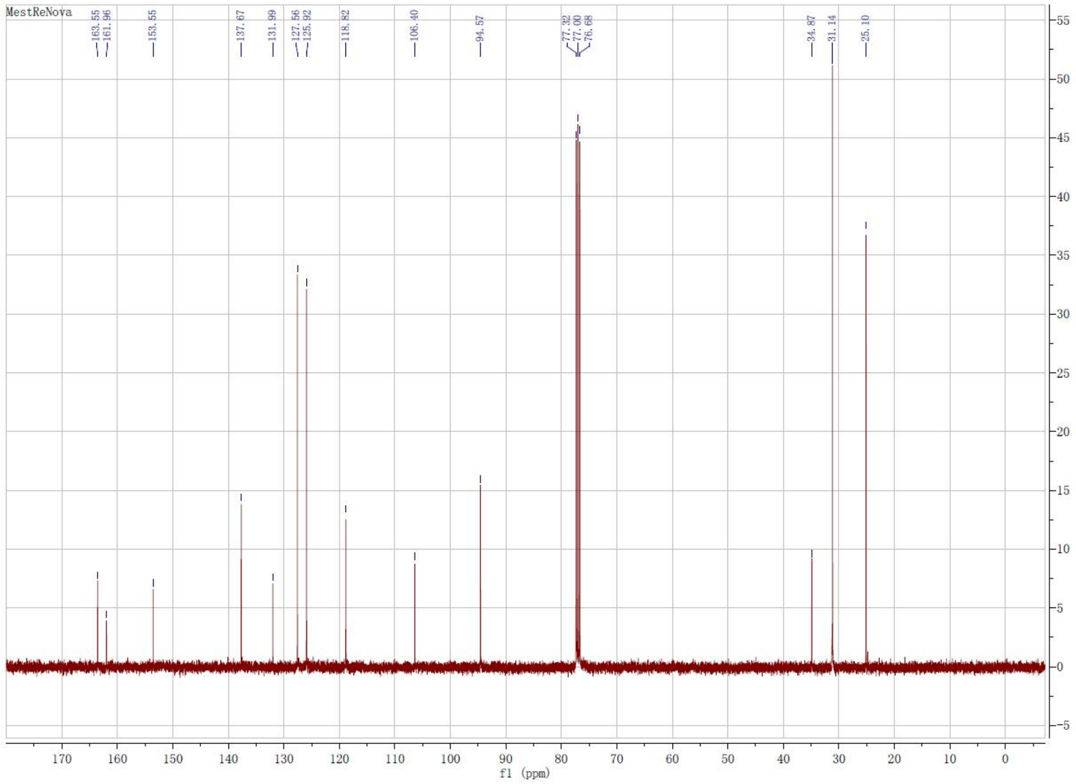

Supplementary Figure 73. ^{13}C NMR spectrum for compound **4b**

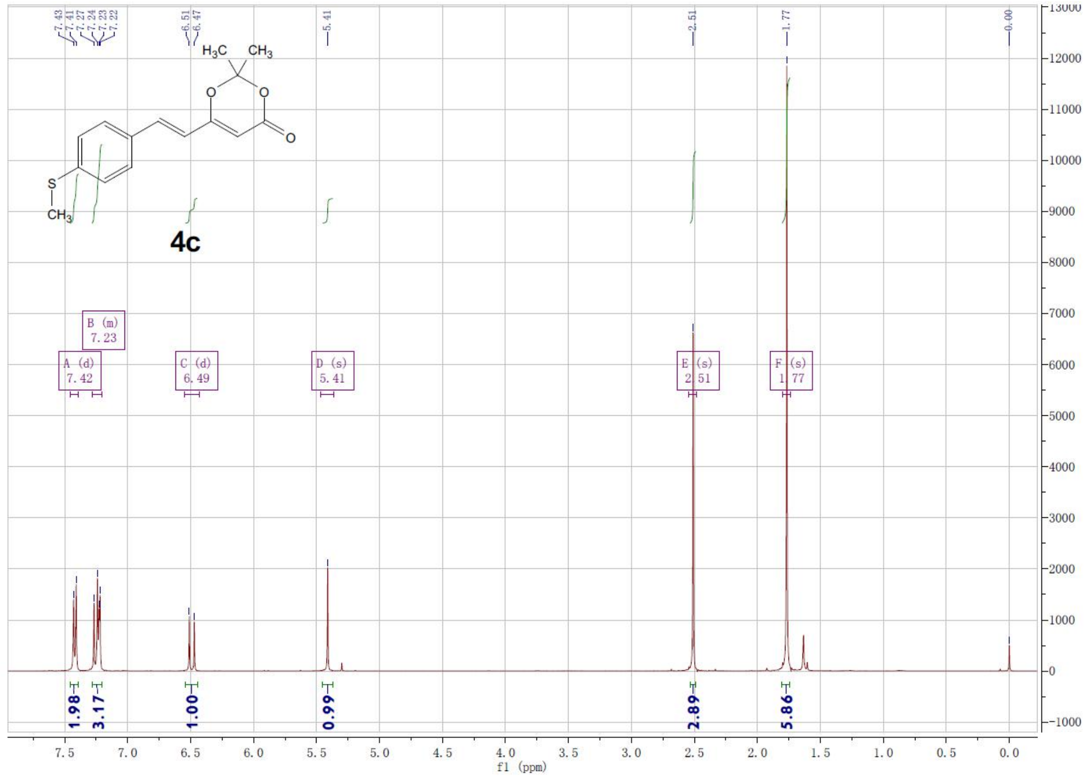

Supplementary Figure 74. ^{1}H NMR spectrum for compound **4c**

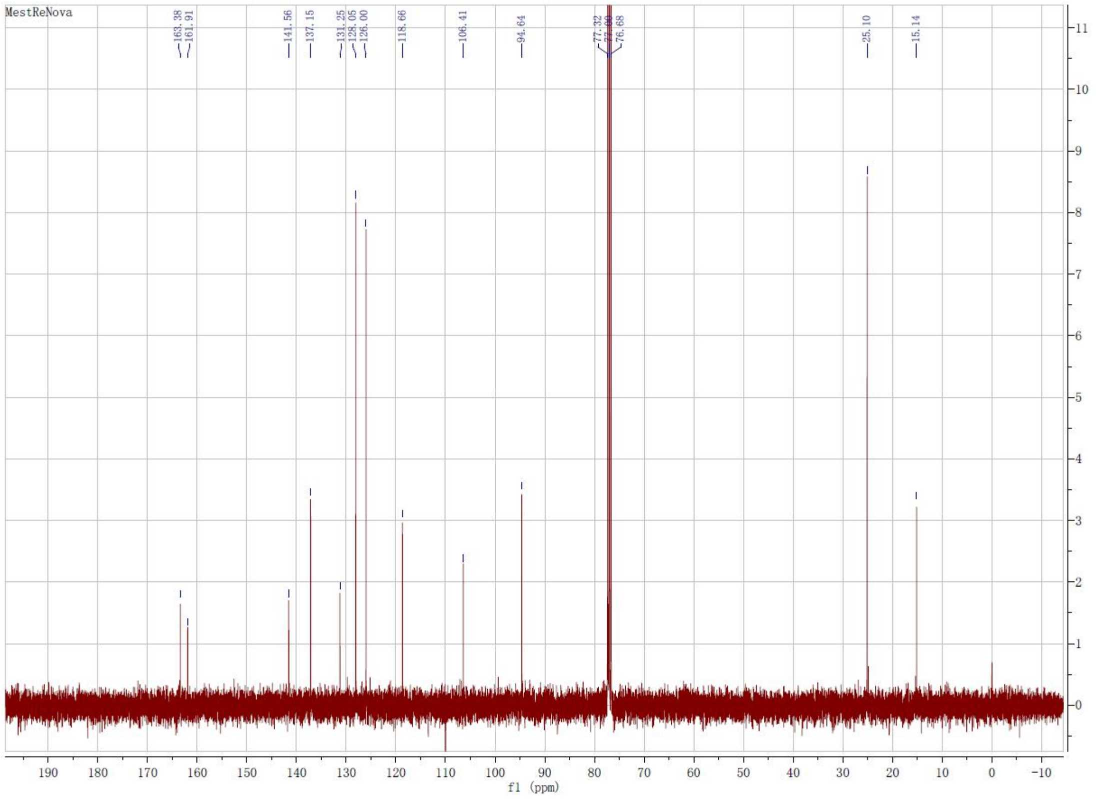

Supplementary Figure 75. ^{1}H NMR spectrum for compound **4c**

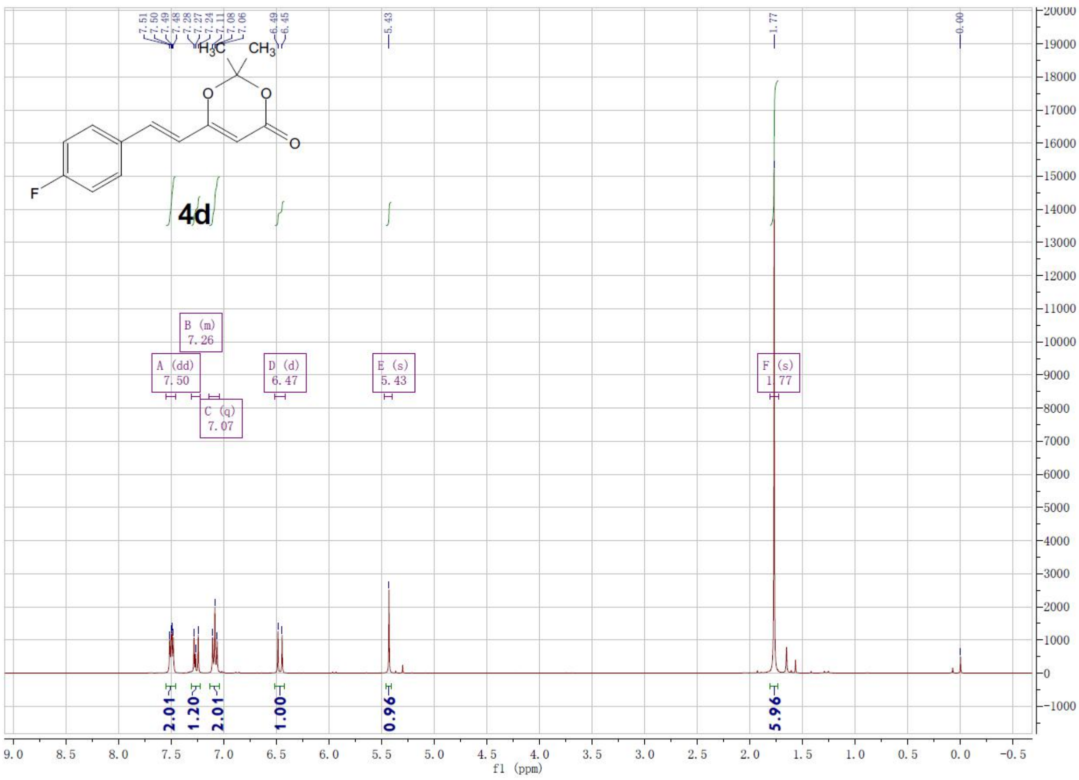

Supplementary Figure 76. ^{1}H NMR spectrum for compound **4d**

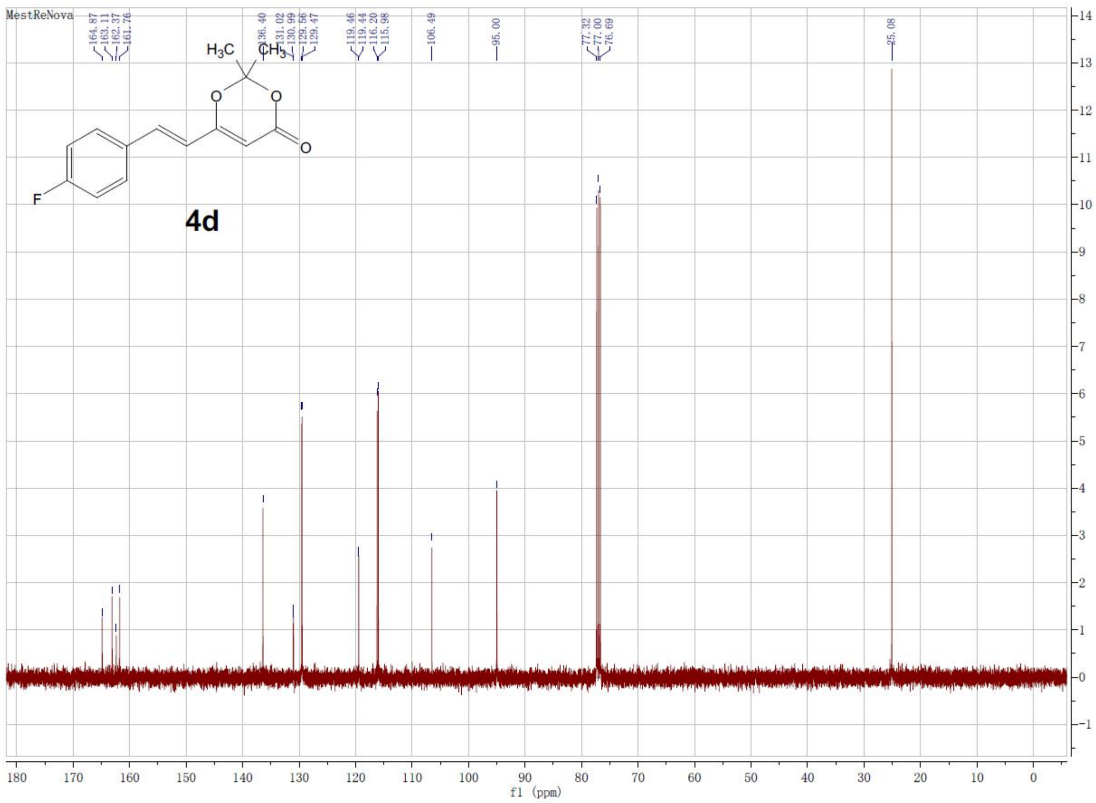

Supplementary Figure 77. ^{1}H NMR spectrum for compound **4d**

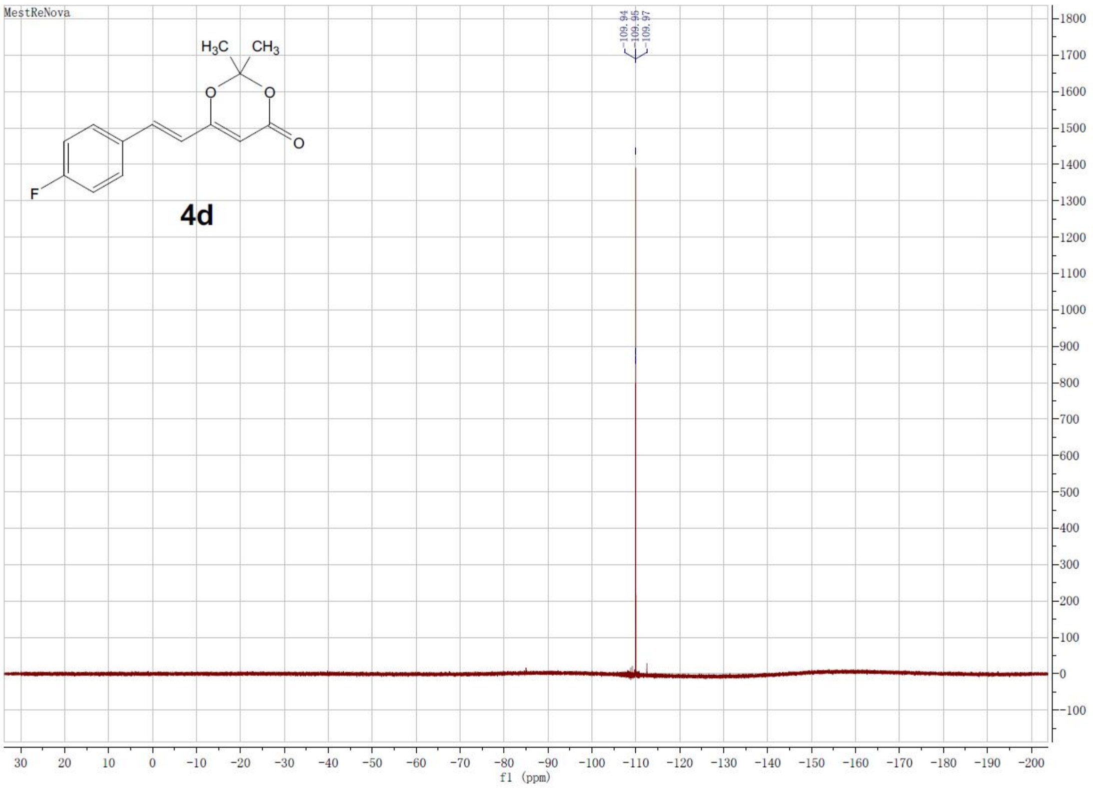

Supplementary Figure 78. ^{19}F NMR spectrum for compound **4d**

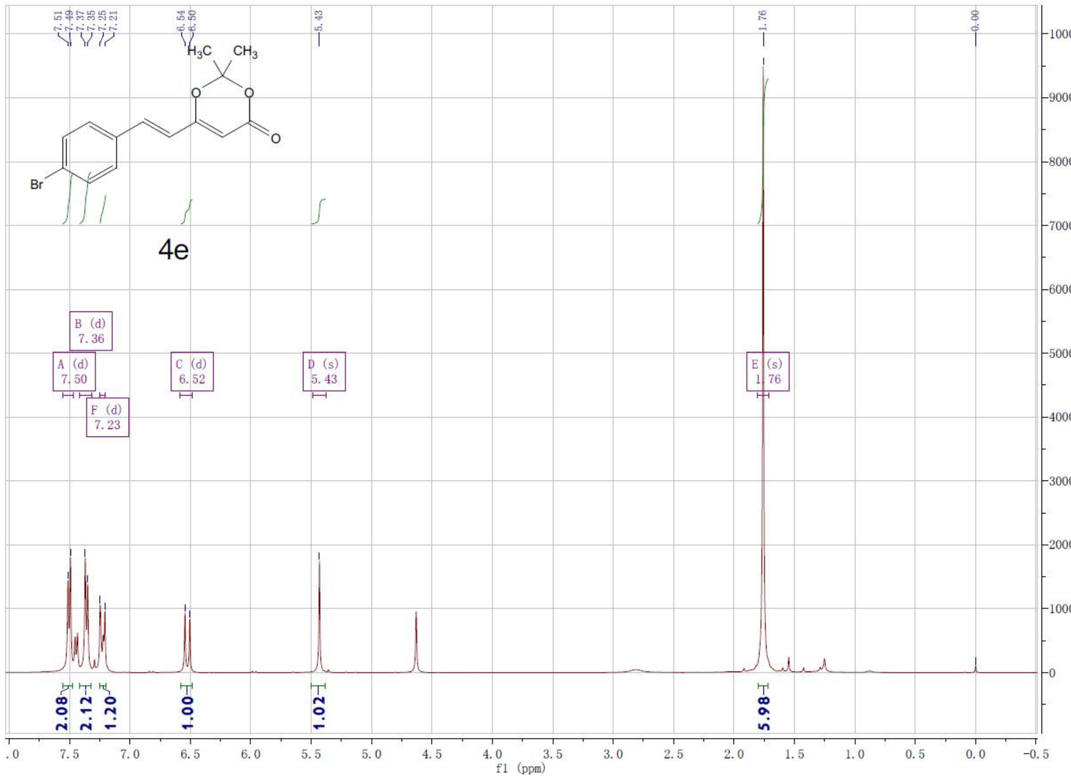

Supplementary Figure 79. ^{1}H NMR spectrum for compound **4e**

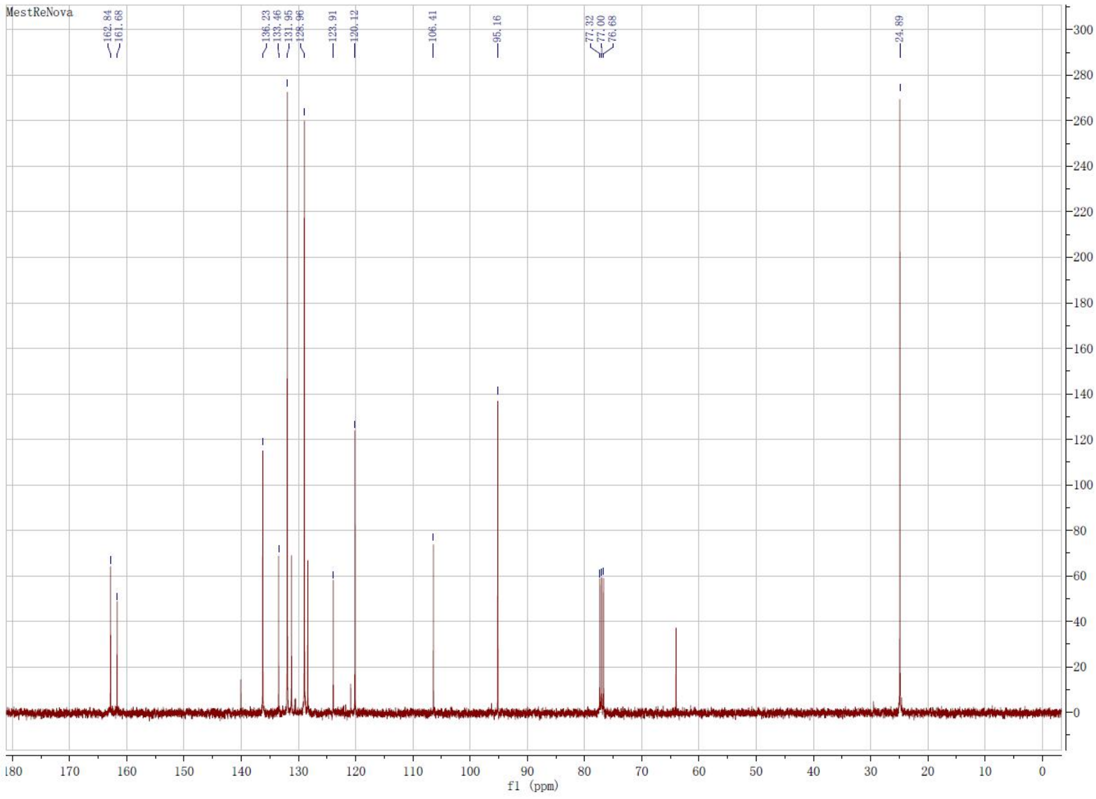

Supplementary Figure 80. ^{13}C NMR spectrum for compound **4e**

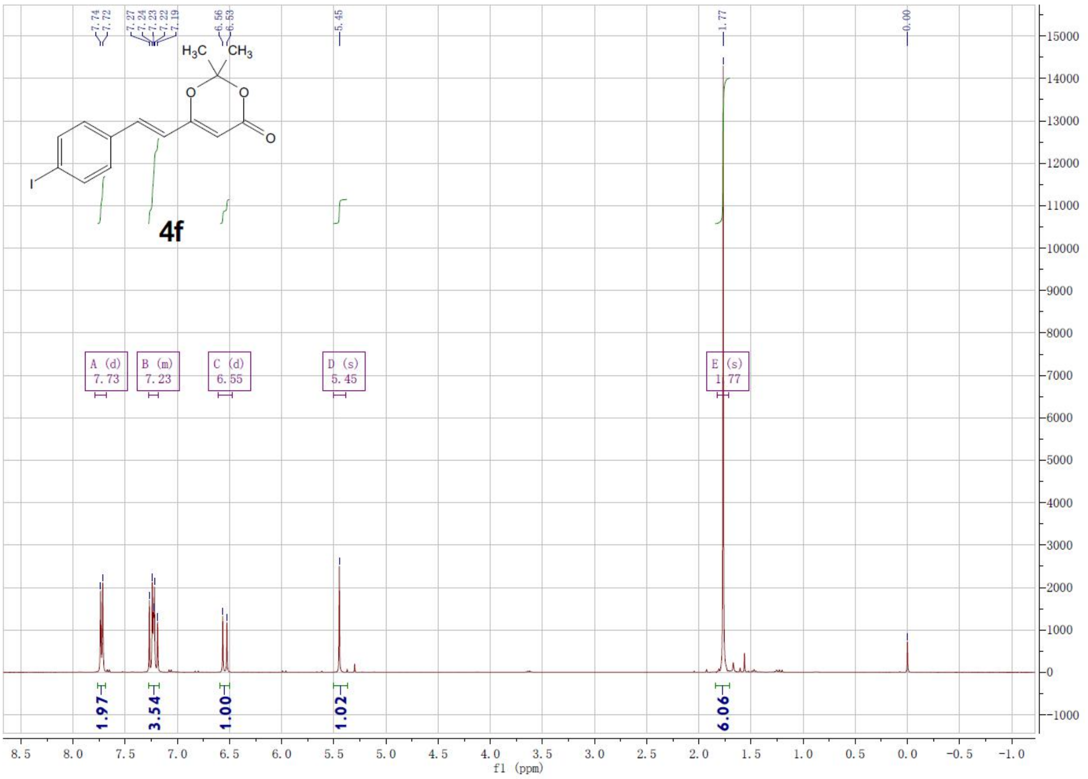

Supplementary Figure 81. ^{1}H NMR spectrum for compound **4f**

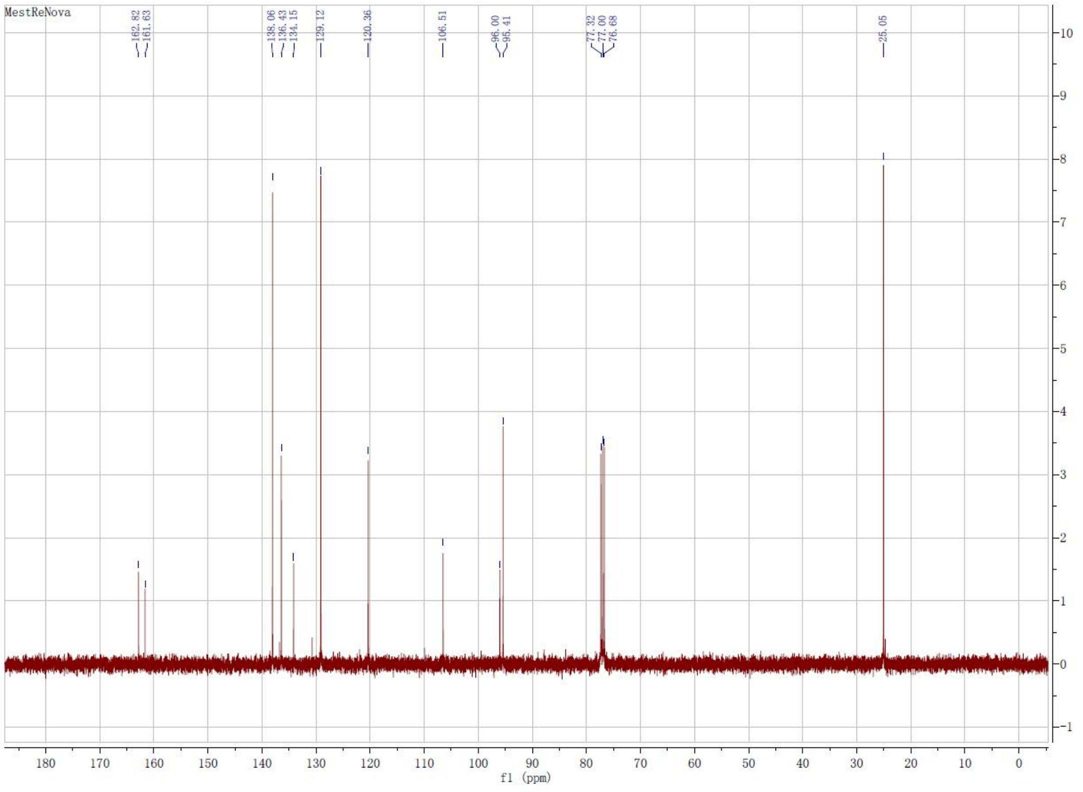

Supplementary Figure 82. ^{13}C NMR spectrum for compound **4f**

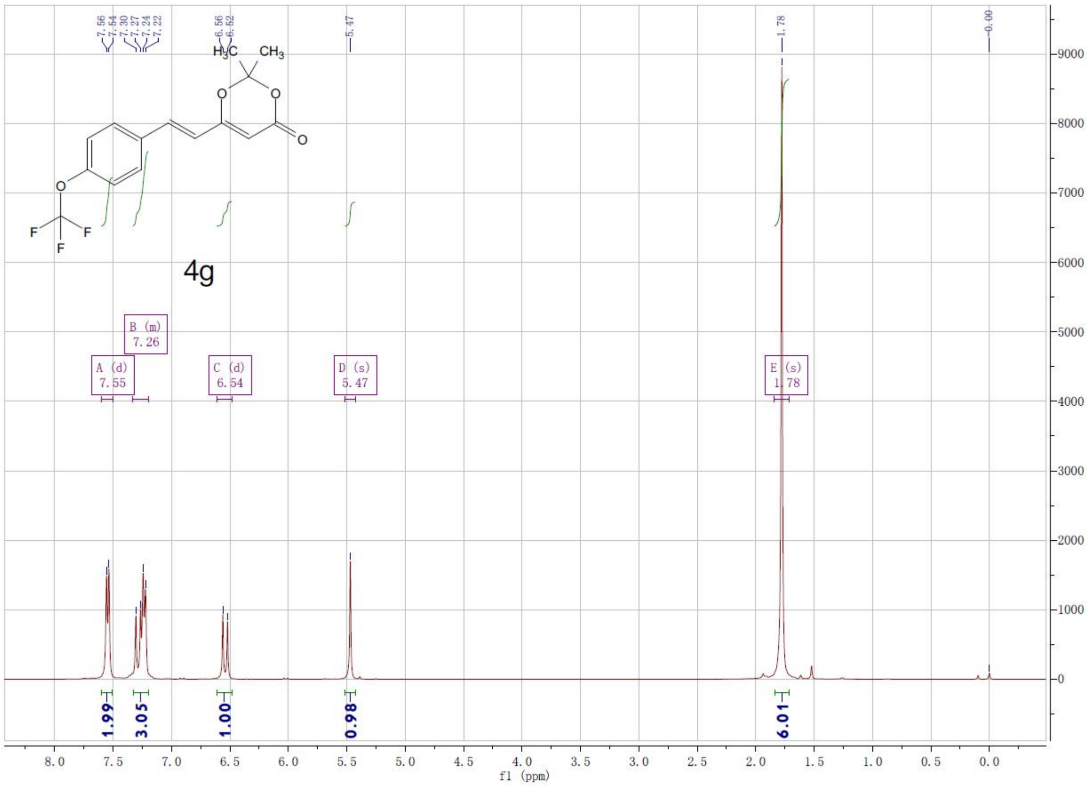

Supplementary Figure 83. ^{1}H NMR spectrum for compound **4g**

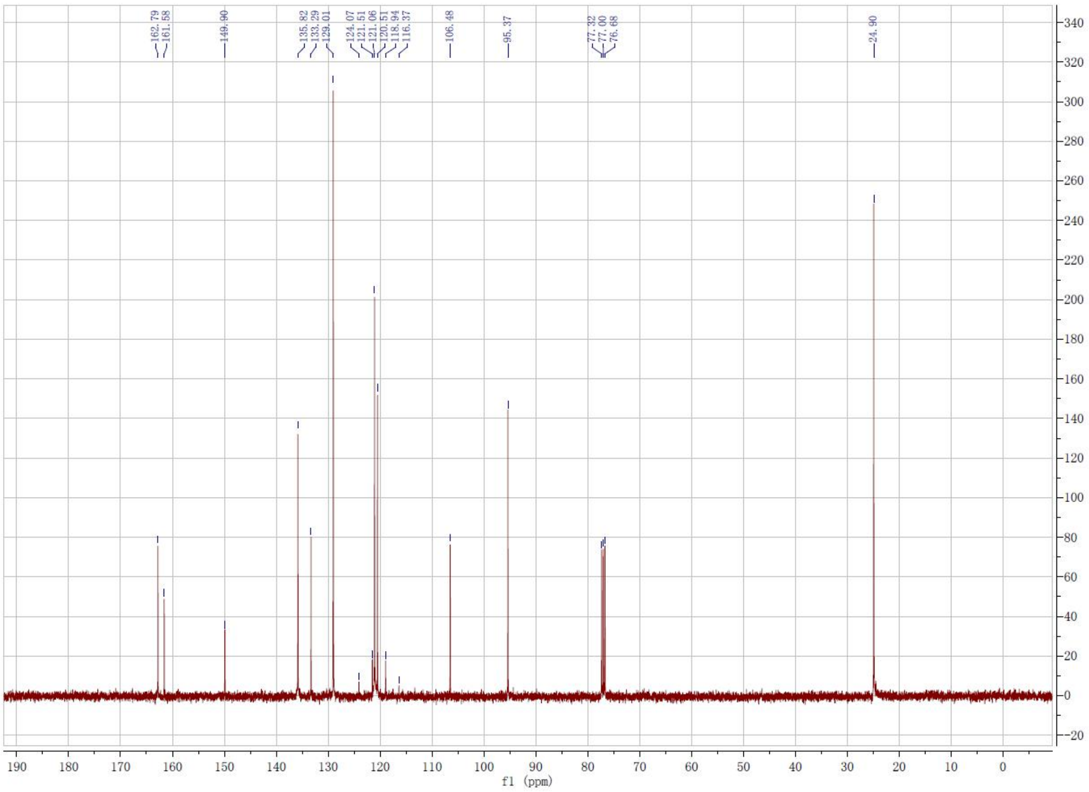

Supplementary Figure 84. ^{13}C NMR spectrum for compound **4g**

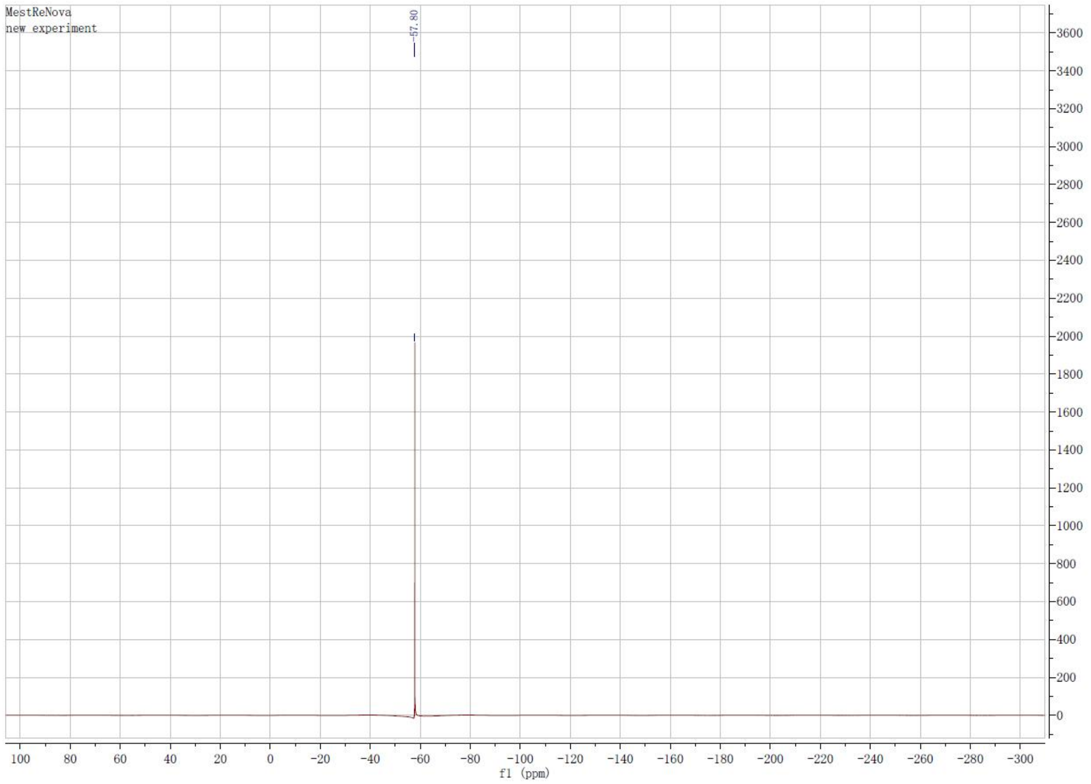

Supplementary Figure 85. ^{19}F NMR spectrum for compound **4g**

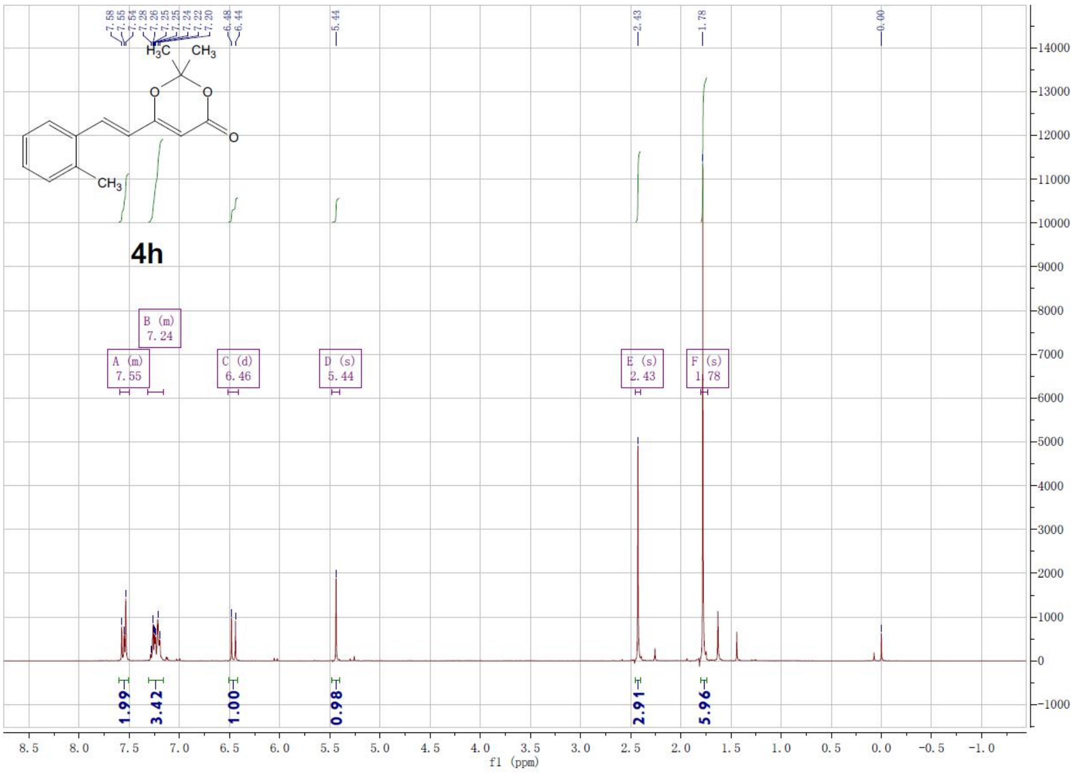

Supplementary Figure 86. ^{1}H NMR spectrum for compound **4h**

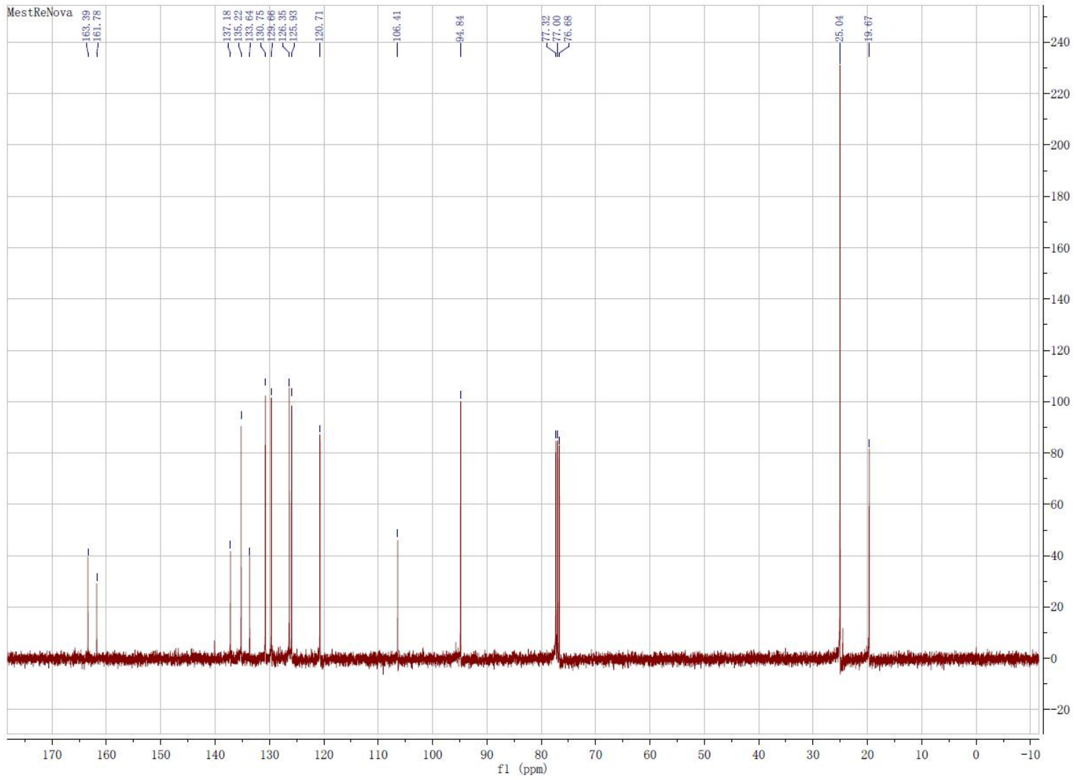

Supplementary Figure 87. ^{13}C NMR spectrum for compound **4h**

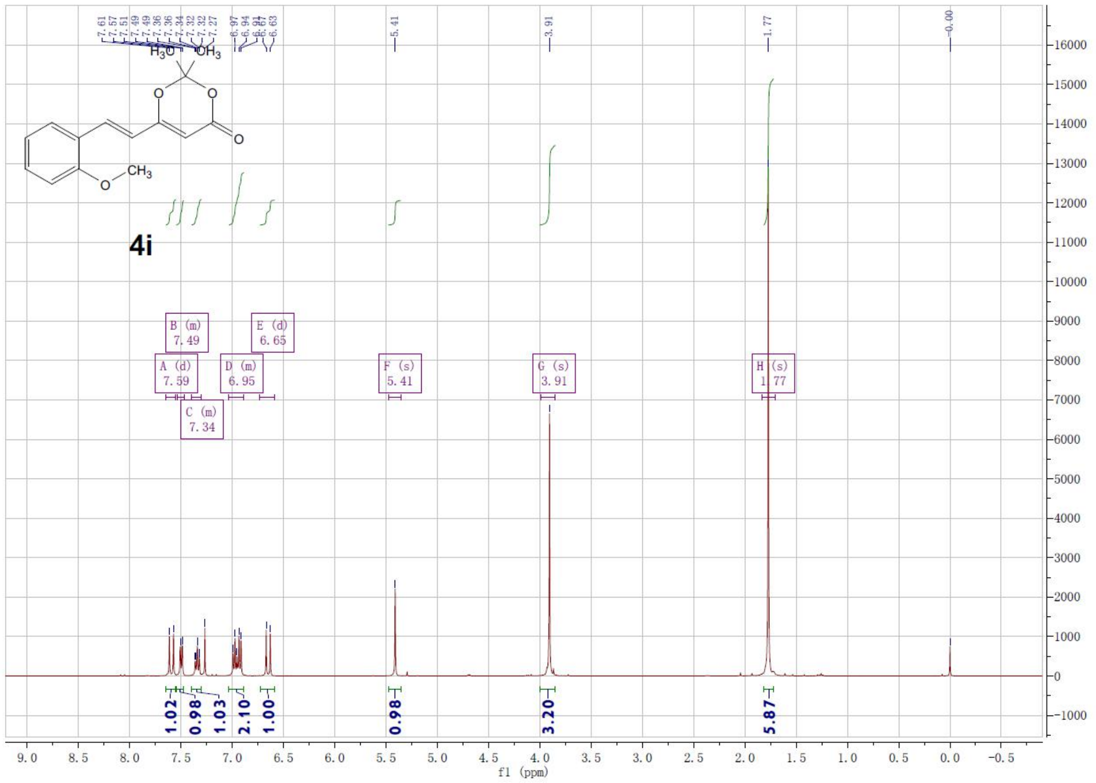

Supplementary Figure 88. ^{1}H NMR spectrum for compound **4i**

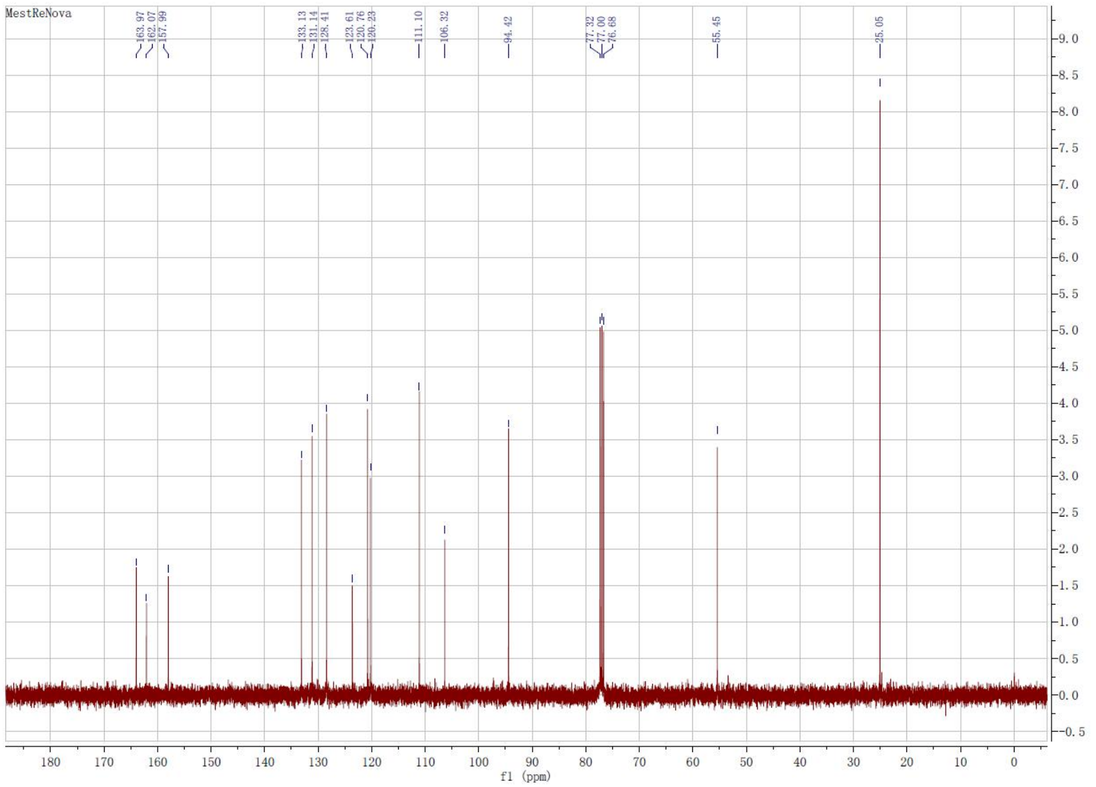

Supplementary Figure 89. ^{13}C NMR spectrum for compound **4i**

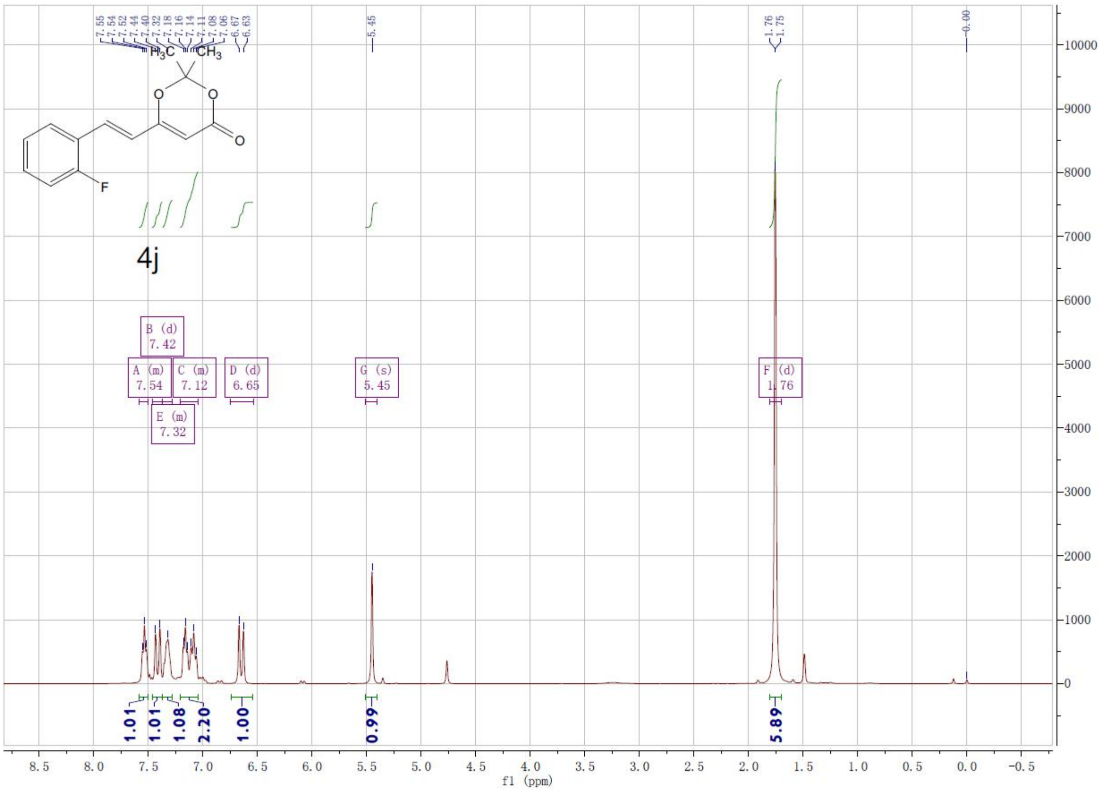

Supplementary Figure 90. ^{1}H NMR spectrum for compound **4j**

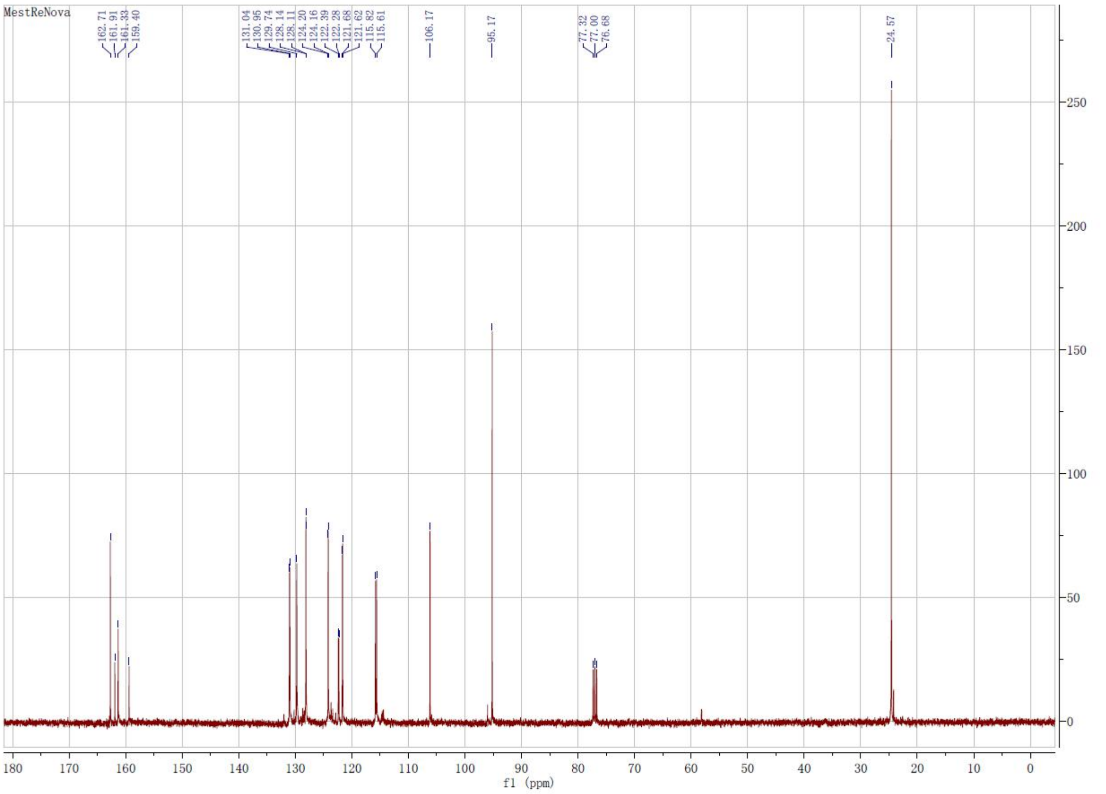

Supplementary Figure 91. ^{13}C NMR spectrum for compound **4j**

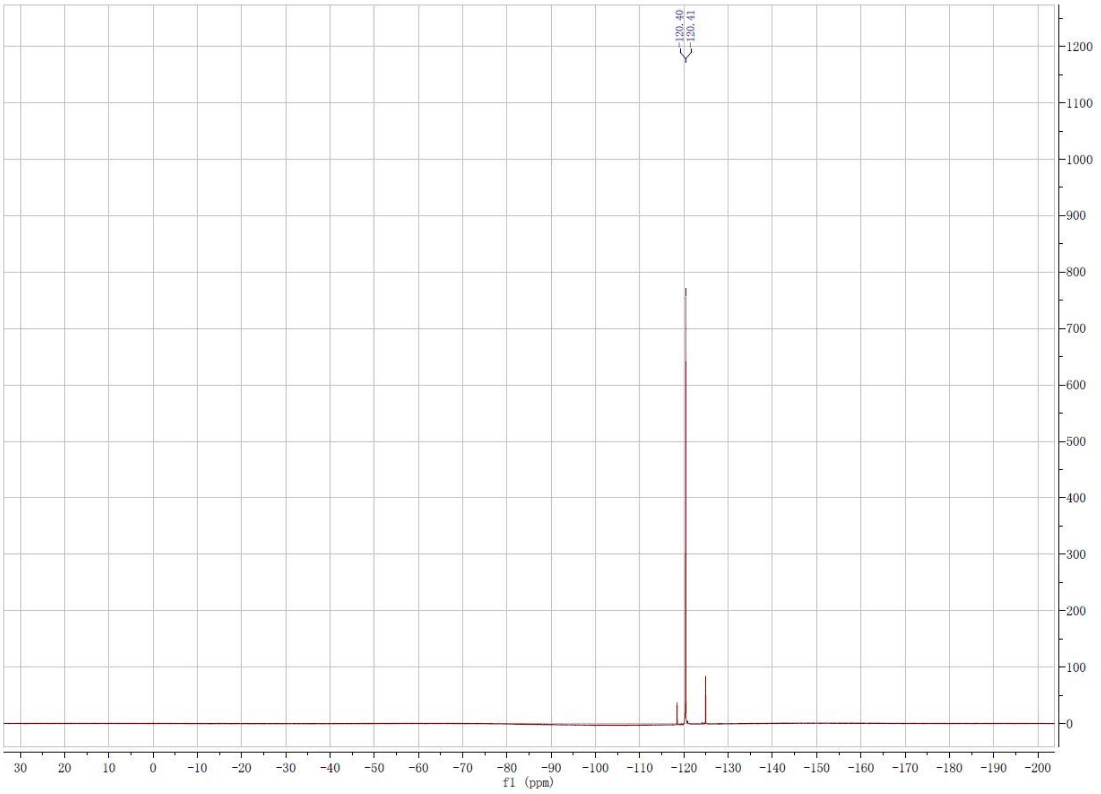

Supplementary Figure 92. ^{19}F NMR spectrum for compound **4j**

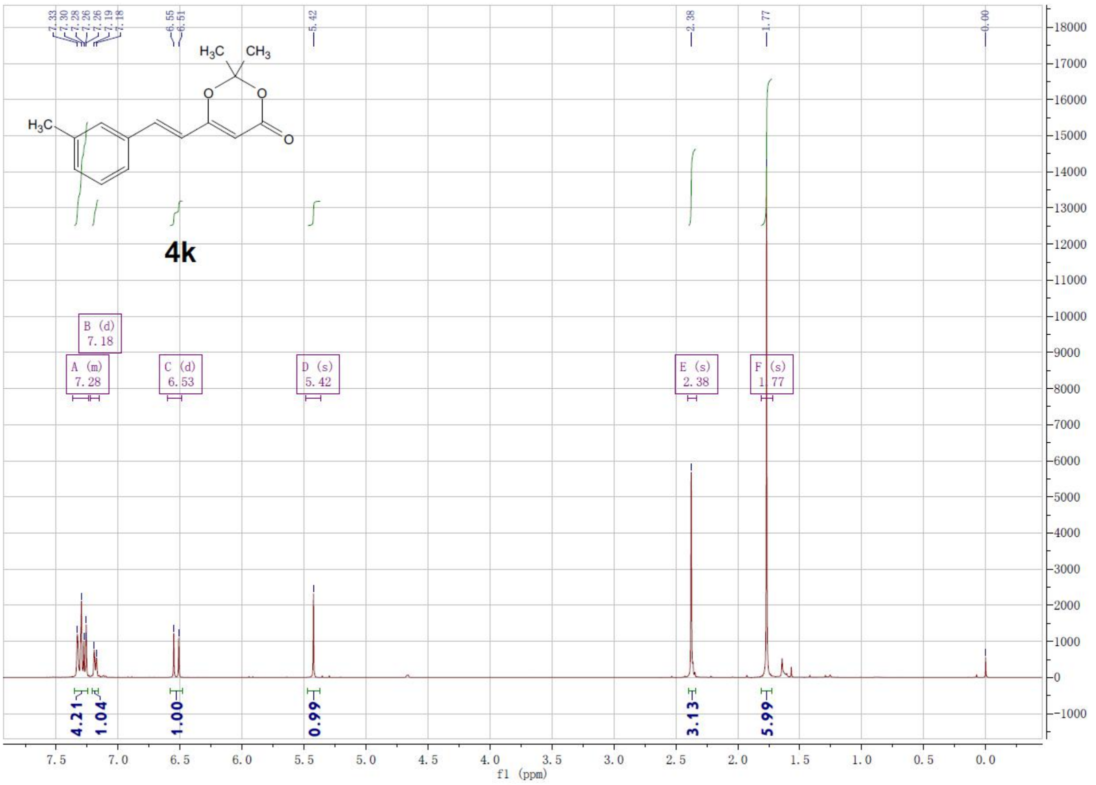

Supplementary Figure 93. ^{1}H NMR spectrum for compound **4k**

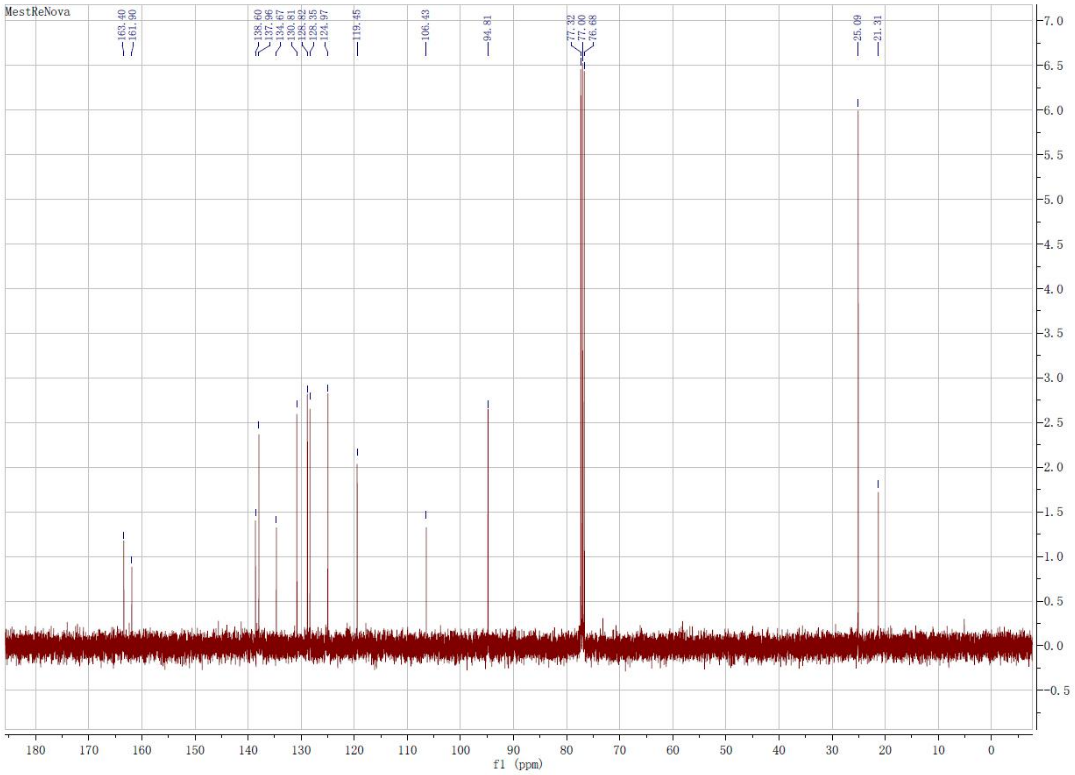

Supplementary Figure 94. ^{13}C NMR spectrum for compound **4k**

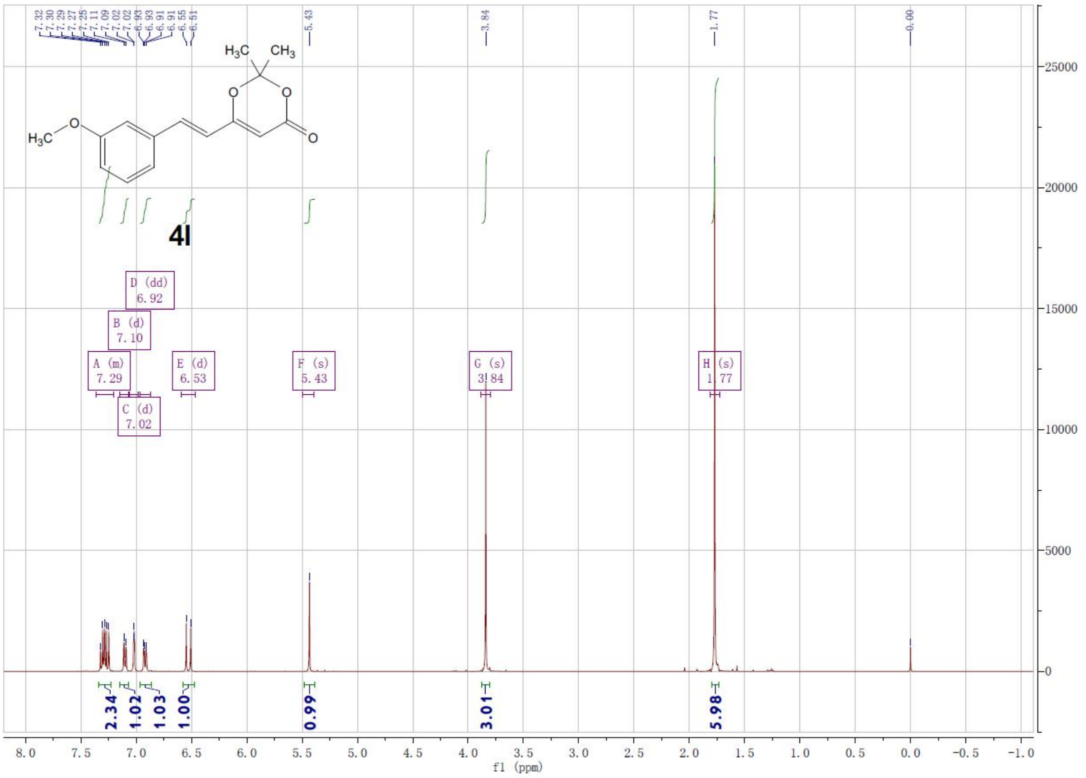

Supplementary Figure 95. ^{1}H NMR spectrum for compound **4l**

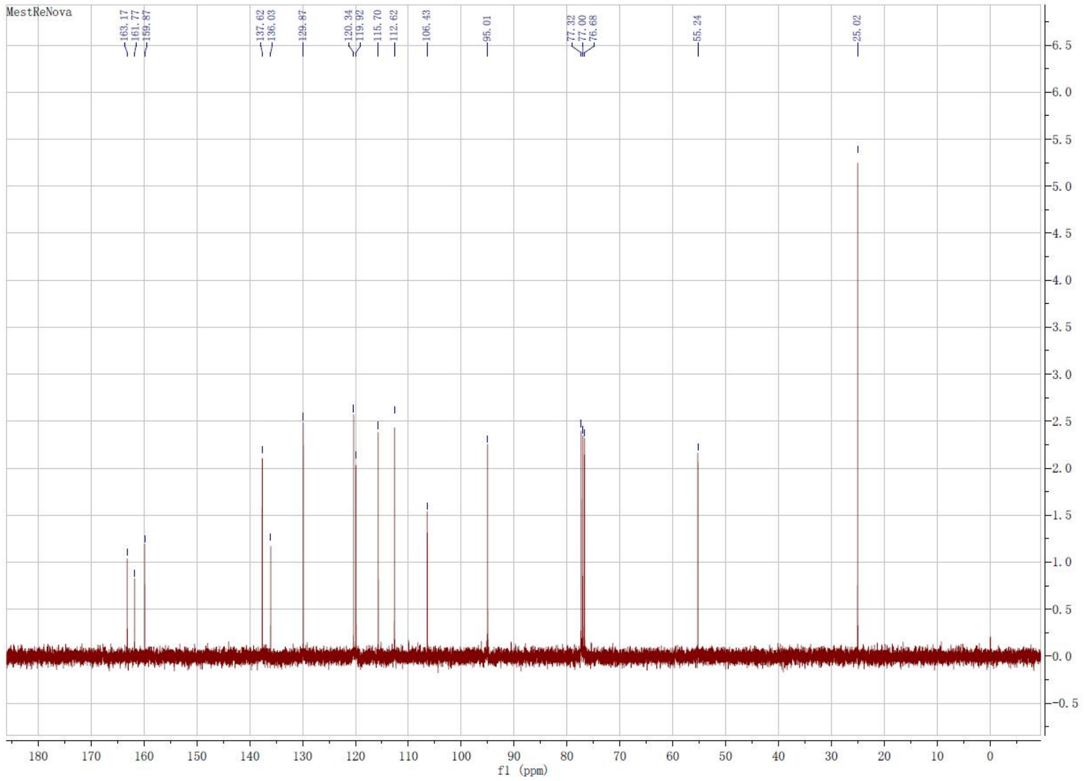

Supplementary Figure 96. ^{13}C NMR spectrum for compound **4l**

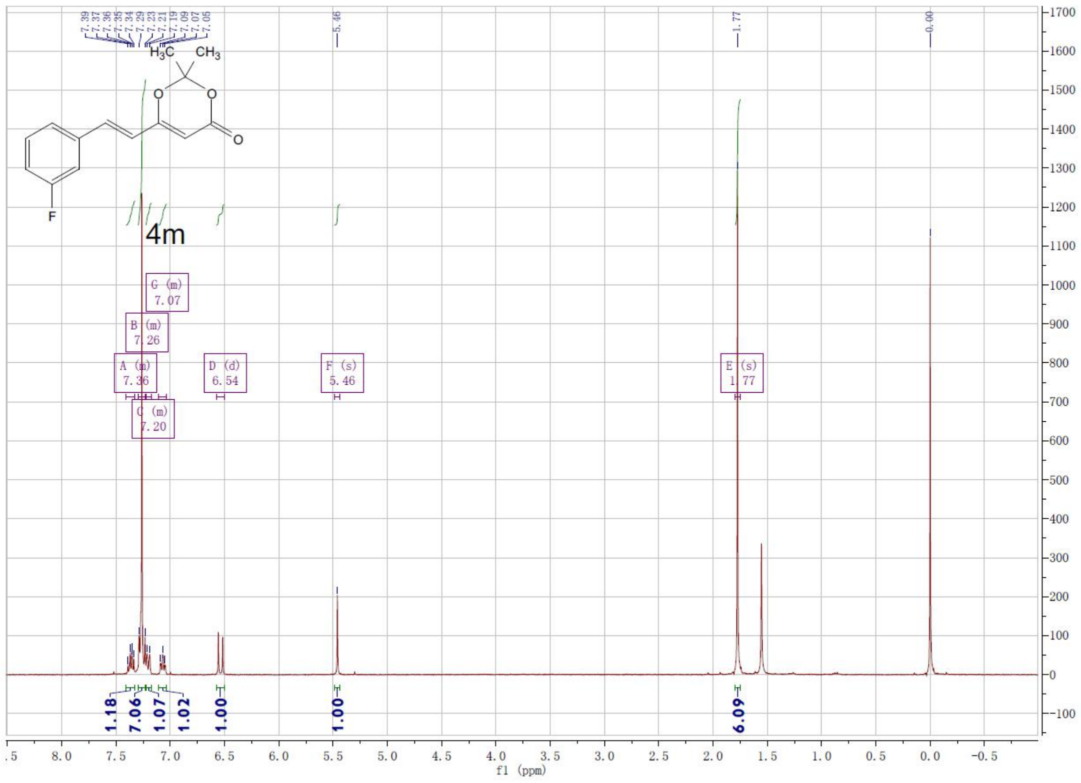

Supplementary Figure 97. ^{1}H NMR spectrum for compound **4m**

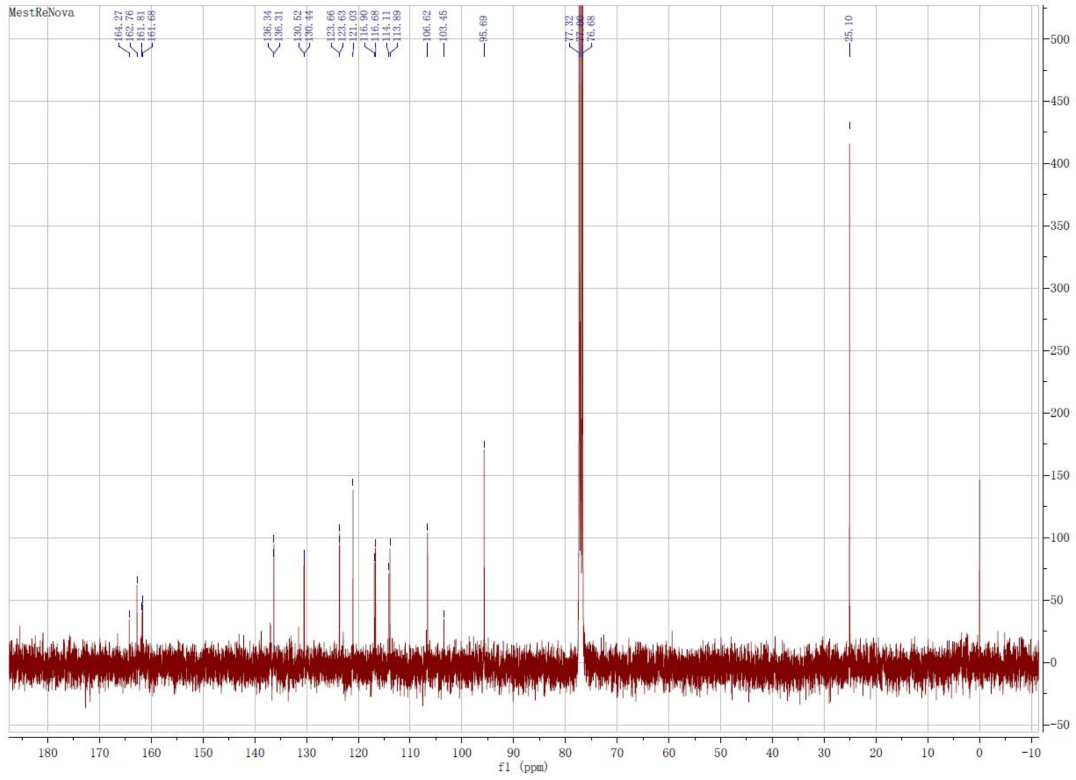

Supplementary Figure 98. ^{13}C NMR spectrum for compound **4m**

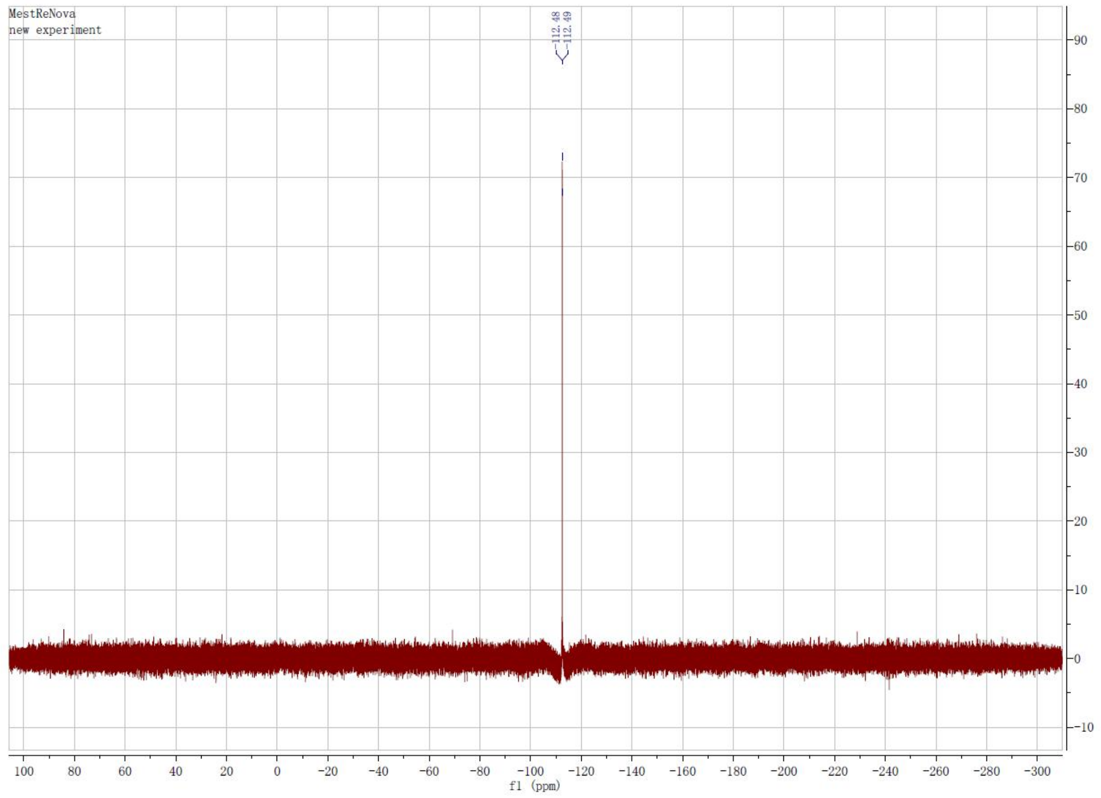

Supplementary Figure 99. ^{19}F NMR spectrum for compound **4m**

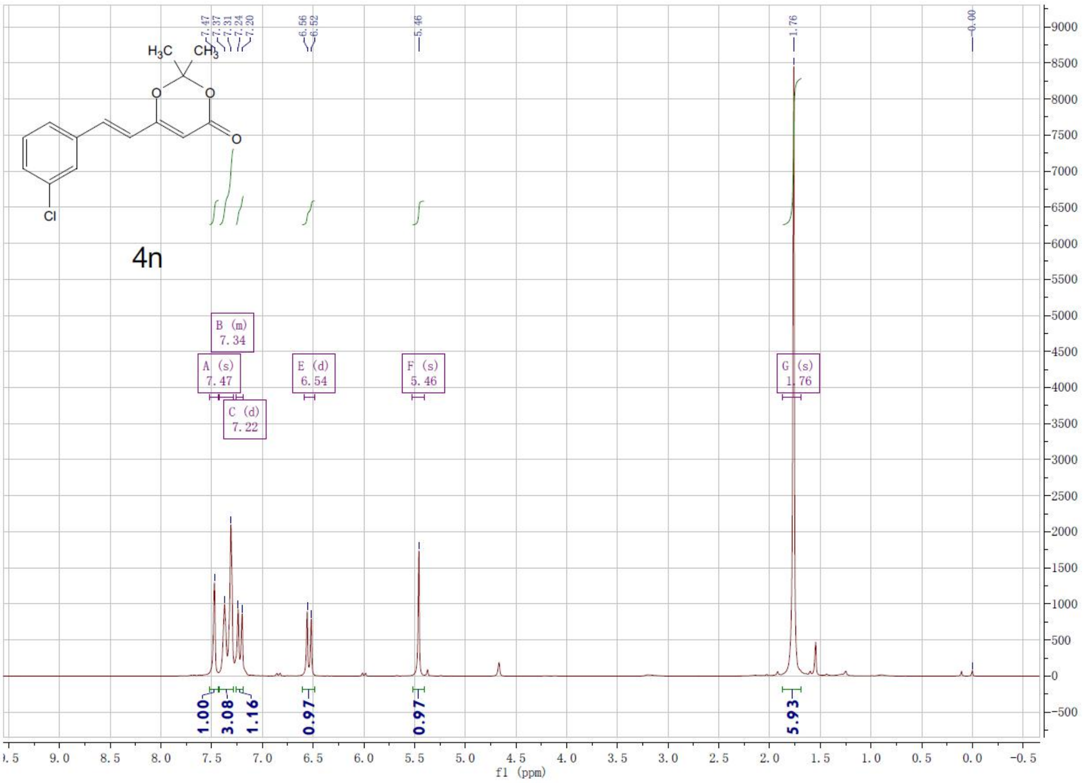

Supplementary Figure 100. ^{1}H NMR spectrum for compound **4n**

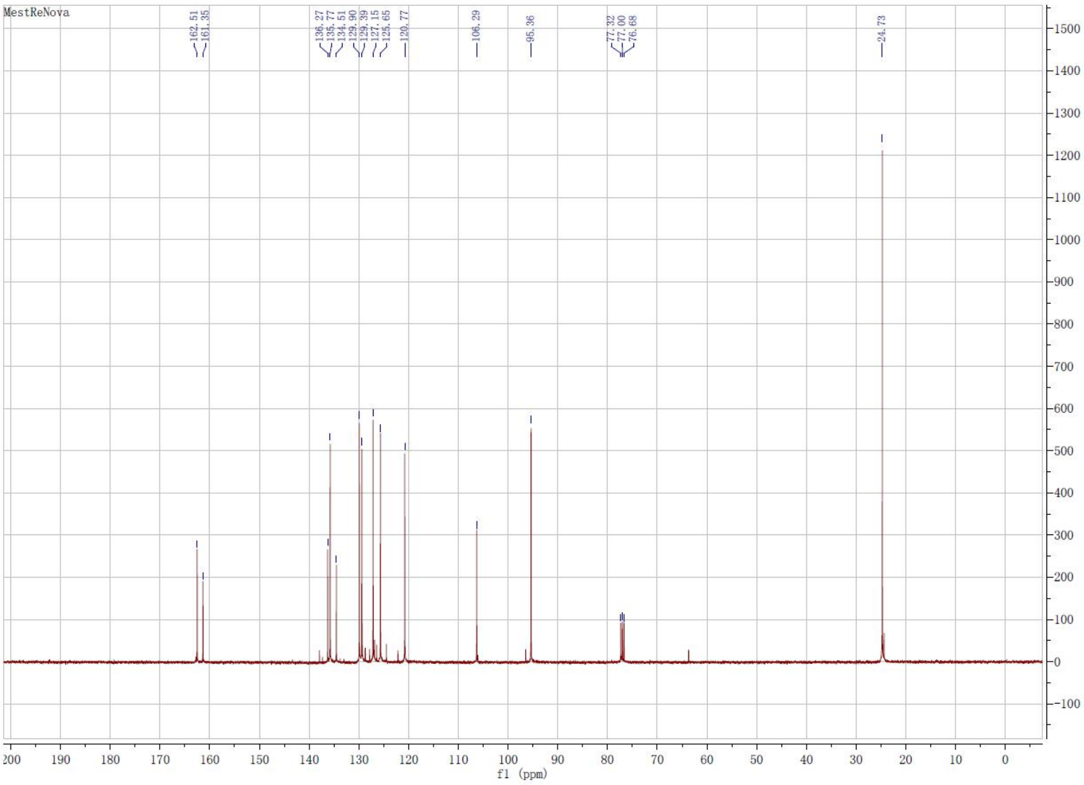

Supplementary Figure 101. ^{13}C NMR spectrum for compound **4n**

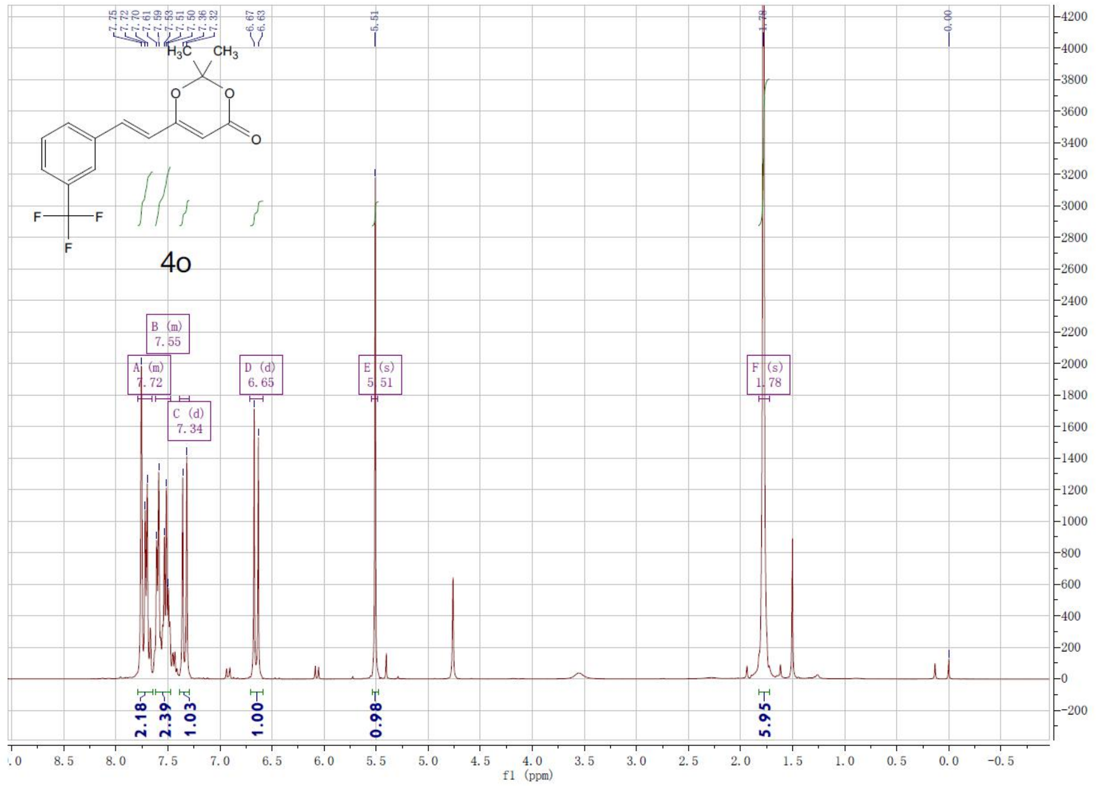

Supplementary Figure 102. ^{1}H NMR spectrum for compound **4o**

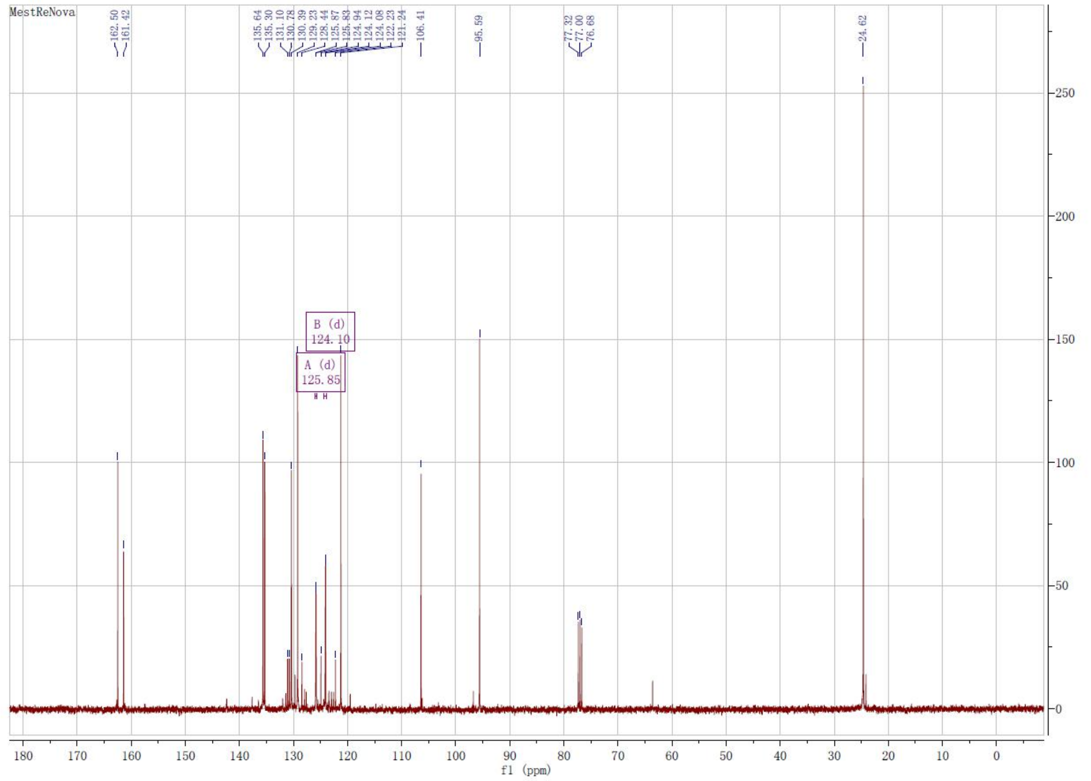

Supplementary Figure 103. ^{13}C NMR spectrum for compound **4o**

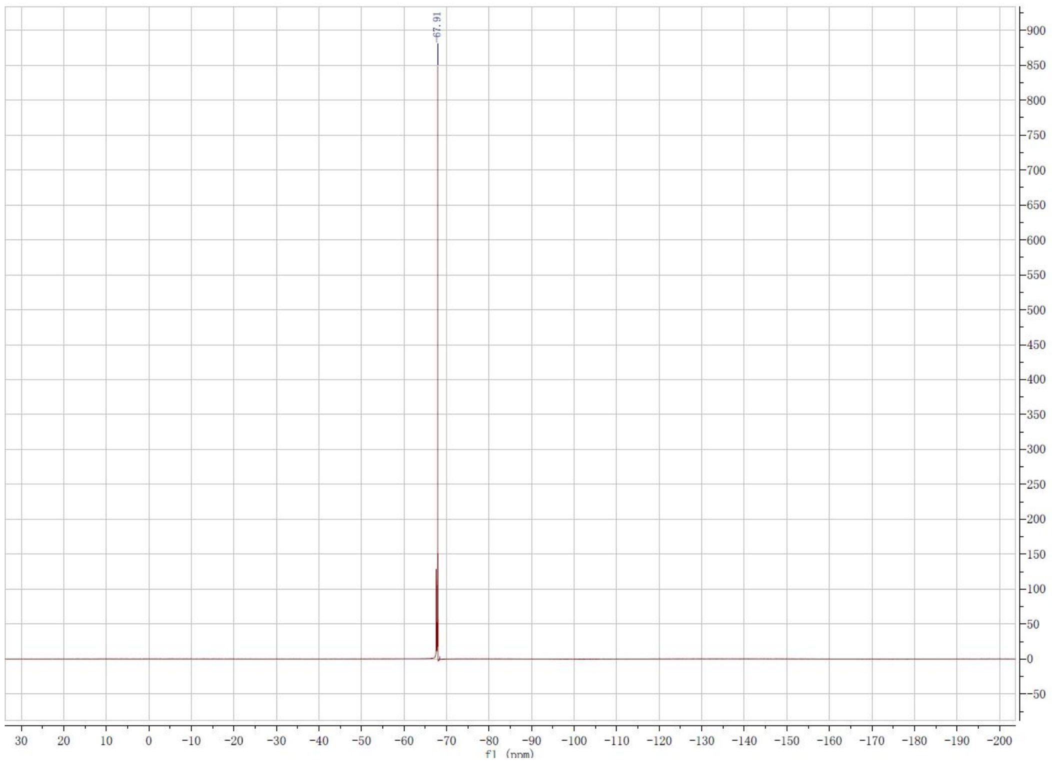

Supplementary Figure 104. ^{19}F NMR spectrum for compound **4o**

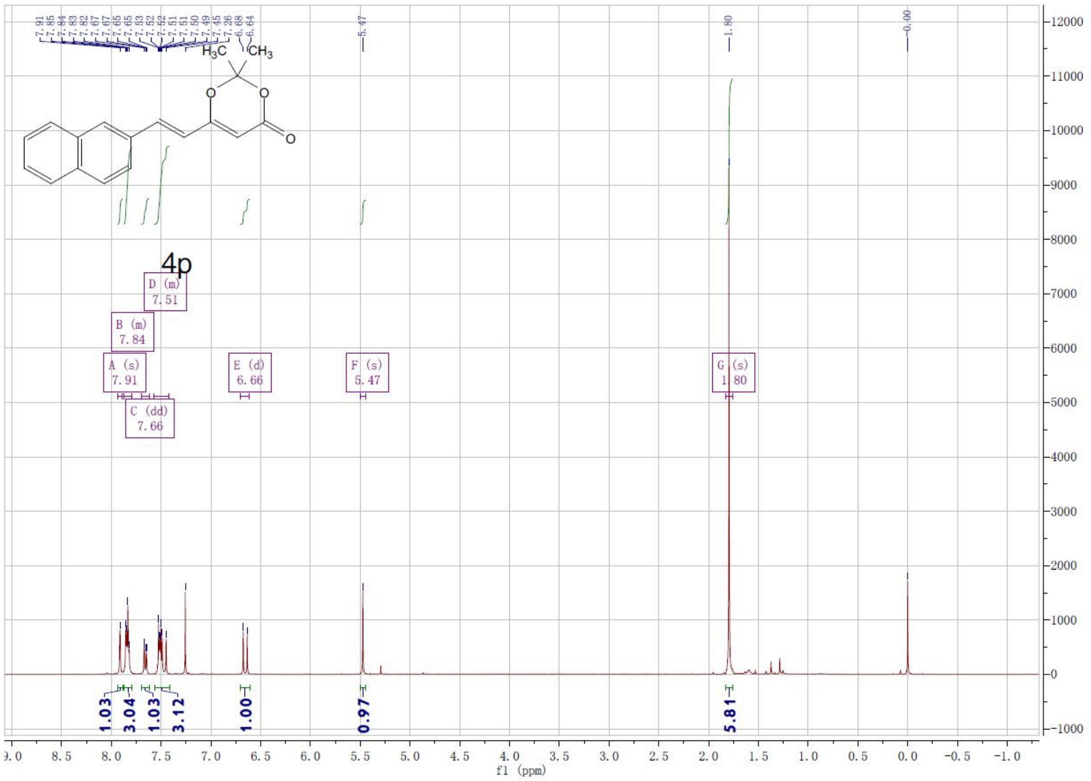

Supplementary Figure 105. ^{1}H NMR spectrum for compound **4p**

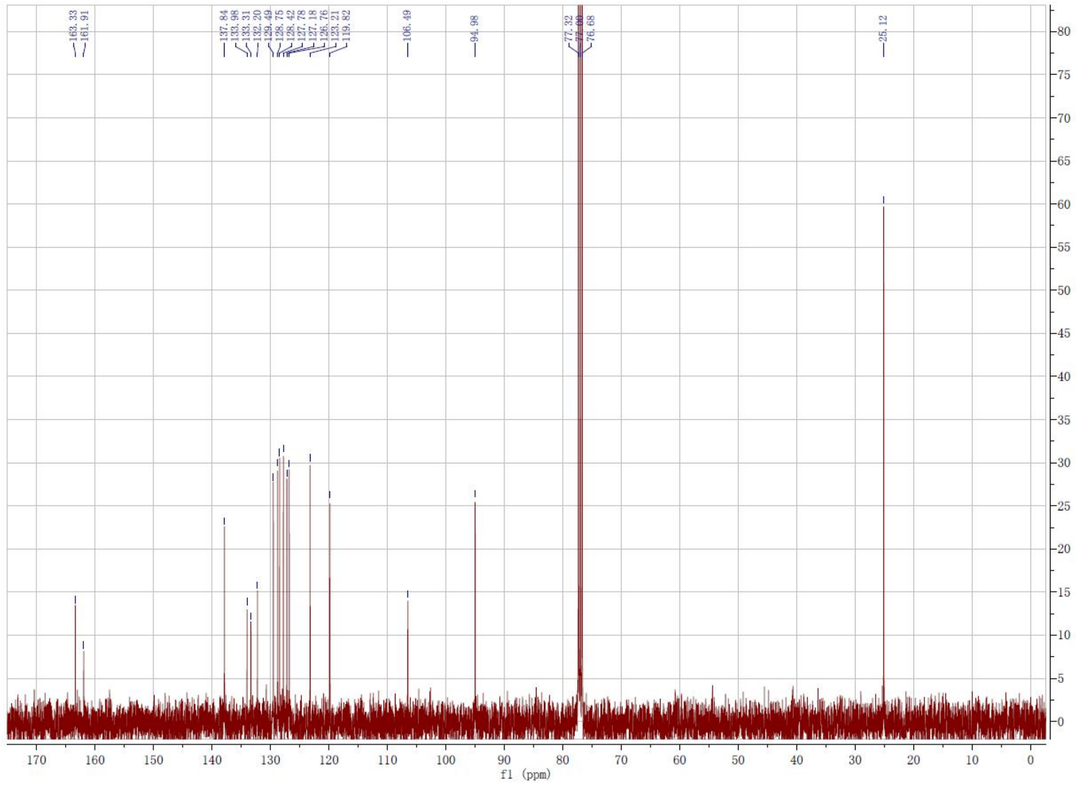

Supplementary Figure 106. ^{13}C NMR spectrum for compound **4p**

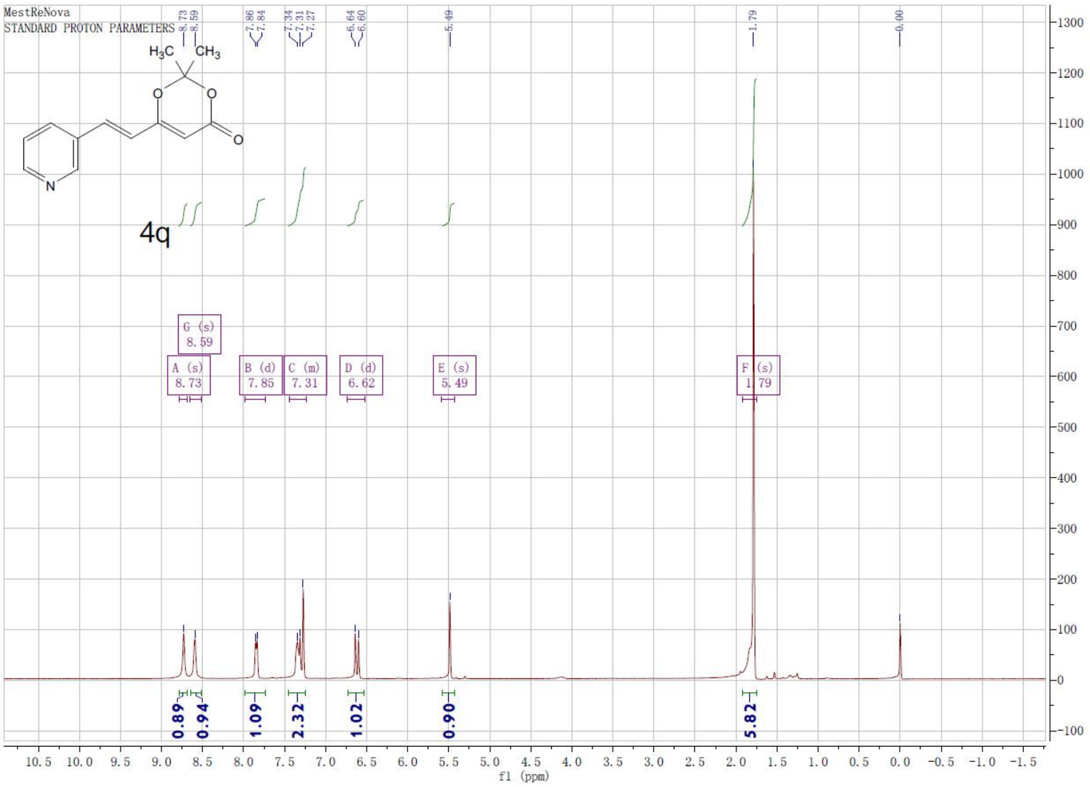

Supplementary Figure 107. ^{1}H NMR spectrum for compound **4q**

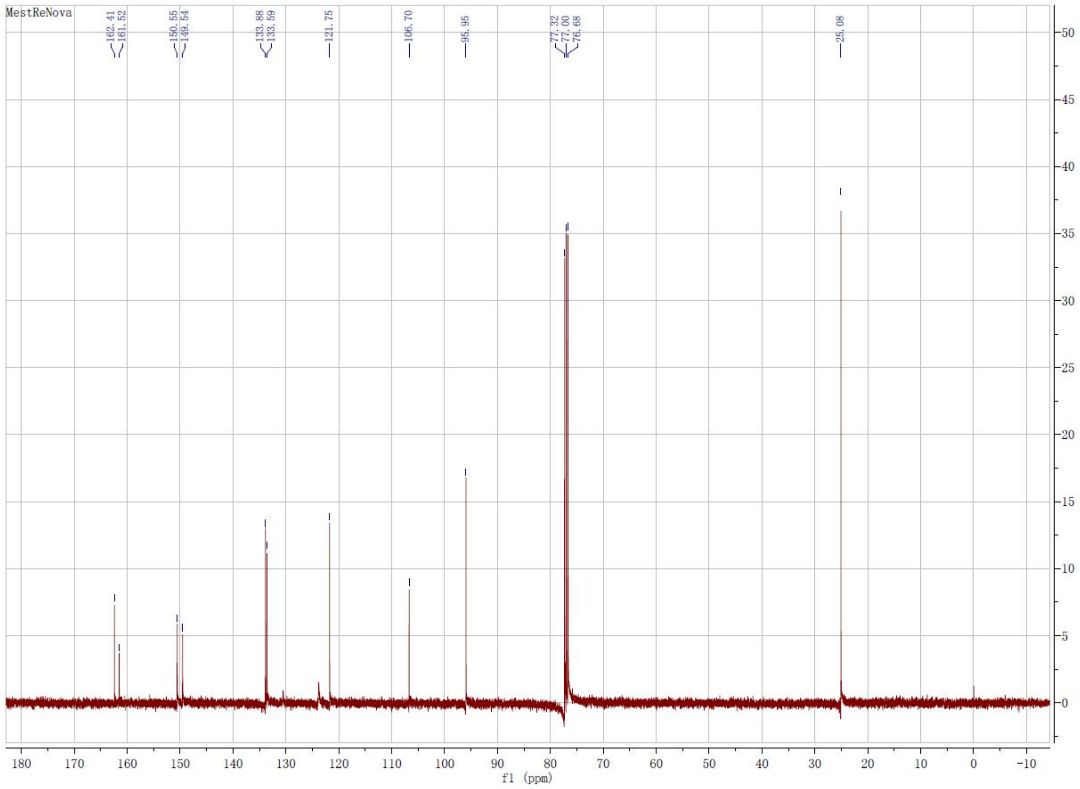

Supplementary Figure 108. ^{13}C NMR spectrum for compound **4q**

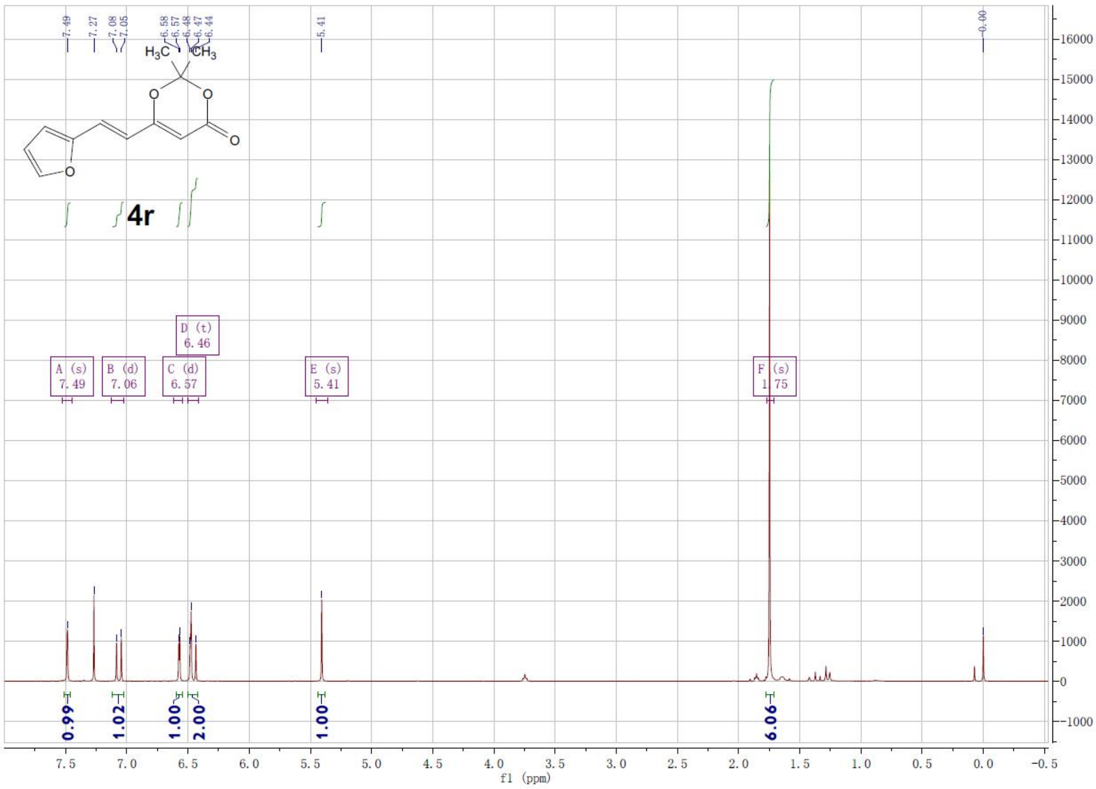

Supplementary Figure 109. ^{1}H NMR spectrum for compound **4r**

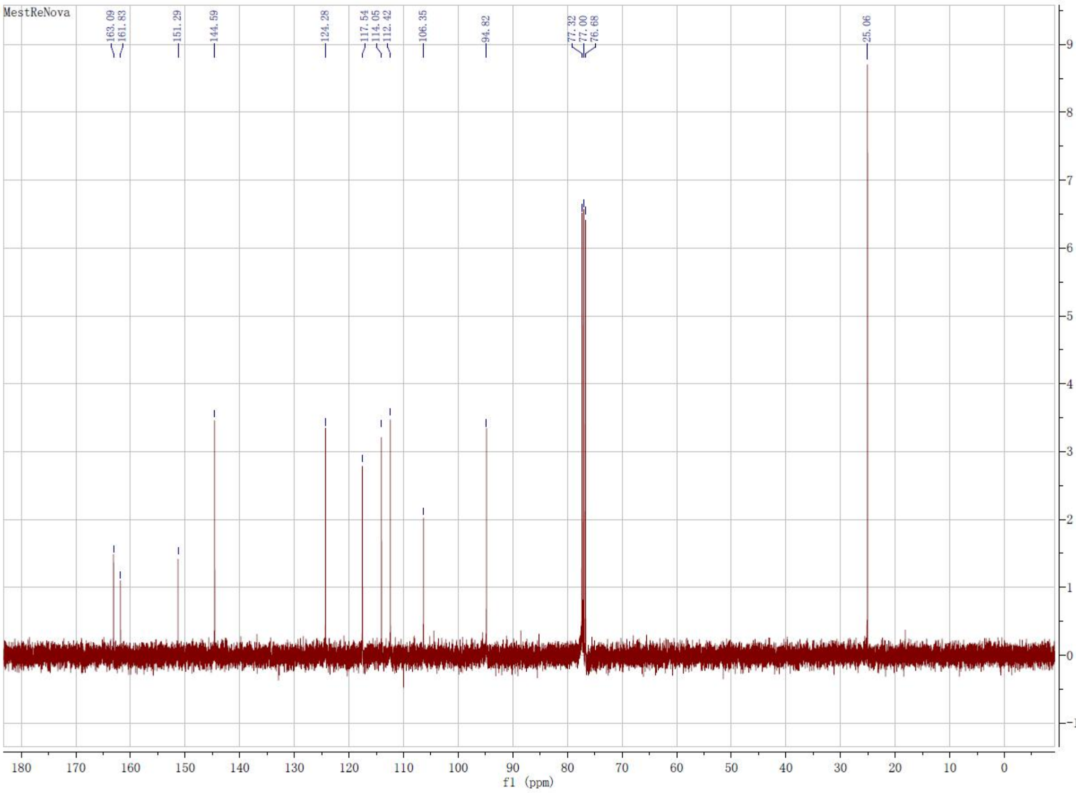

Supplementary Figure 110. ^{13}C NMR spectrum for compound **4r**

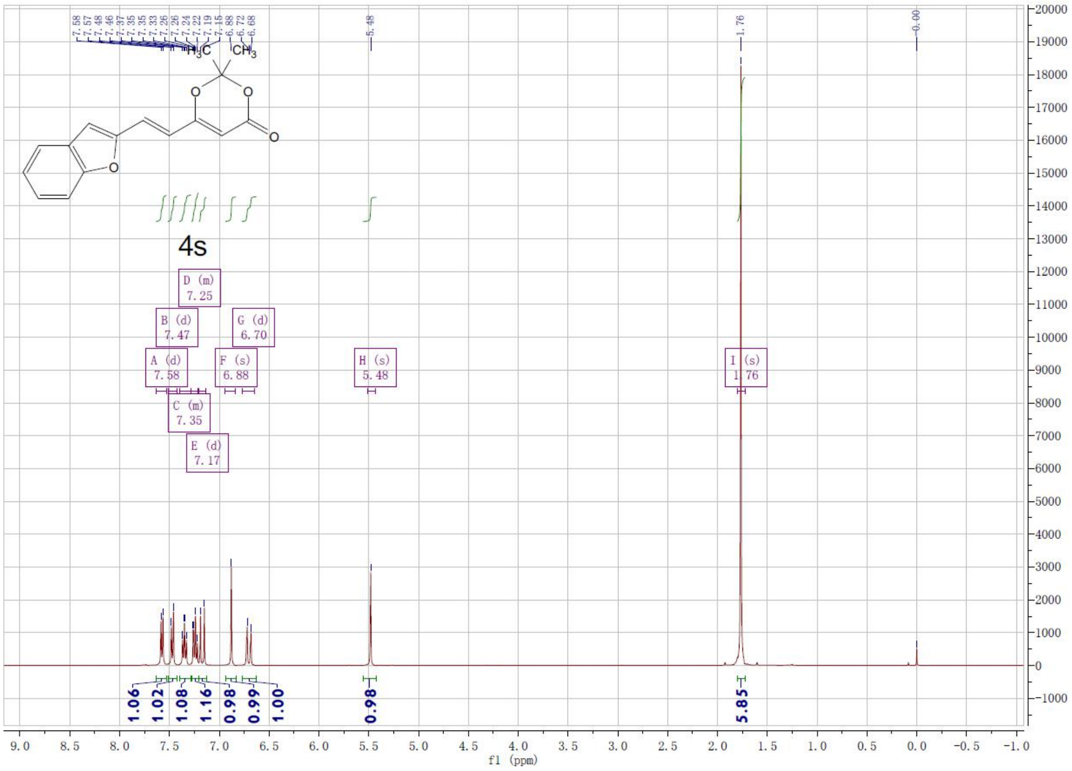

Supplementary Figure 111. ^{1}H NMR spectrum for compound **4s**

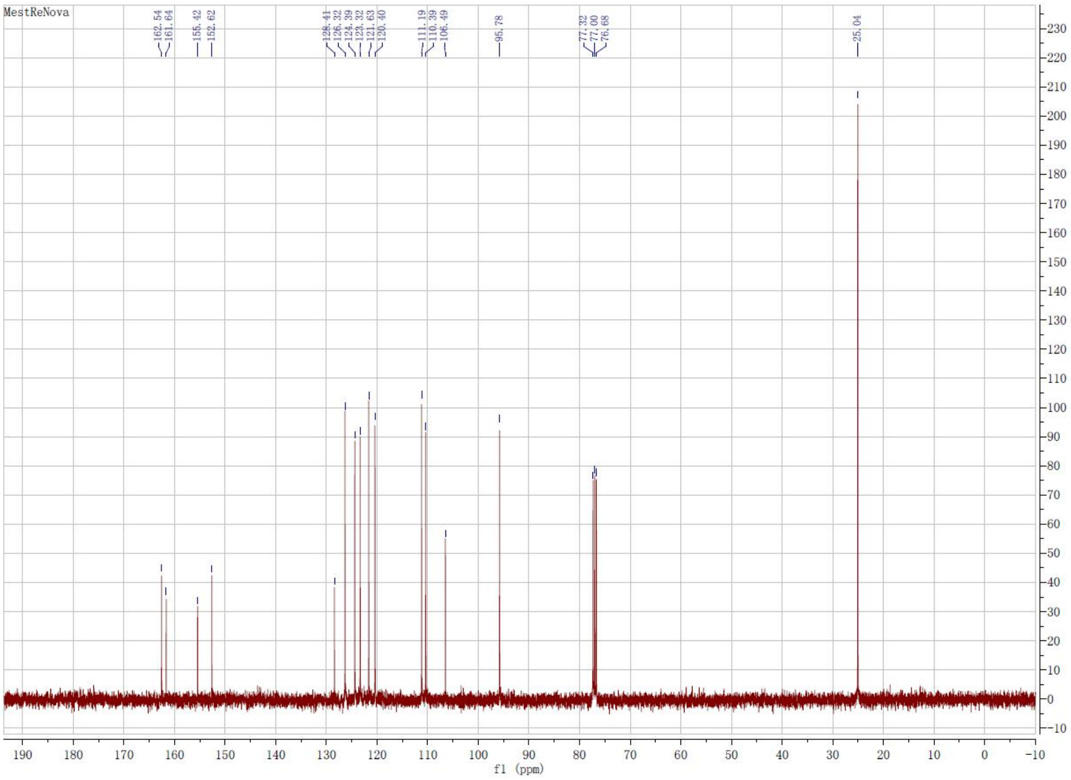

Supplementary Figure 112. ^{13}C NMR spectrum for compound **4s**

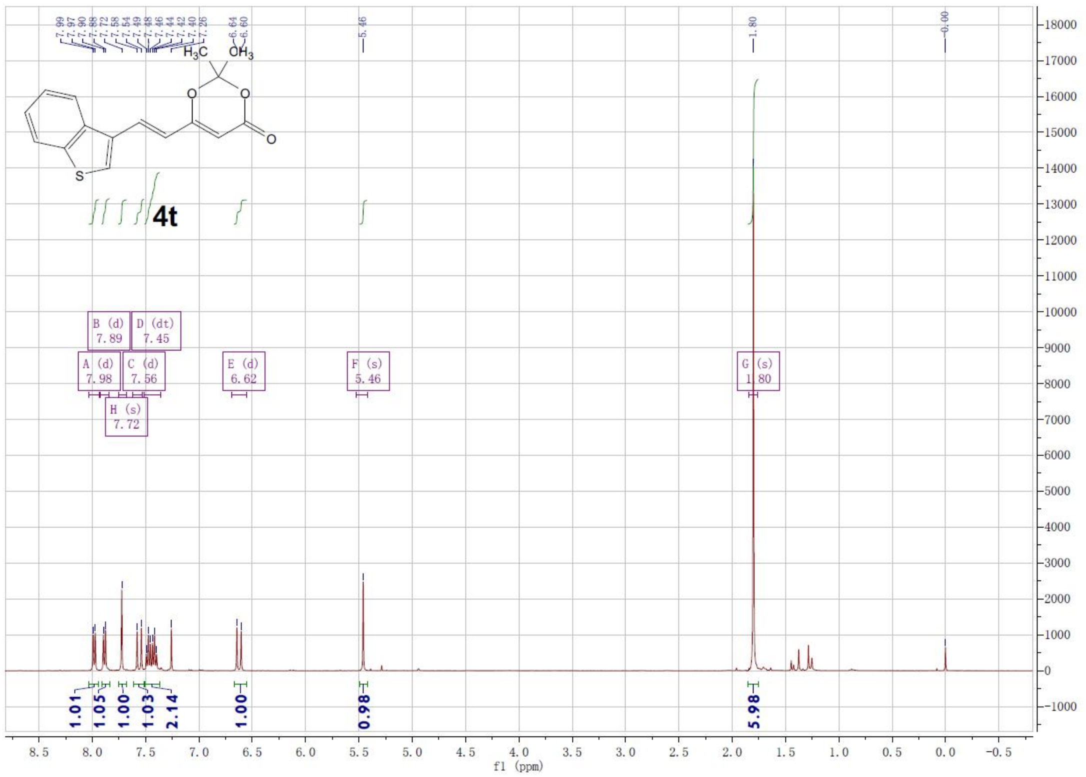

Supplementary Figure 113. ^{1}H NMR spectrum for compound **4t**

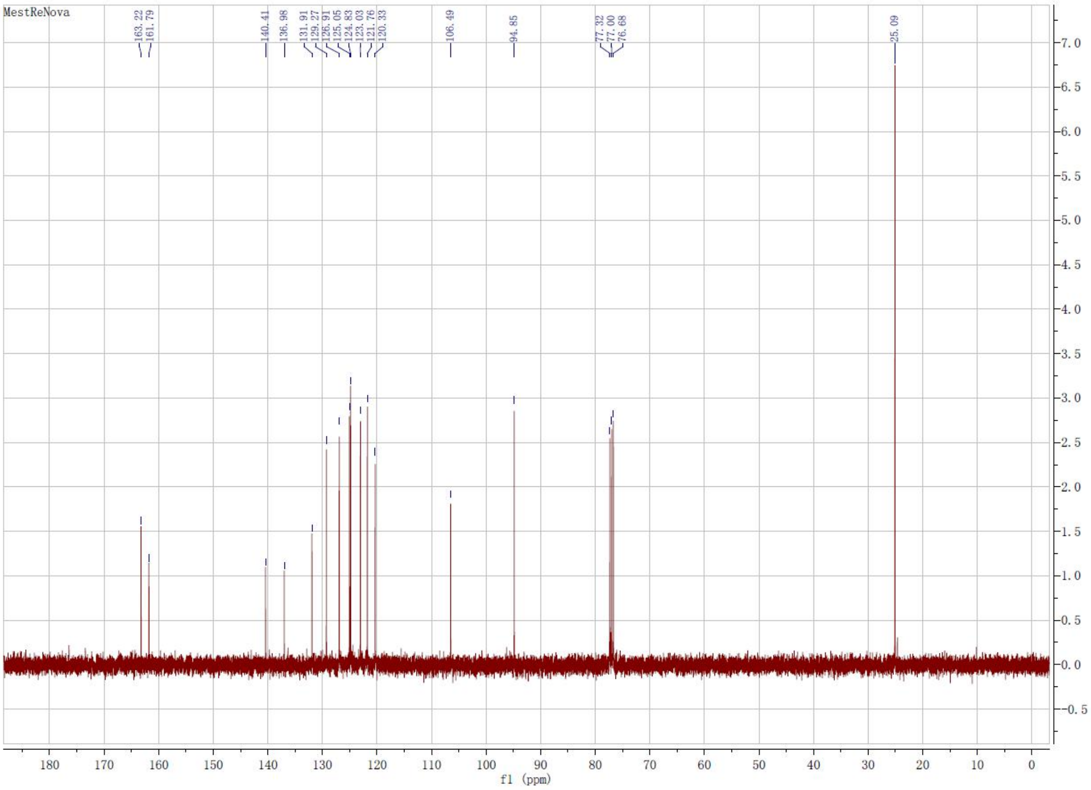

Supplementary Figure 114. ^{13}C NMR spectrum for compound **4t**

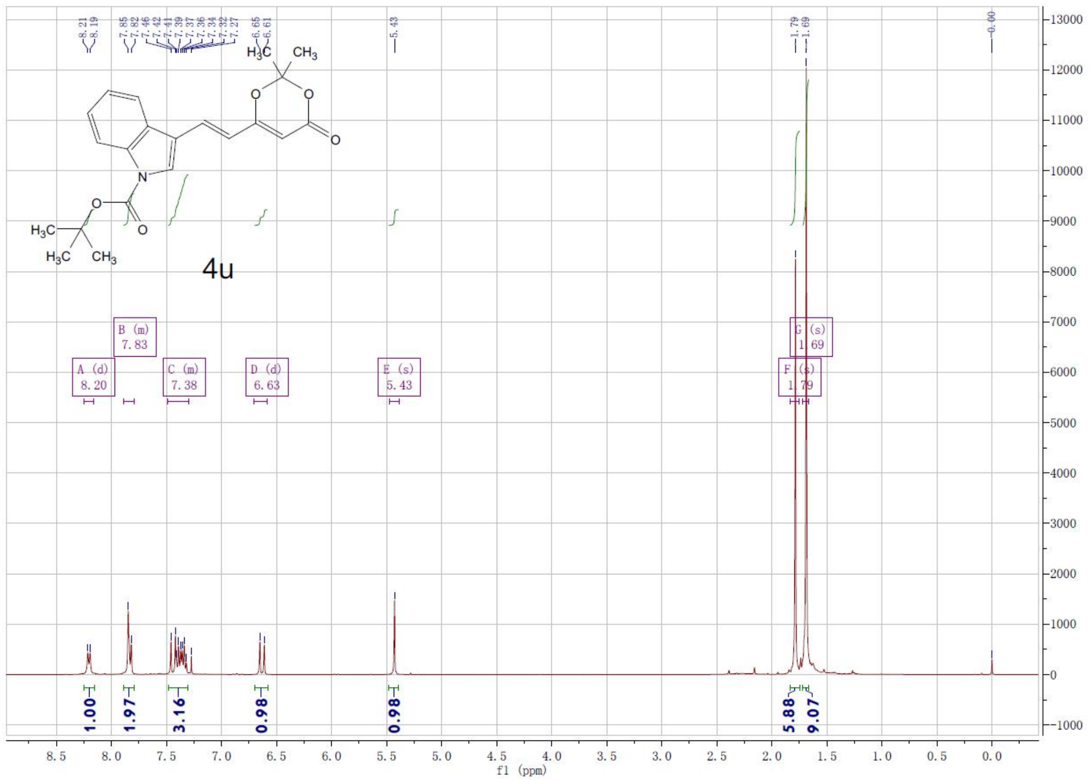

Supplementary Figure 115. ^{1}H NMR spectrum for compound **4u**

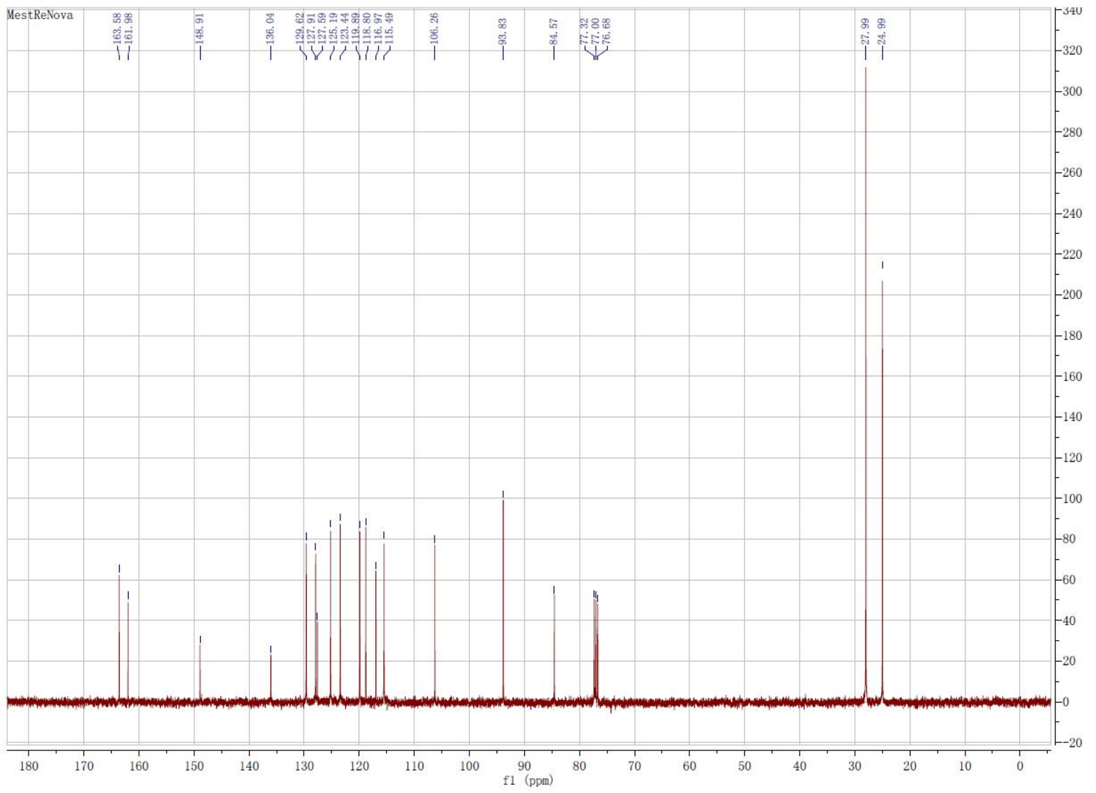

Supplementary Figure 116. ^{13}C NMR spectrum for compound **4u**

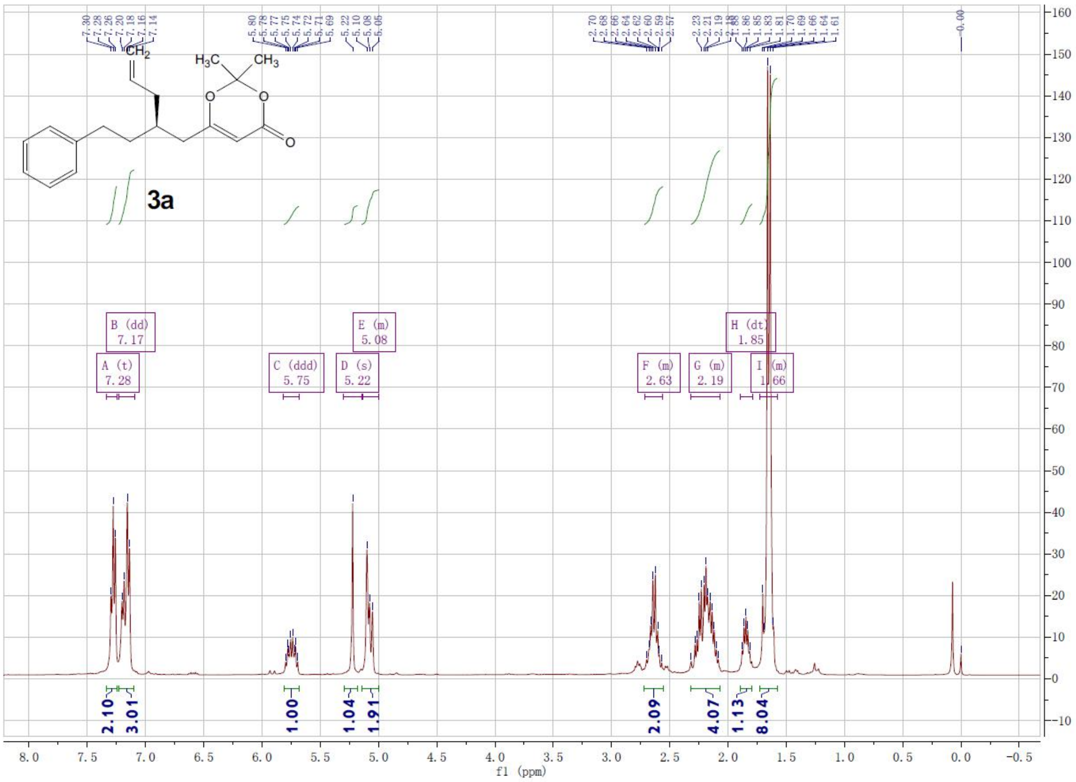

Supplementary Figure 117. ^{1}H NMR spectrum for compound **3a**

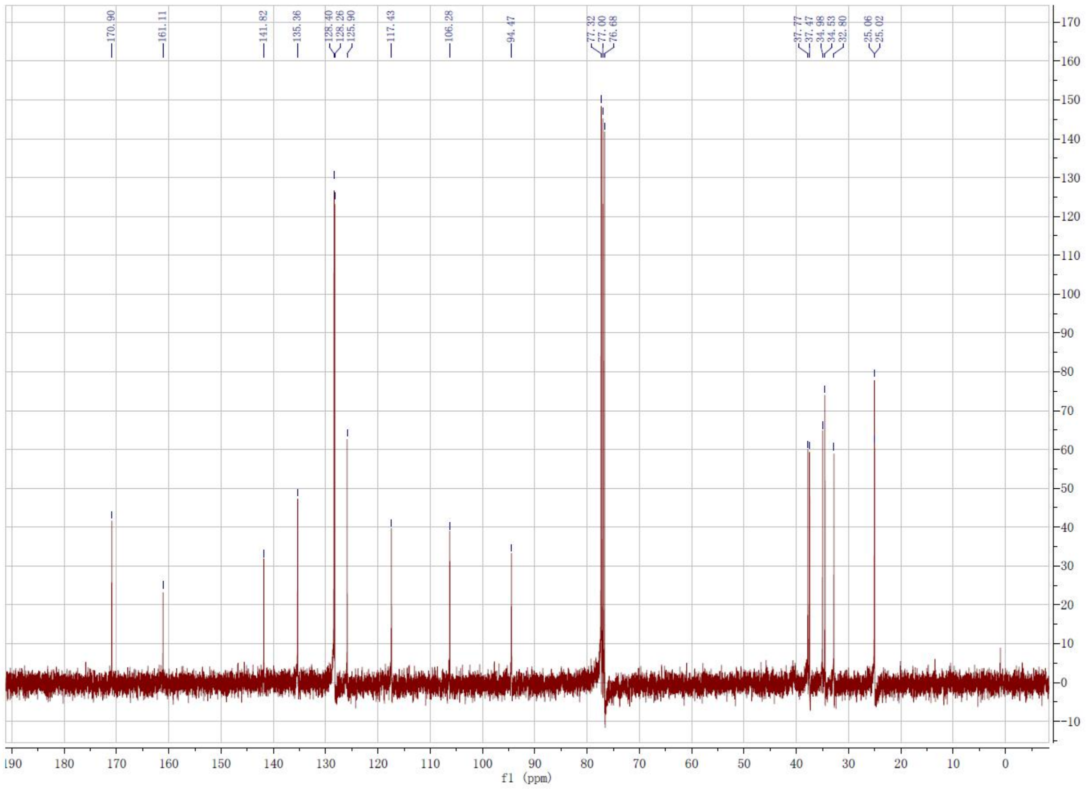

Supplementary Figure 118. ^{13}C NMR spectrum for compound **3a**

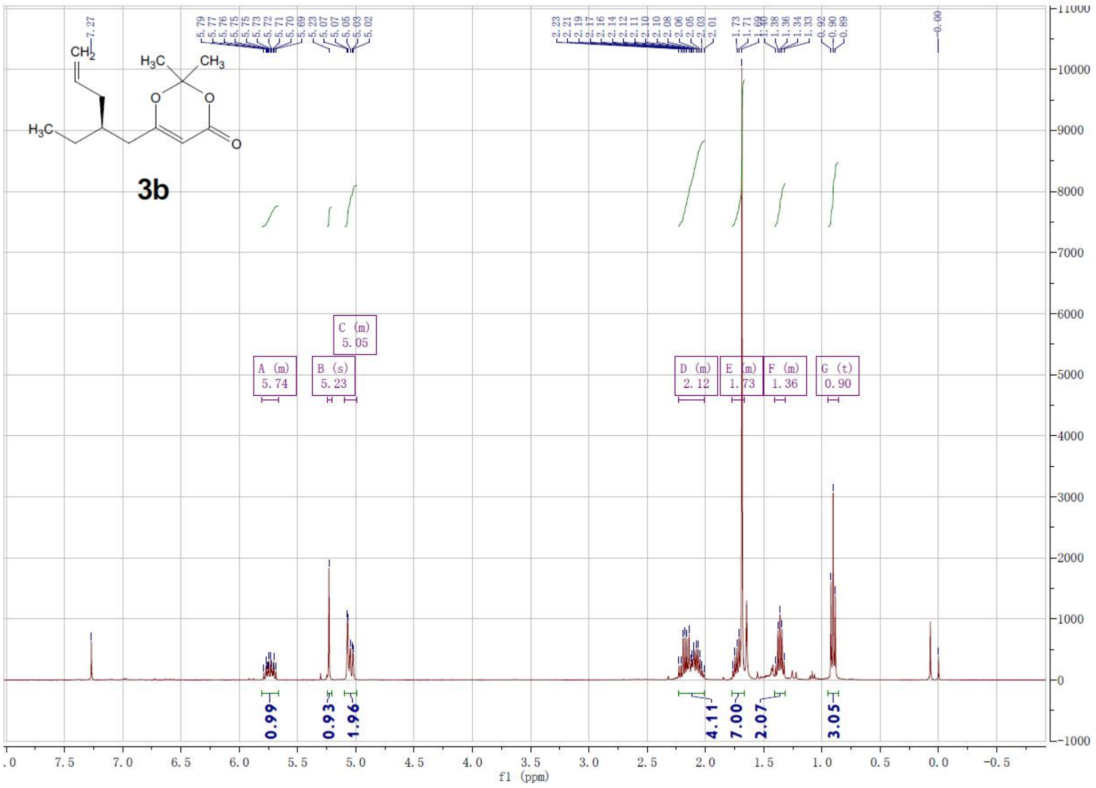

Supplementary Figure 119. ^{1}H NMR spectrum for compound **3b**

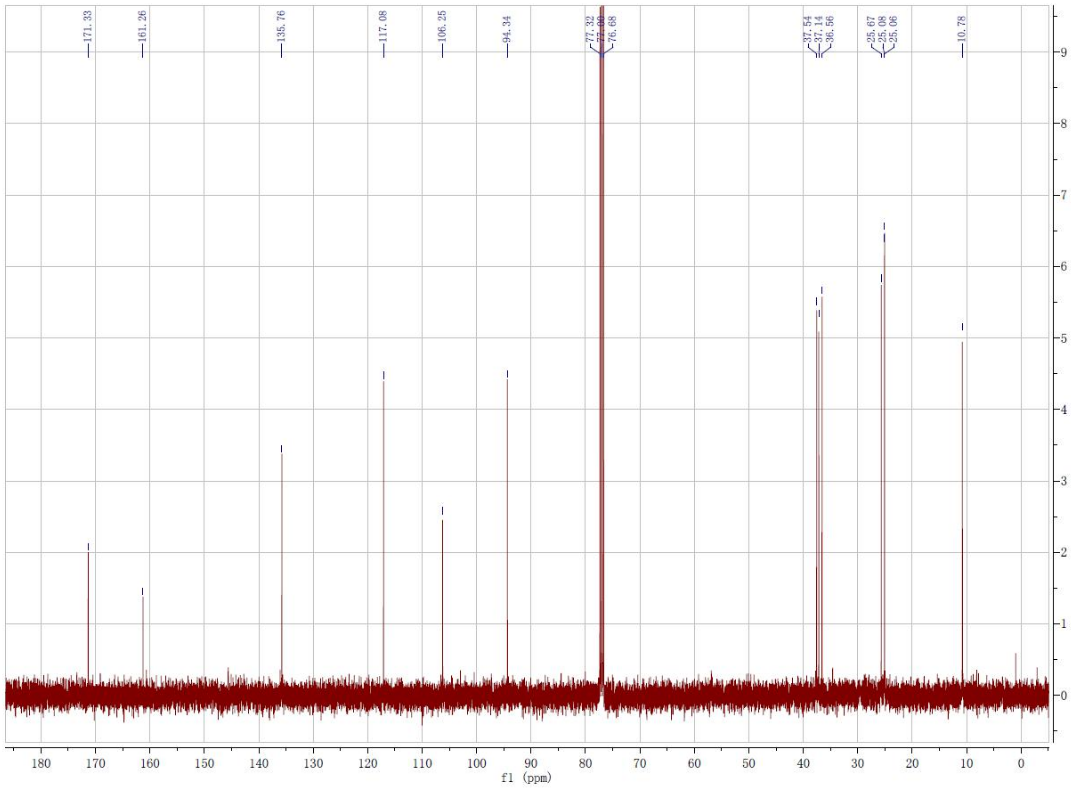

Supplementary Figure 120. ^{13}C NMR spectrum for compound **3b**

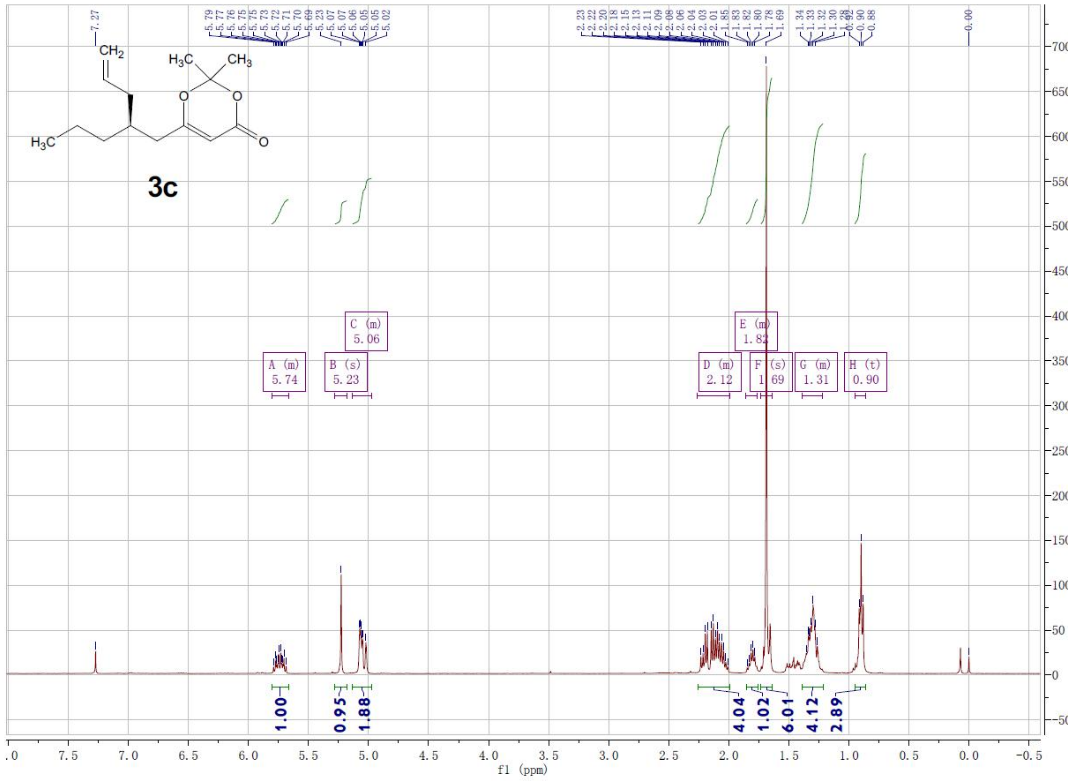

Supplementary Figure 121. ^{1}H NMR spectrum for compound **3c**

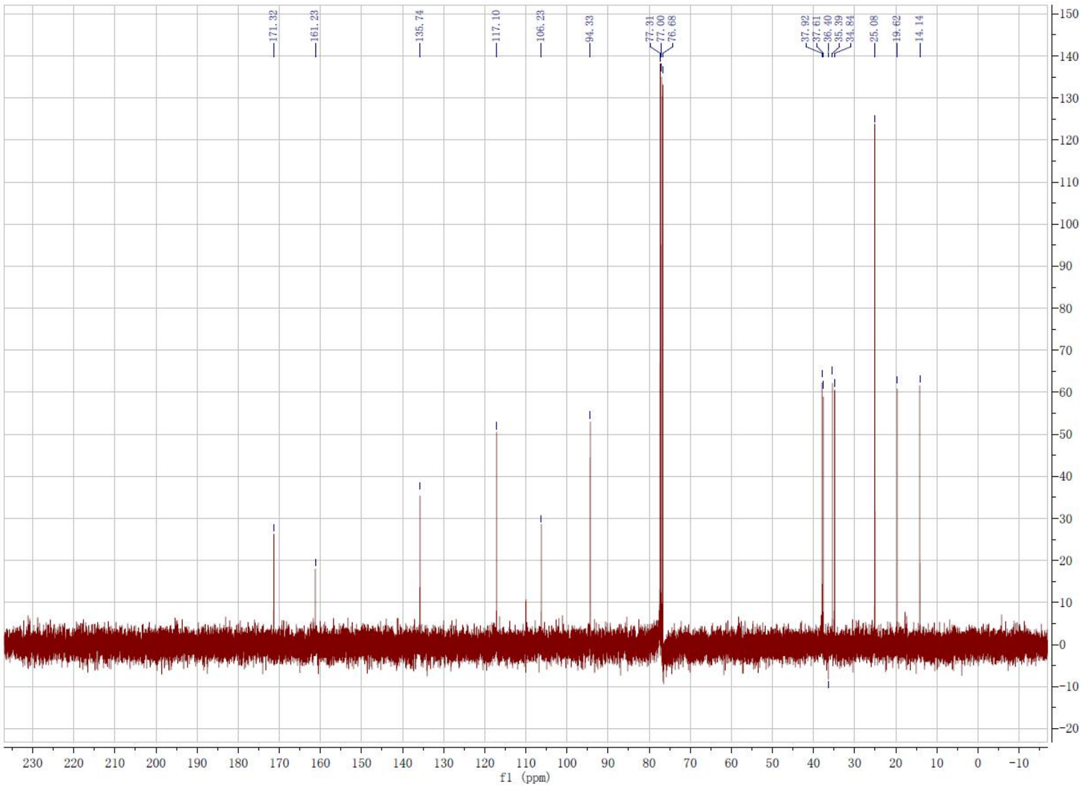

Supplementary Figure 122. ^{13}C NMR spectrum for compound **3c**

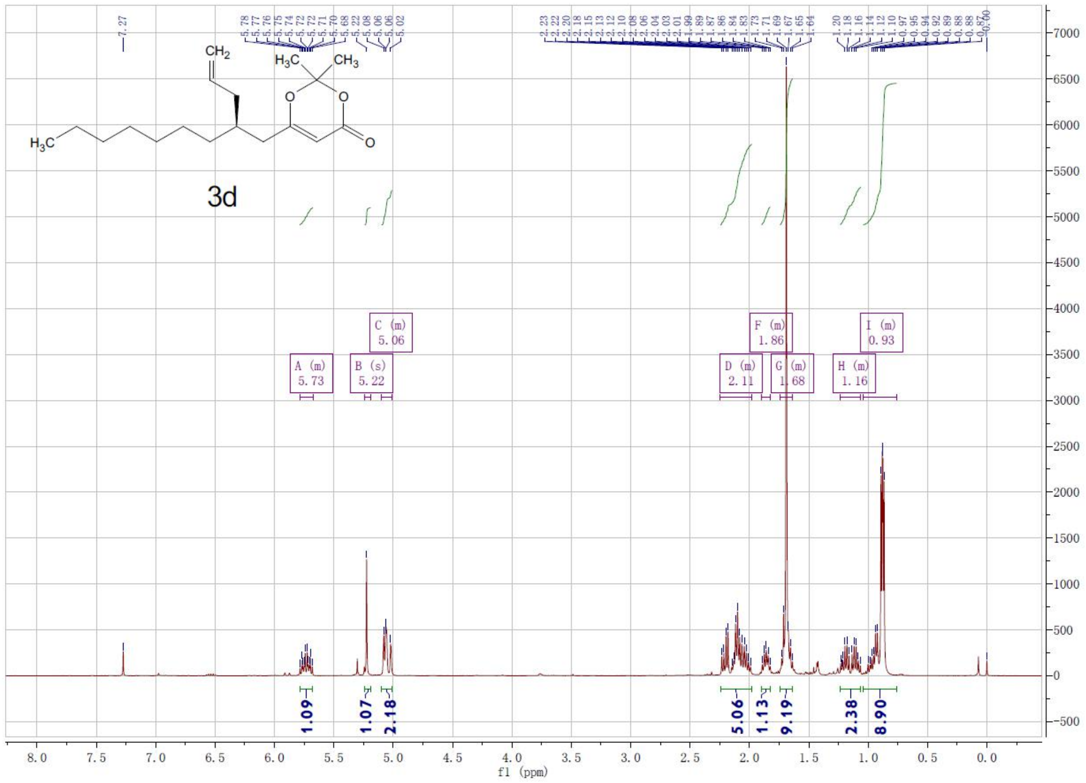

Supplementary Figure 123. ^{1}H NMR spectrum for compound **3d**

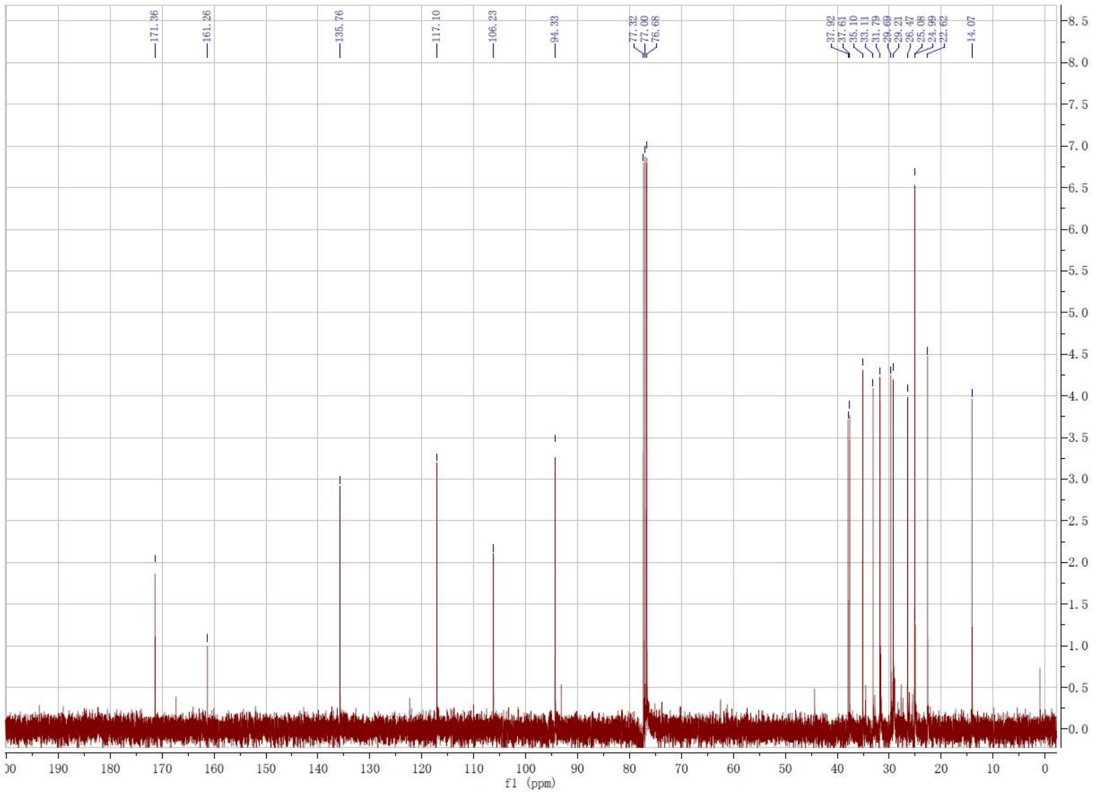

Supplementary Figure 124. ^{13}C NMR spectrum for compound **3d**

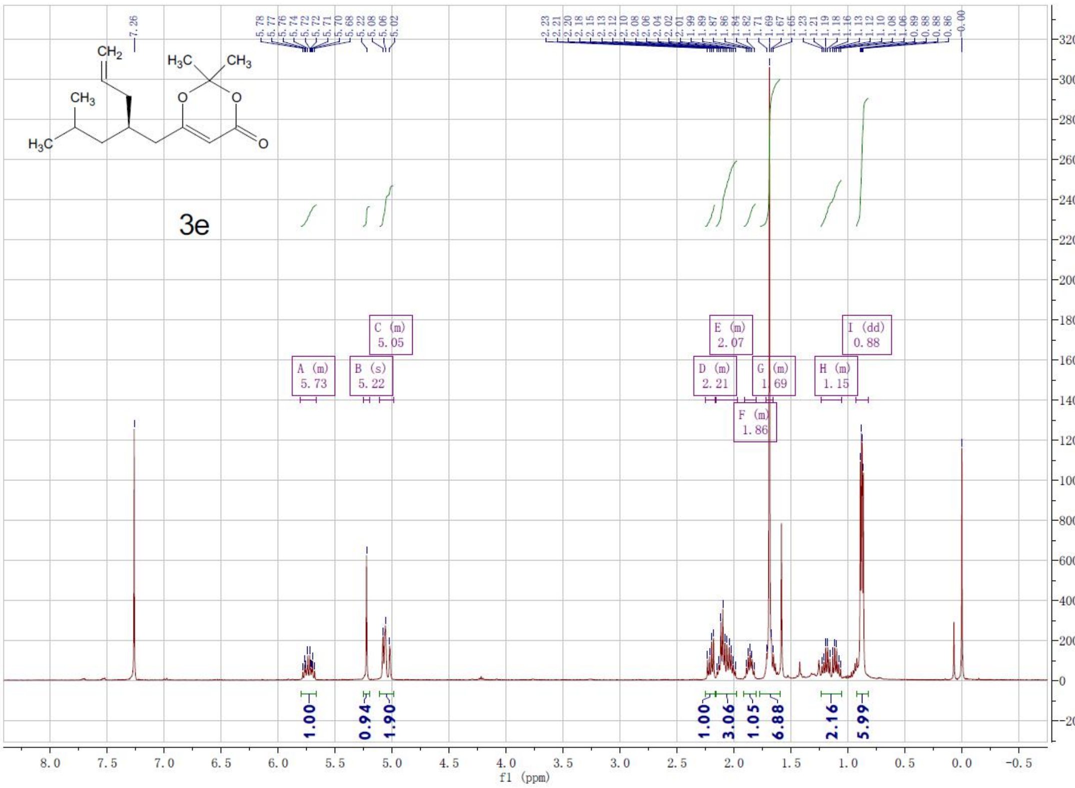

Supplementary Figure 125. ^{1}H NMR spectrum for compound **3e**

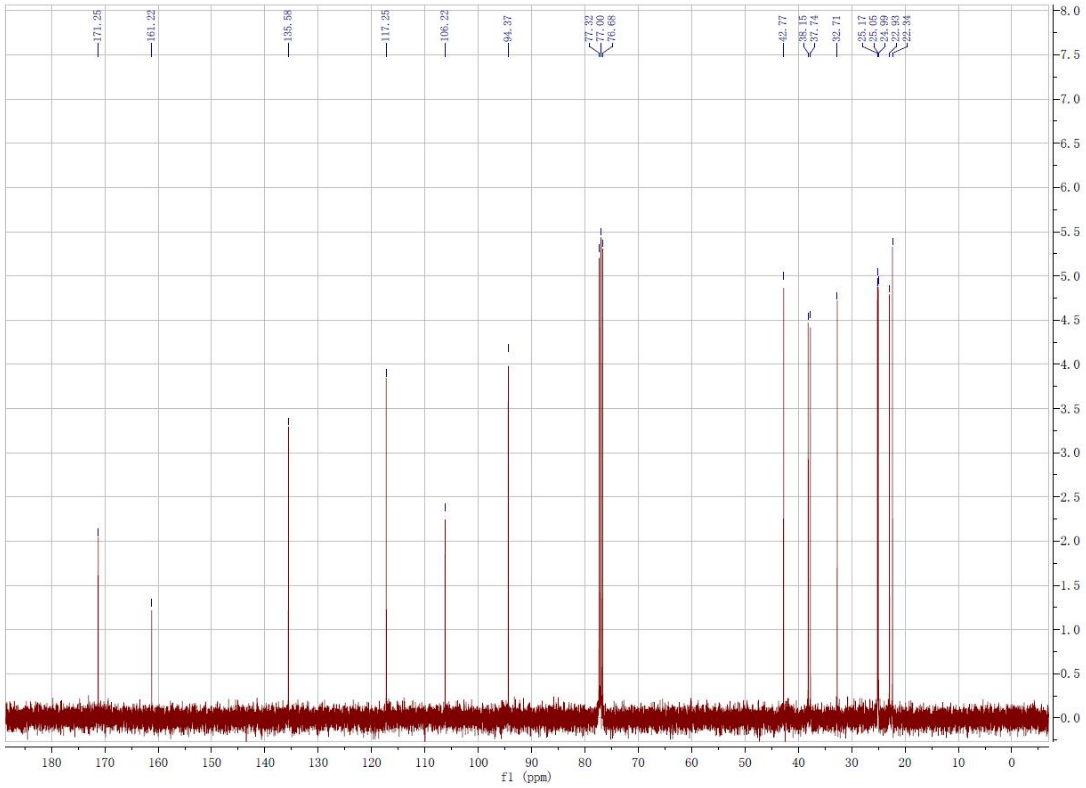

Supplementary Figure 126. ^{13}C NMR spectrum for compound **3e**

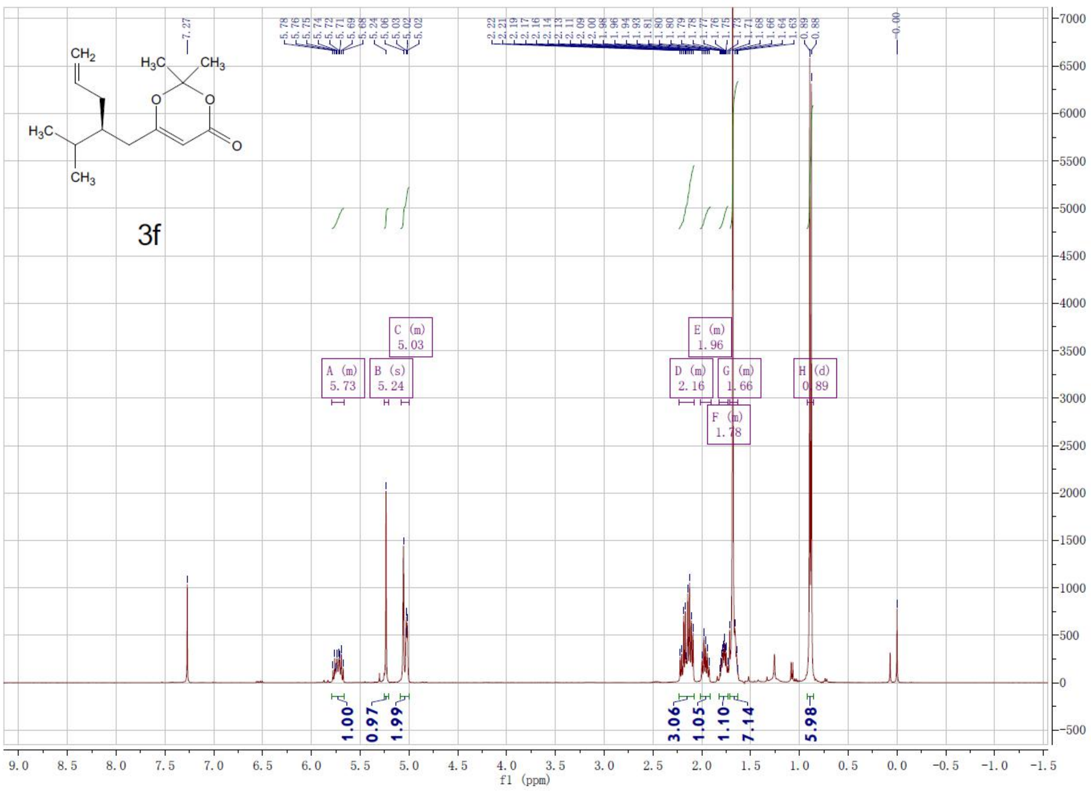

Supplementary Figure 127. ^{1}H NMR spectrum for compound **3f**

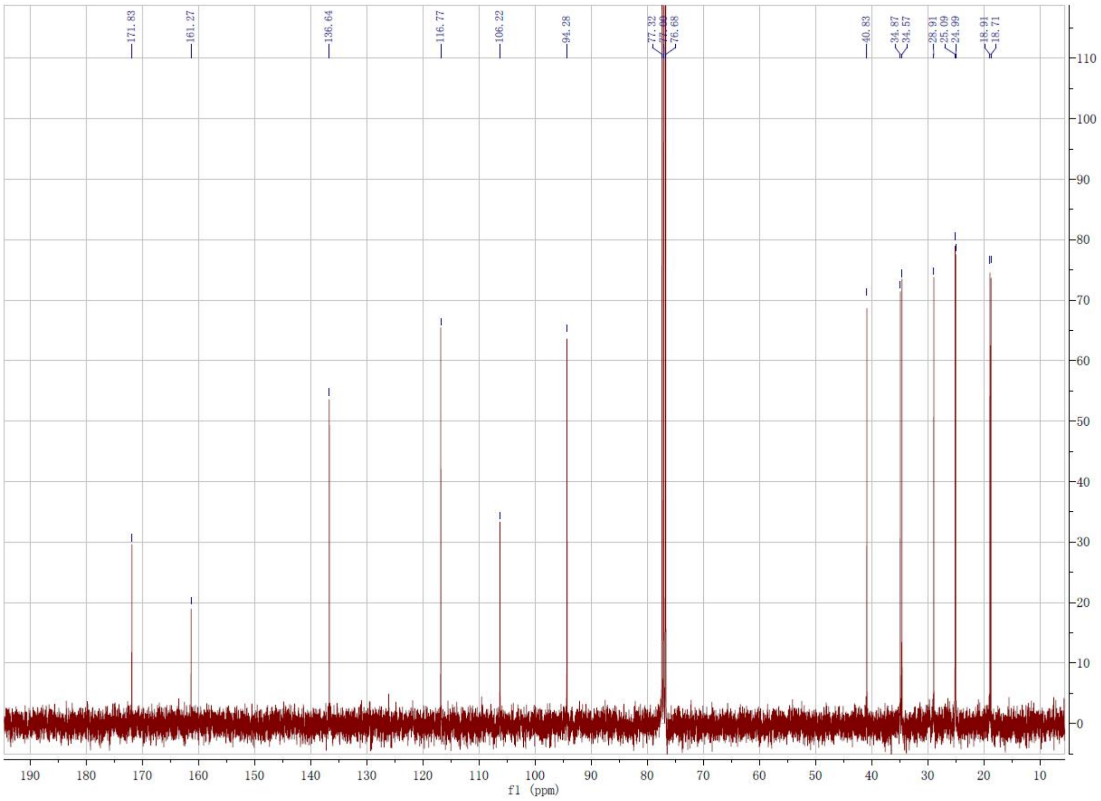

Supplementary Figure 128. ^{13}C NMR spectrum for compound **3f**

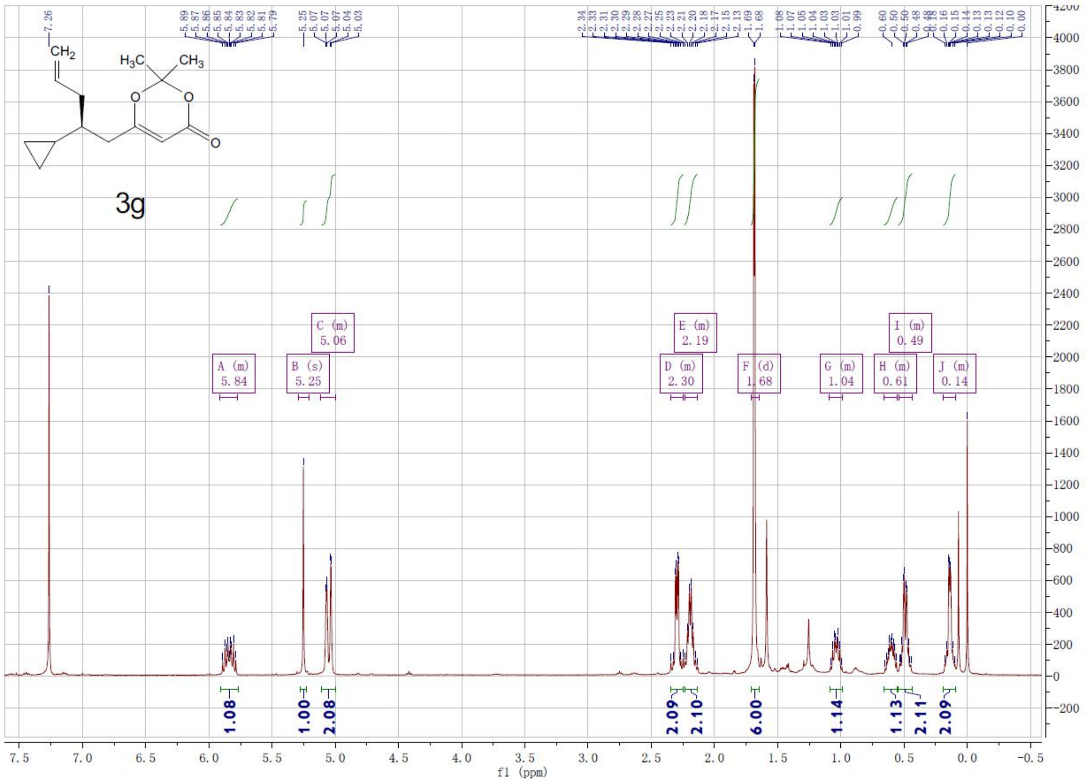

Supplementary Figure 129. ^{1}H NMR spectrum for compound **3g**

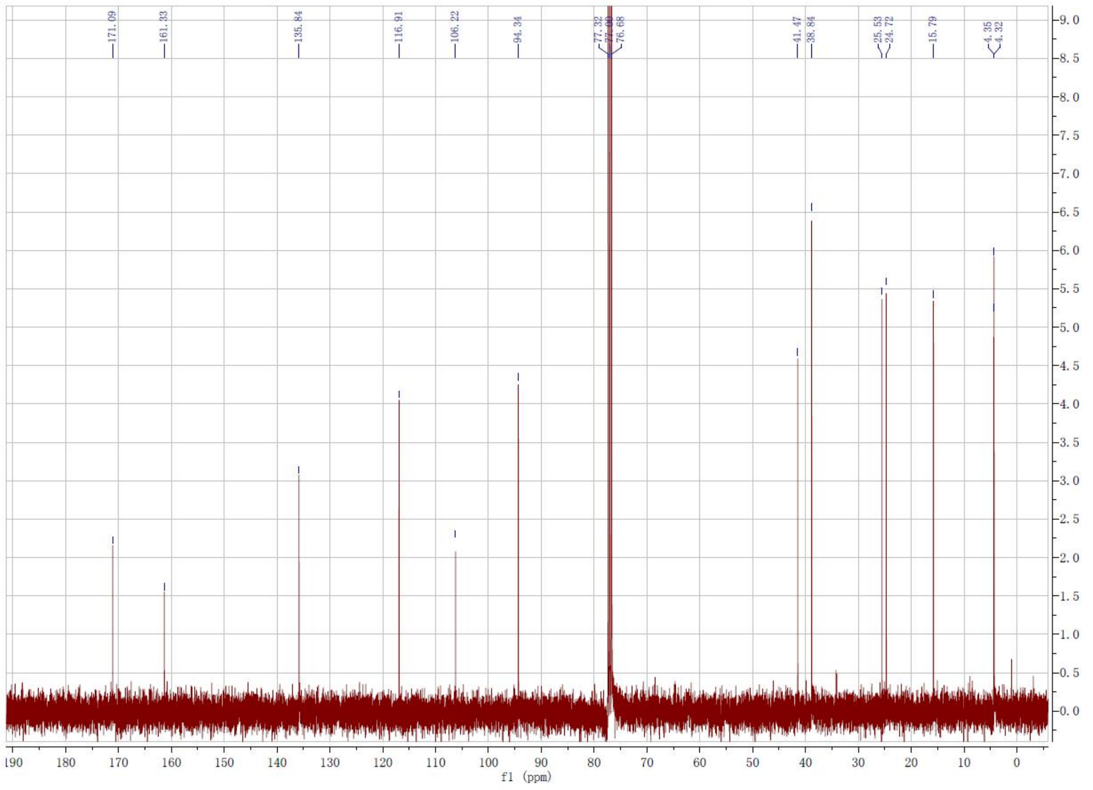

Supplementary Figure 130. ^{13}C NMR spectrum for compound **3g**

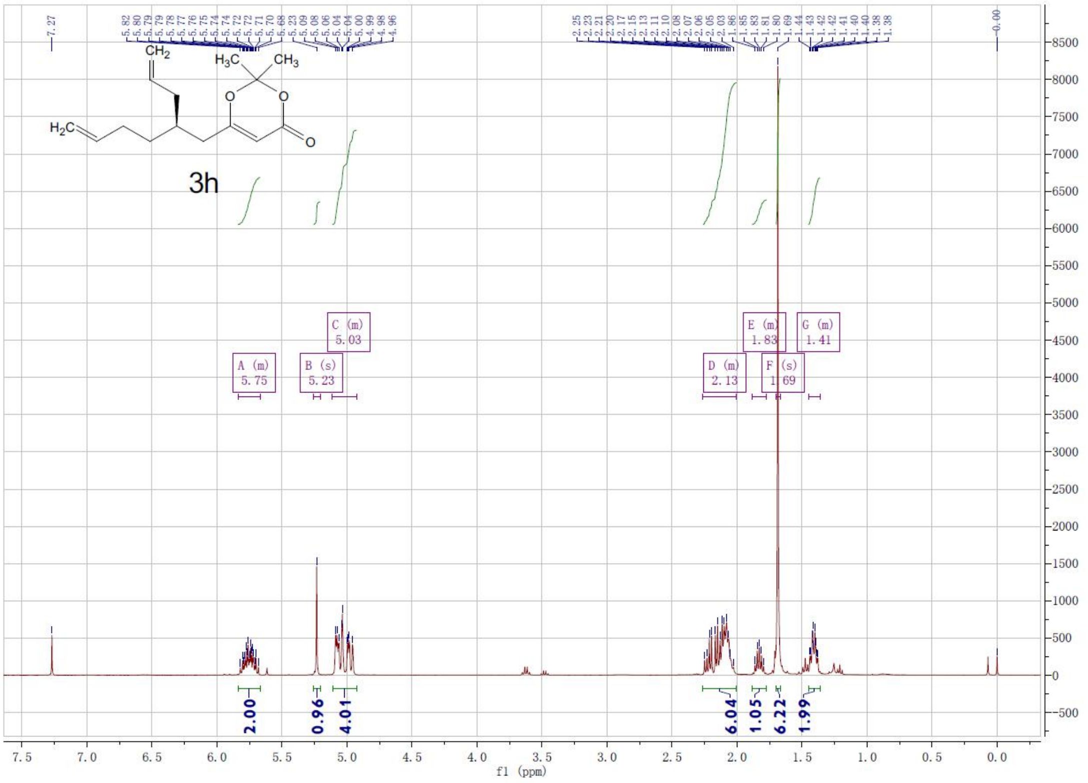

Supplementary Figure 131. ^{1}H NMR spectrum for compound **3h**

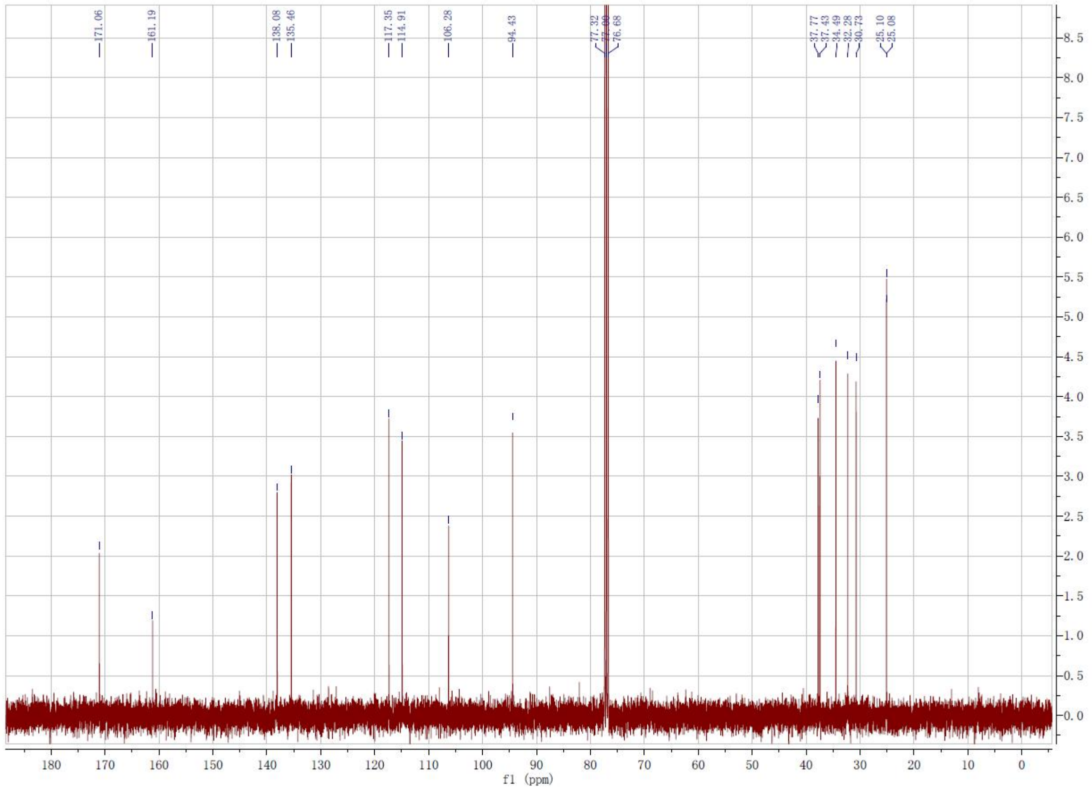

Supplementary Figure 132. ^{13}C NMR spectrum for compound **3h**

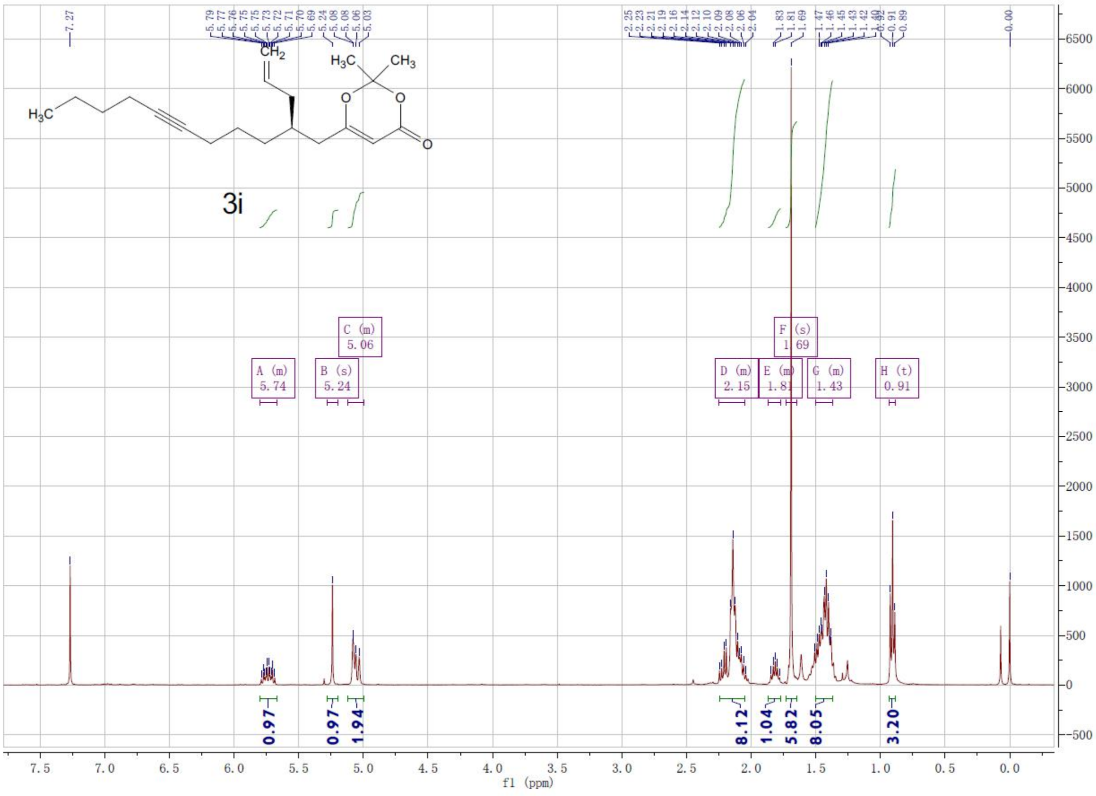

Supplementary Figure 133. ^{1}H NMR spectrum for compound **3i**

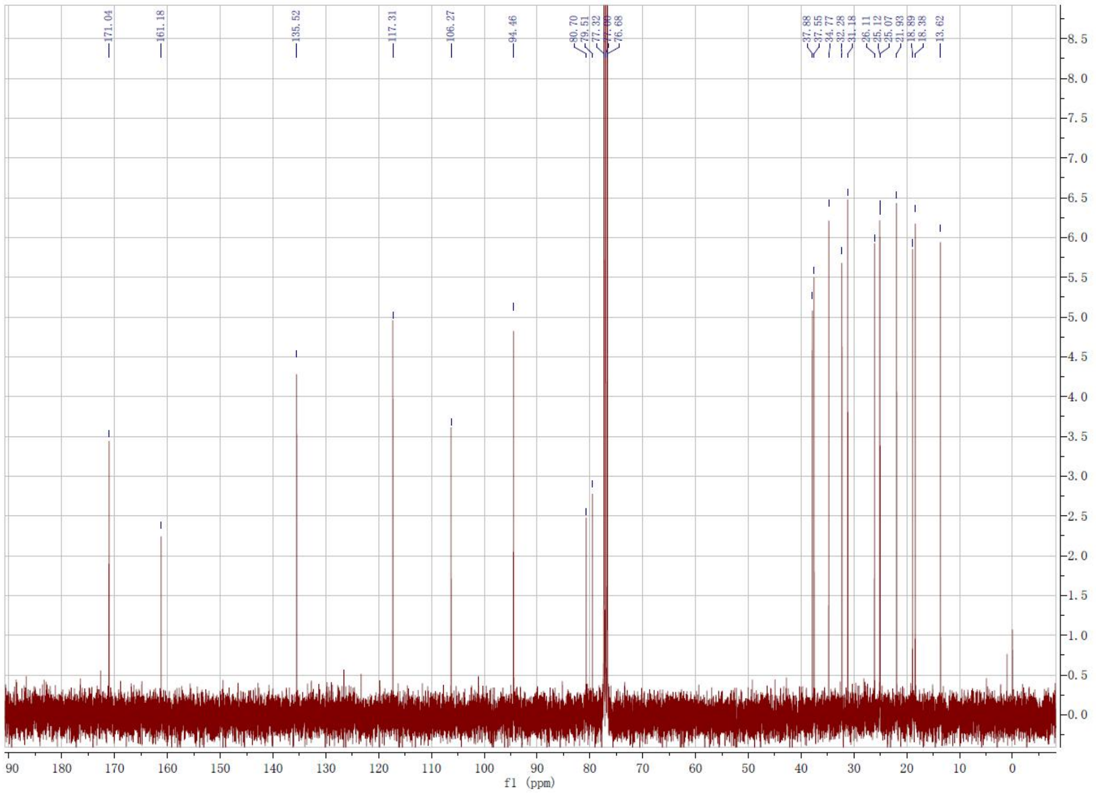

Supplementary Figure 134. ^{13}C NMR spectrum for compound **3i**

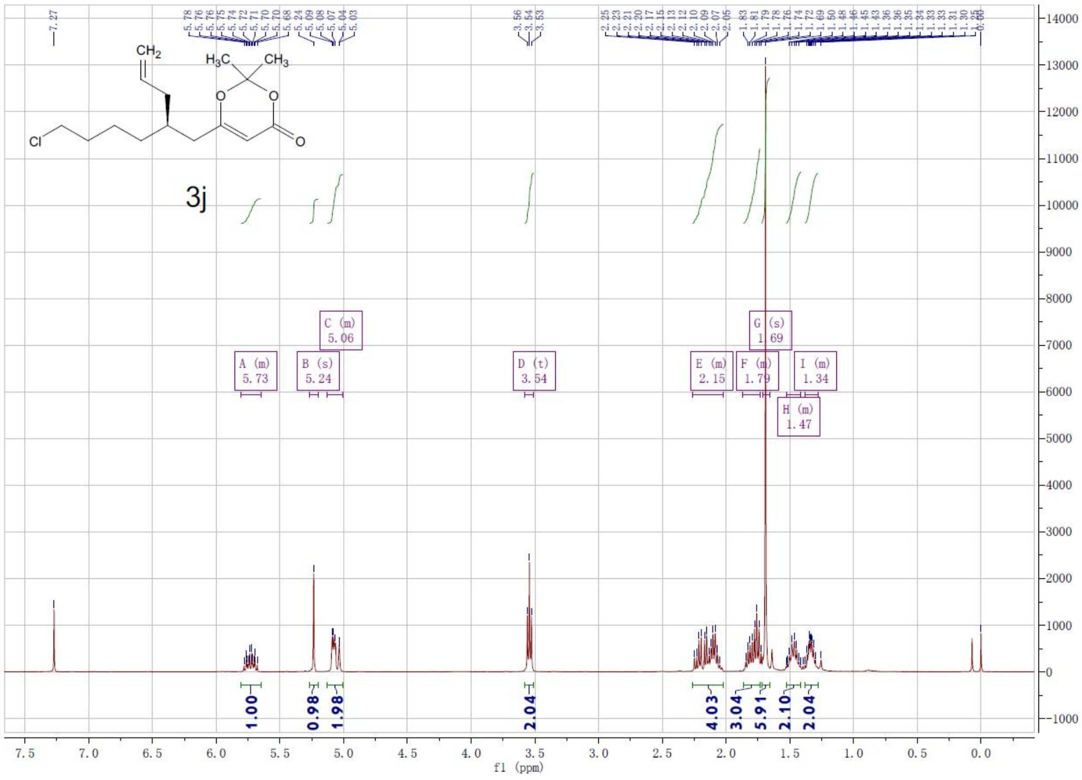

Supplementary Figure 135. ^{1}H NMR spectrum for compound **3j**

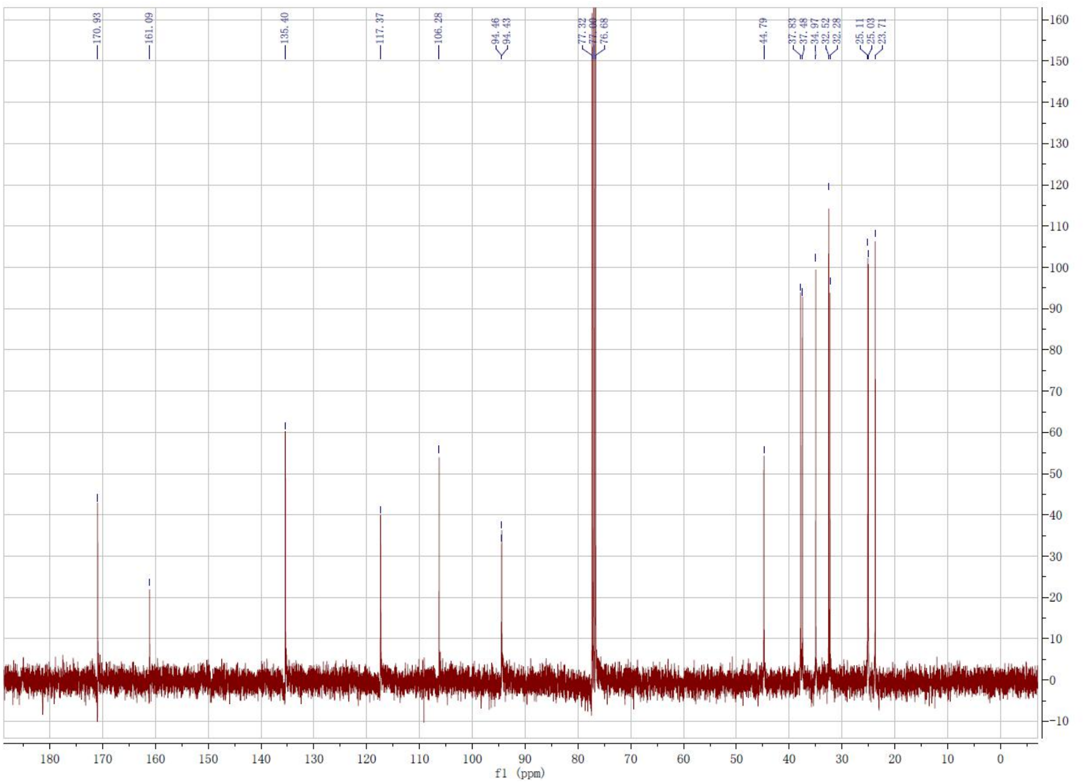

Supplementary Figure 136. ^{13}C NMR spectrum for compound **3j**

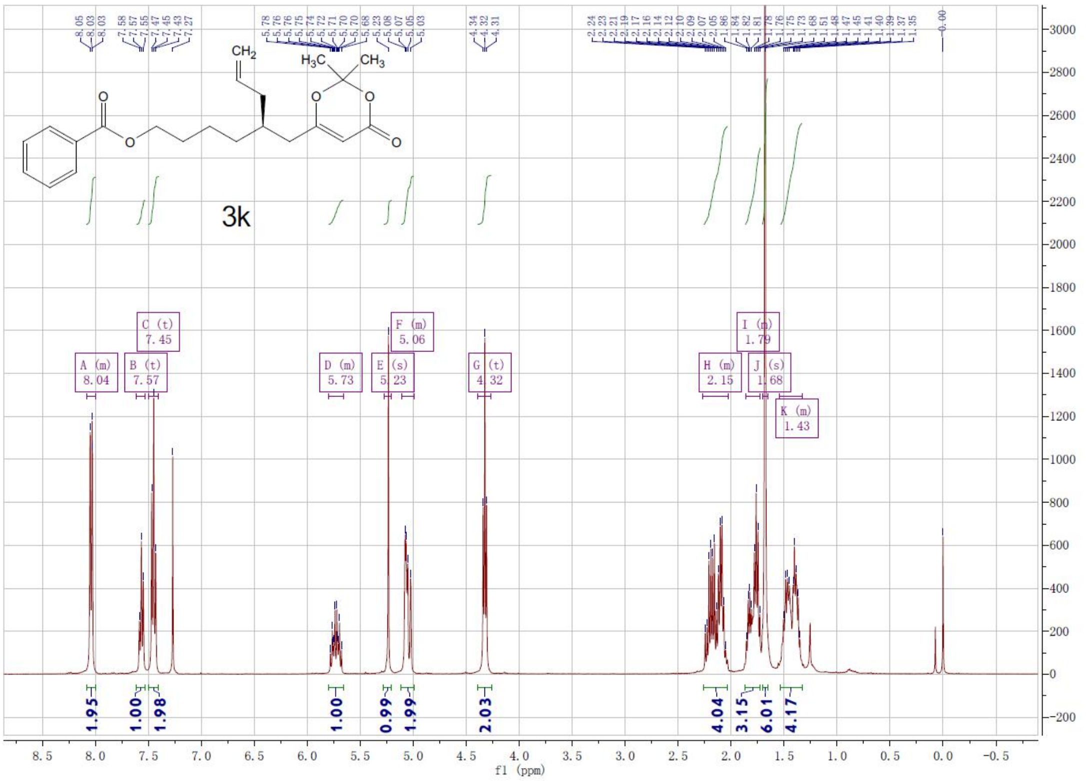

Supplementary Figure 137. ^{1}H NMR spectrum for compound **3k**

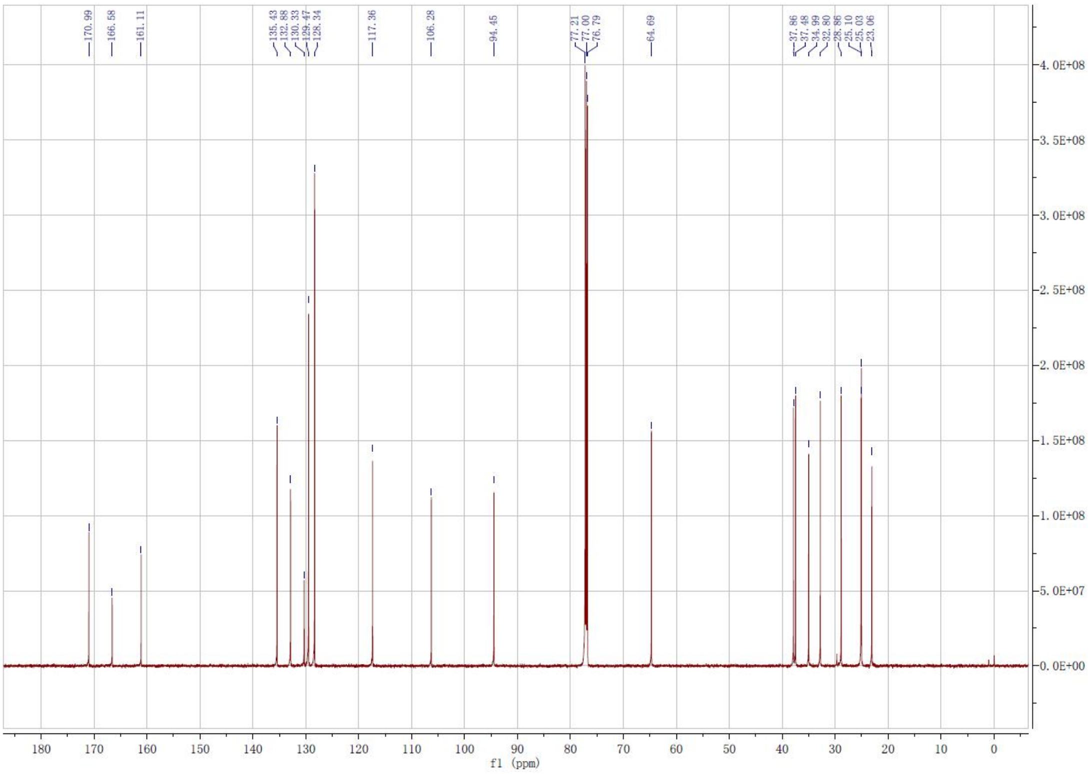

Supplementary Figure 138. ^{13}C NMR spectrum for compound **3k**

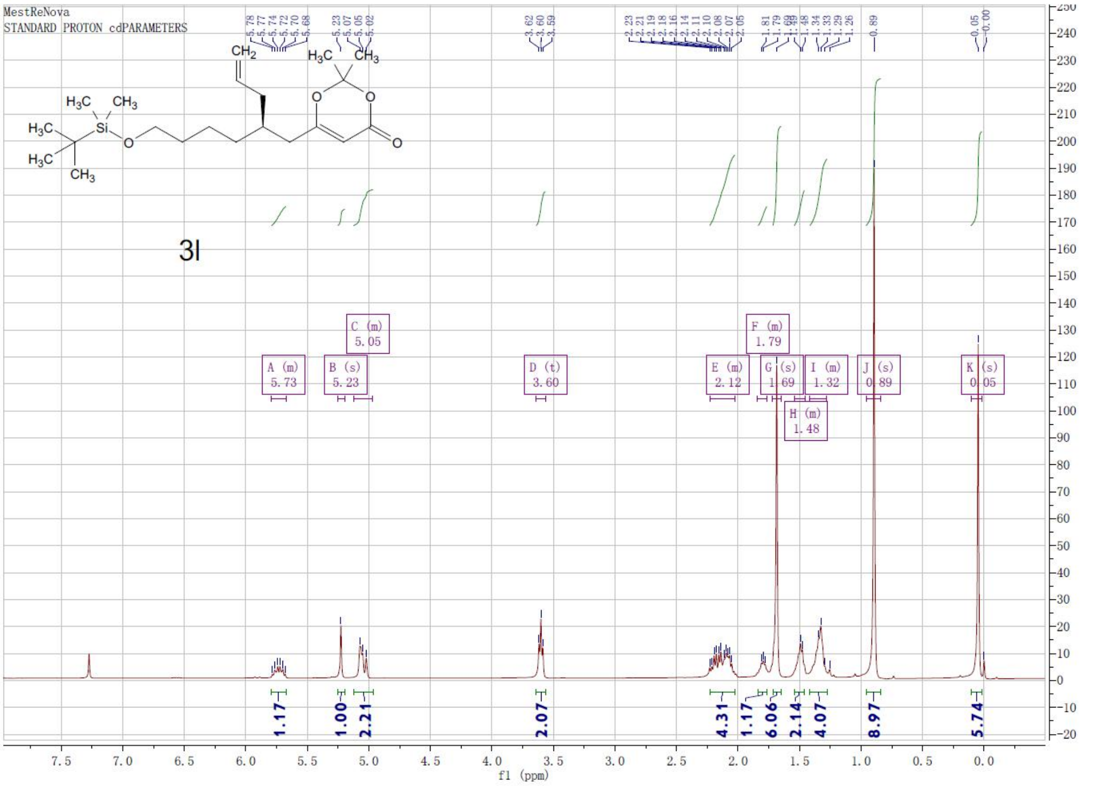

Supplementary Figure 139. ^{1}H NMR spectrum for compound **3l**

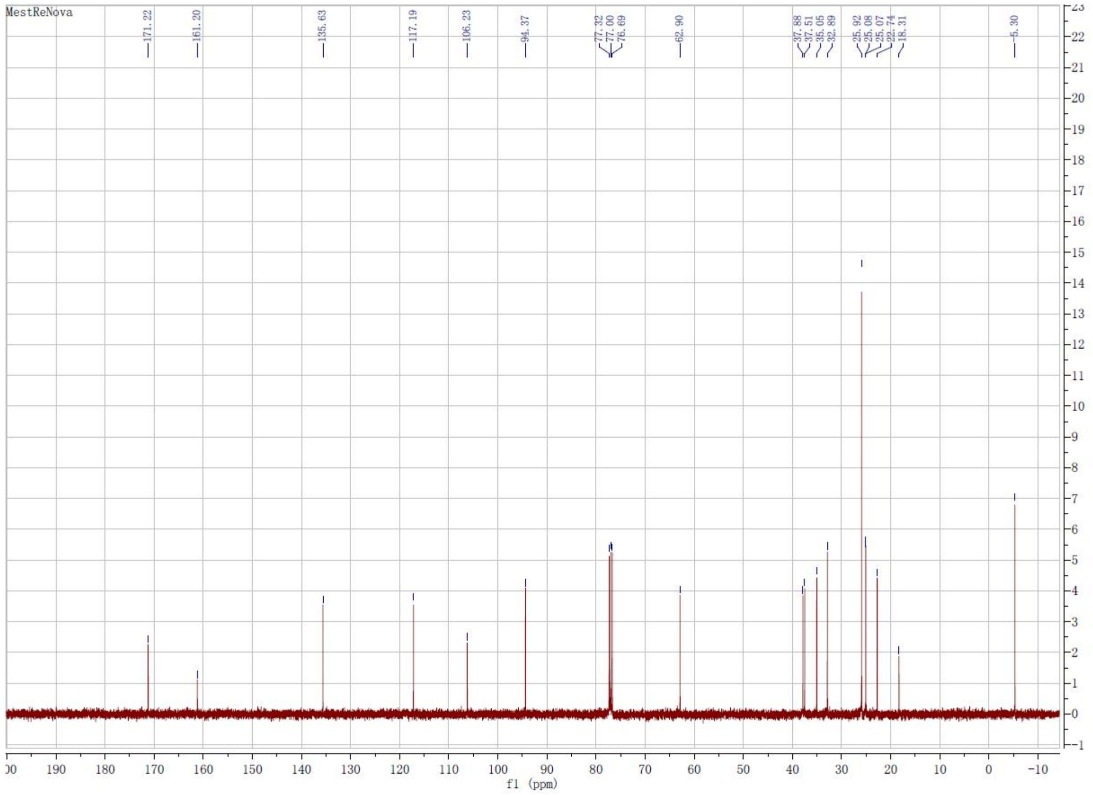

Supplementary Figure 140. ^{13}C NMR spectrum for compound **3l**

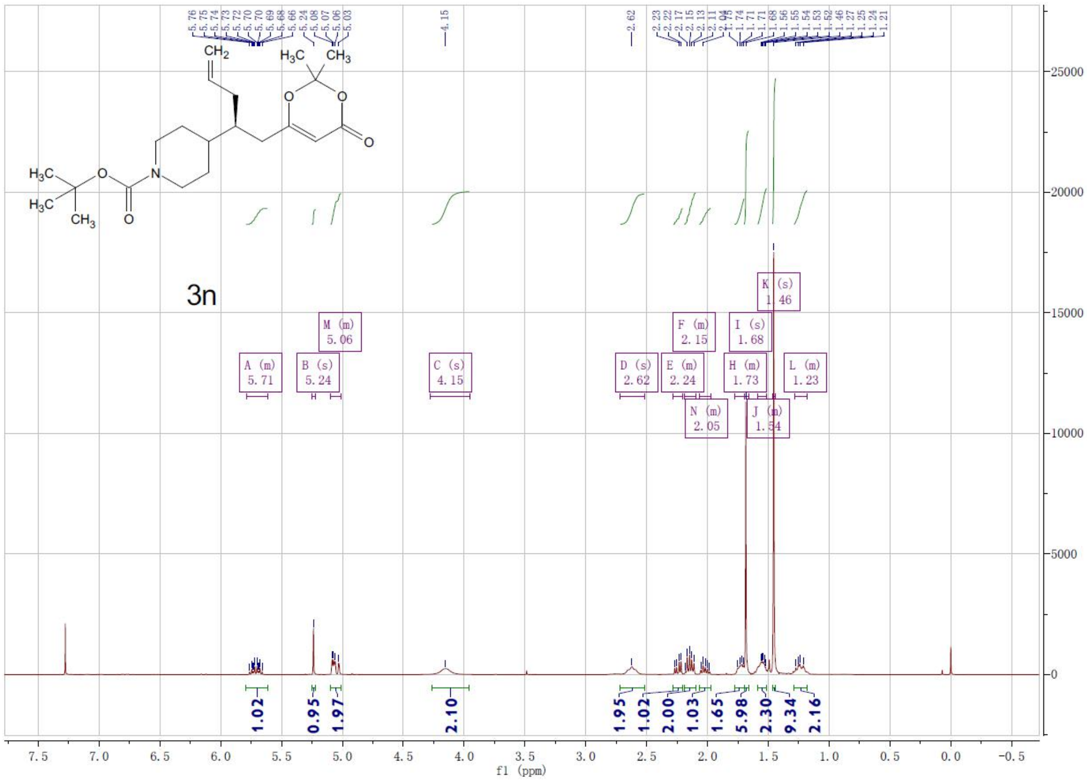

Supplementary Figure 141. ^{1}H NMR spectrum for compound **3n**

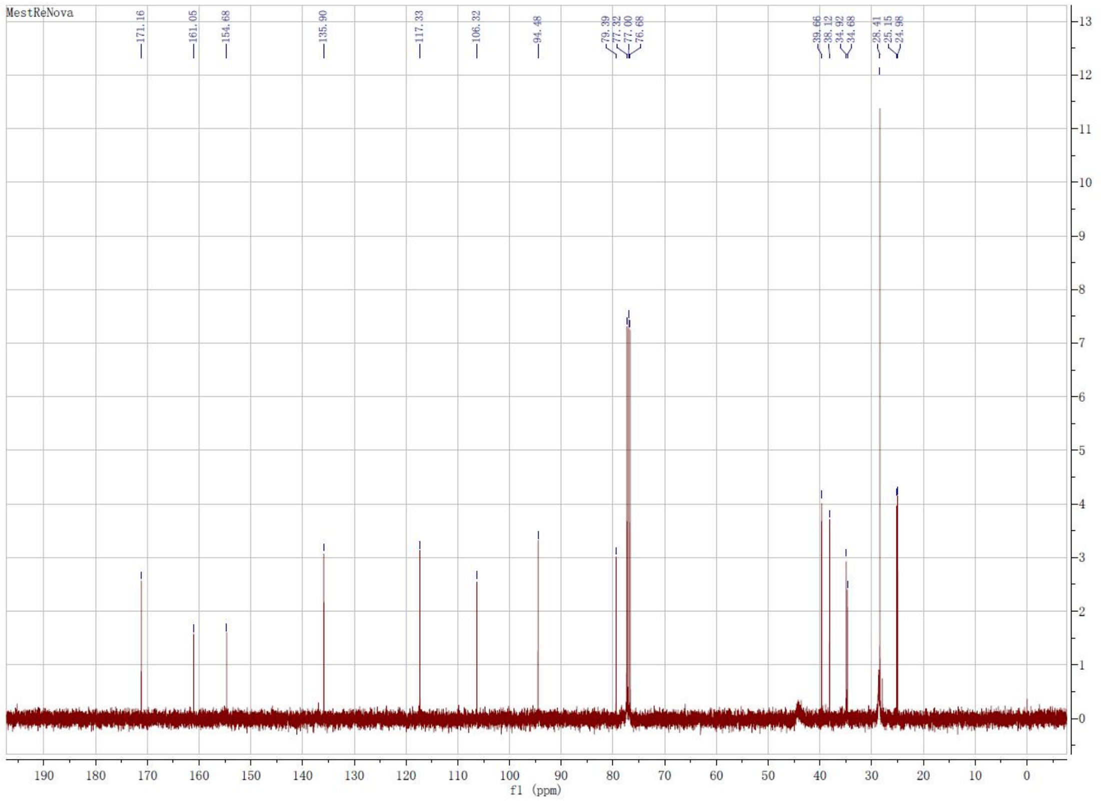

Supplementary Figure 142. ^{13}C NMR spectrum for compound **3n**

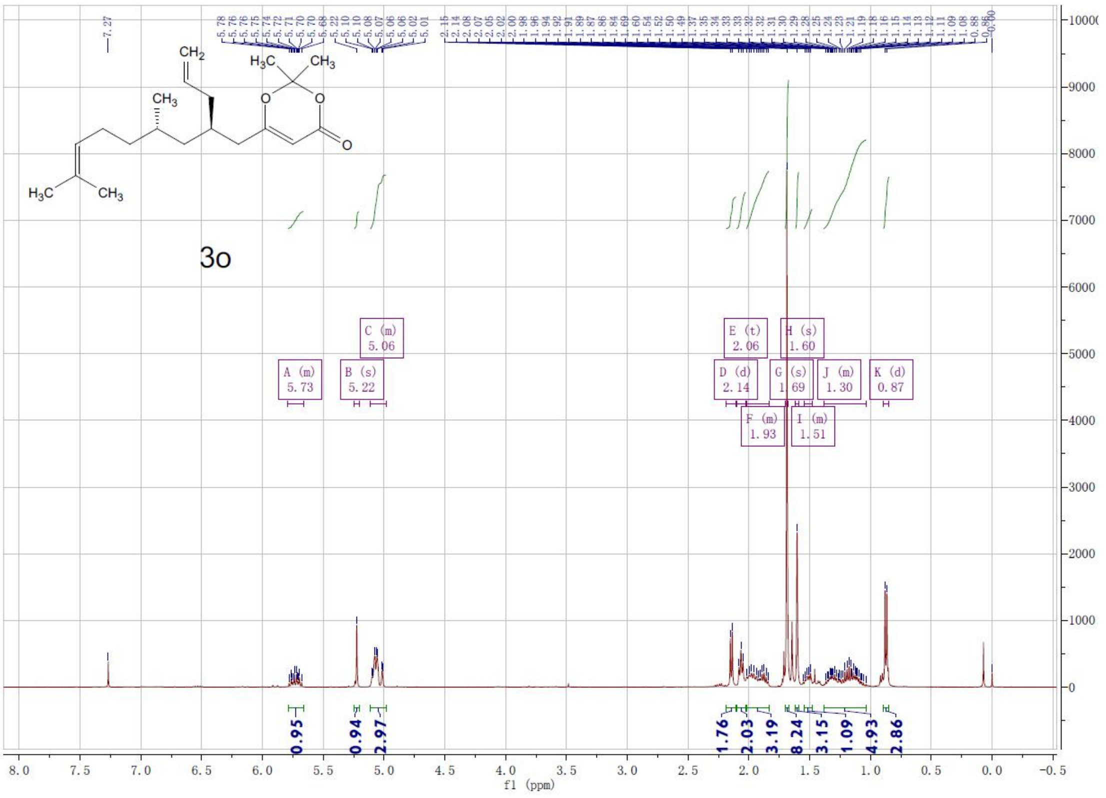

Supplementary Figure 143. ^{1}H NMR spectrum for compound **3o**

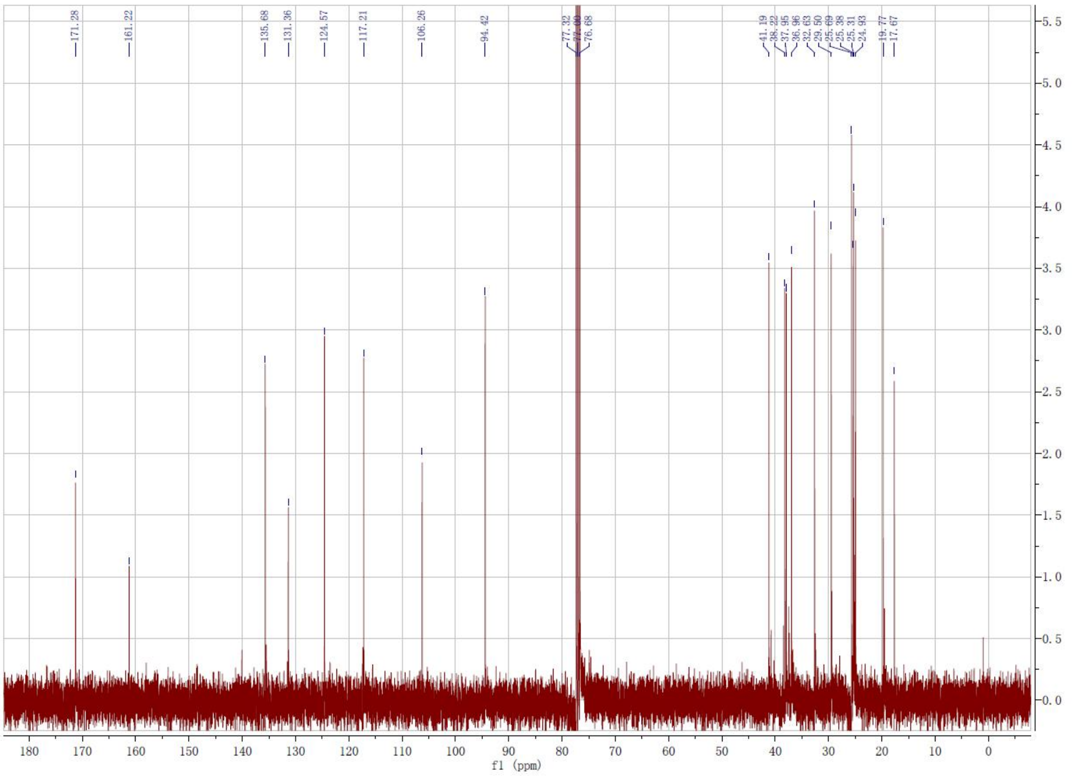

Supplementary Figure 144. ^{13}C NMR spectrum for compound **3o**

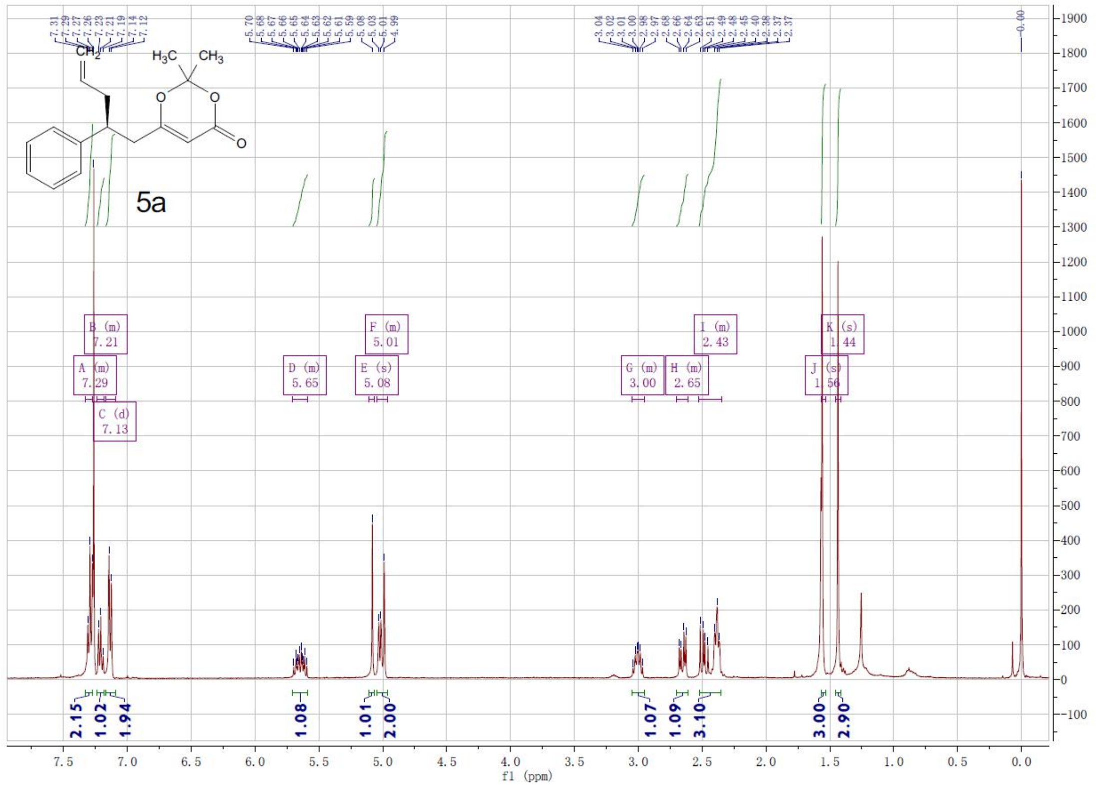

Supplementary Figure 145. ^{1}H NMR spectrum for compound **5a**

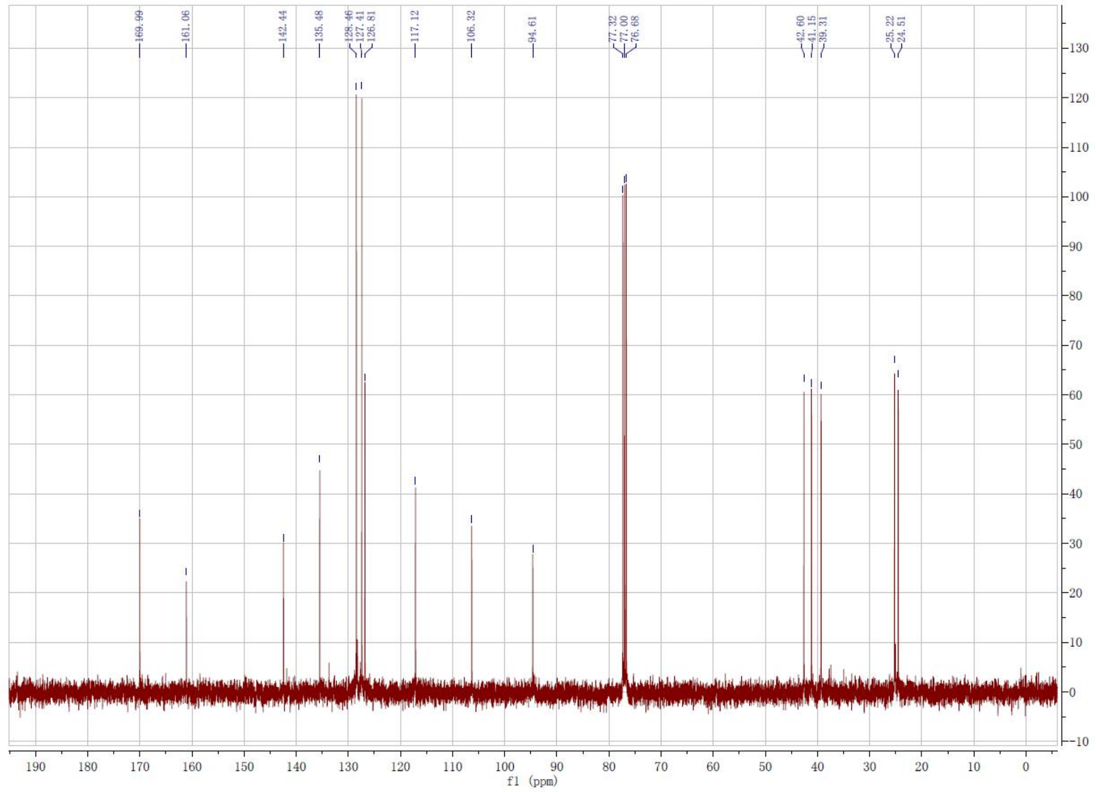

Supplementary Figure 146. ^{13}C NMR spectrum for compound **5a**

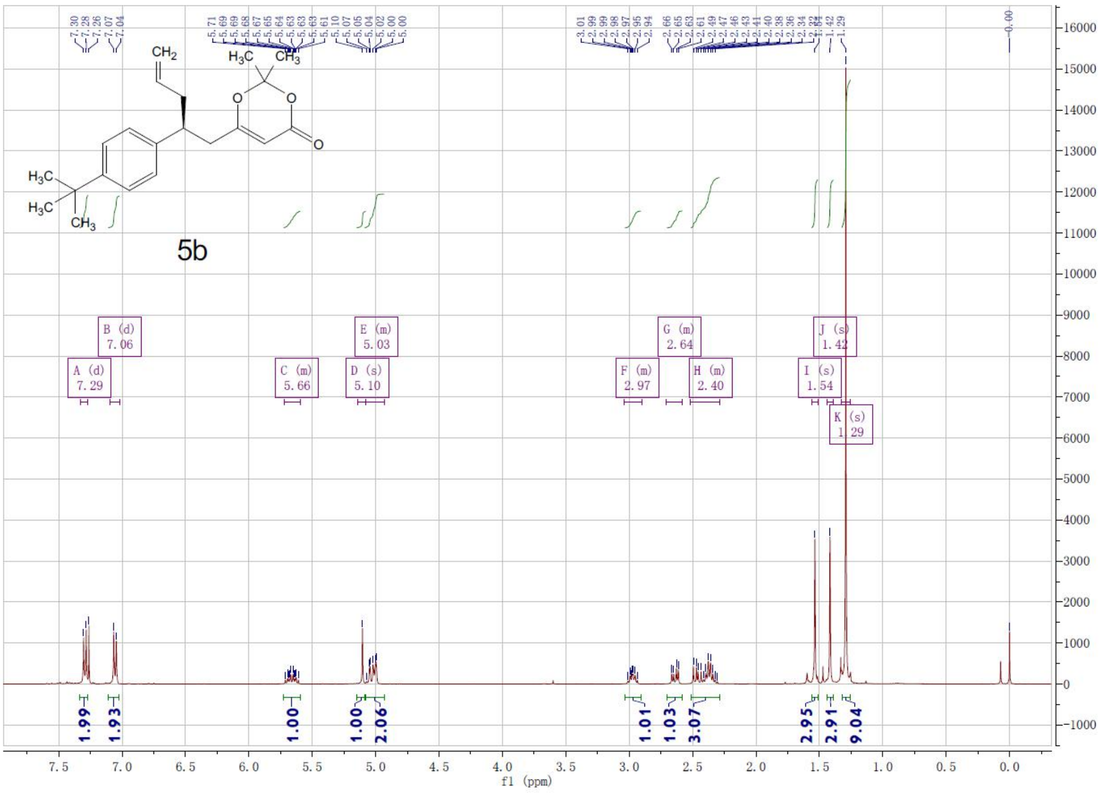

Supplementary Figure 147. ^{1}H NMR spectrum for compound **5b**

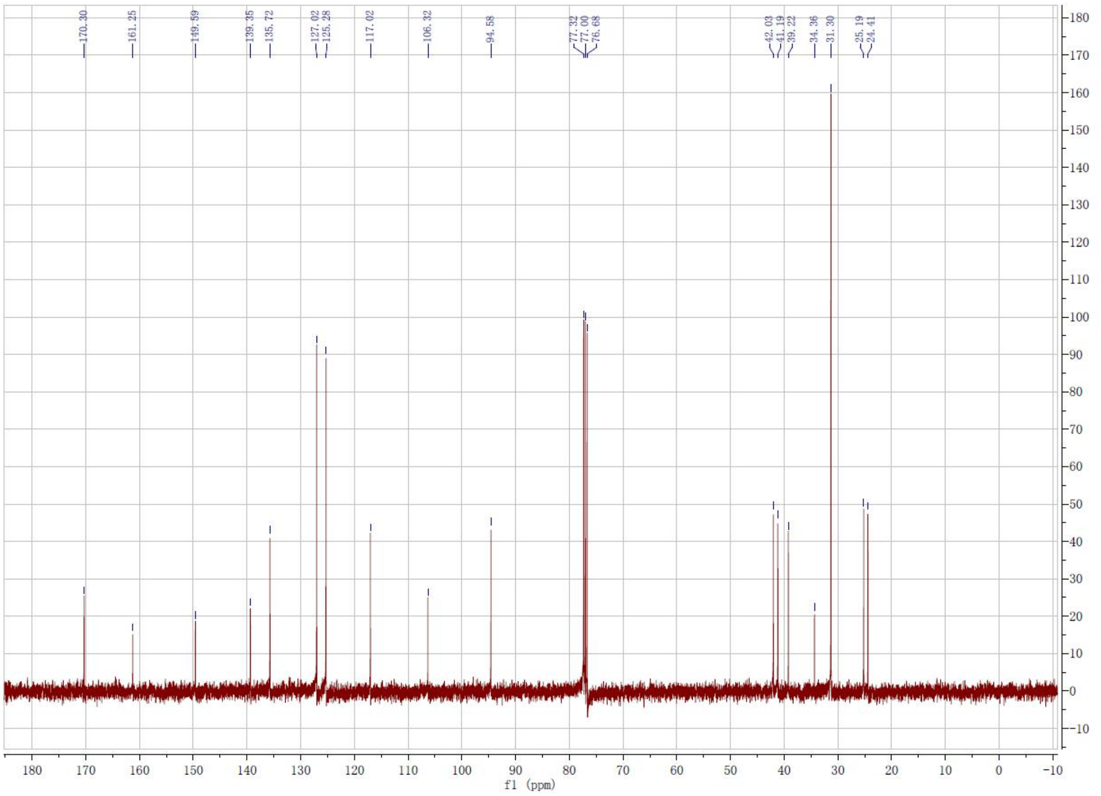

Supplementary Figure 148. ^{13}C NMR spectrum for compound **5b**

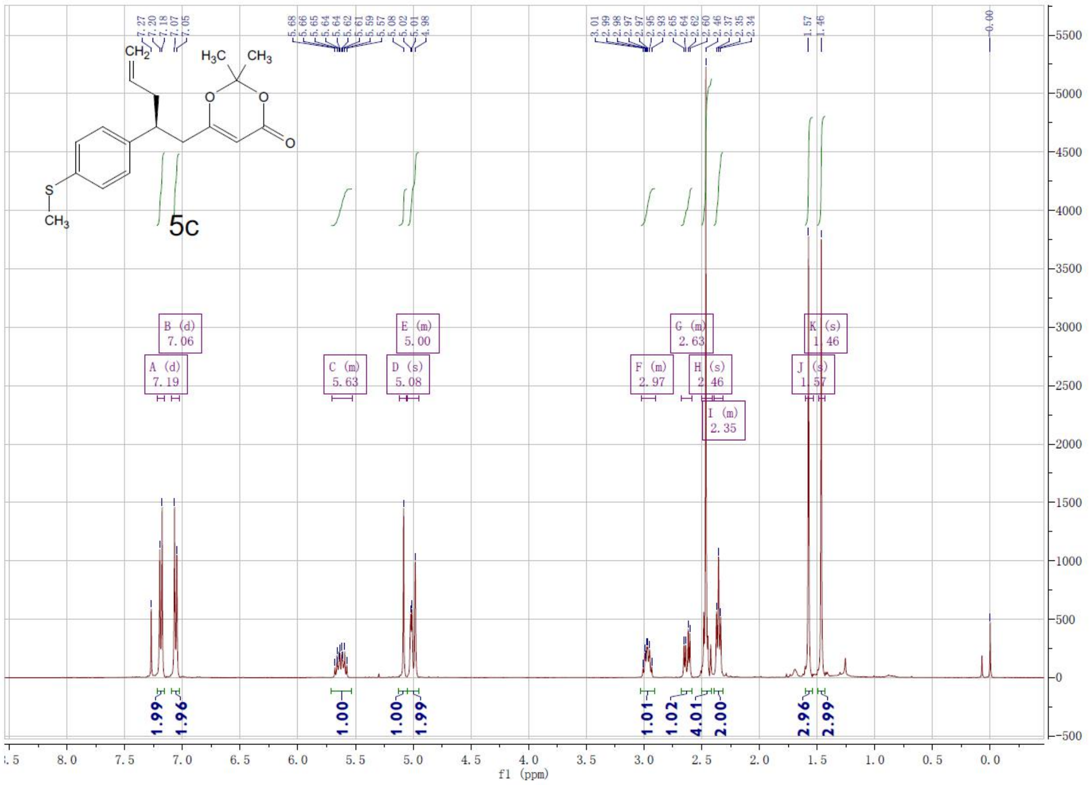

Supplementary Figure 149. ^{1}H NMR spectrum for compound **5c**

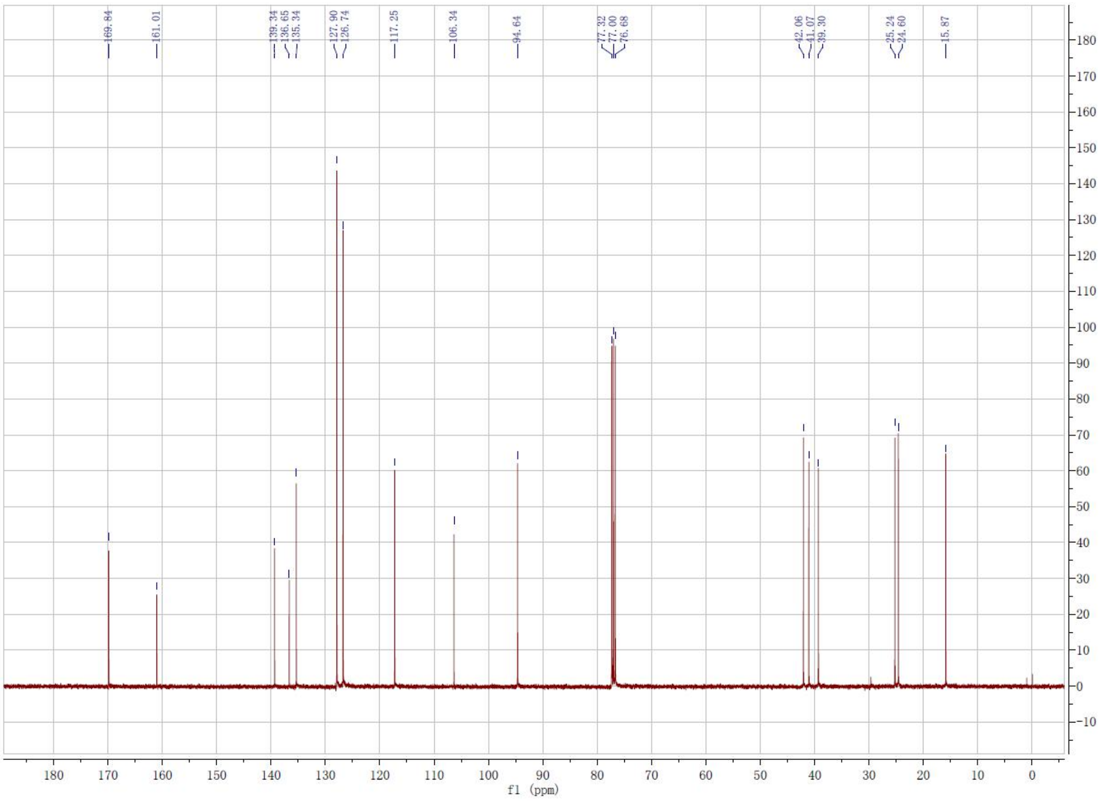

Supplementary Figure 150. ^{13}C NMR spectrum for compound **5c**

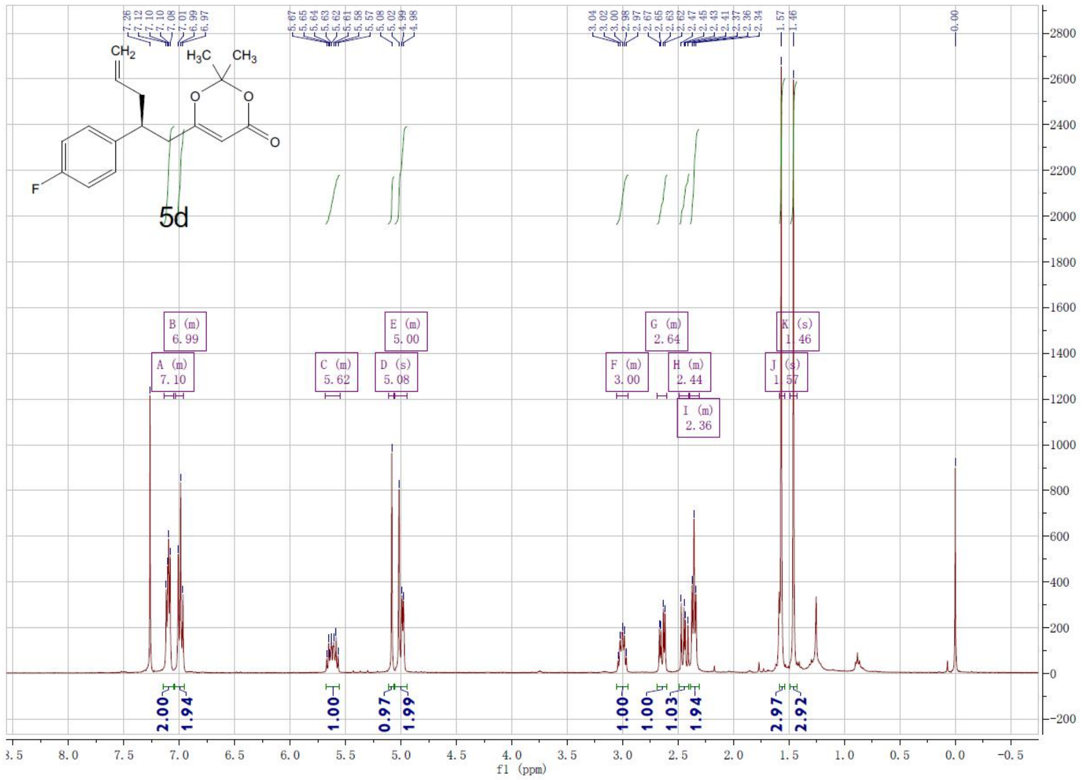

Supplementary Figure 151. ^{1}H NMR spectrum for compound **5d**

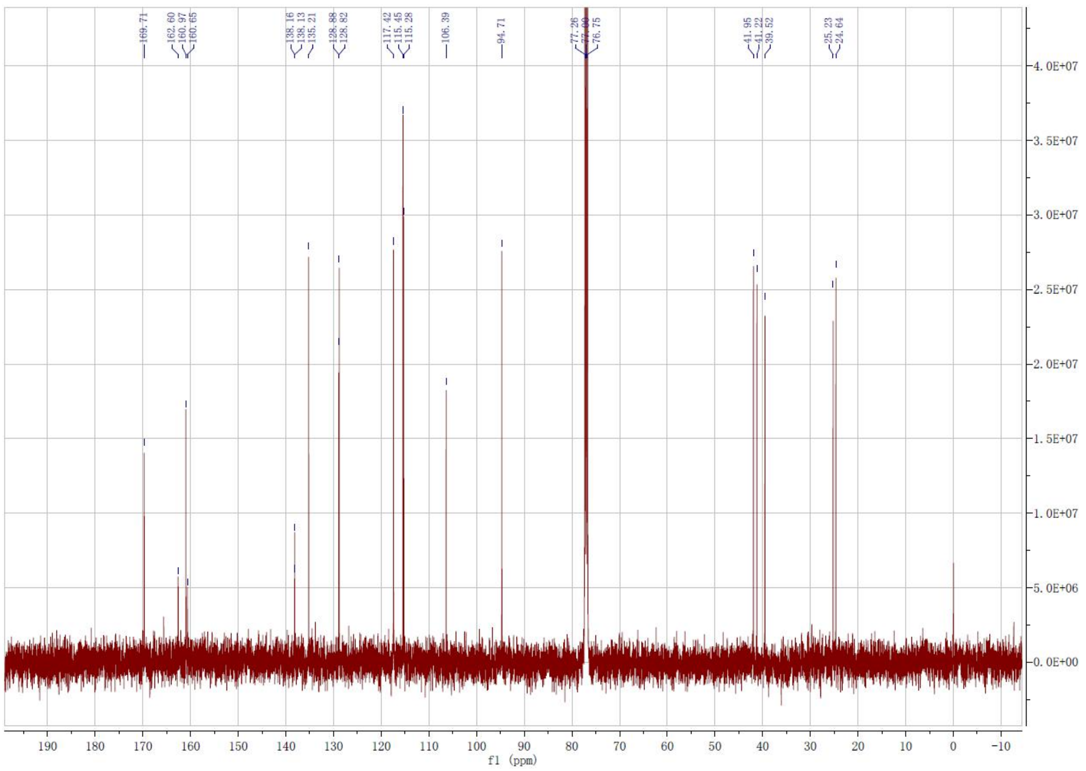

Supplementary Figure 152. ^{13}C NMR spectrum for compound **5d**

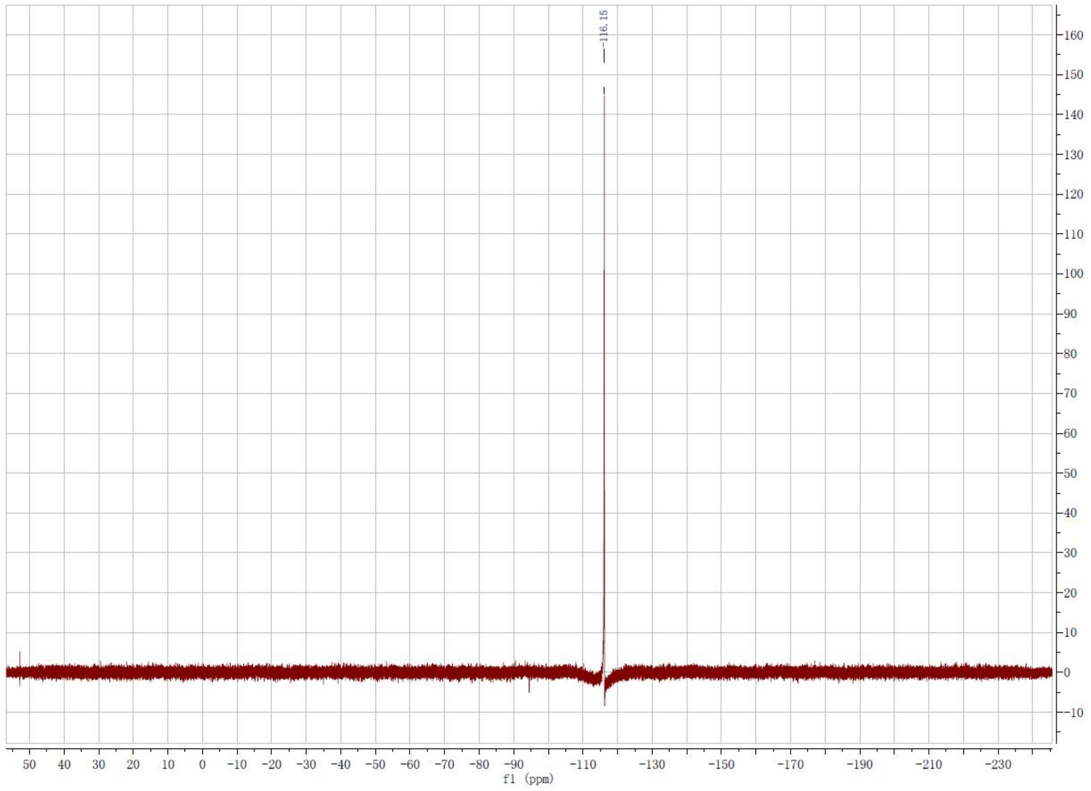

Supplementary Figure 153. ^{19}F NMR spectrum for compound **5d**

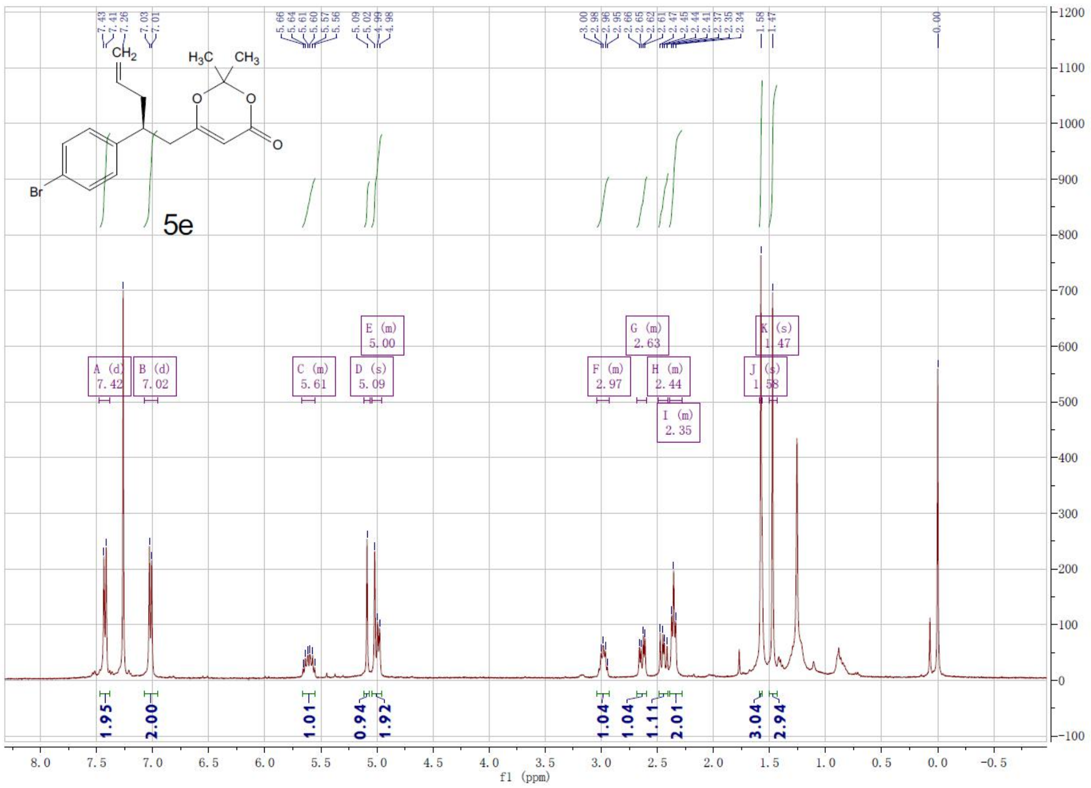

Supplementary Figure 154. ^{1}H NMR spectrum for compound **5e**

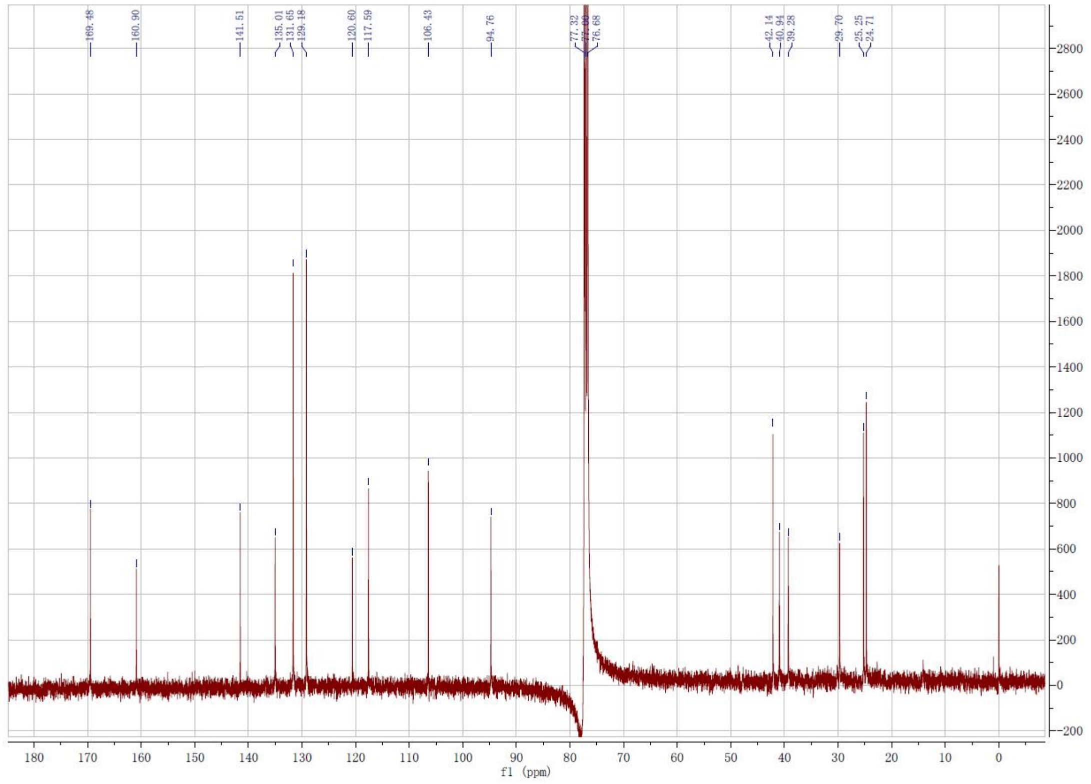

Supplementary Figure 155. ^{13}C NMR spectrum for compound **5e**

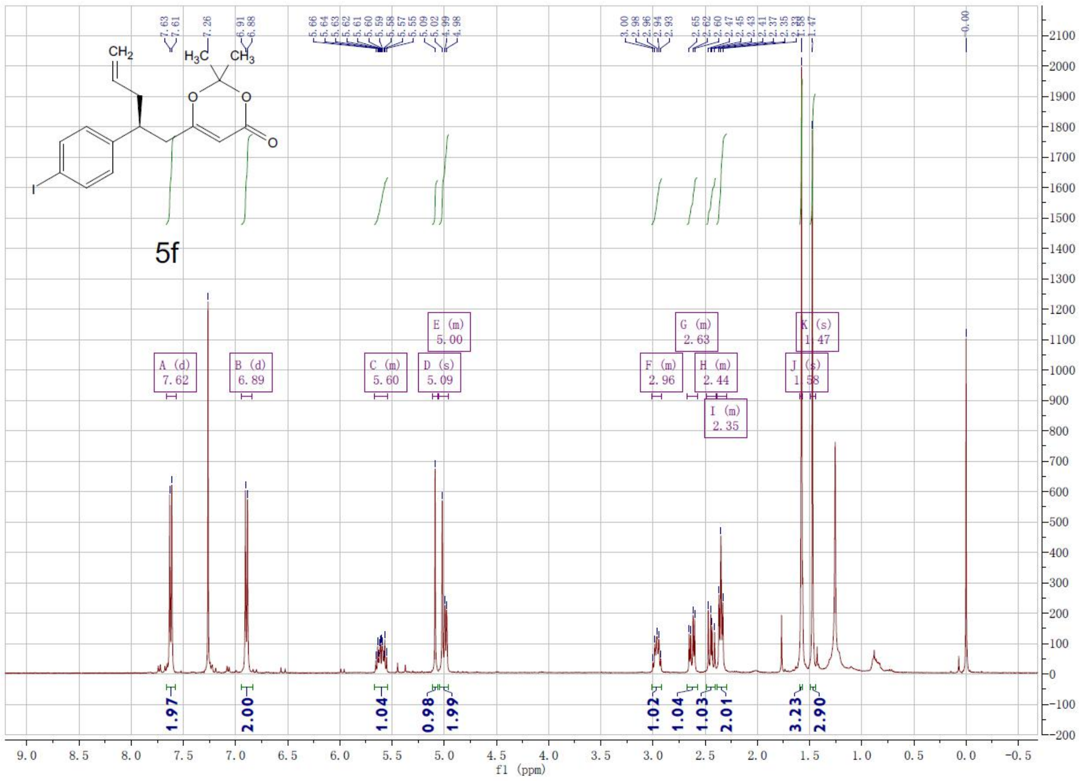

Supplementary Figure 156. ^{1}H NMR spectrum for compound **5f**

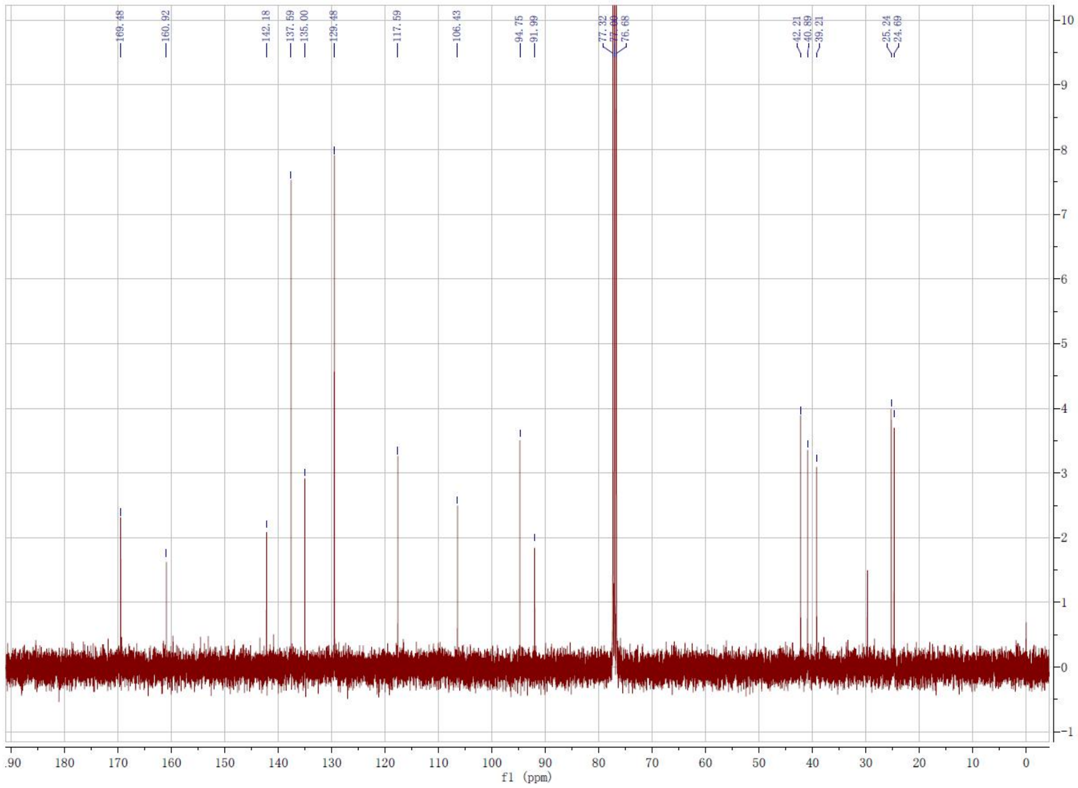

Supplementary Figure 157. ^{13}C NMR spectrum for compound **5f**

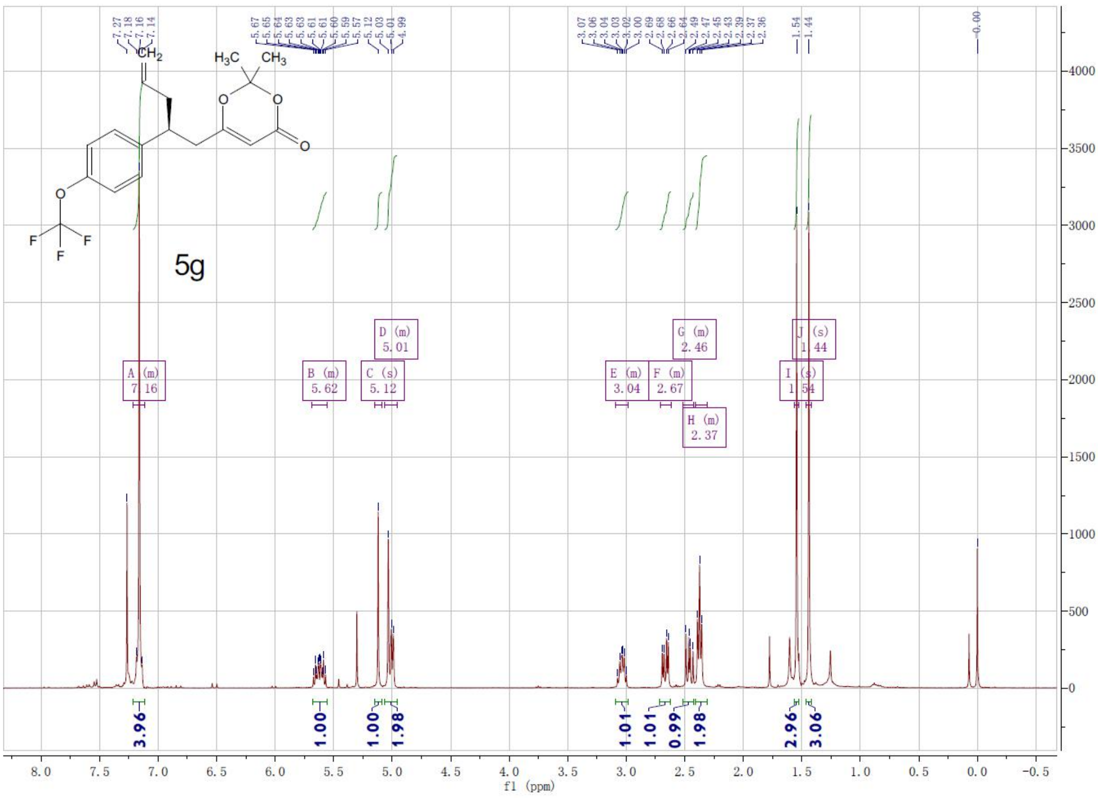

Supplementary Figure 158. ^{1}H NMR spectrum for compound **5g**

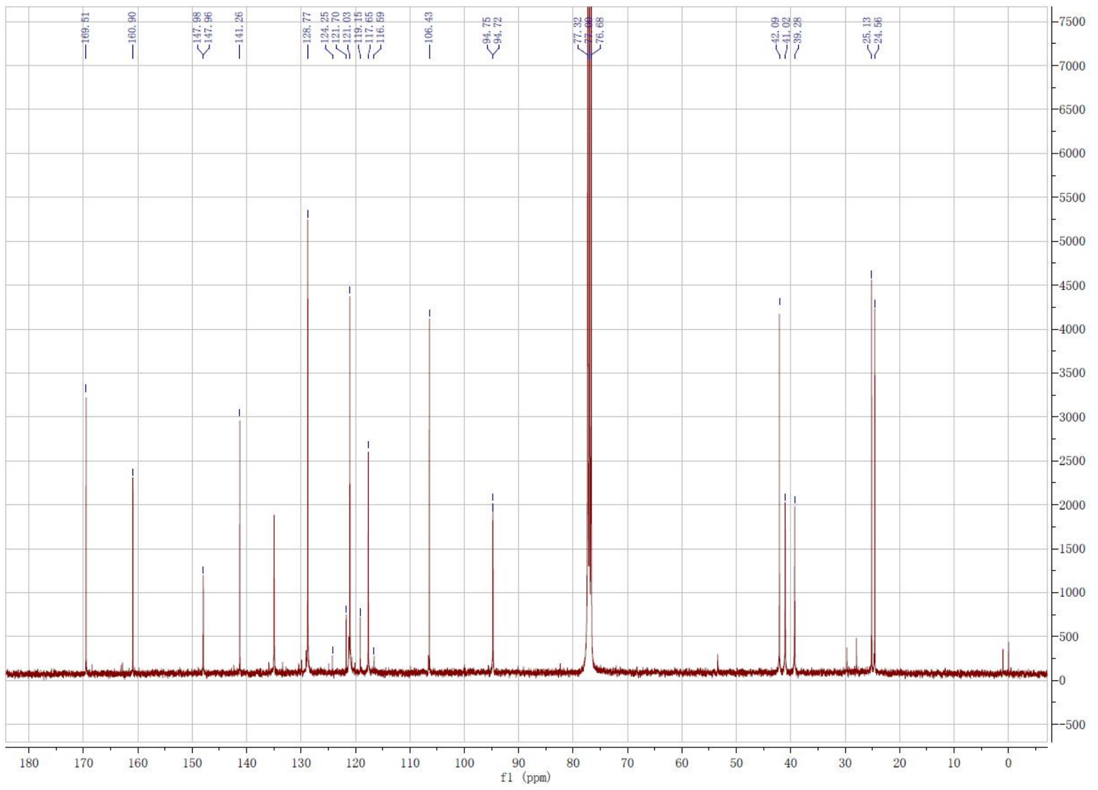

Supplementary Figure 159. ^{13}C NMR spectrum for compound **5g**

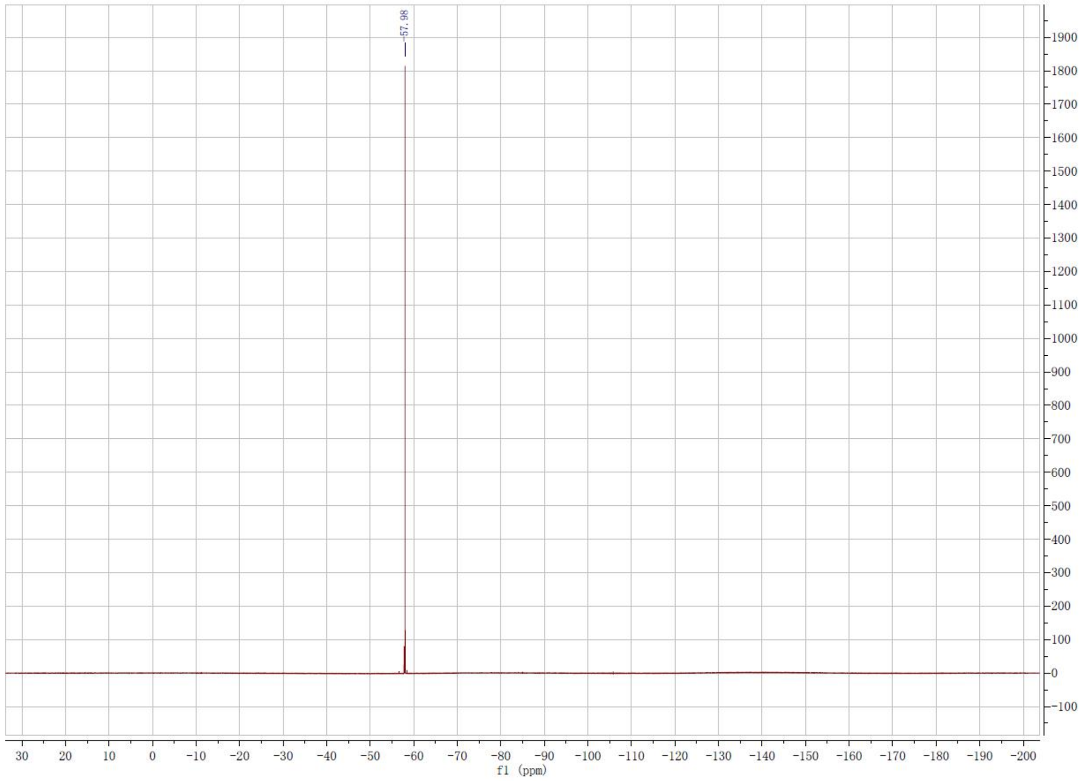

Supplementary Figure 160. ^{19}F NMR spectrum for compound **5g**

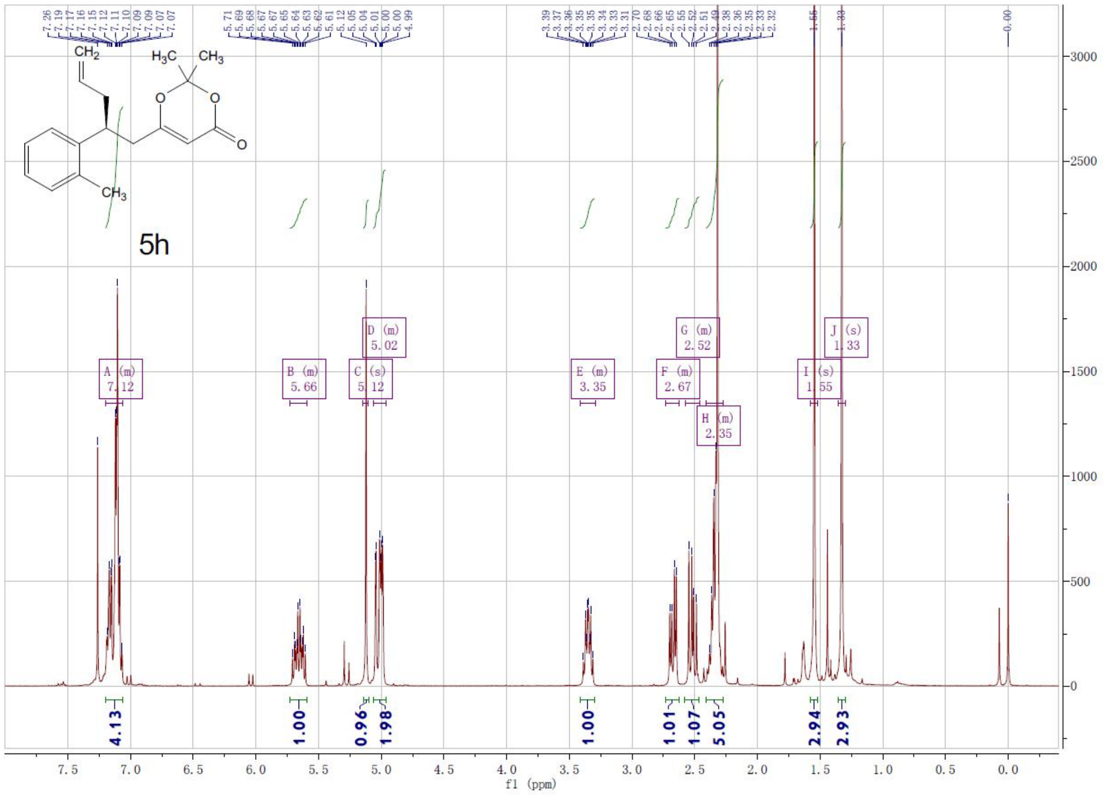

Supplementary Figure 161. ^{1}H NMR spectrum for compound **5h**

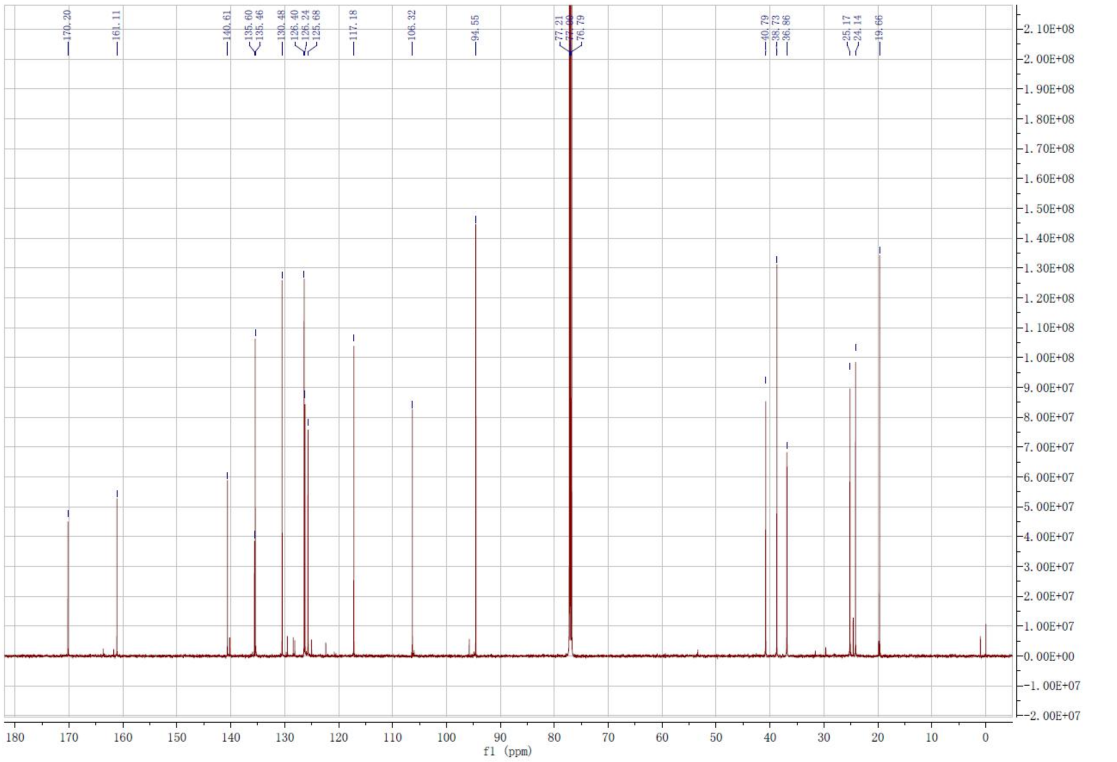

Supplementary Figure 162. ^{13}C NMR spectrum for compound **5h**

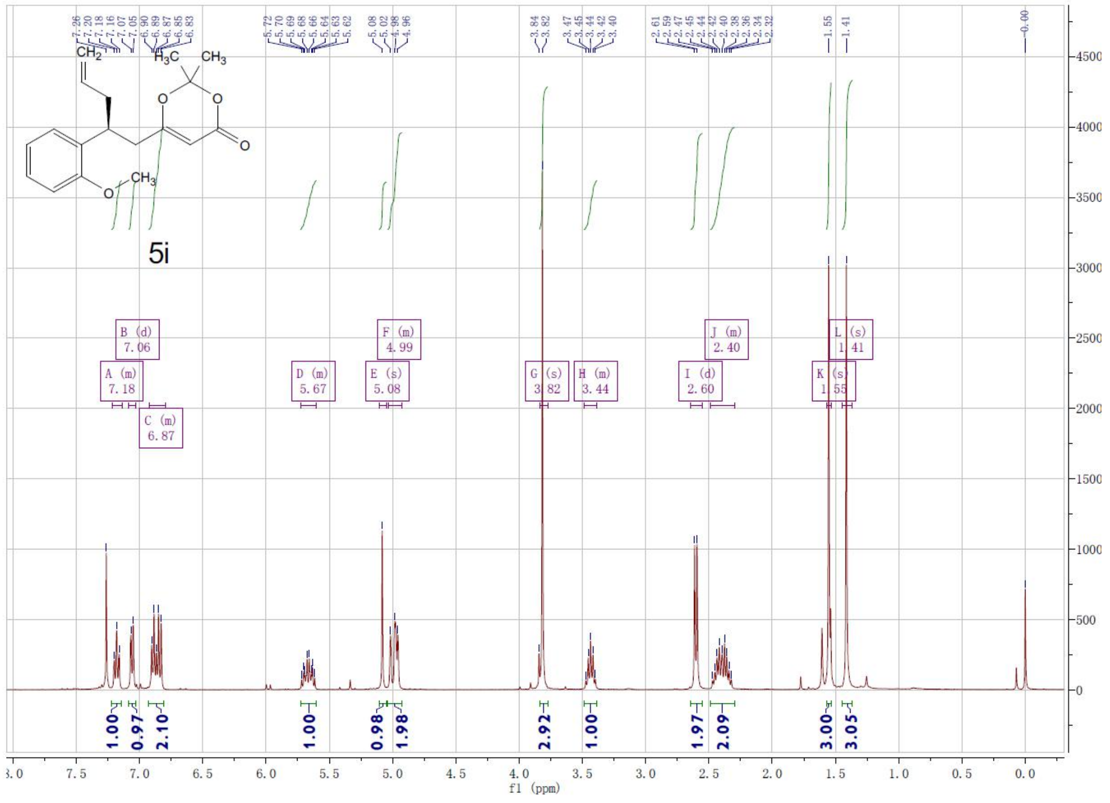

Supplementary Figure 163. ^{1}H NMR spectrum for compound **5i**

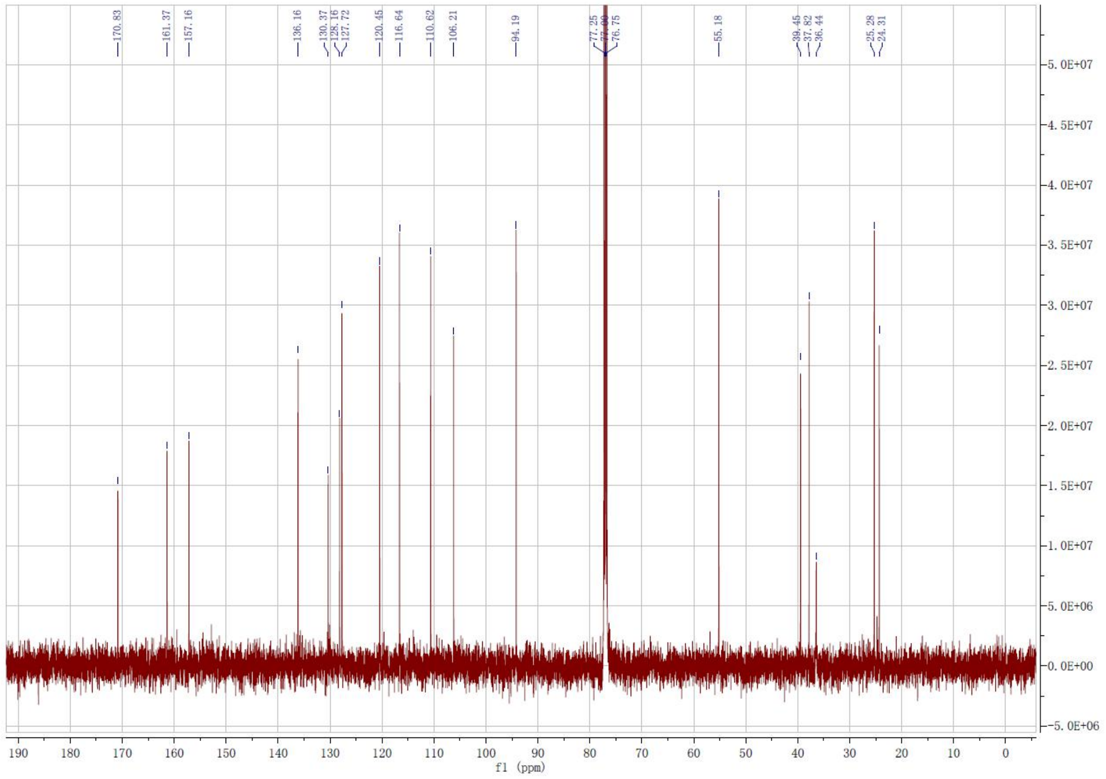

Supplementary Figure 164. ^{13}C NMR spectrum for compound **5i**

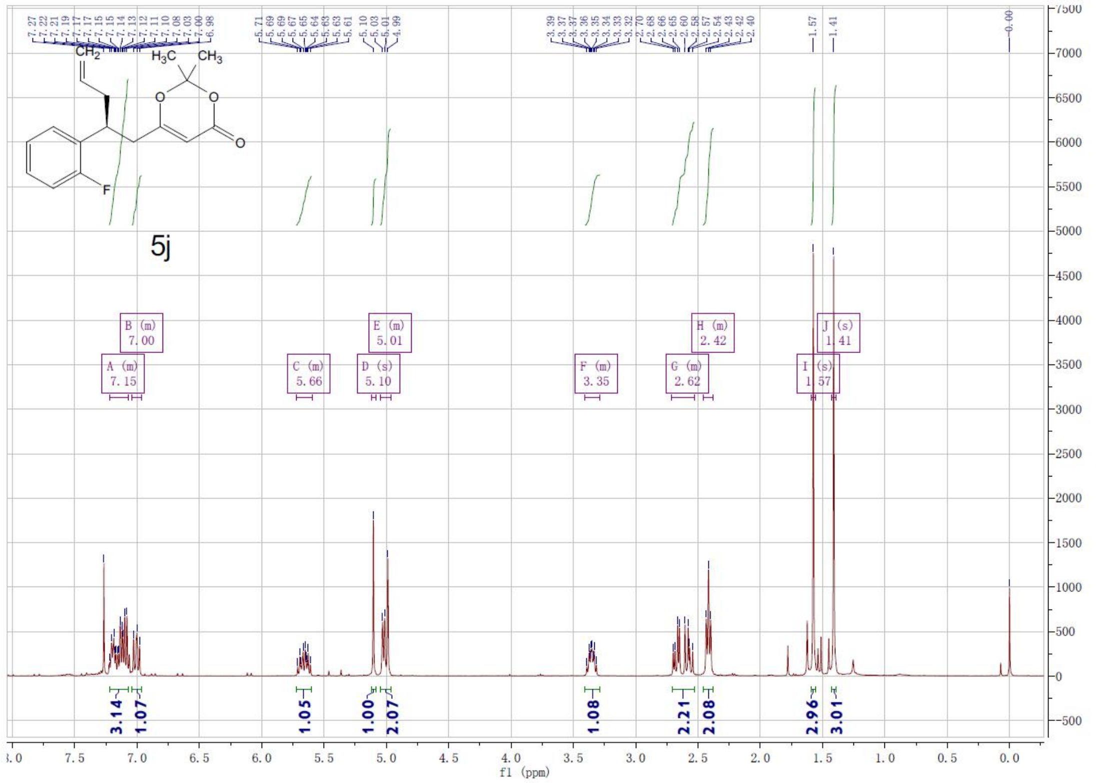

Supplementary Figure 165. ^{1}H NMR spectrum for compound **5j**

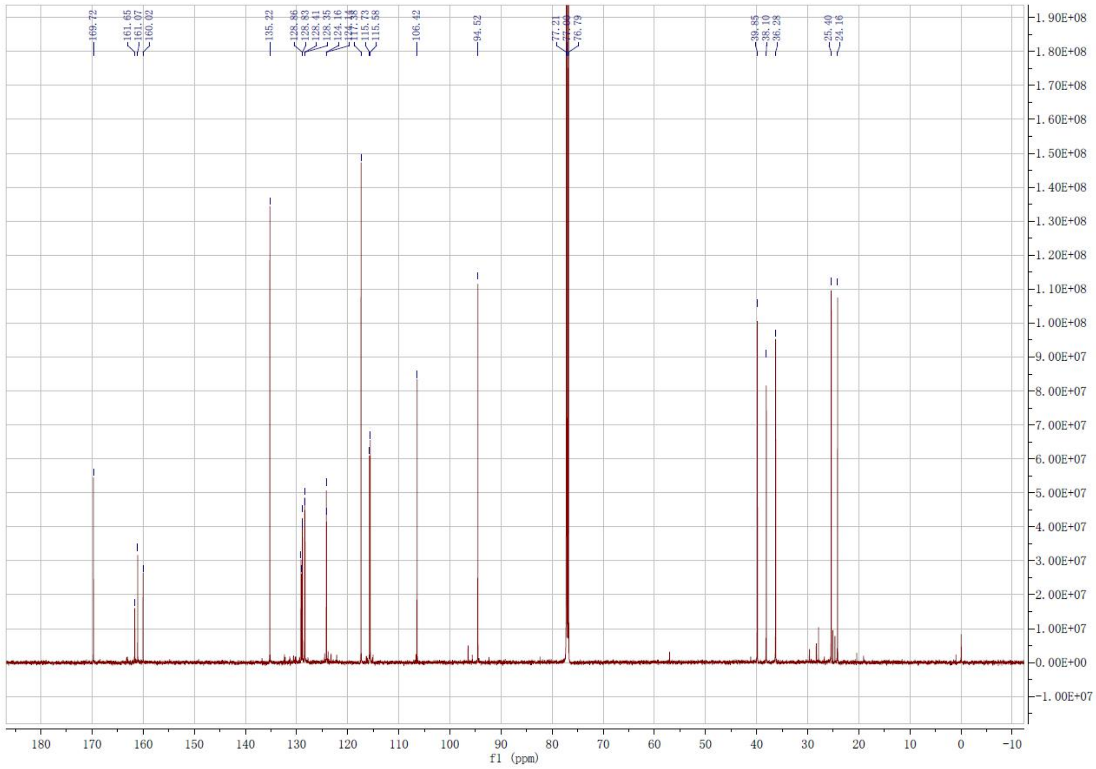

Supplementary Figure 166. ^{13}C NMR spectrum for compound **5j**

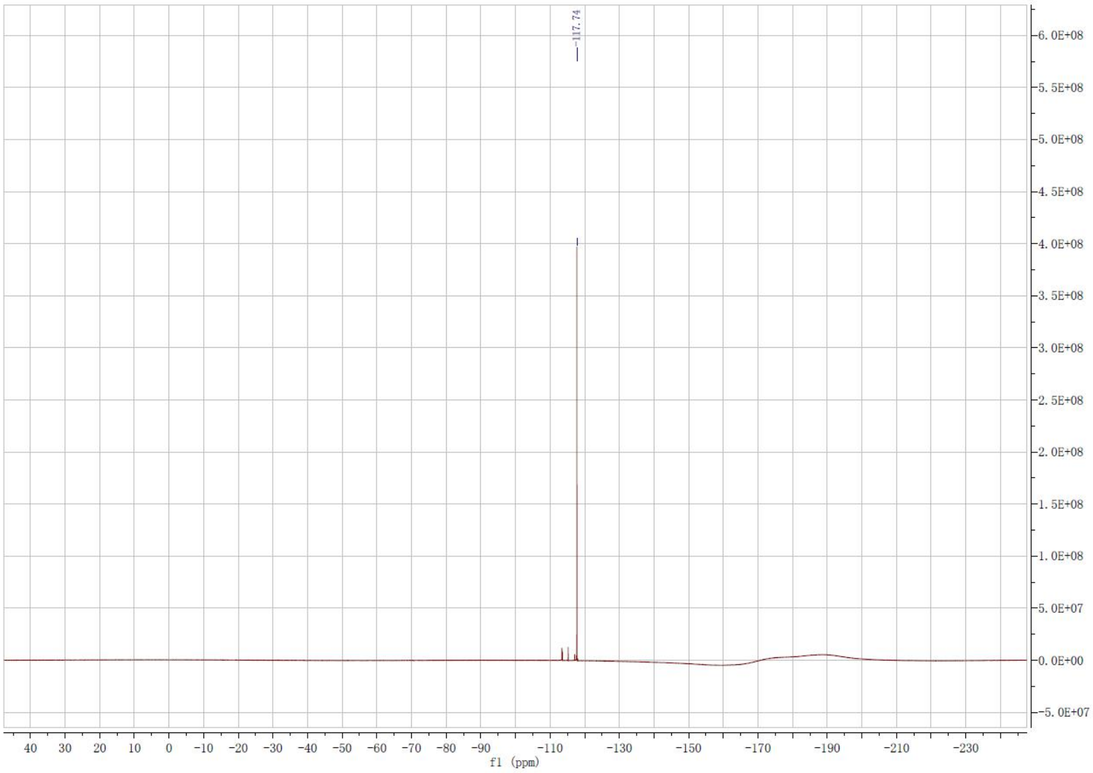

Supplementary Figure 167. ^{19}F NMR spectrum for compound **5j**

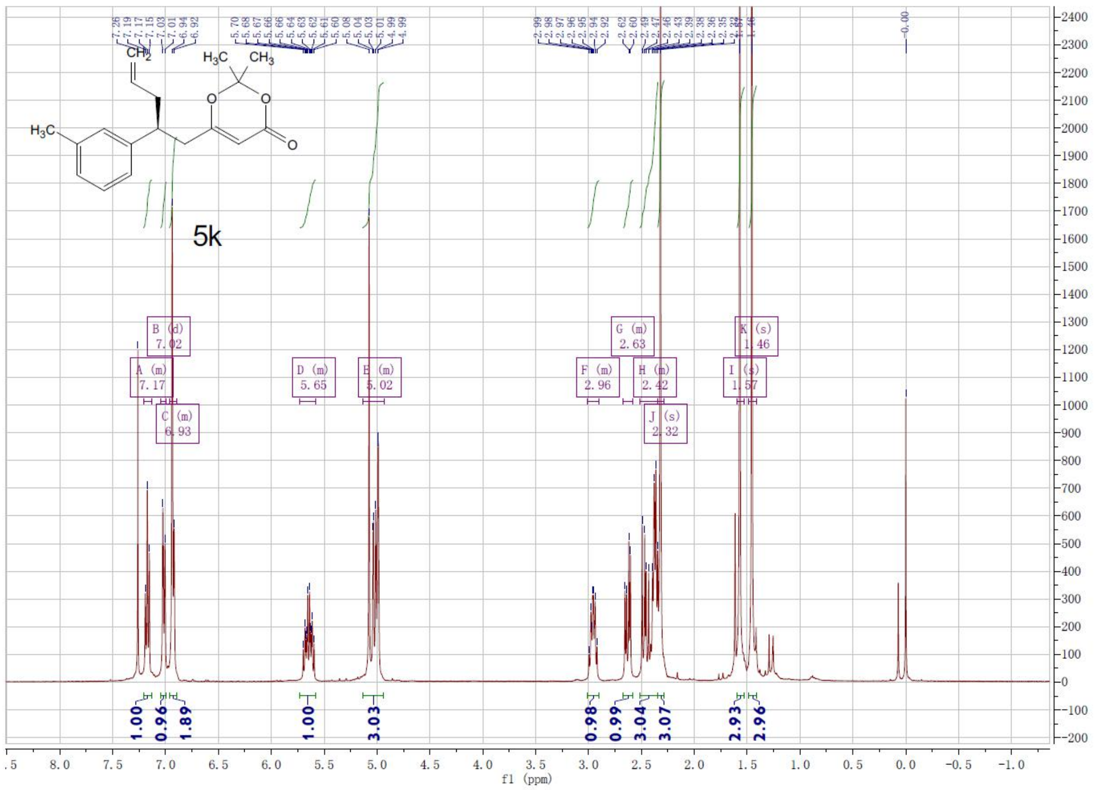

Supplementary Figure 168. ^{1}H NMR spectrum for compound **5k**

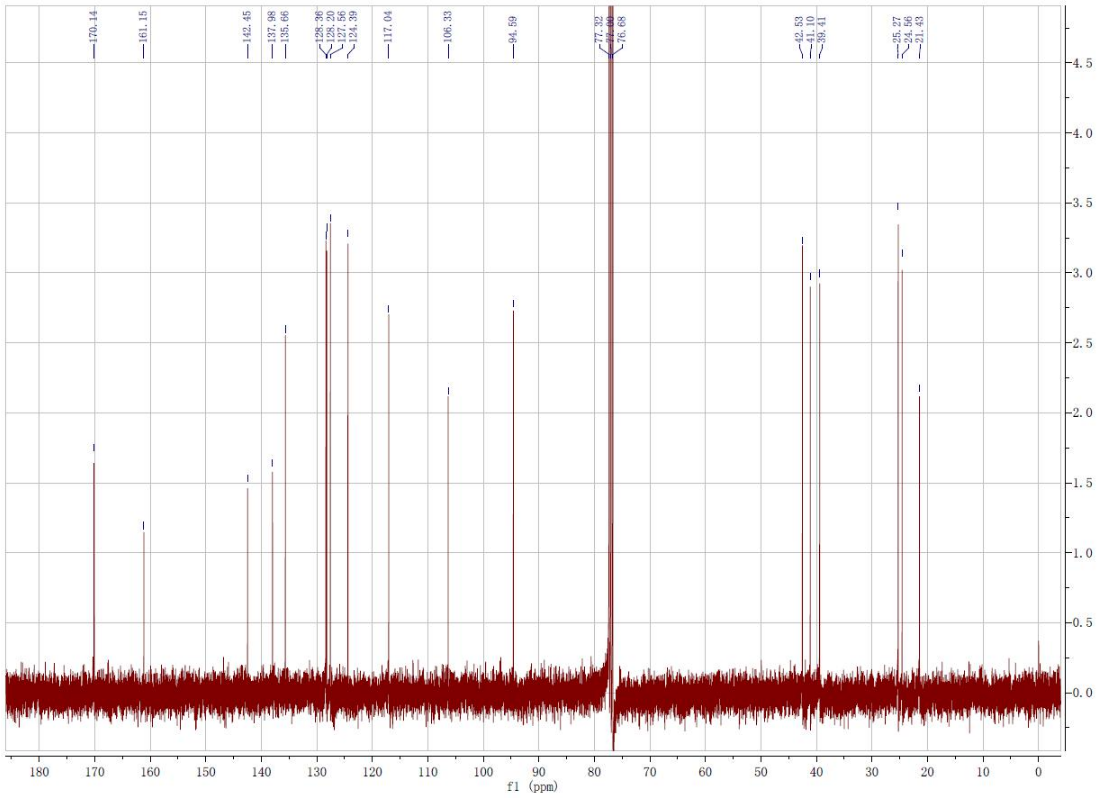

Supplementary Figure 169. ^{13}C NMR spectrum for compound **5k**

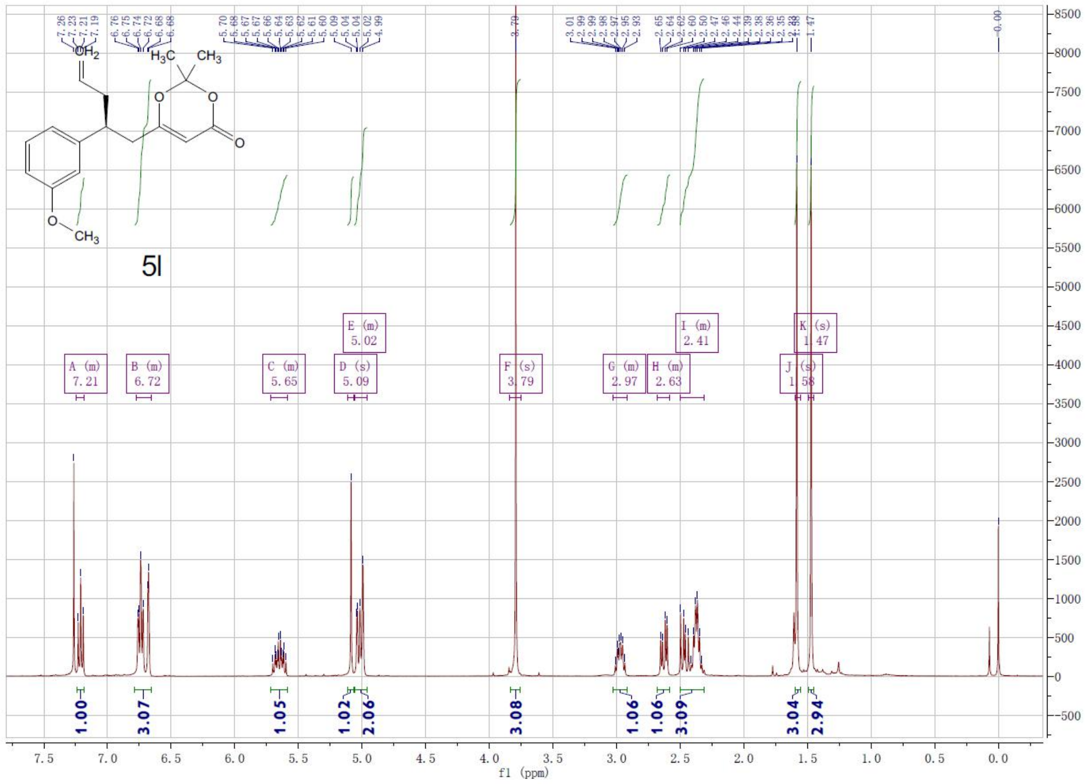

Supplementary Figure 170. ^{1}H NMR spectrum for compound **5l**

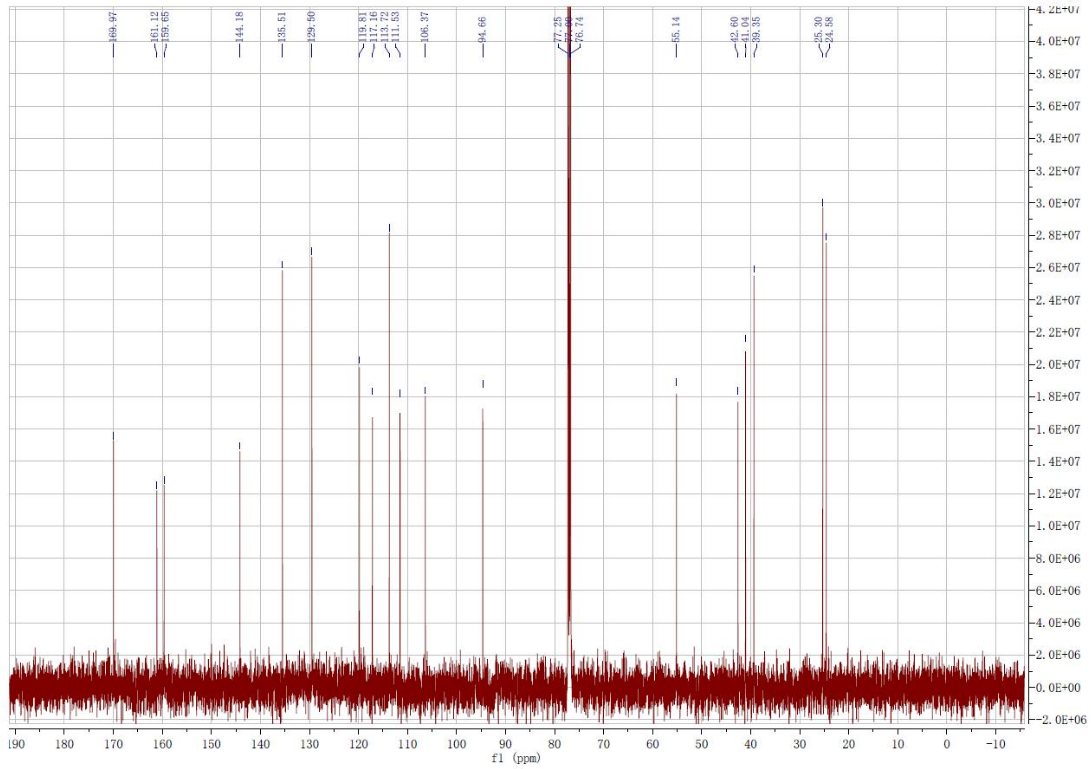

Supplementary Figure 171. ^{13}C NMR spectrum for compound **5l**

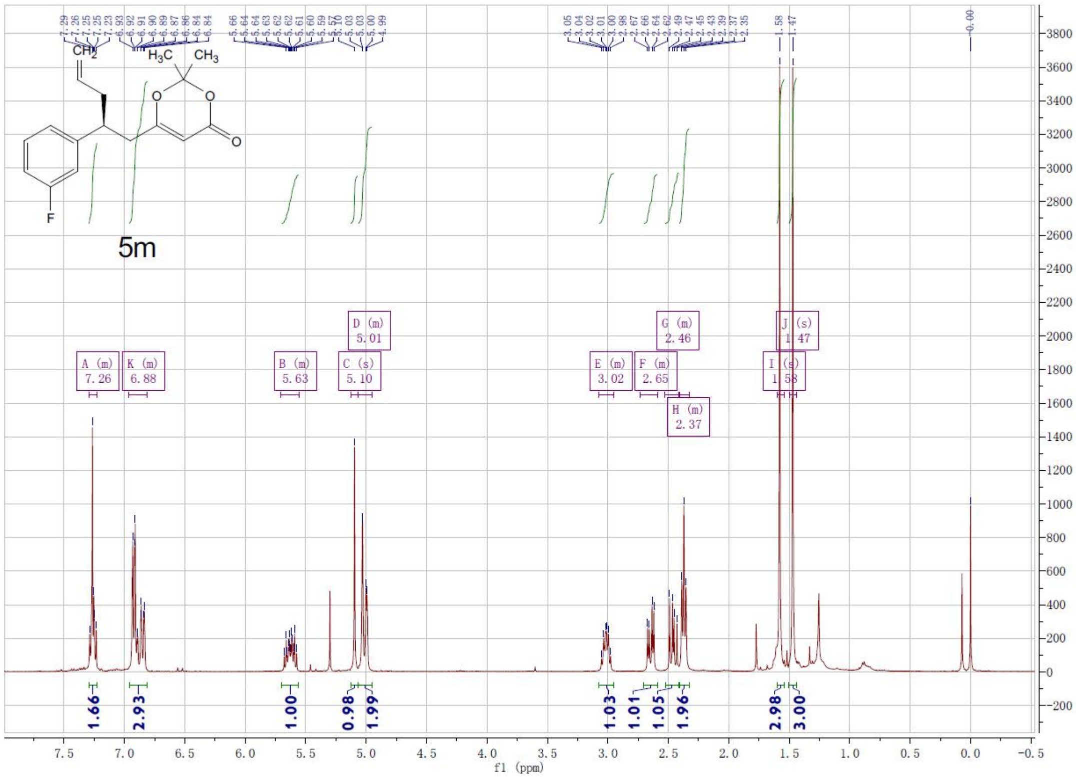

Supplementary Figure 172. ^{1}H NMR spectrum for compound **5m**

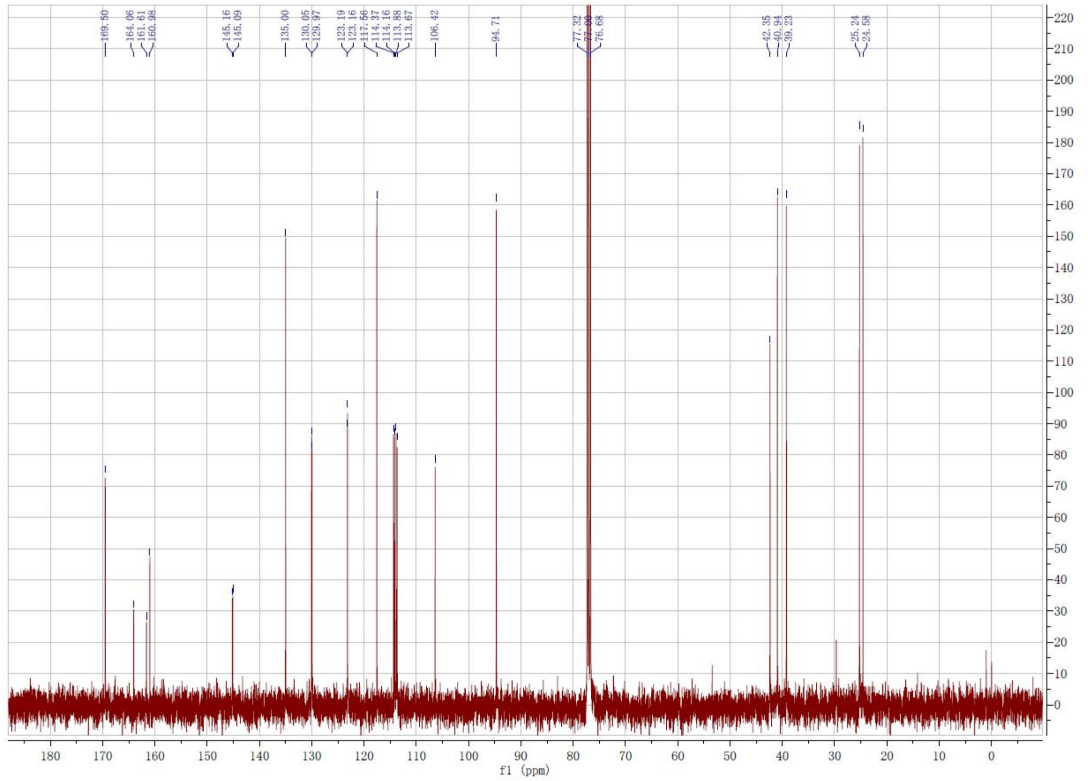

Supplementary Figure 173. ^{13}C NMR spectrum for compound **5m**

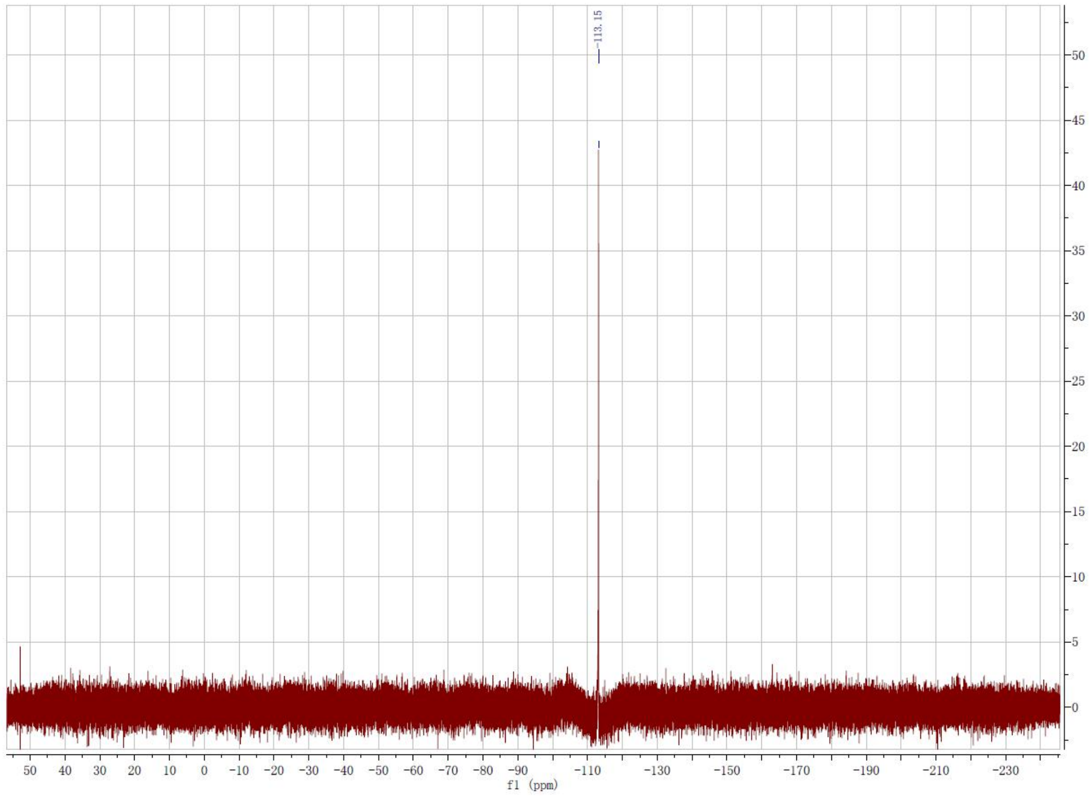

Supplementary Figure 174. ^{19}F NMR spectrum for compound **5m**

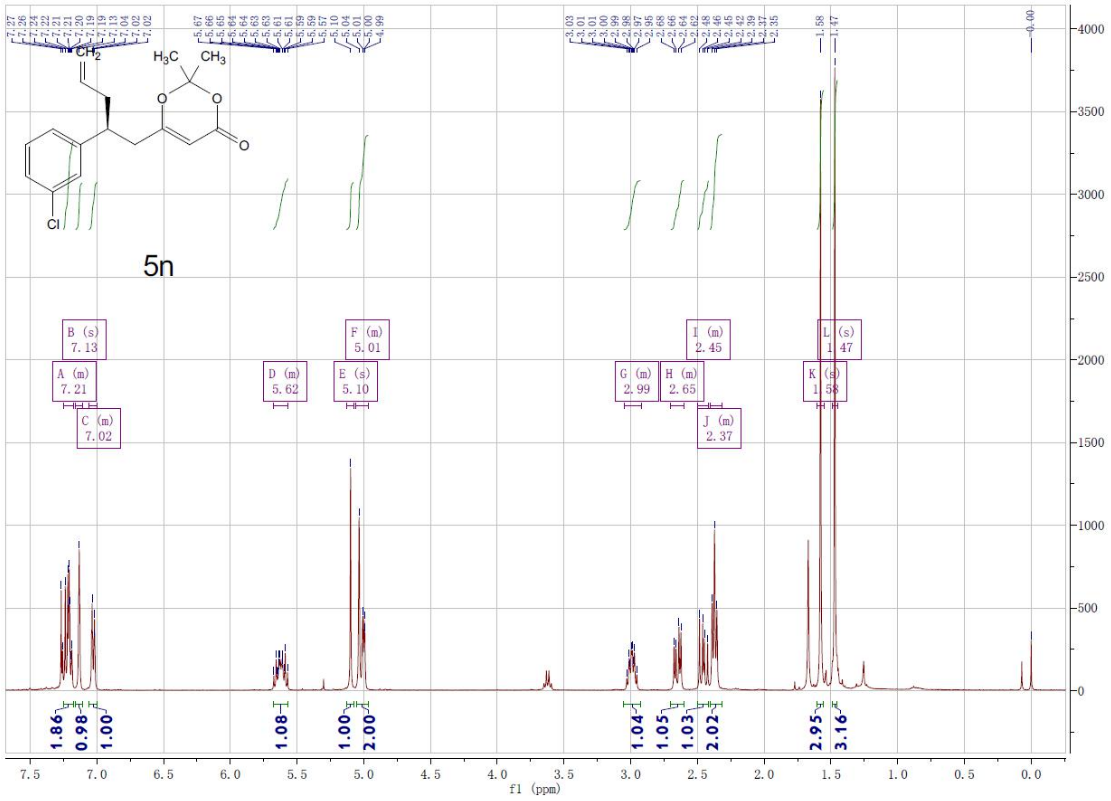

Supplementary Figure 175. ^{1}H NMR spectrum for compound **5n**

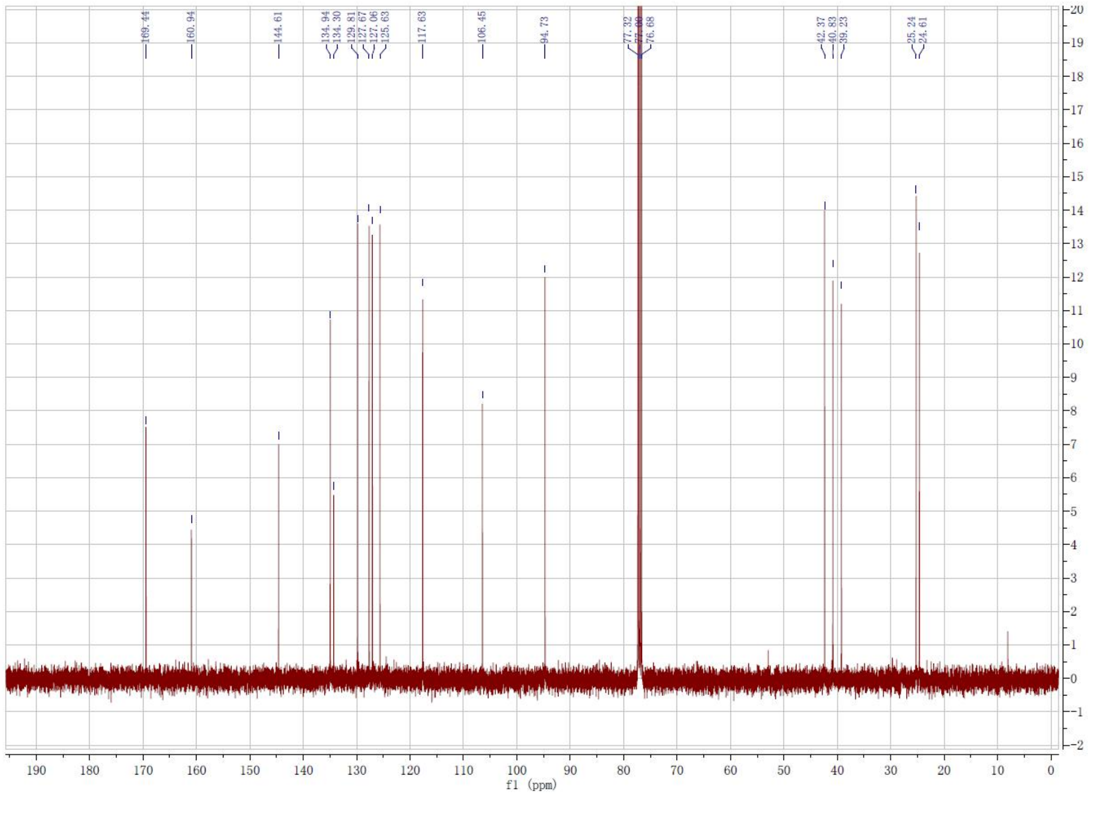

Supplementary Figure 176. ^{13}C NMR spectrum for compound **5n**

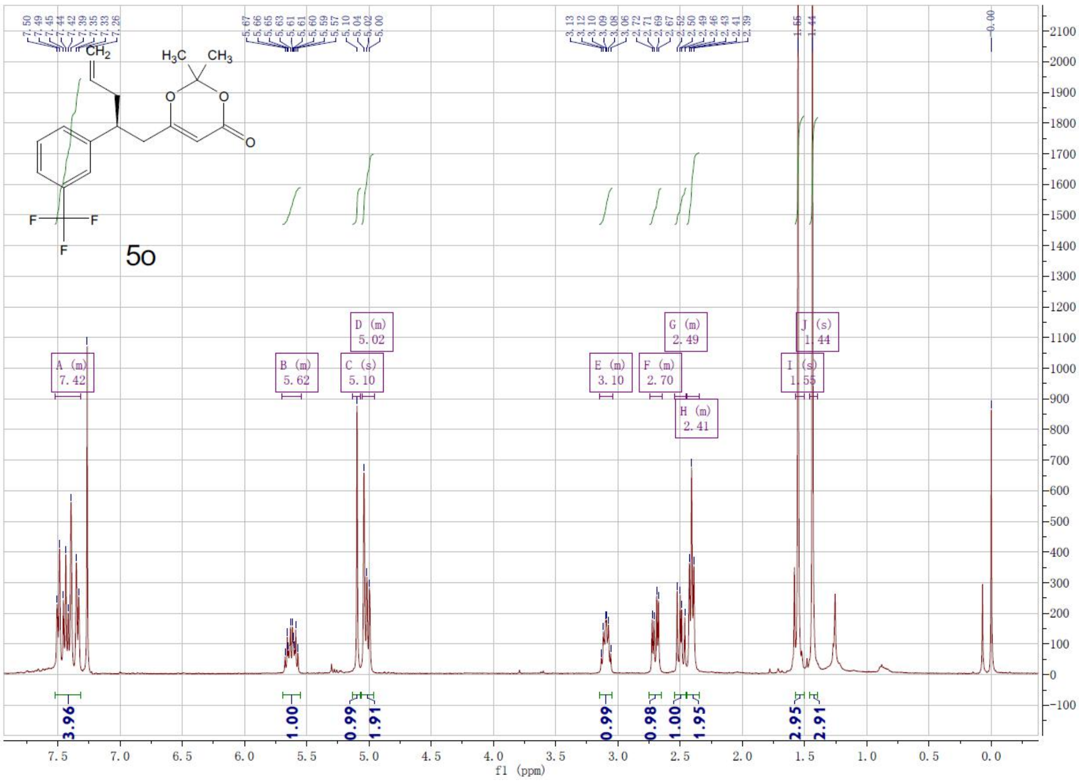

Supplementary Figure 177. ^{1}H NMR spectrum for compound **5o**

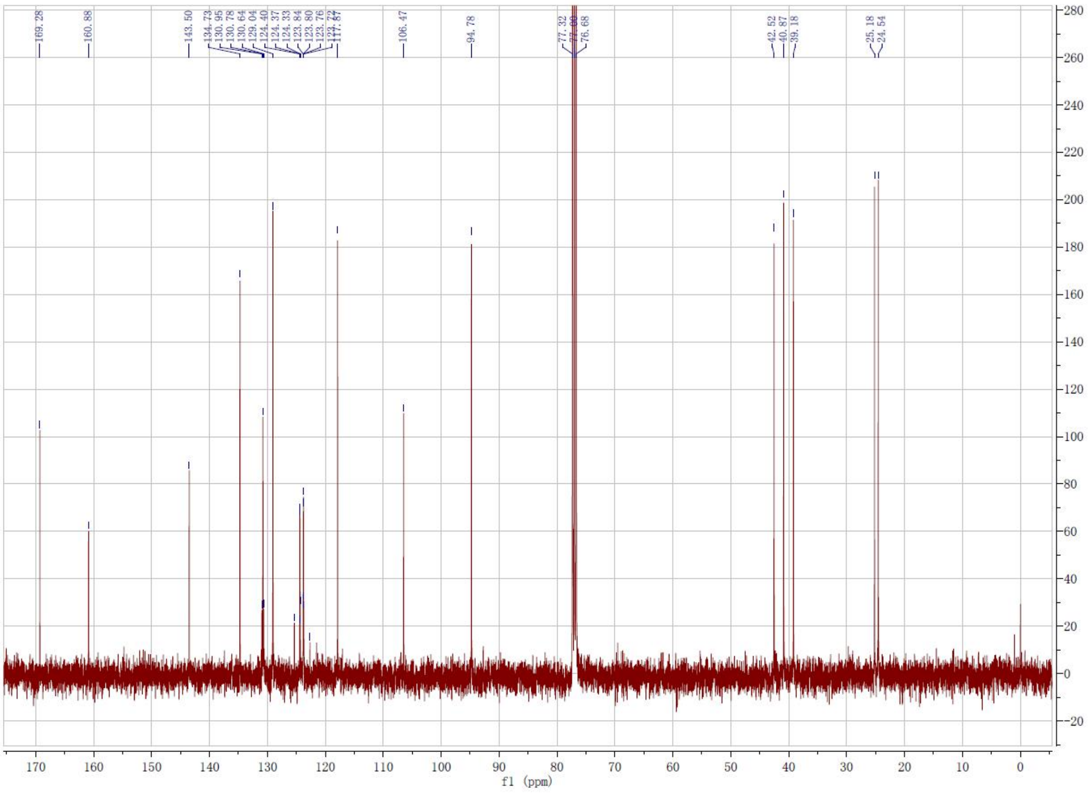

Supplementary Figure 178. ^{13}C NMR spectrum for compound **5o**

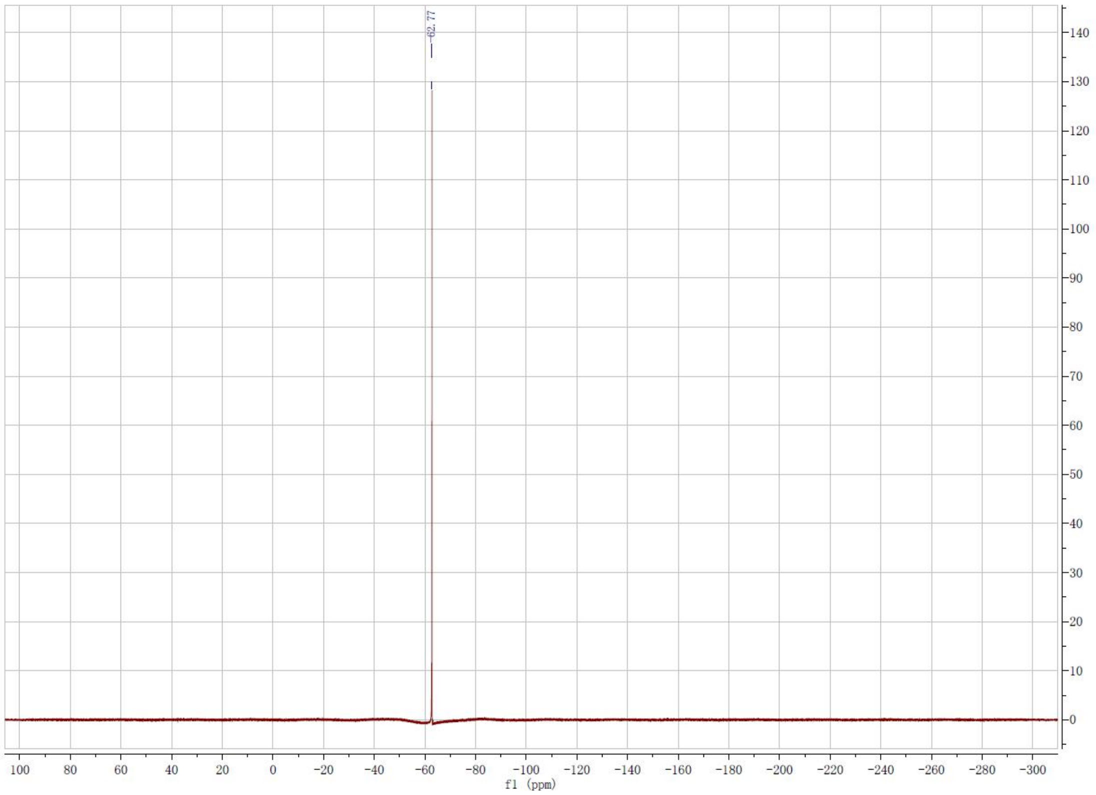

Supplementary Figure 179. ^{19}F NMR spectrum for compound **5o**

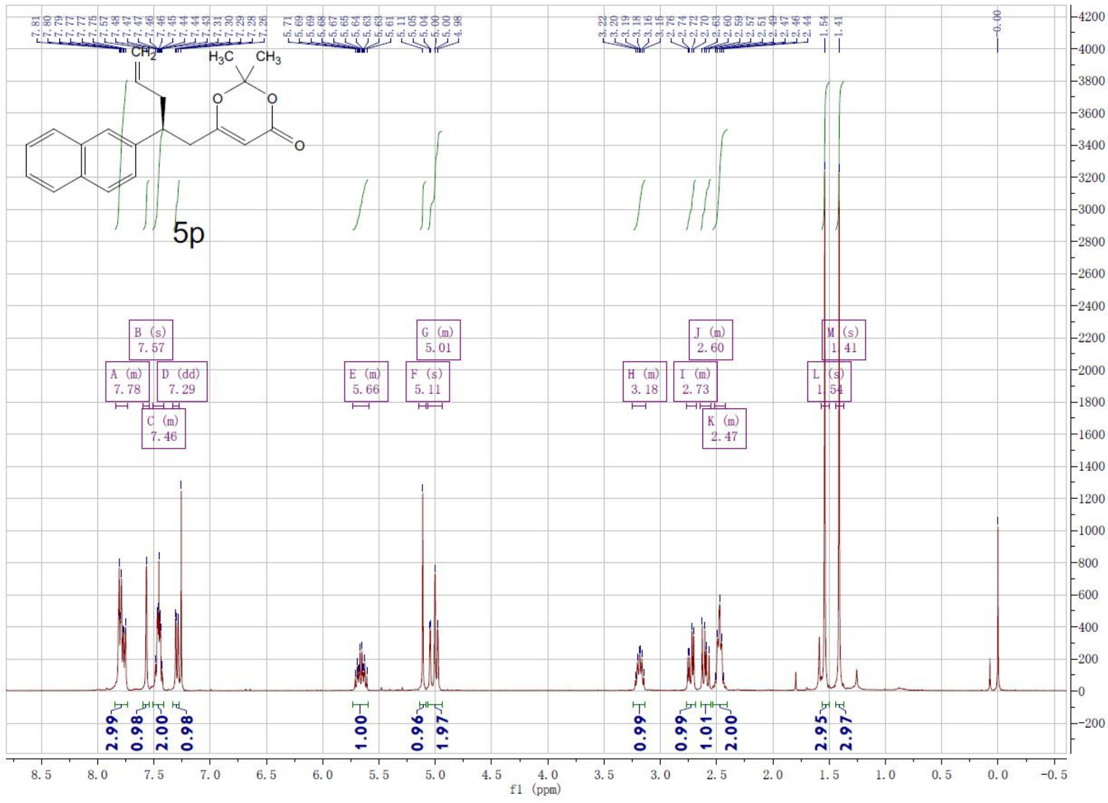

Supplementary Figure 180. ^{1}H NMR spectrum for compound **5p**

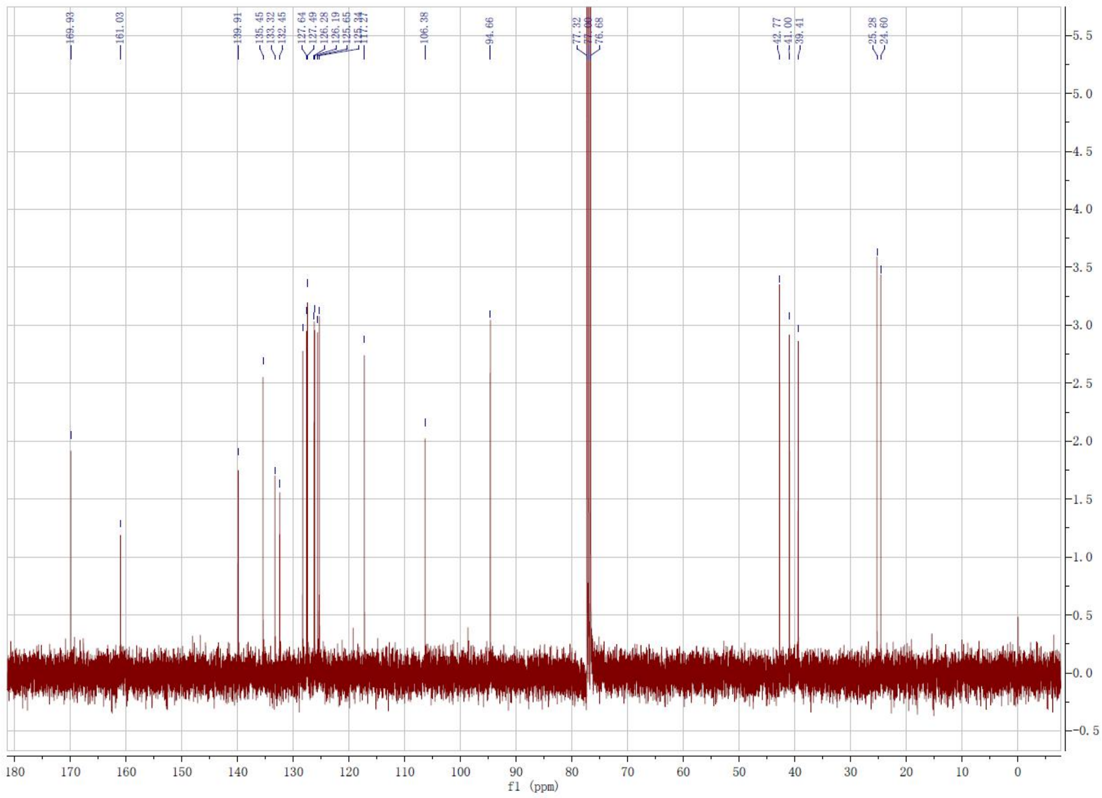

Supplementary Figure 181. ^{13}C NMR spectrum for compound **5p**

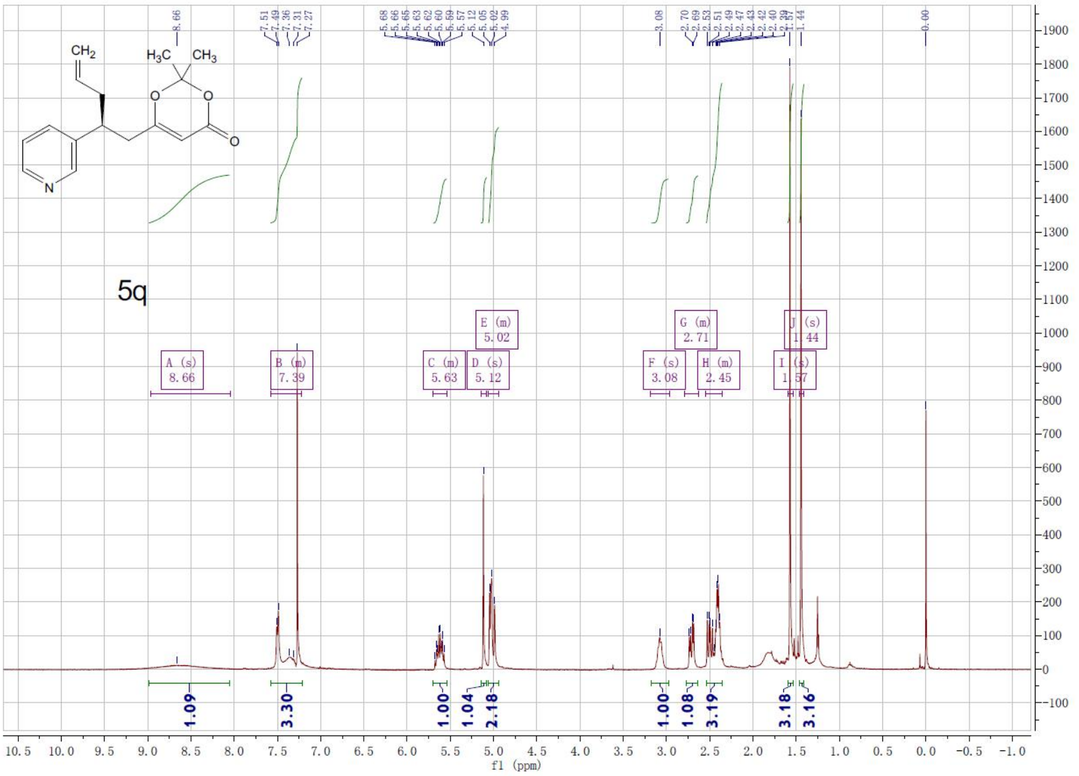

Supplementary Figure 182. ^{1}H NMR spectrum for compound **5q**

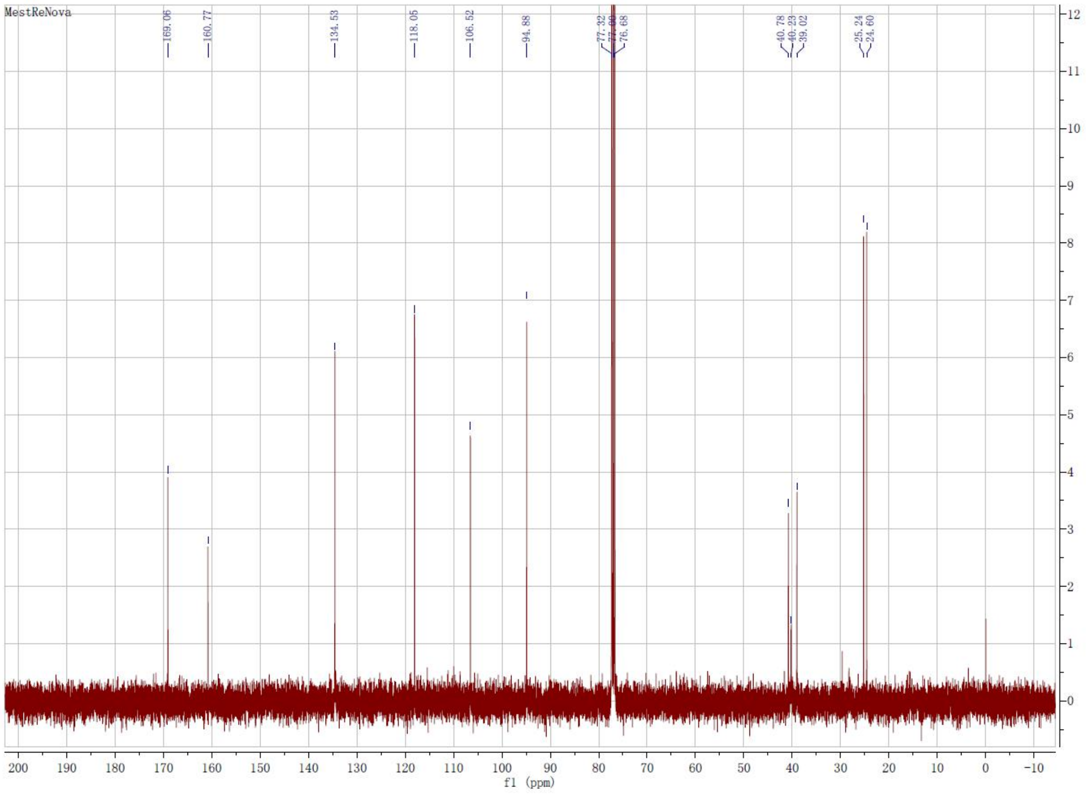

Supplementary Figure 183. ^{13}C NMR spectrum for compound **5q**

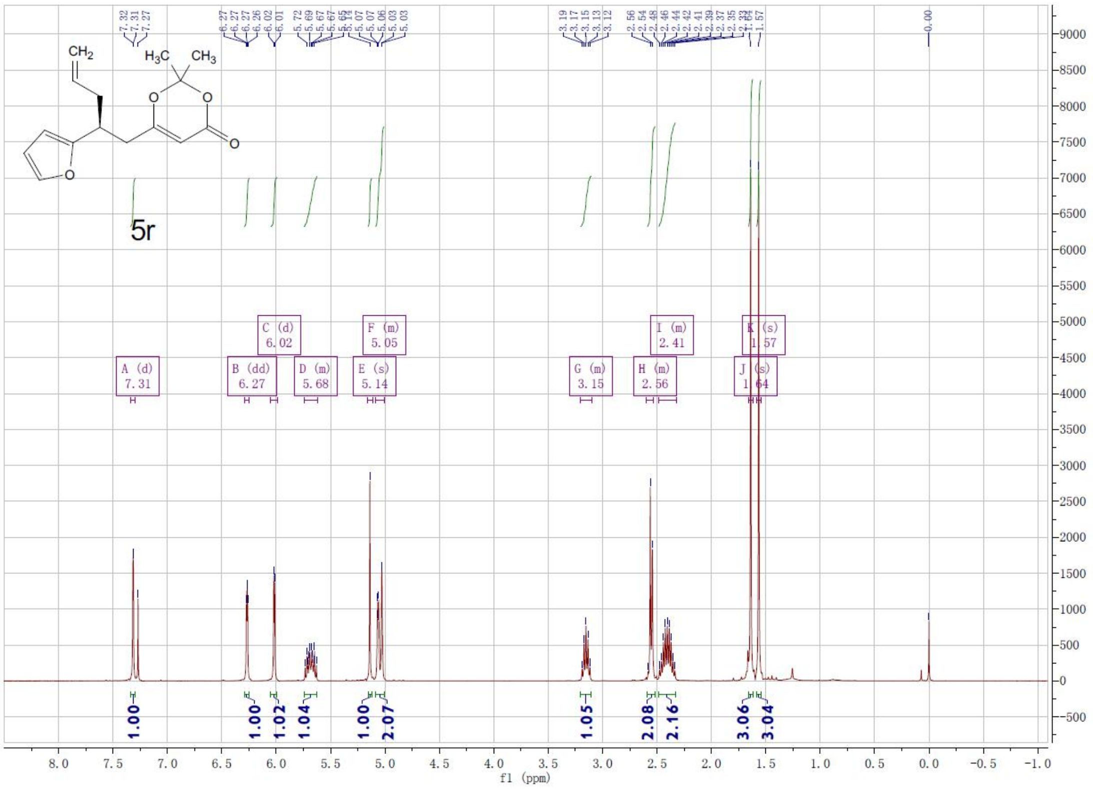

Supplementary Figure 184. ^{1}H NMR spectrum for compound **5r**

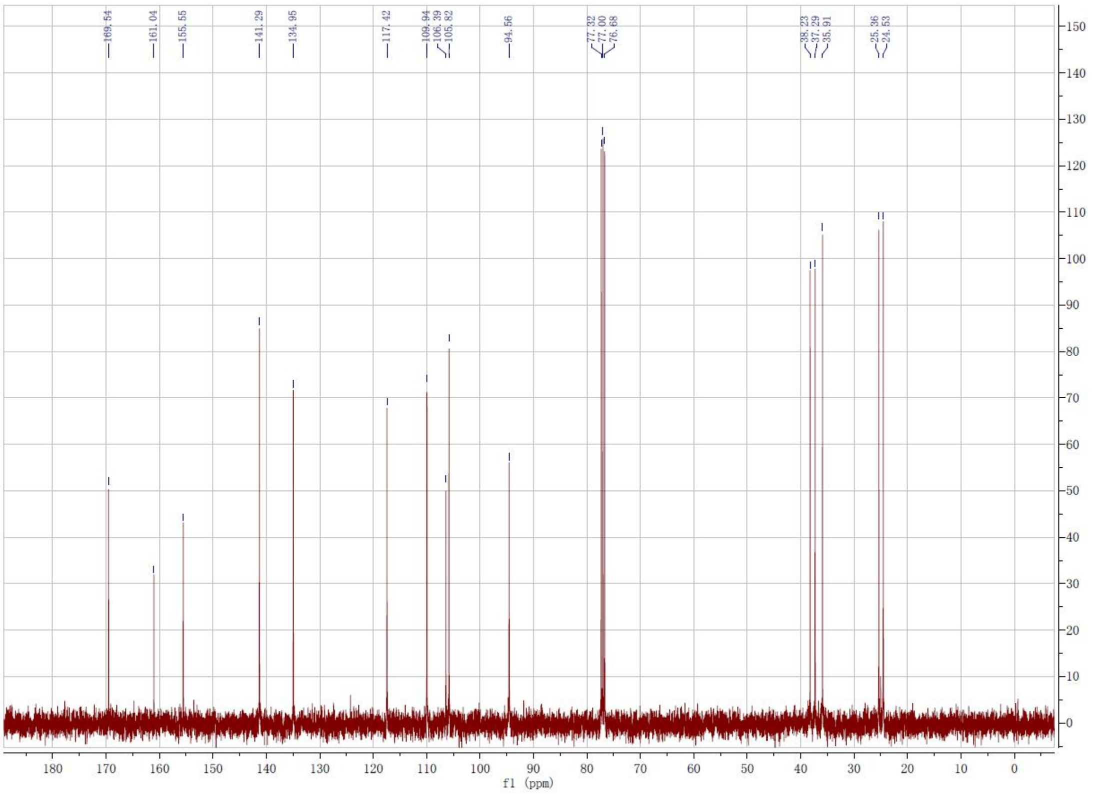

Supplementary Figure 185. ^{13}C NMR spectrum for compound **5r**

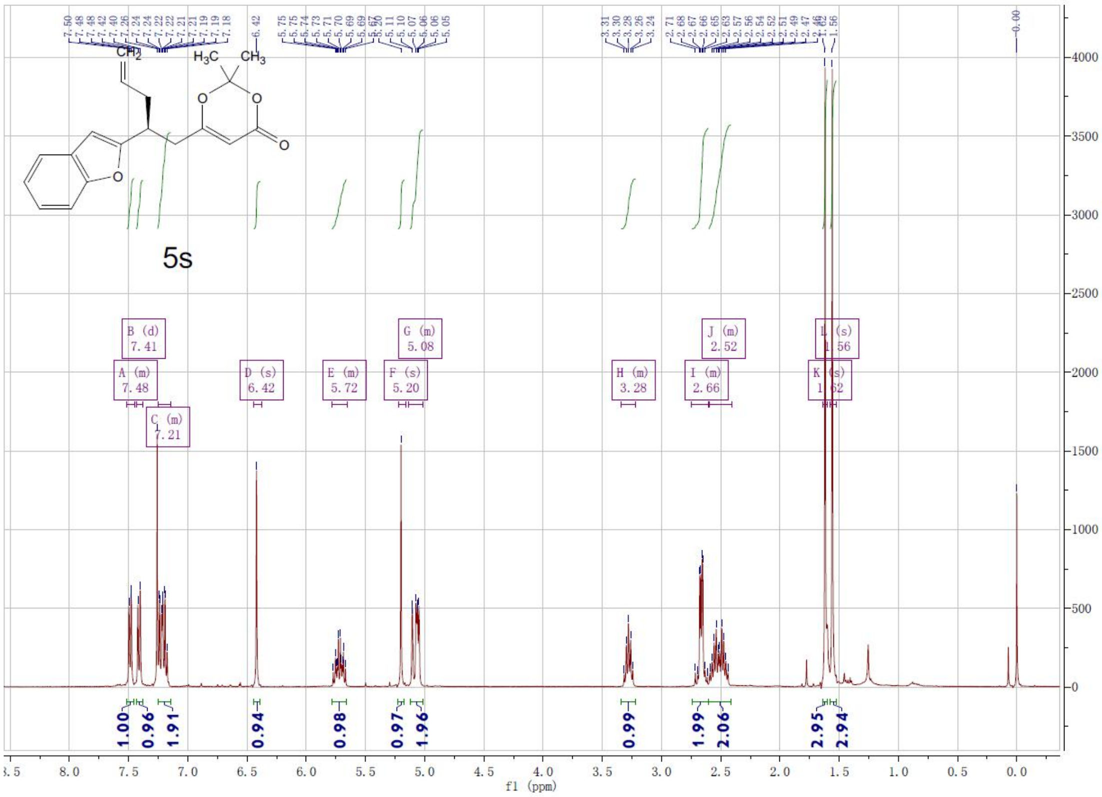

Supplementary Figure 186. ^{1}H NMR spectrum for compound **5s**

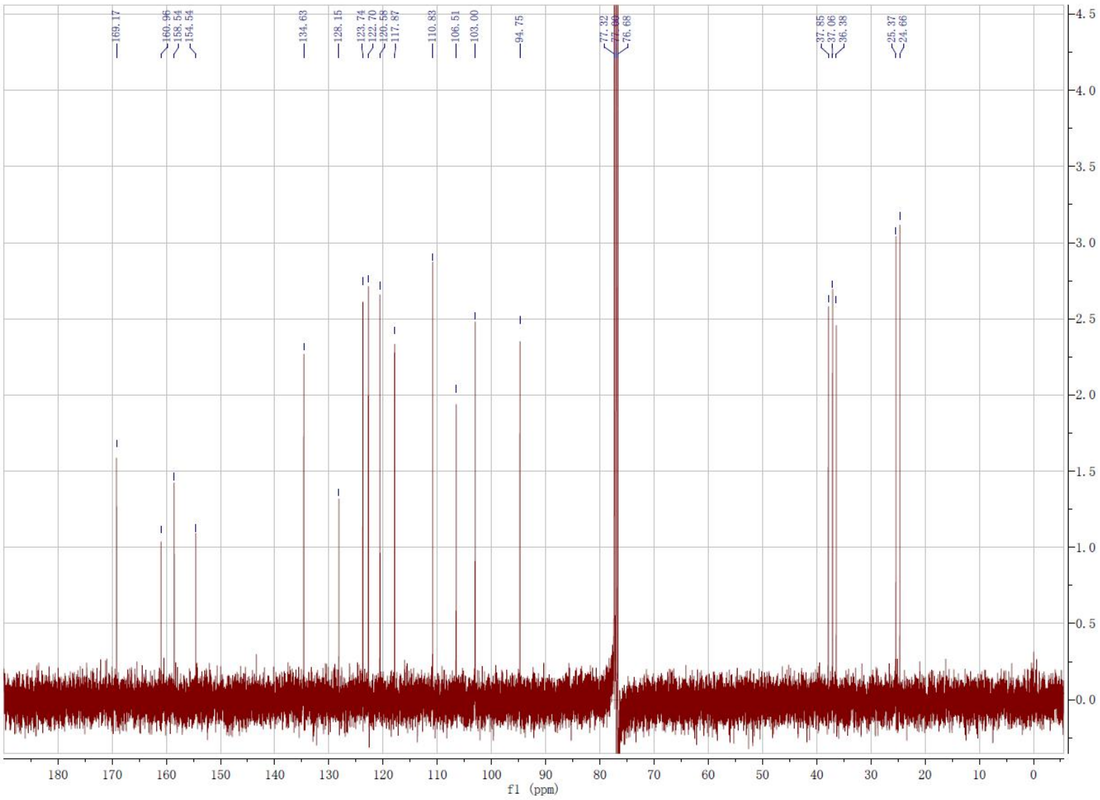

Supplementary Figure 187. ^{13}C NMR spectrum for compound **5s**

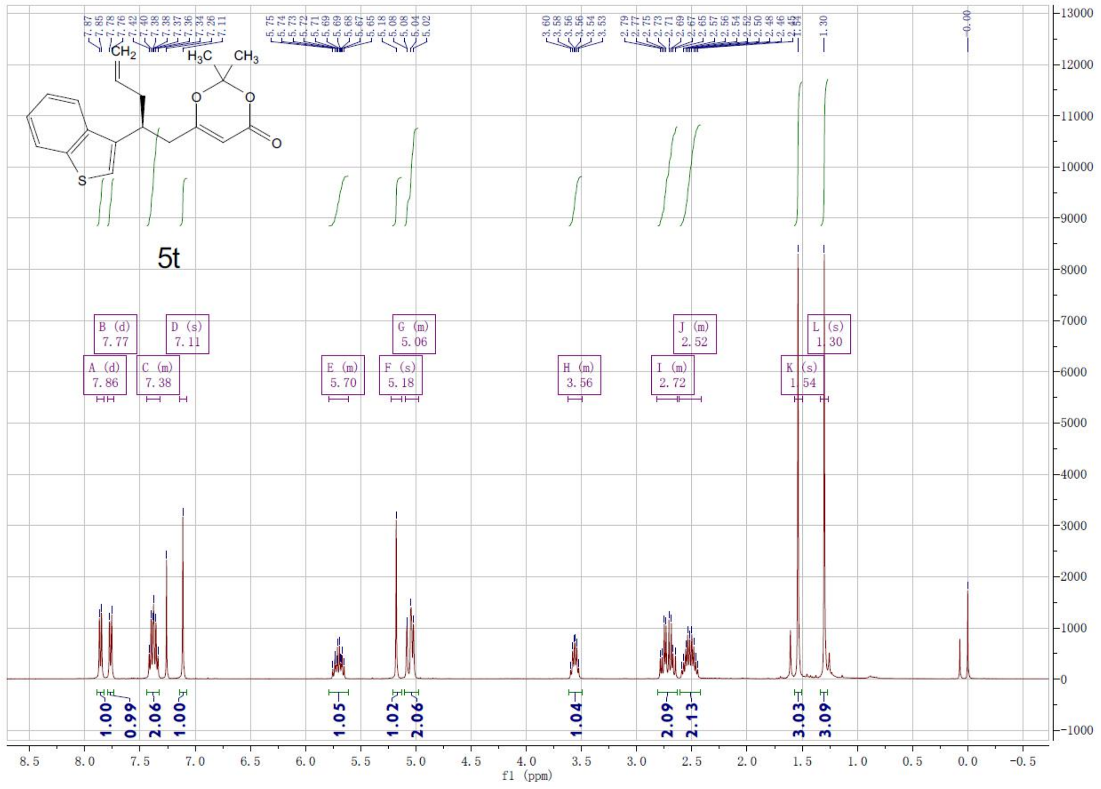

Supplementary Figure 188. ^{1}H NMR spectrum for compound **5t**

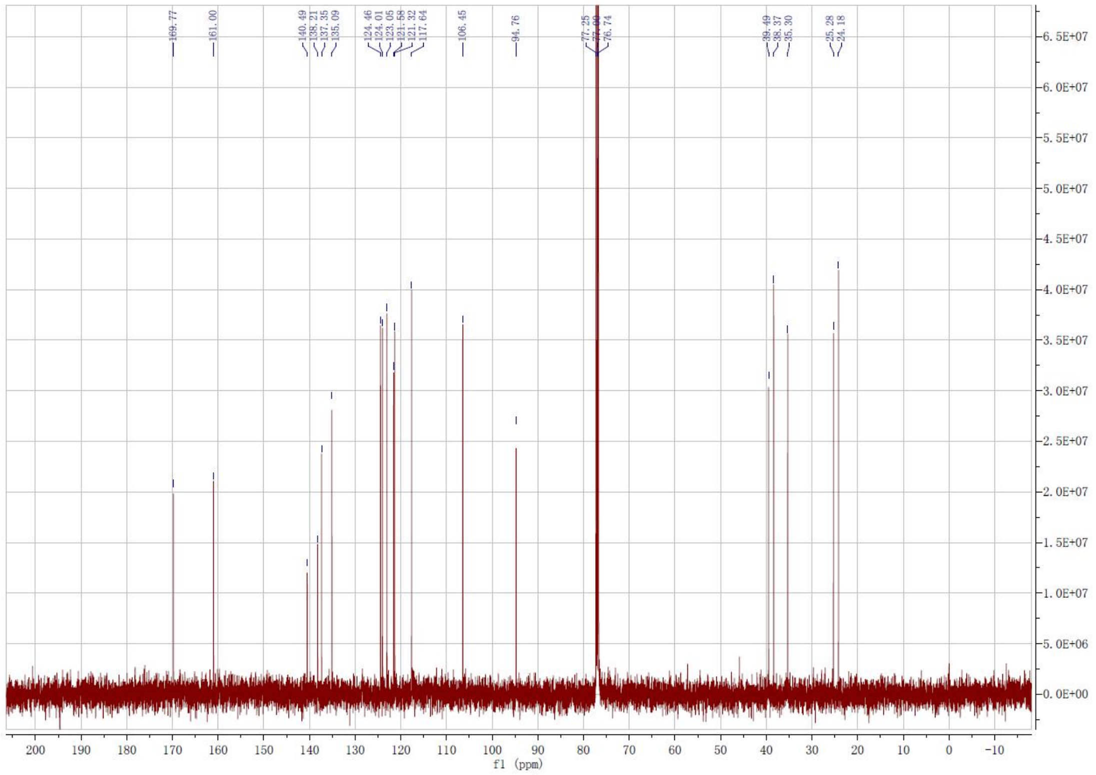

Supplementary Figure 189. ^{13}C NMR spectrum for compound **5t**

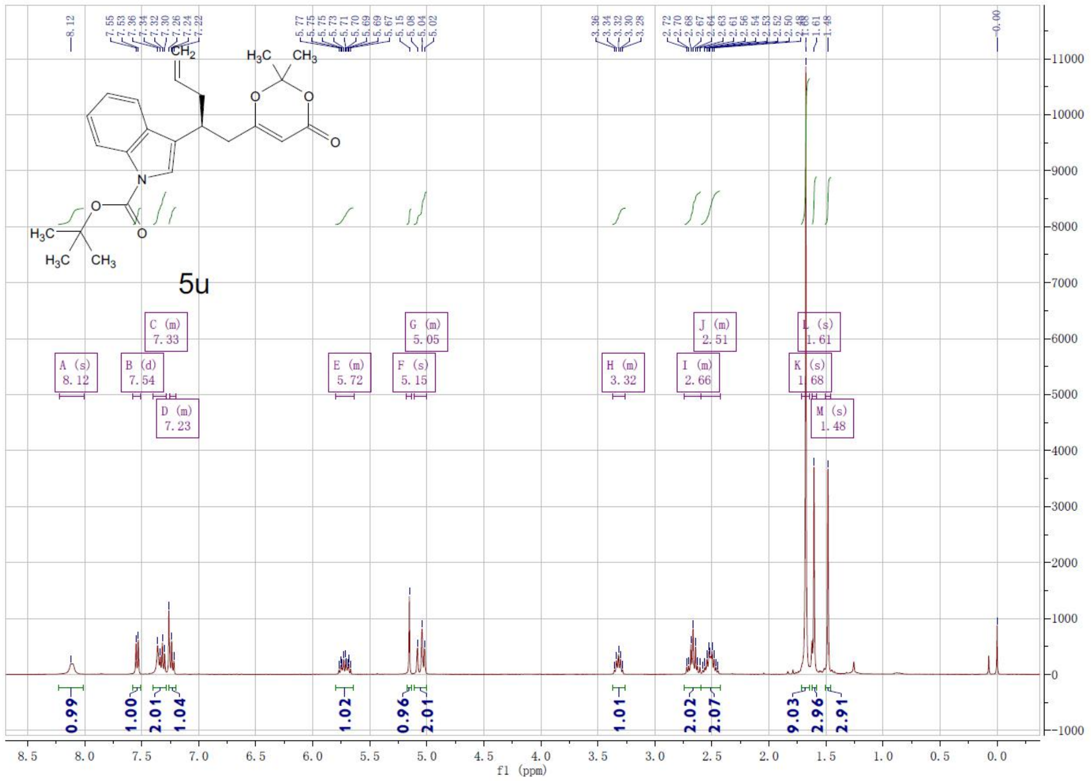

Supplementary Figure 190. ^{1}H NMR spectrum for compound **5u**

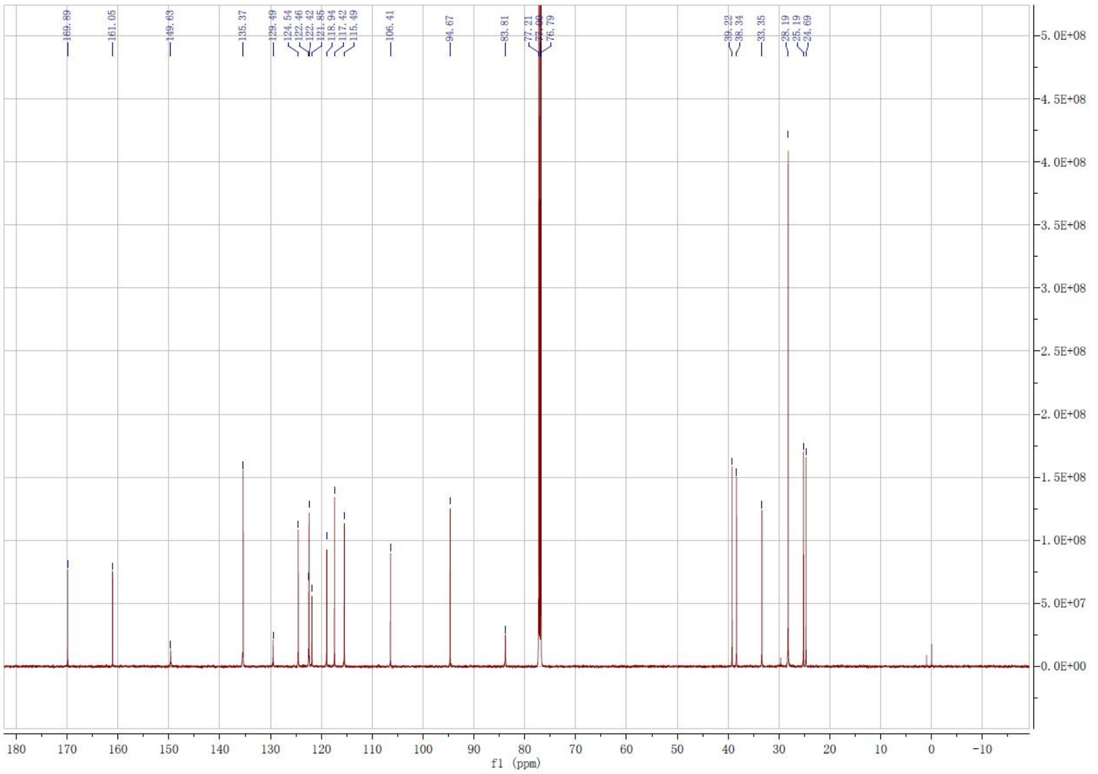

Supplementary Figure 191. ^{13}C NMR spectrum for compound **5u**

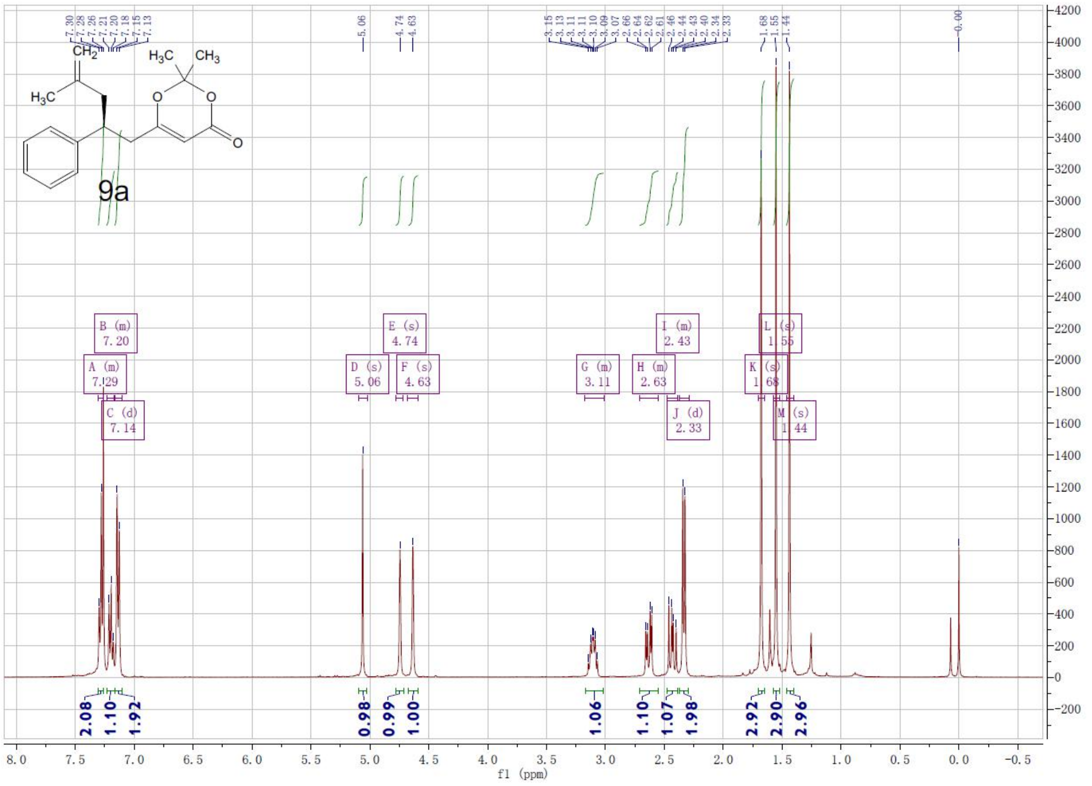

Supplementary Figure 192. ^{1}H NMR spectrum for compound **9a**

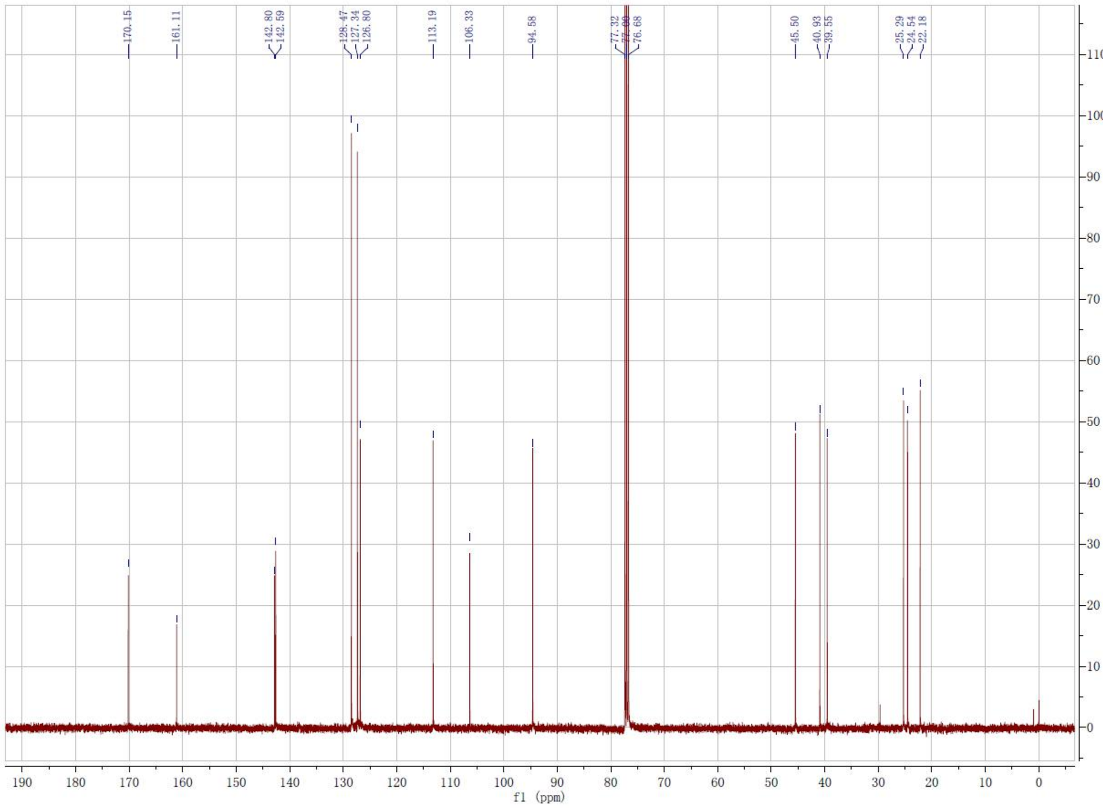

Supplementary Figure 193. ^{13}C NMR spectrum for compound **9a**

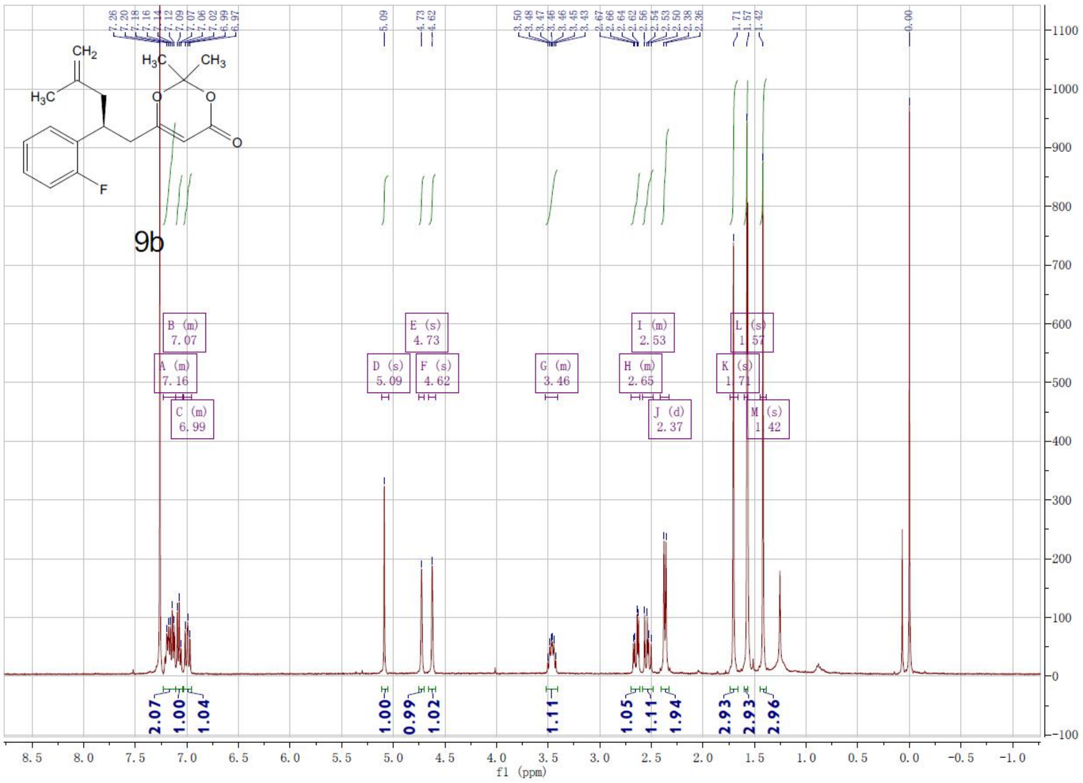

Supplementary Figure 194. ^{1}H NMR spectrum for compound **9b**

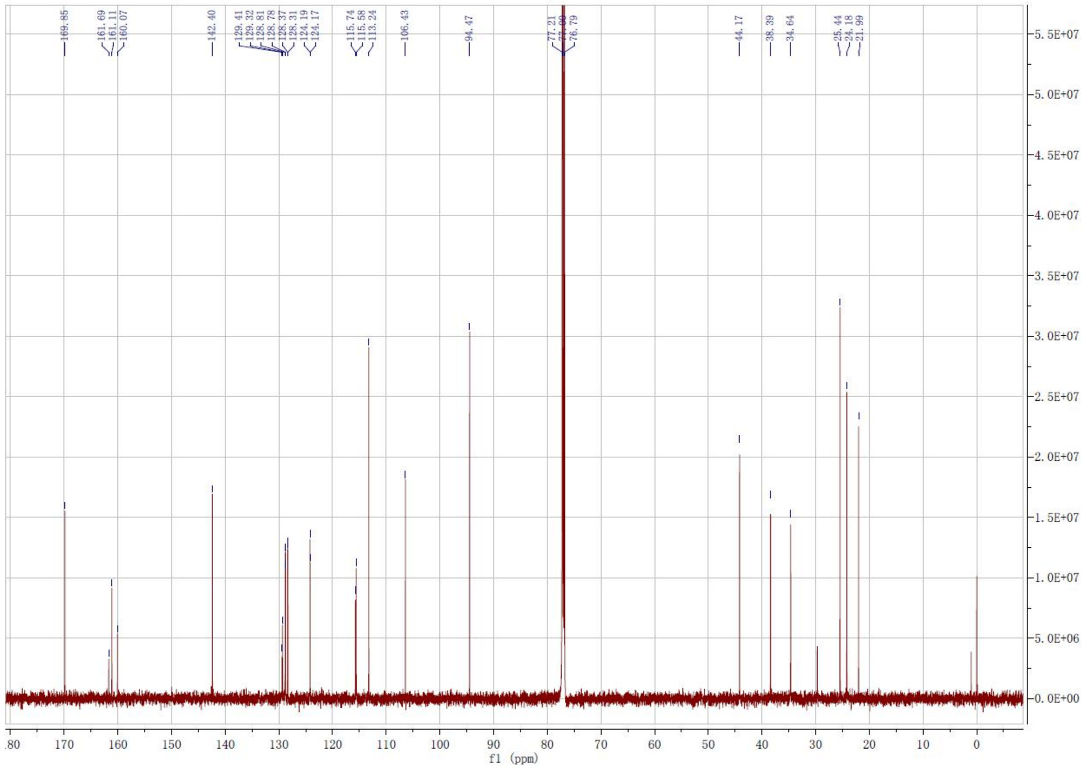

Supplementary Figure 195. ^{13}C NMR spectrum for compound **9b**

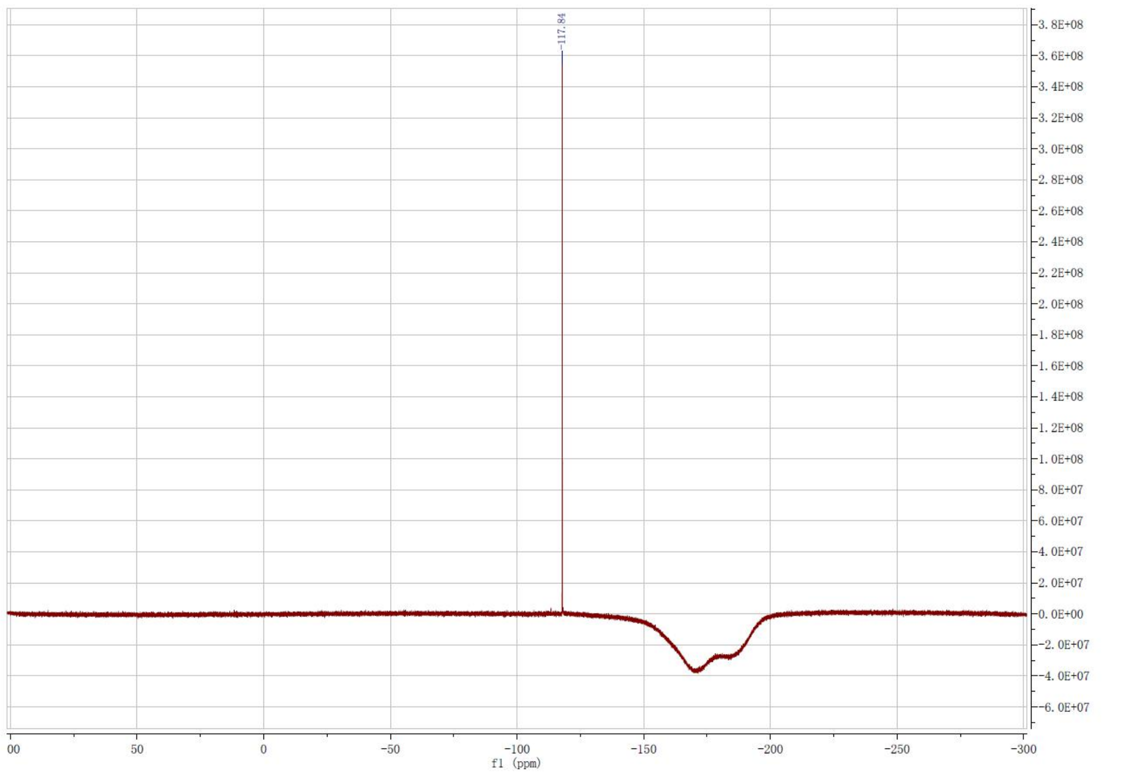

Supplementary Figure 196. ^{19}F NMR spectrum for compound **9b**

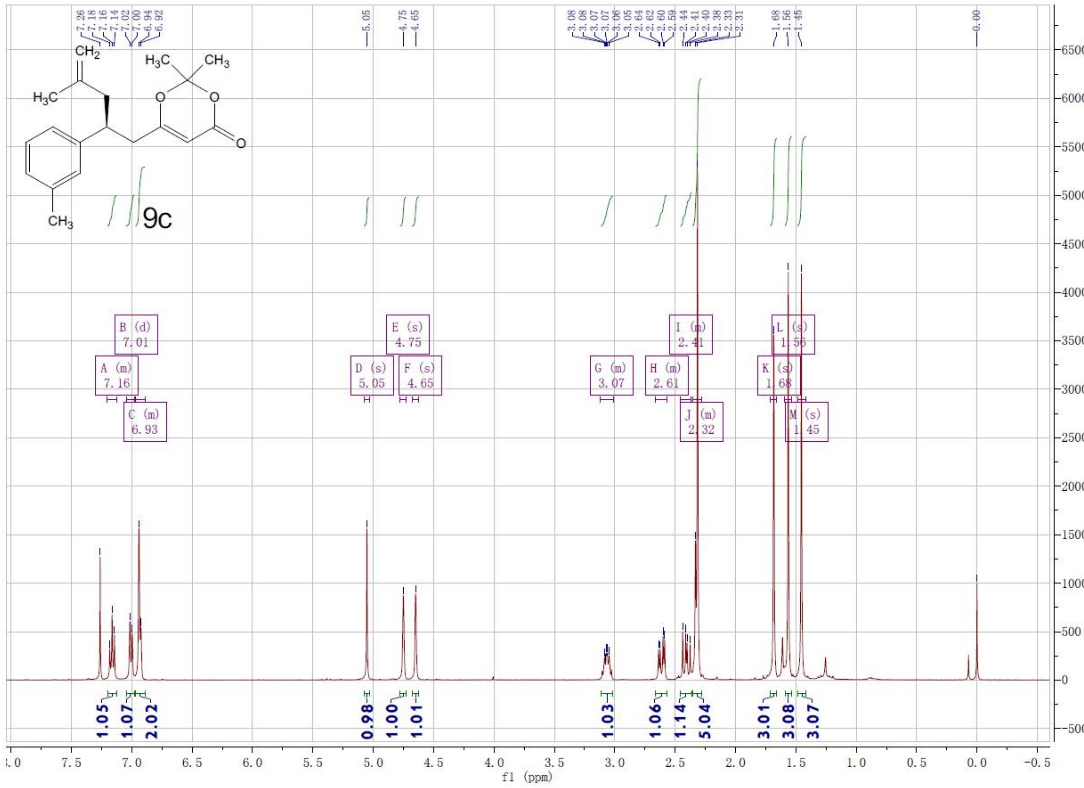

Supplementary Figure 197. ^{1}H NMR spectrum for compound **9c**

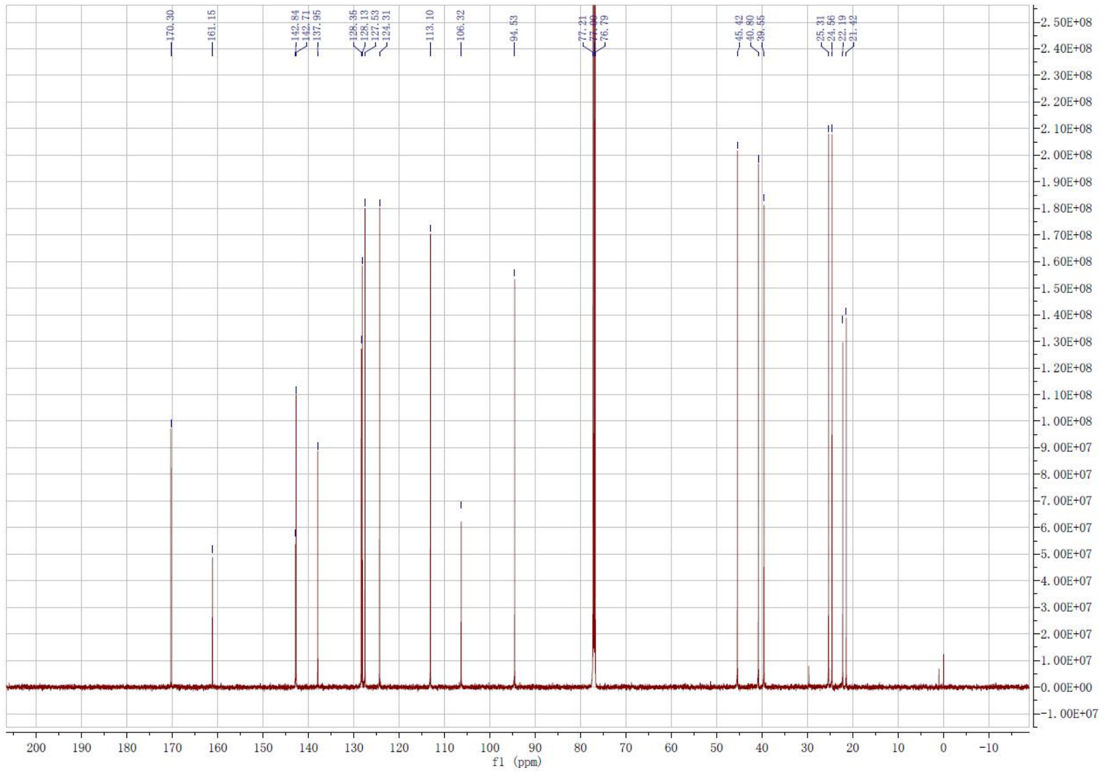

Supplementary Figure 198. ^{13}C NMR spectrum for compound **9c**

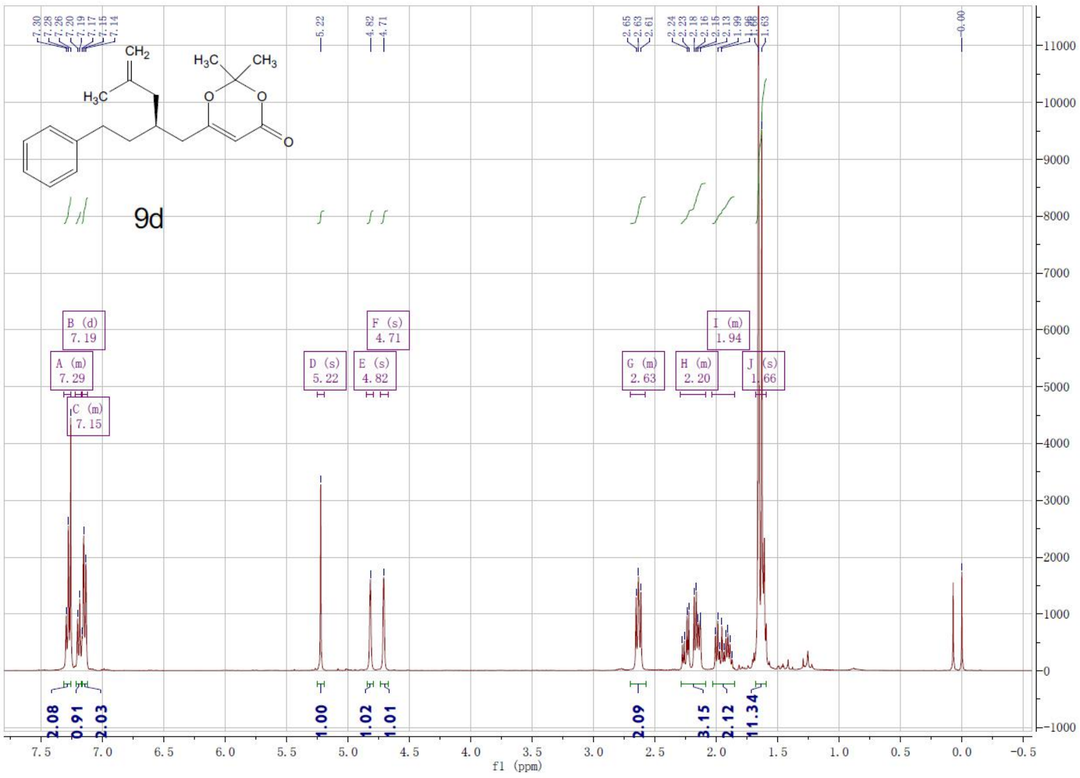

Supplementary Figure 199. ^{1}H NMR spectrum for compound **9d**

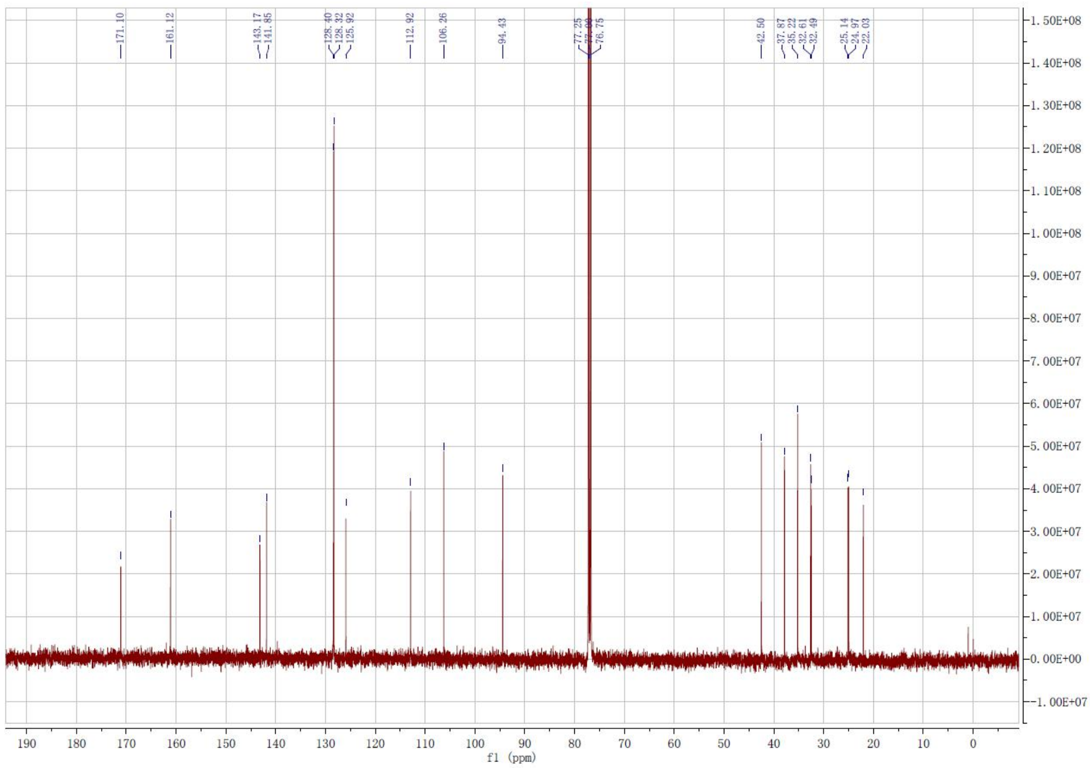

Supplementary Figure 200. ^{13}C NMR spectrum for compound **9d**

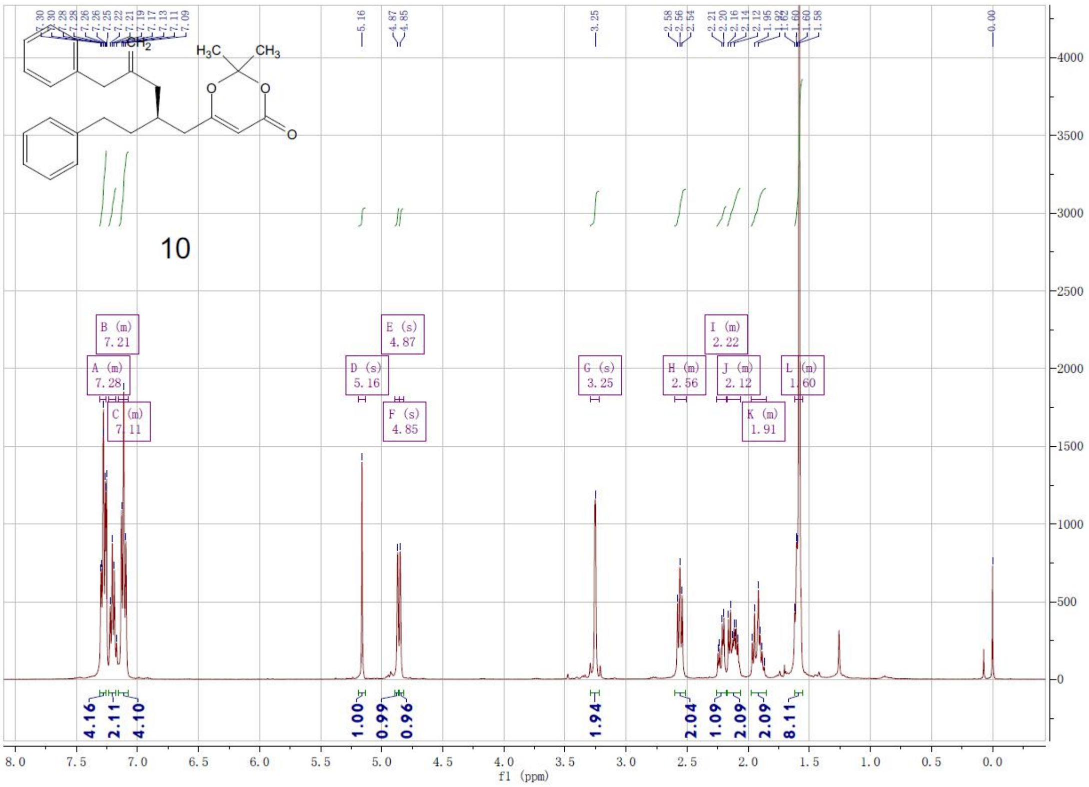

Supplementary Figure 201. ^{1}H NMR spectrum for compound **10**

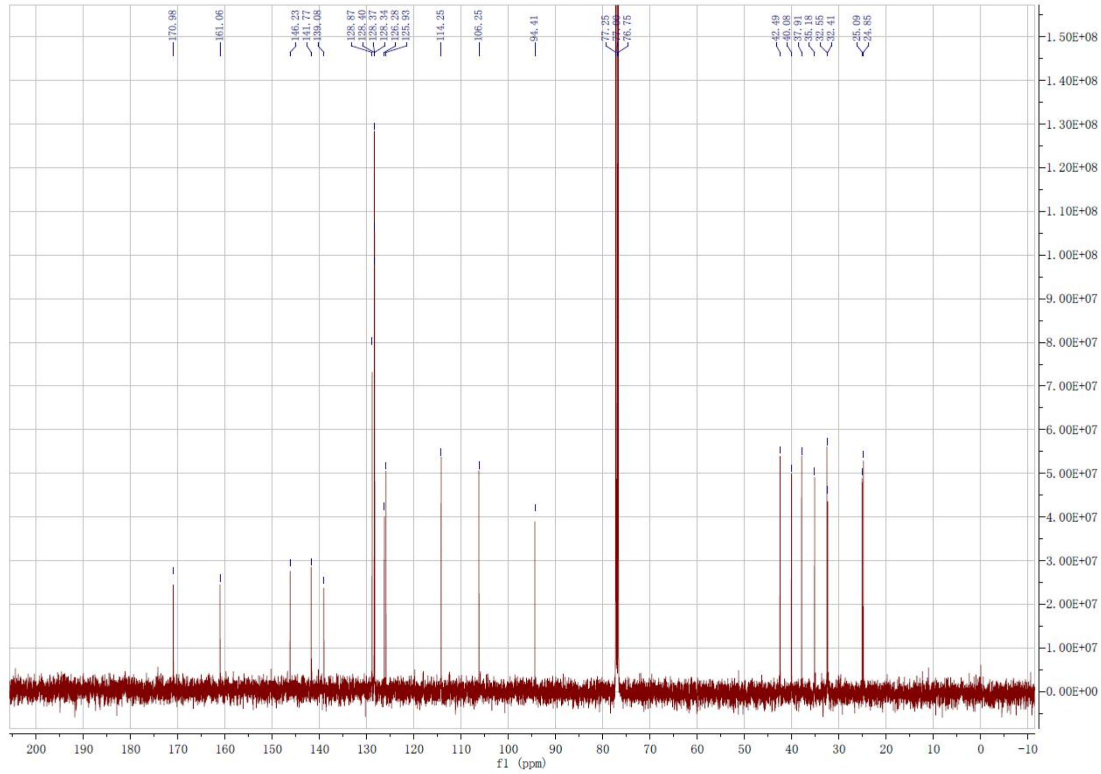

Supplementary Figure 202. ^{13}C NMR spectrum for compound **10**

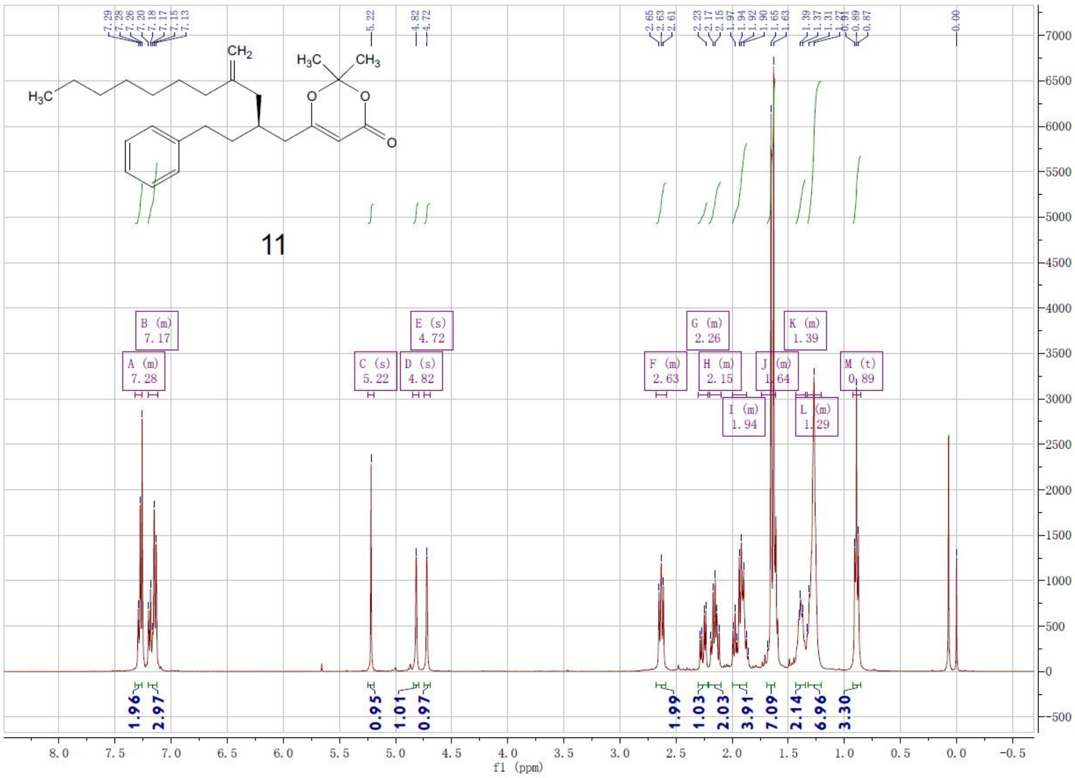

Supplementary Figure 203. ^{1}H NMR spectrum for compound **11**

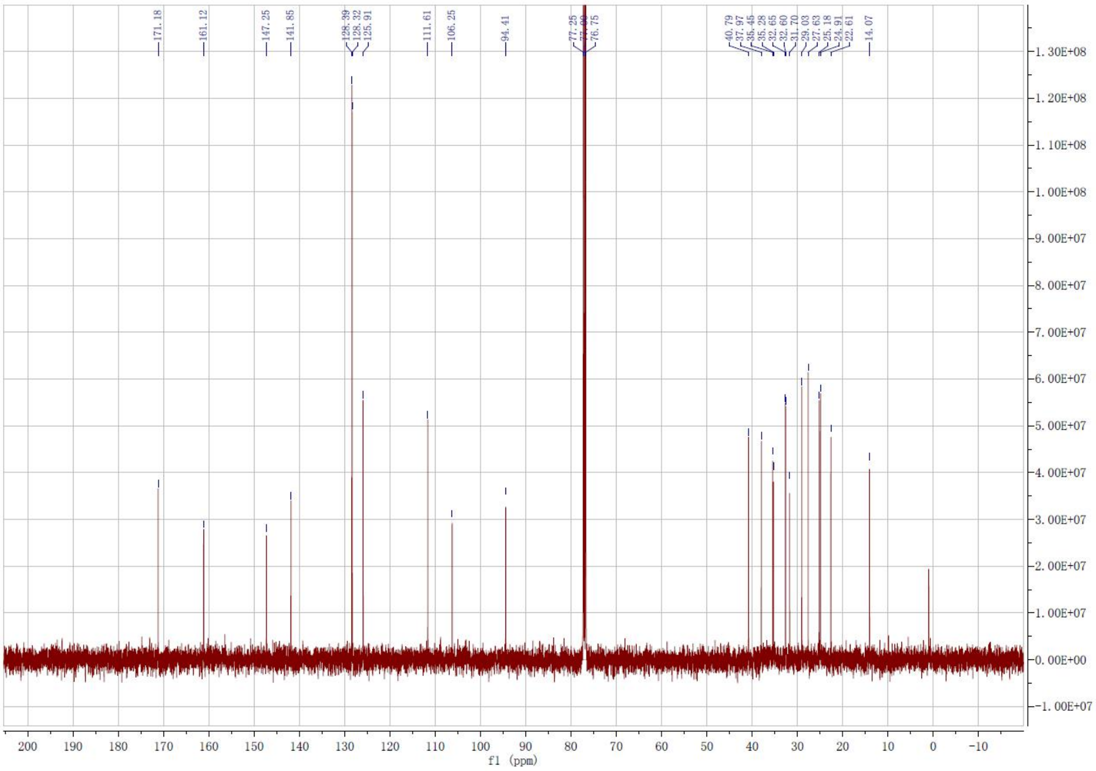

Supplementary Figure 204. ^{13}C NMR spectrum for compound **11**

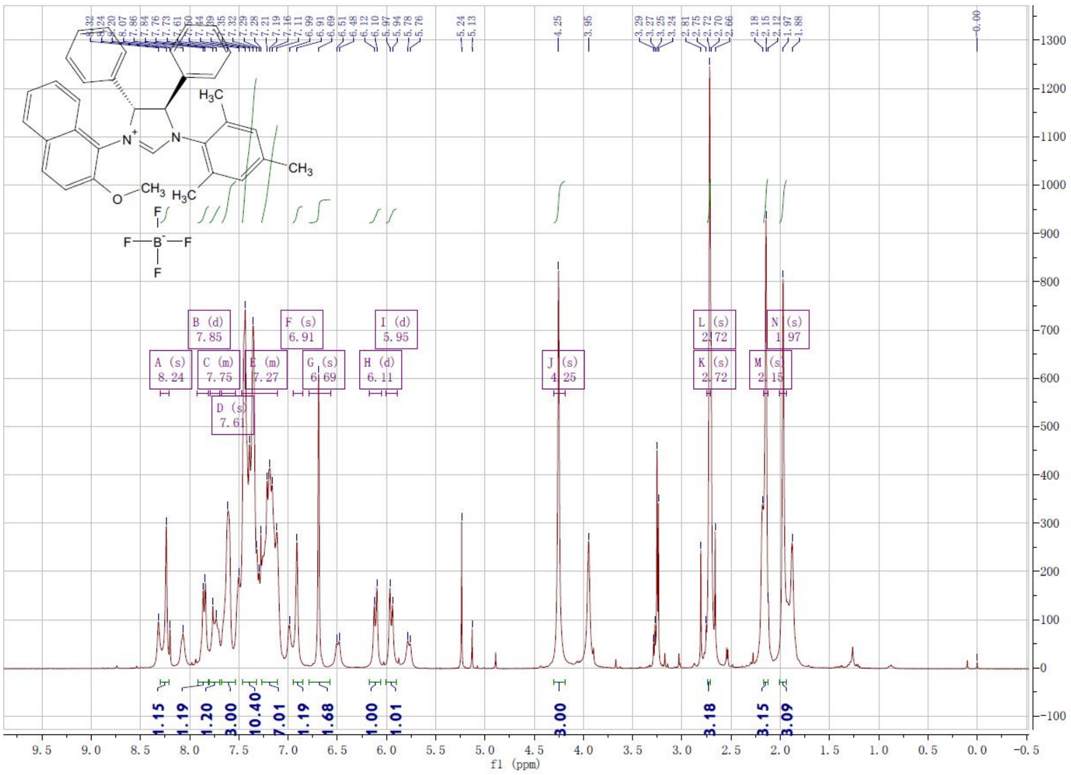

Supplementary Figure 205. ^{1}H NMR spectrum for compound **NHC-L6•HBF4**

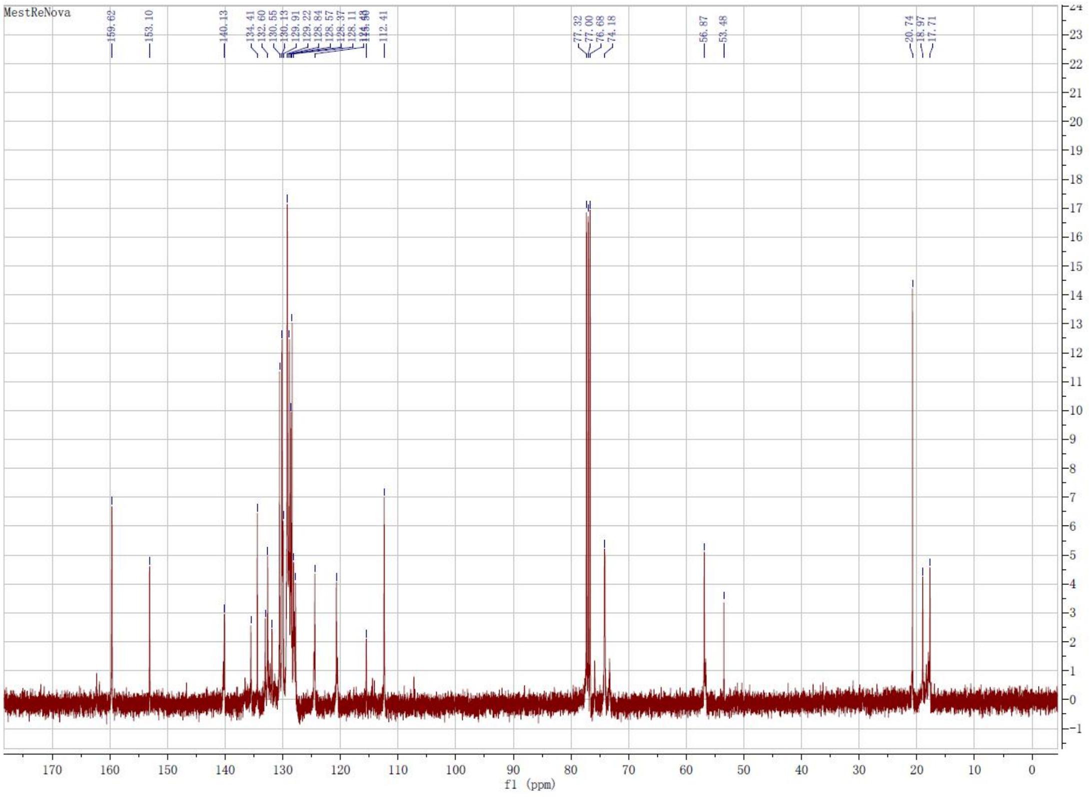

Supplementary Figure 206. ^{13}C NMR spectrum for compound **NHC-L6•HBF4**

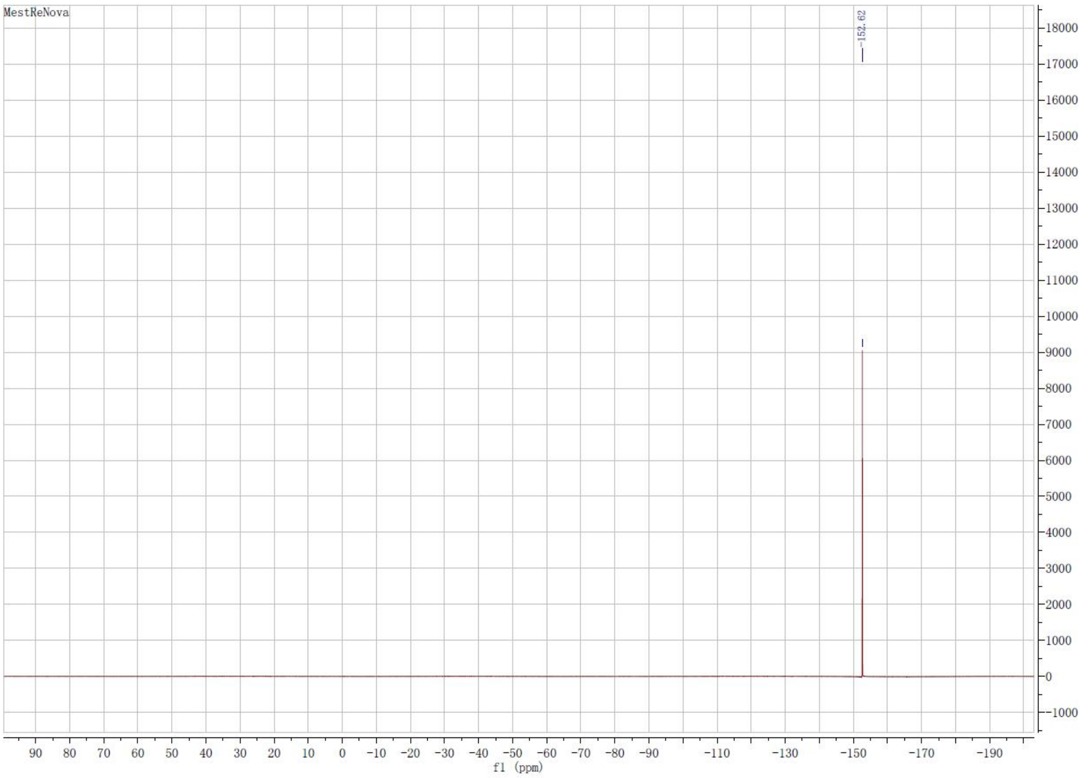

Supplementary Figure 207. ^{19}F NMR spectrum for compound **NHC-L6•HBF4**

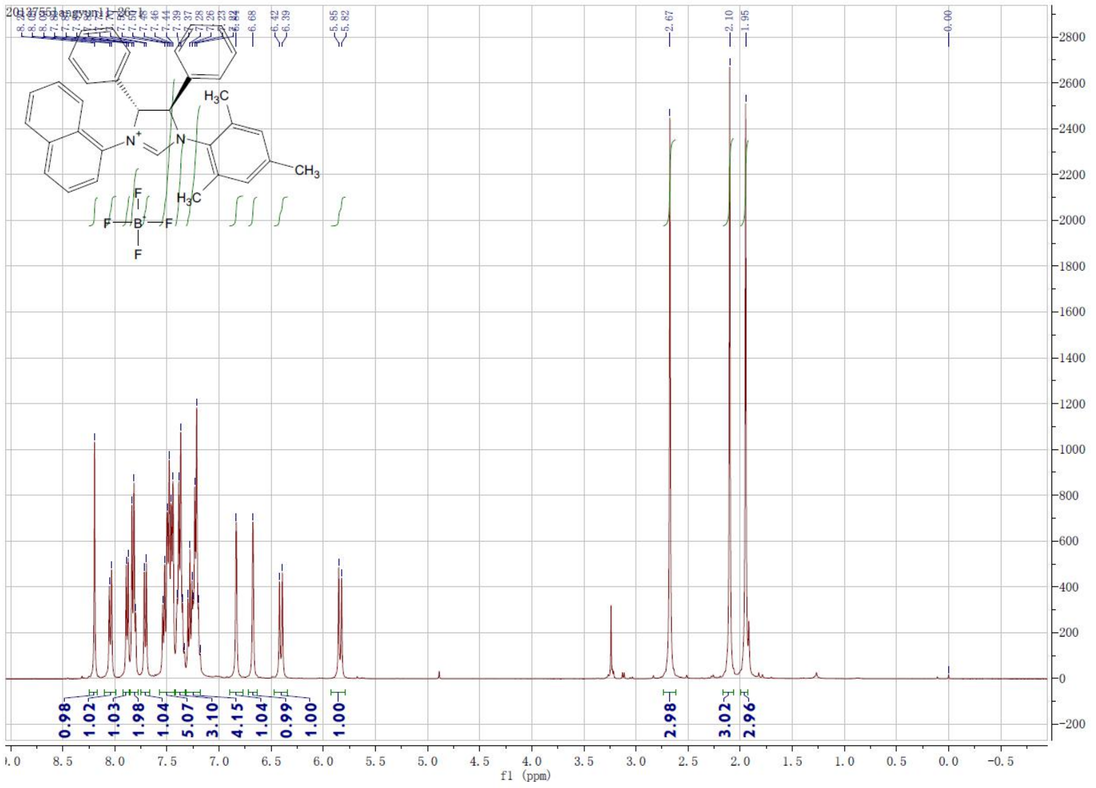

Supplementary Figure 208. ^{1}H NMR spectrum for compound **NHC-L7•HBF4**

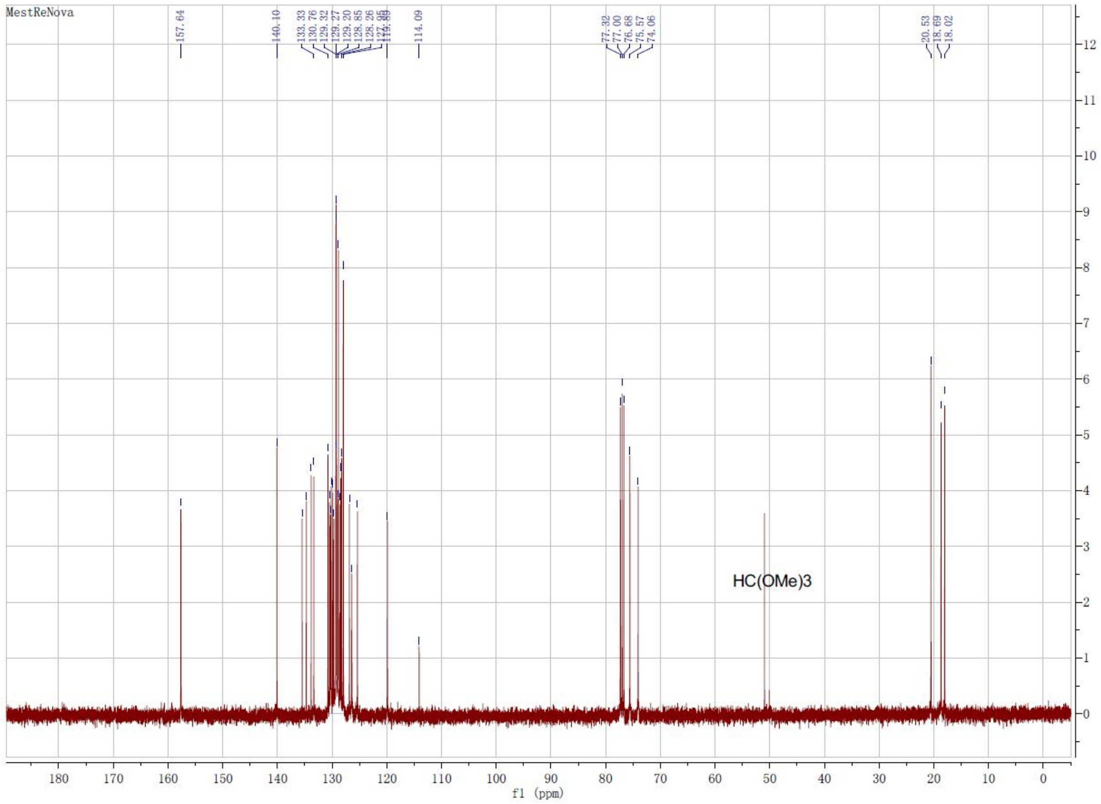

Supplementary Figure 209. ^{13}C NMR spectrum for compound **NHC-L7•HBF4**

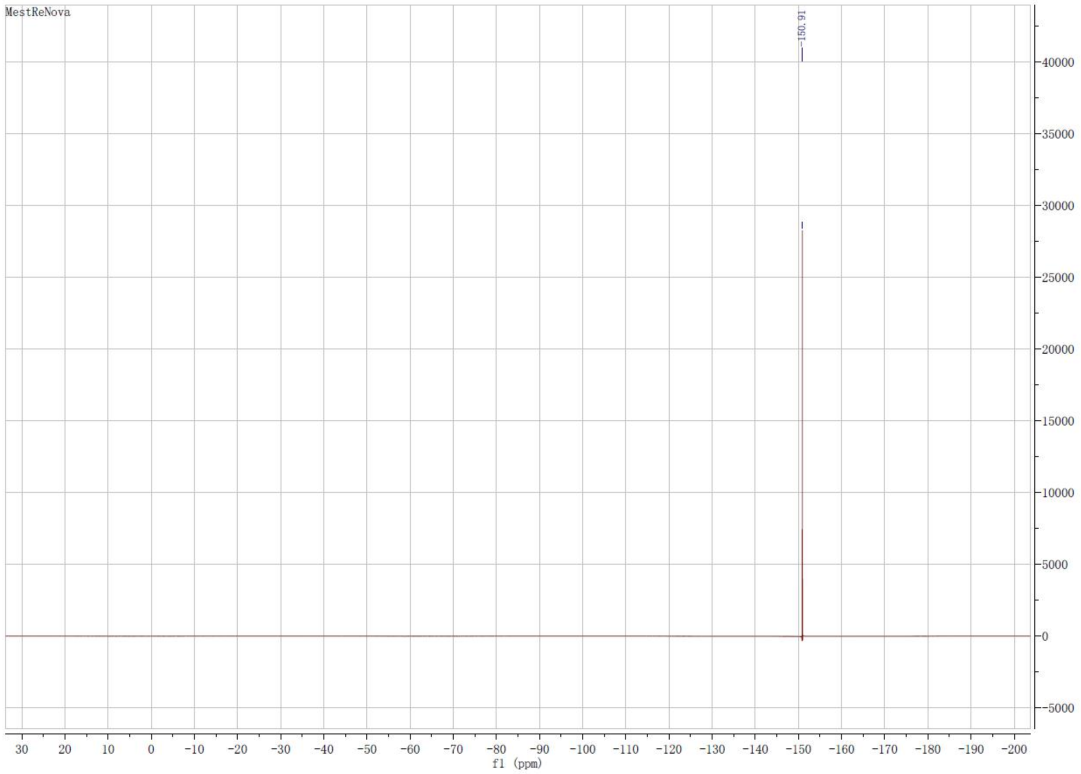

Supplementary Figure 210. ^{19}F NMR spectrum for compound **NHC-L7•HBF4**

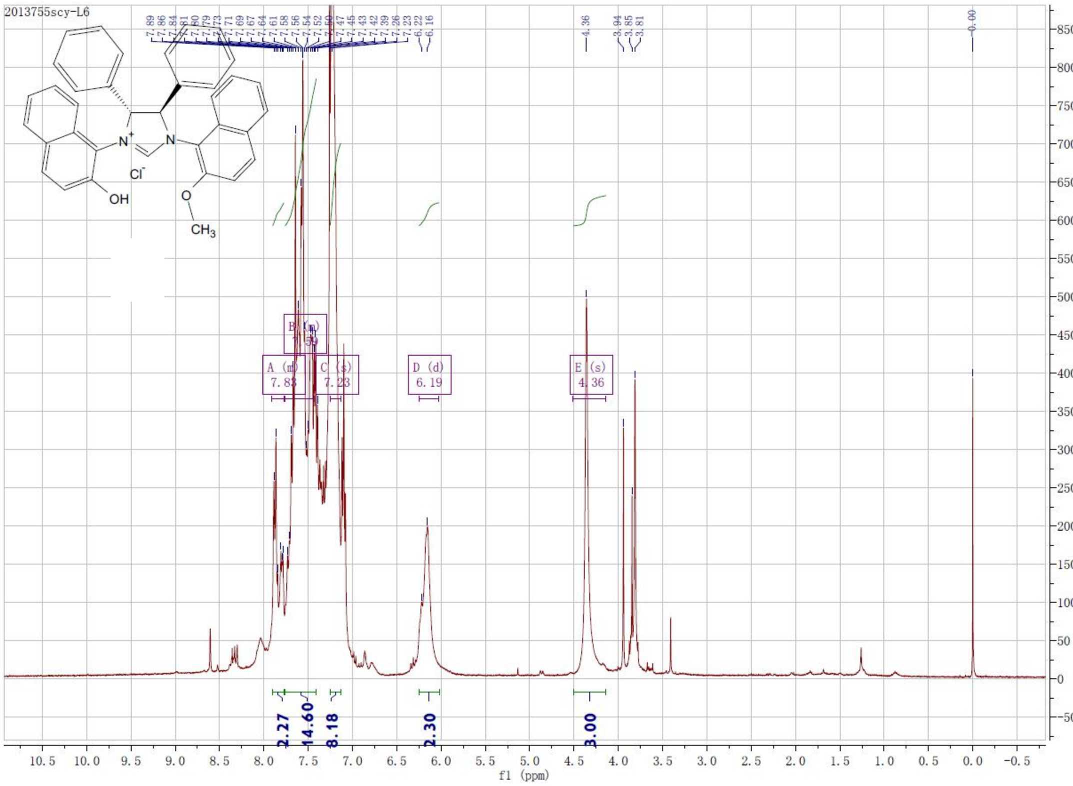

Supplementary Figure 211. ^{1}H NMR spectrum for compound **NHC-L8•HBF4**

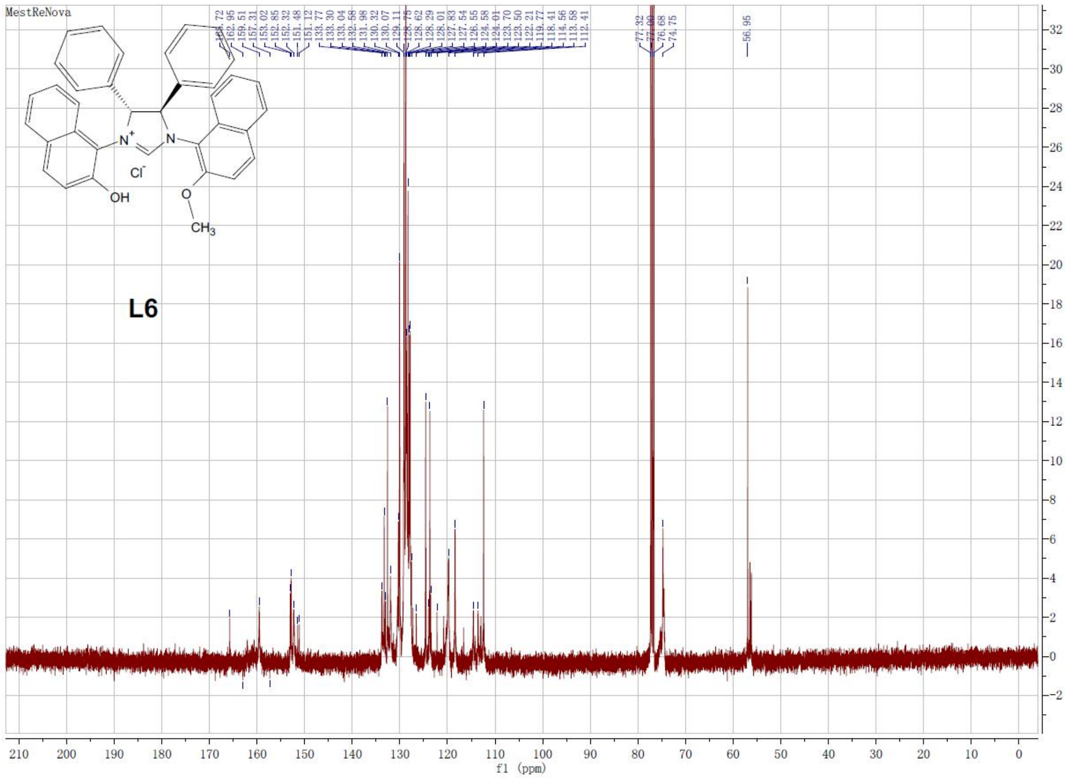

Supplementary Figure 212. ^{13}C NMR spectrum for compound **NHC-L8•HBF4**

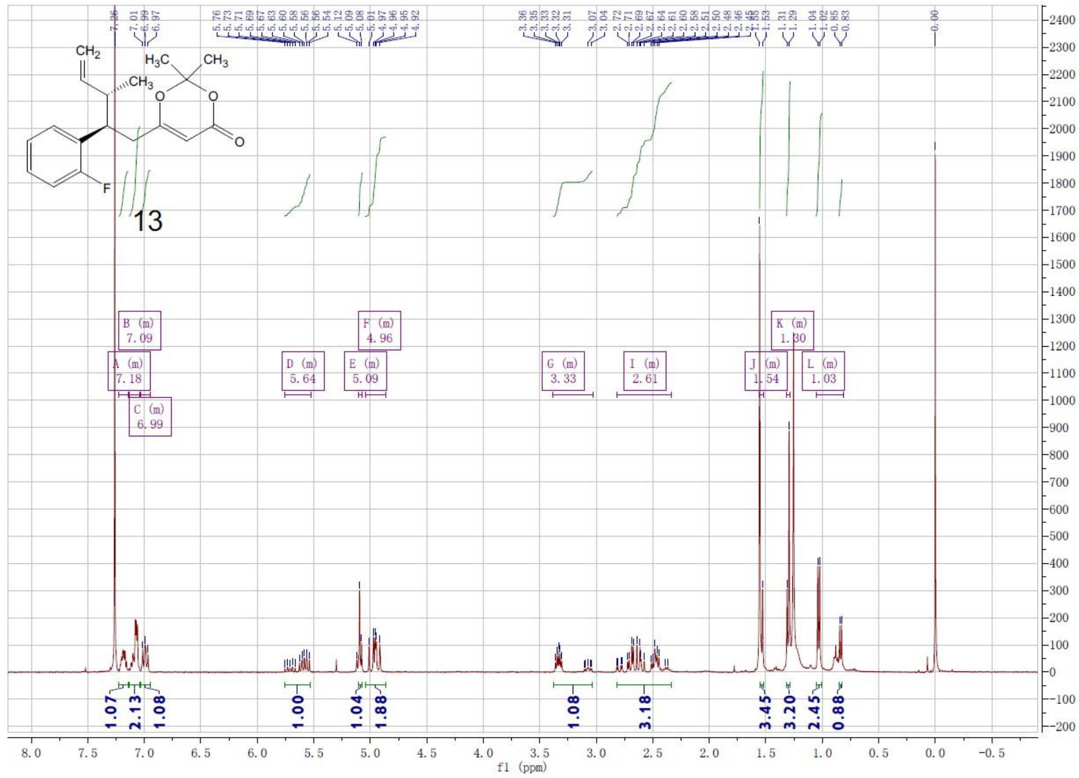

Supplementary Figure 213. ^{1}H NMR spectrum for compound **13**

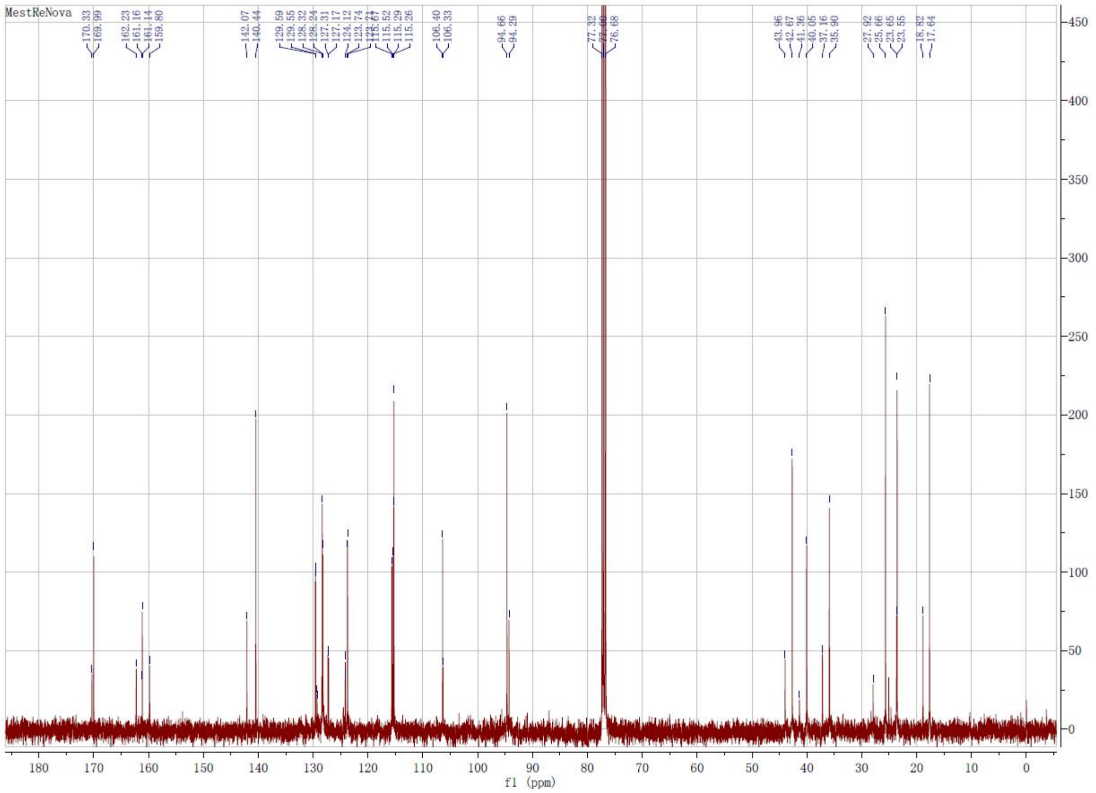

Supplementary Figure 214. ^{13}C NMR spectrum for compound **13**

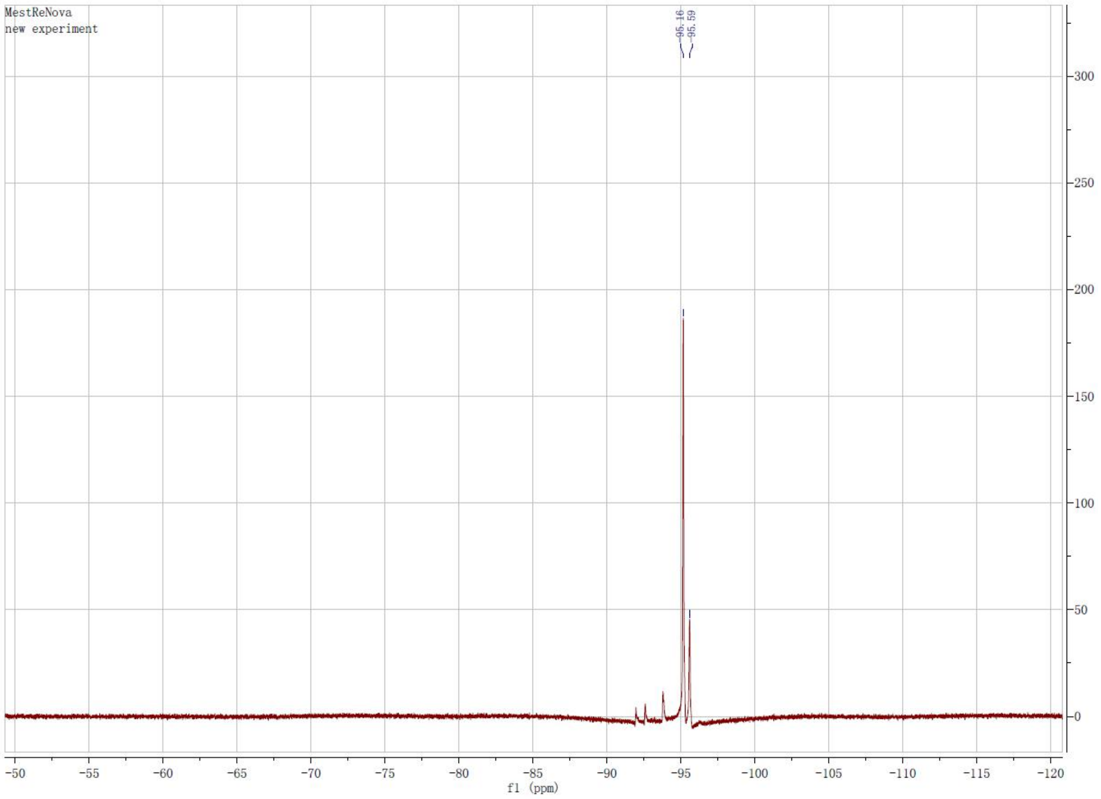

Supplementary Figure 215. ^{19}F NMR spectrum for compound **13**

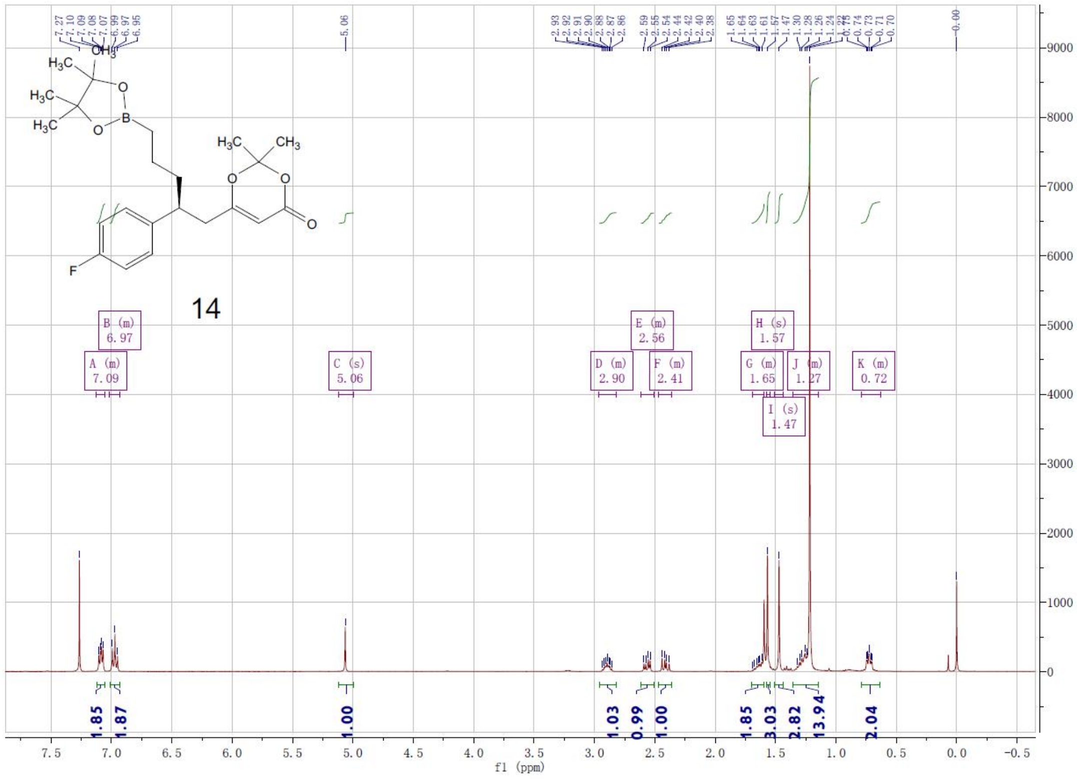

Supplementary Figure 216. ^{1}H NMR spectrum for compound **14**

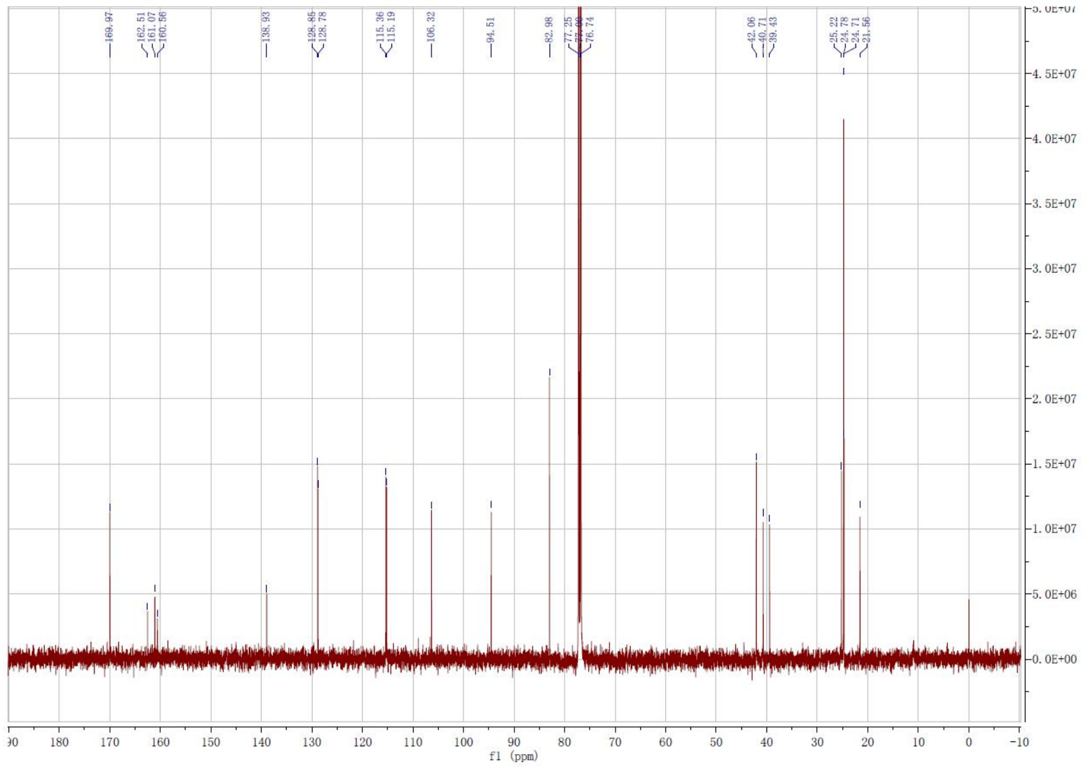

Supplementary Figure 217. ^{13}C NMR spectrum for compound **14**

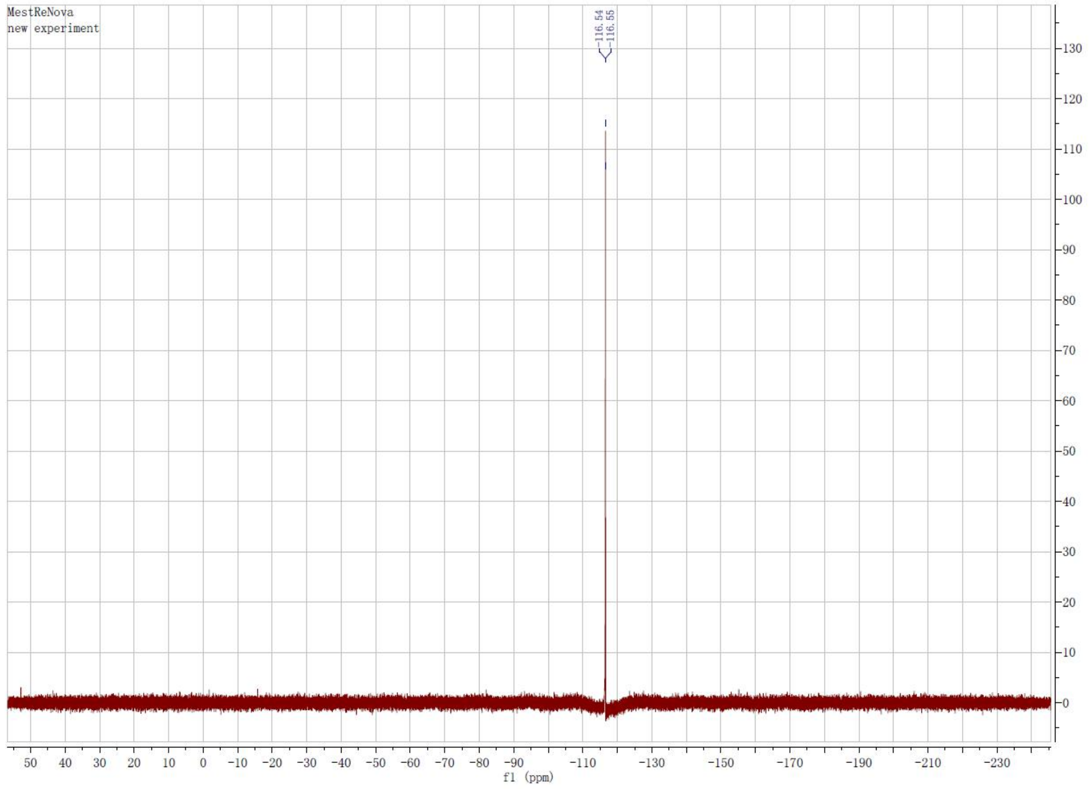

Supplementary Figure 218. ^{19}F NMR spectrum for compound **14**

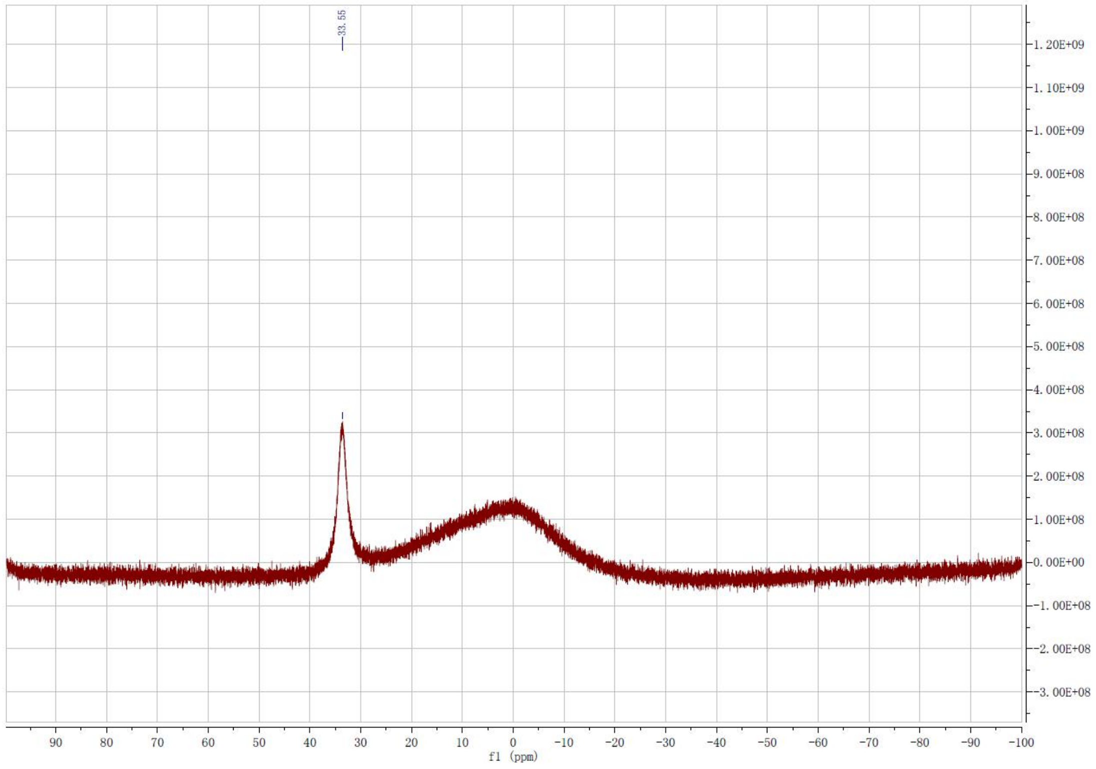

Supplementary Figure 219. ^{11}B NMR spectrum for compound **14**

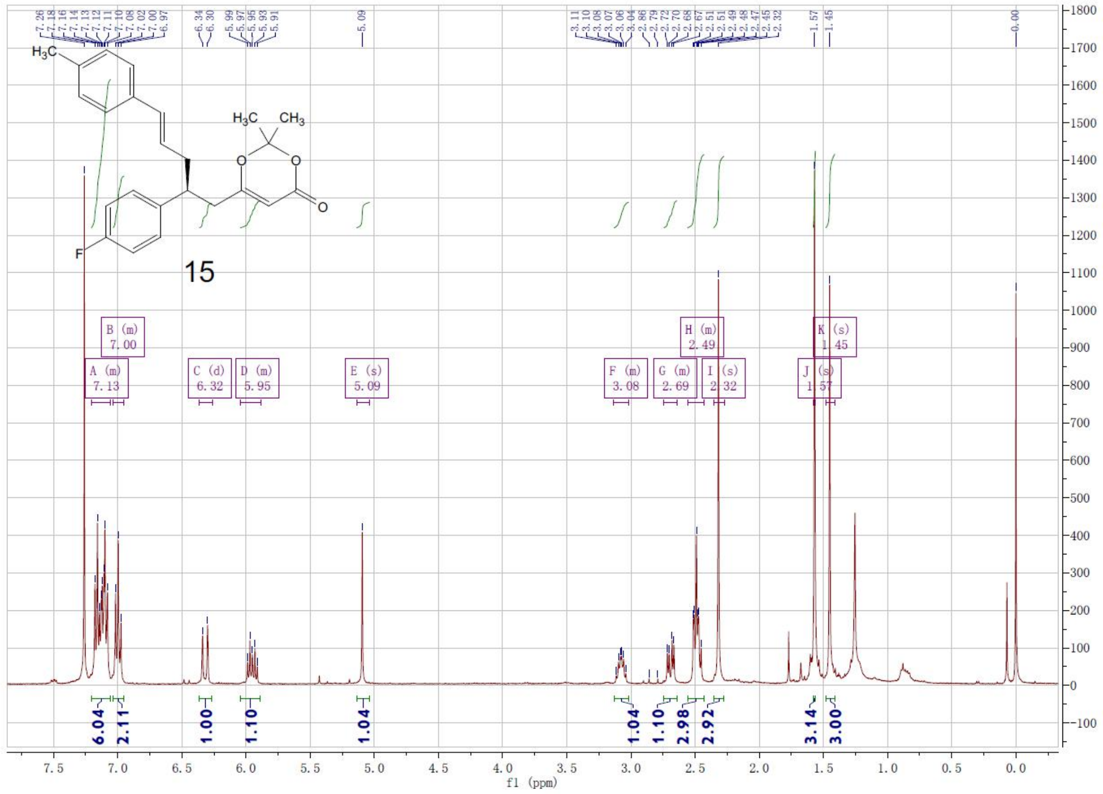

Supplementary Figure 220. ^{1}H NMR spectrum for compound **15**

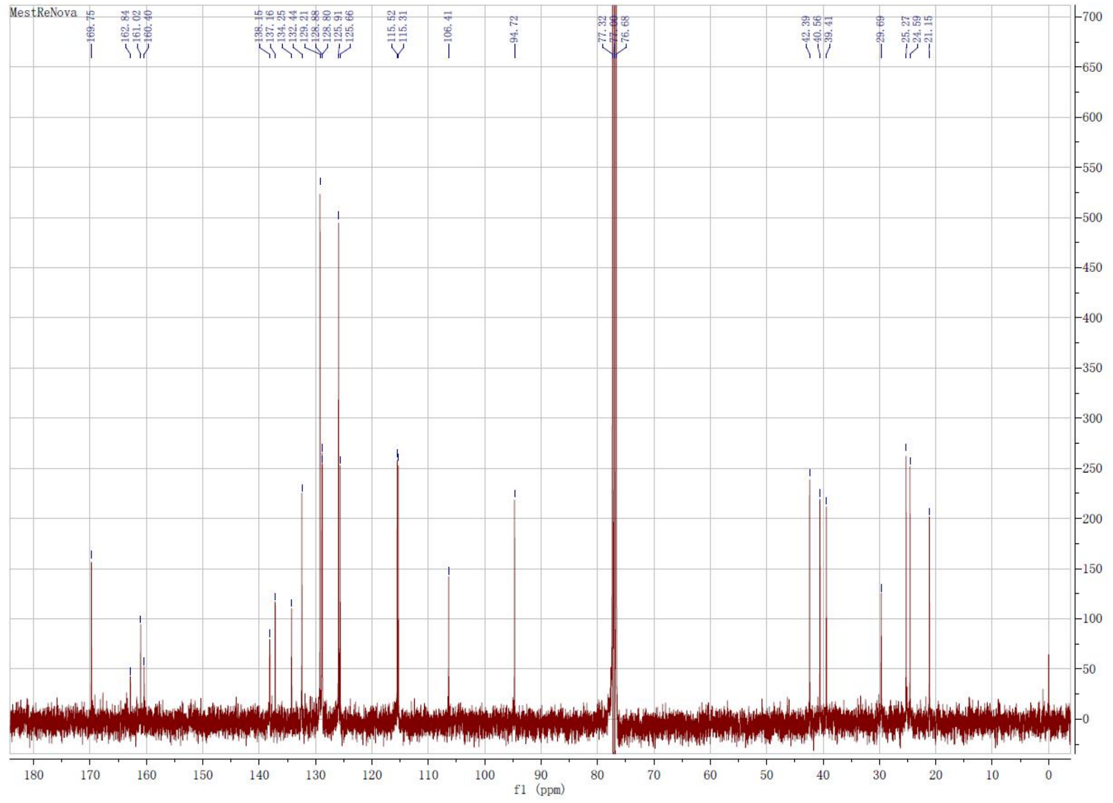

Supplementary Figure 221. ^{13}C NMR spectrum for compound **15**

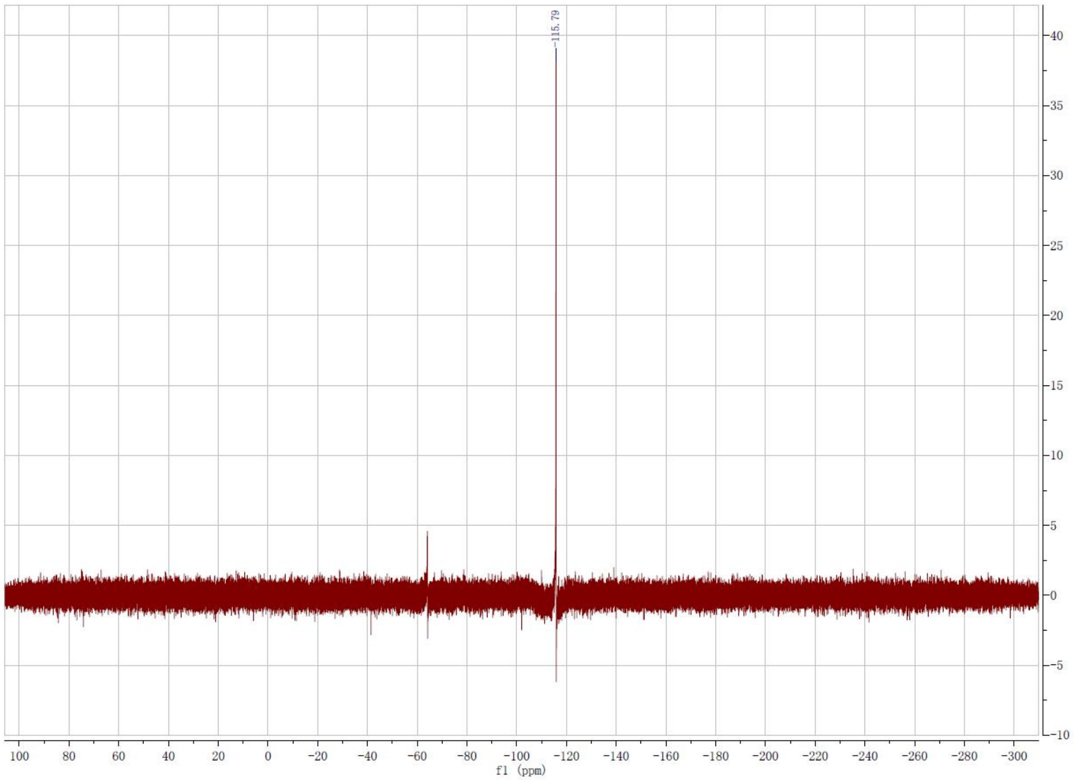

Supplementary Figure 222. ^{19}F NMR spectrum for compound **15**

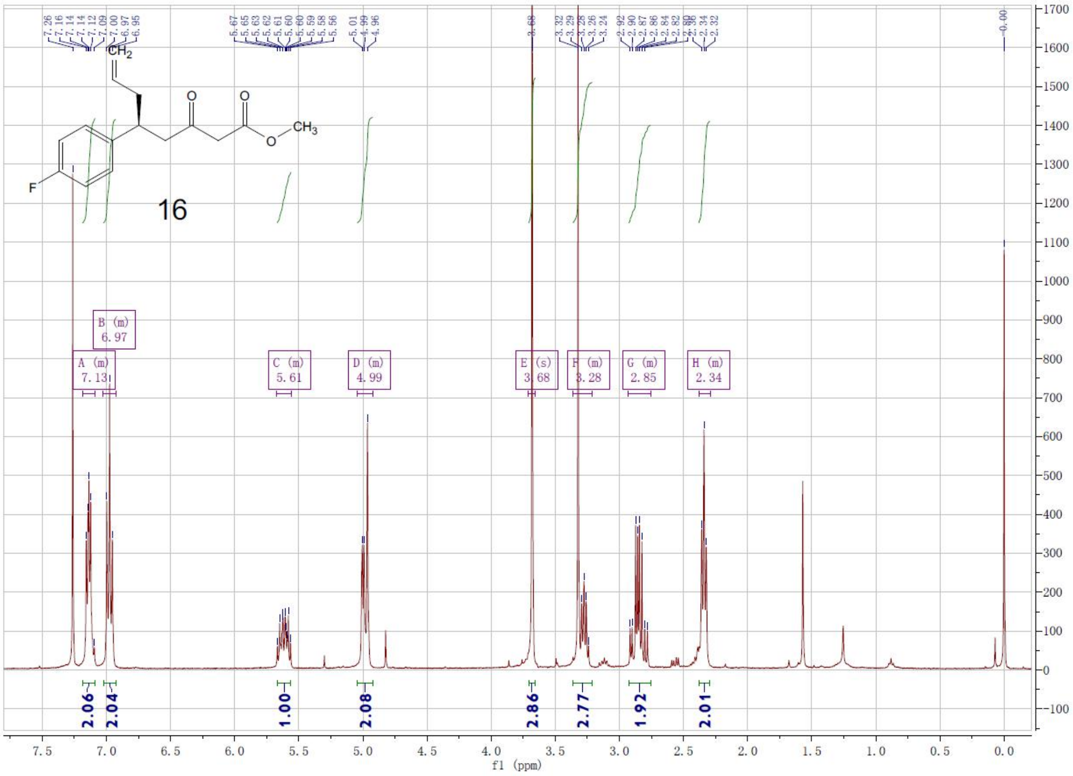

Supplementary Figure 223. ^{1}H NMR spectrum for compound **16**

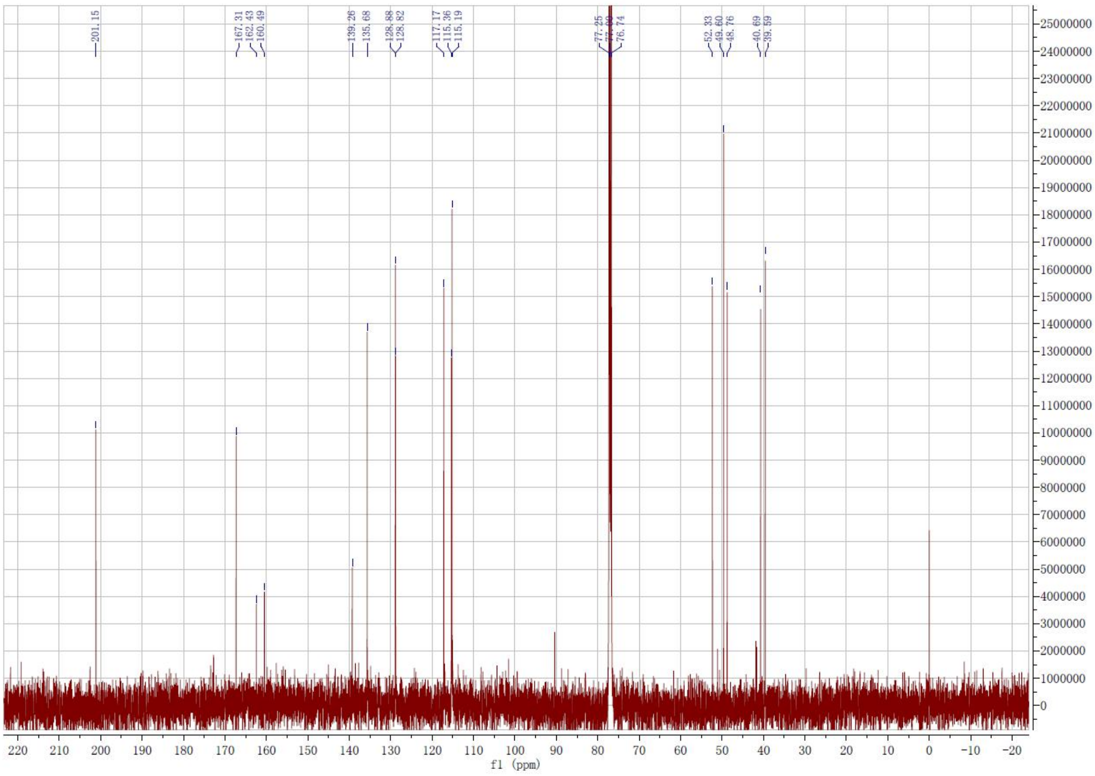

Supplementary Figure 224. ^{13}C NMR spectrum for compound **16**

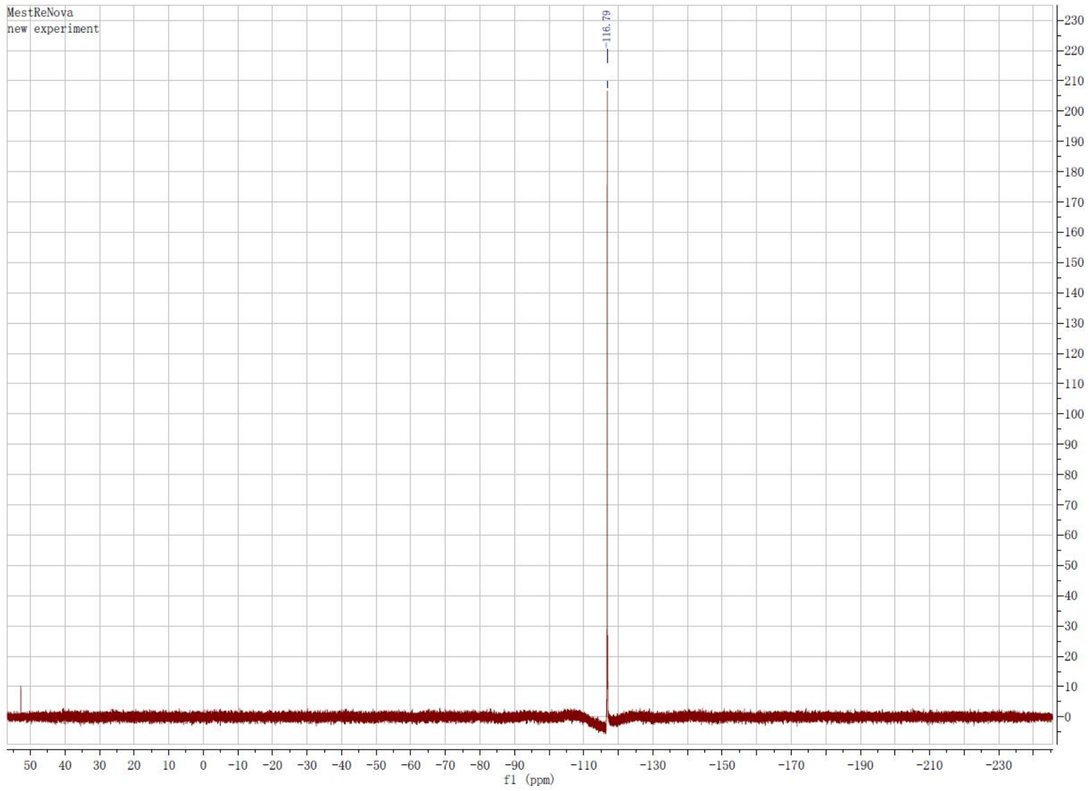

Supplementary Figure 225. ^{19}F NMR spectrum for compound **16**

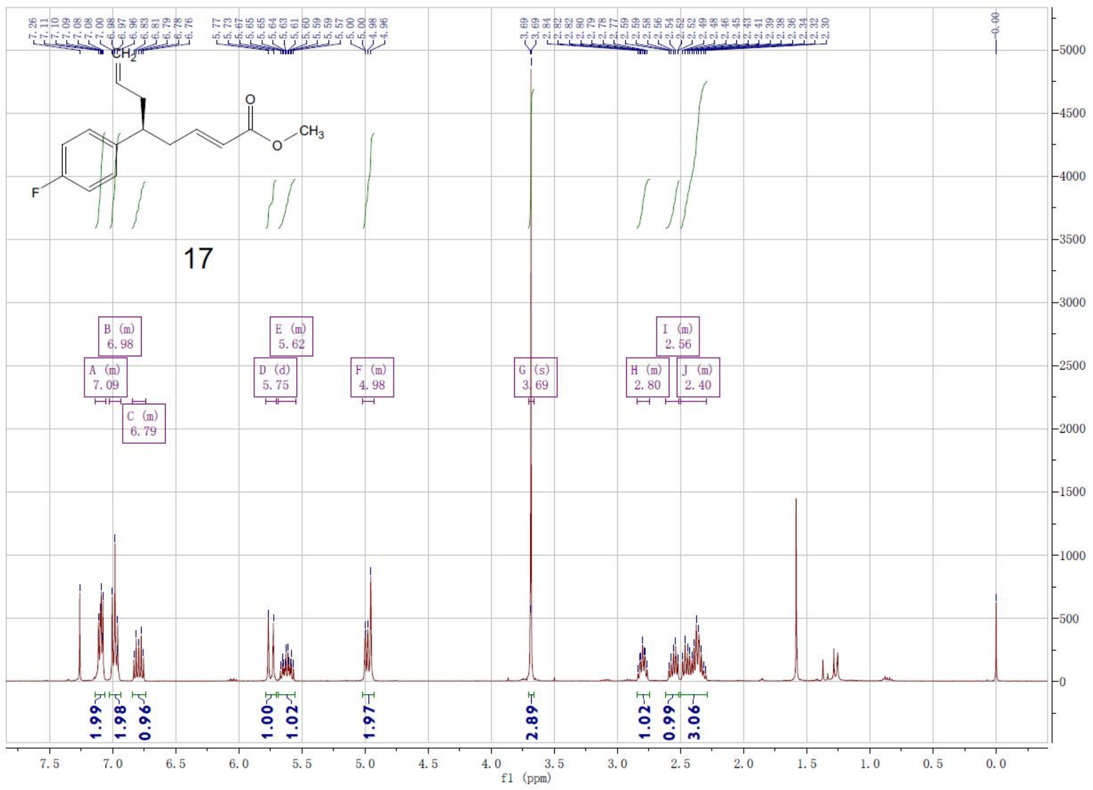

Supplementary Figure 226. ^{1}H NMR spectrum for compound **17**

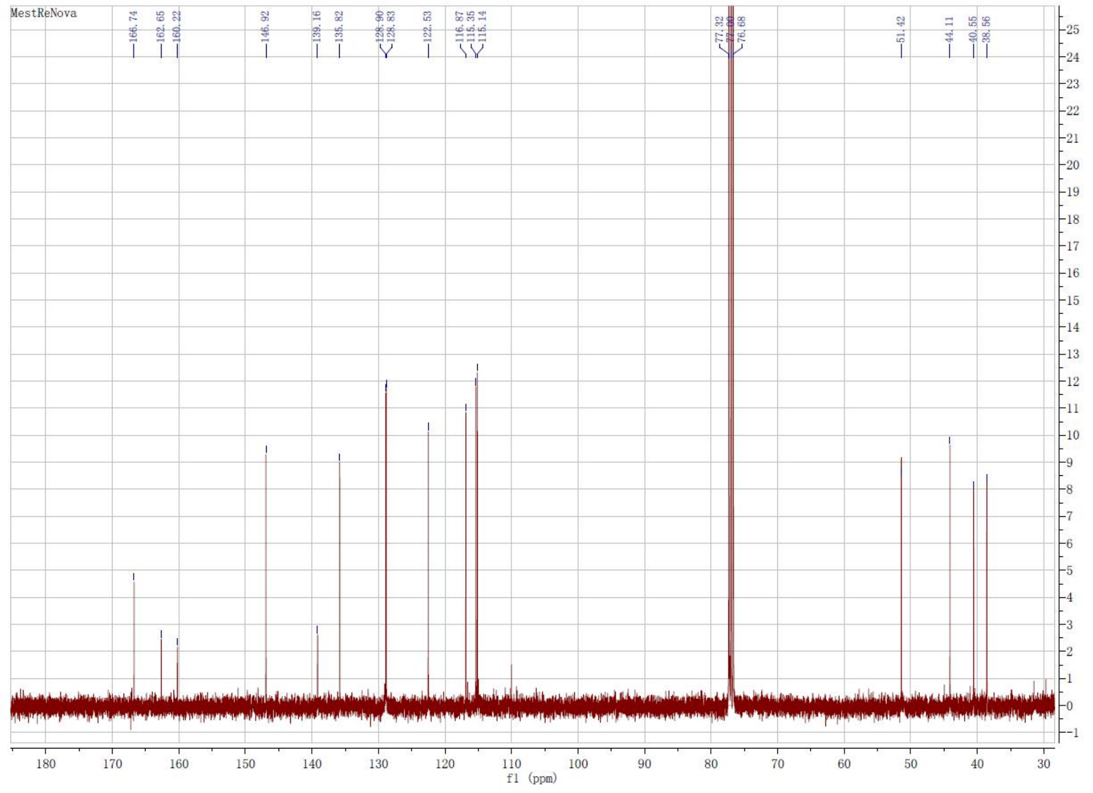

Supplementary Figure 227. ^{13}C NMR spectrum for compound **17**

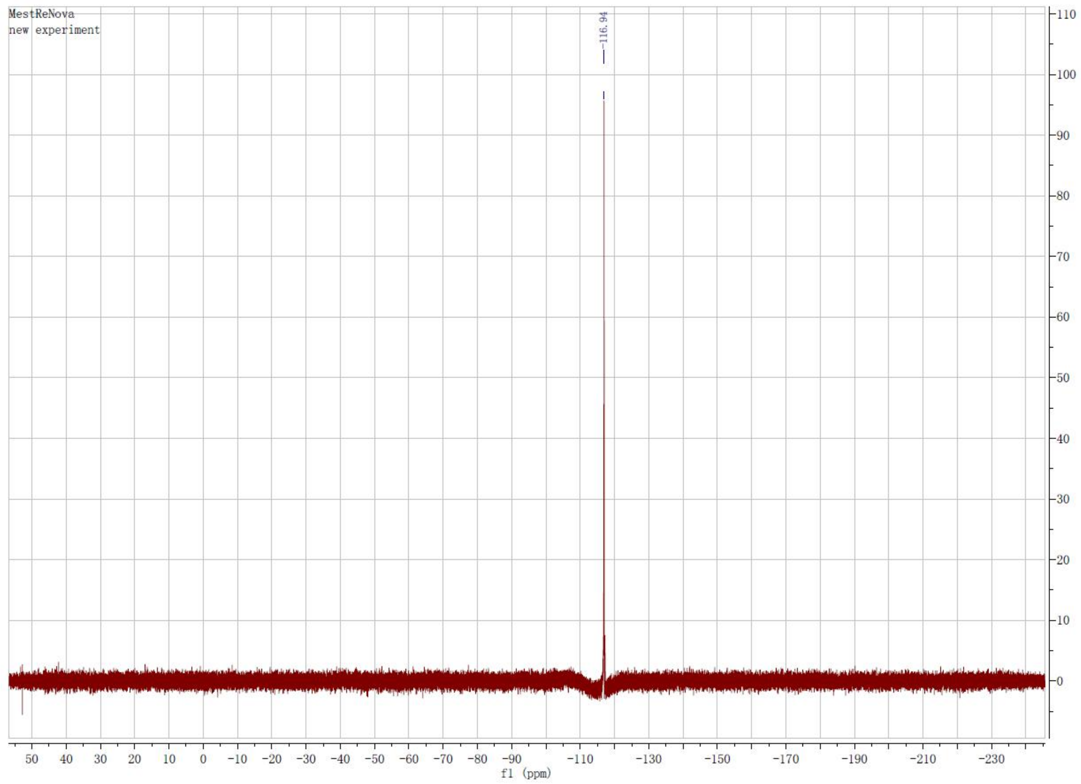

Supplementary Figure 228. ^{19}F NMR spectrum for compound **17**

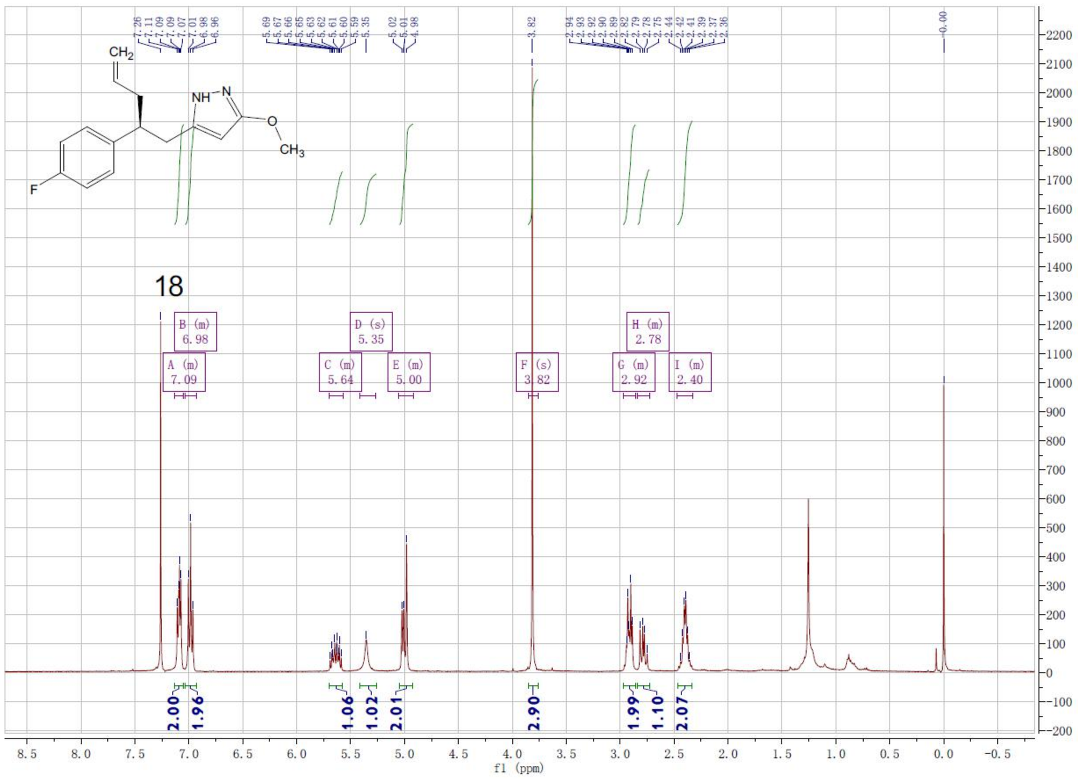

Supplementary Figure 229. ^{1}H NMR spectrum for compound **18**

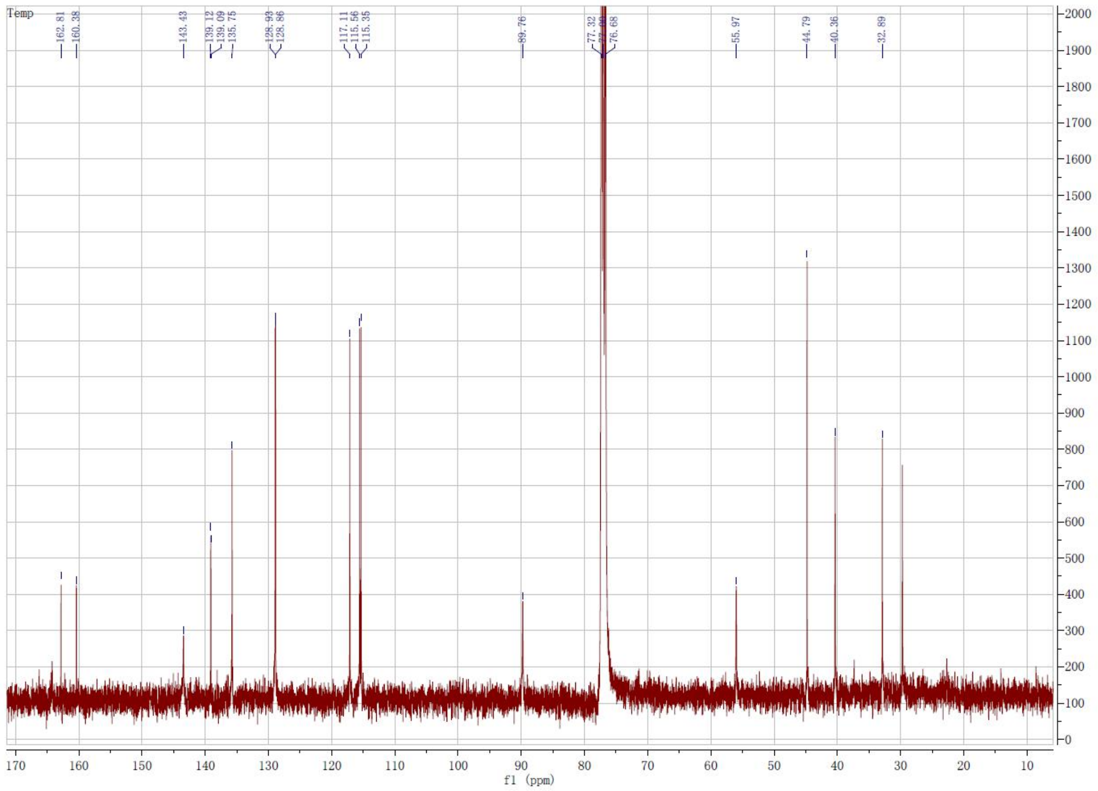

Supplementary Figure 230. ^{13}C NMR spectrum for compound **18**

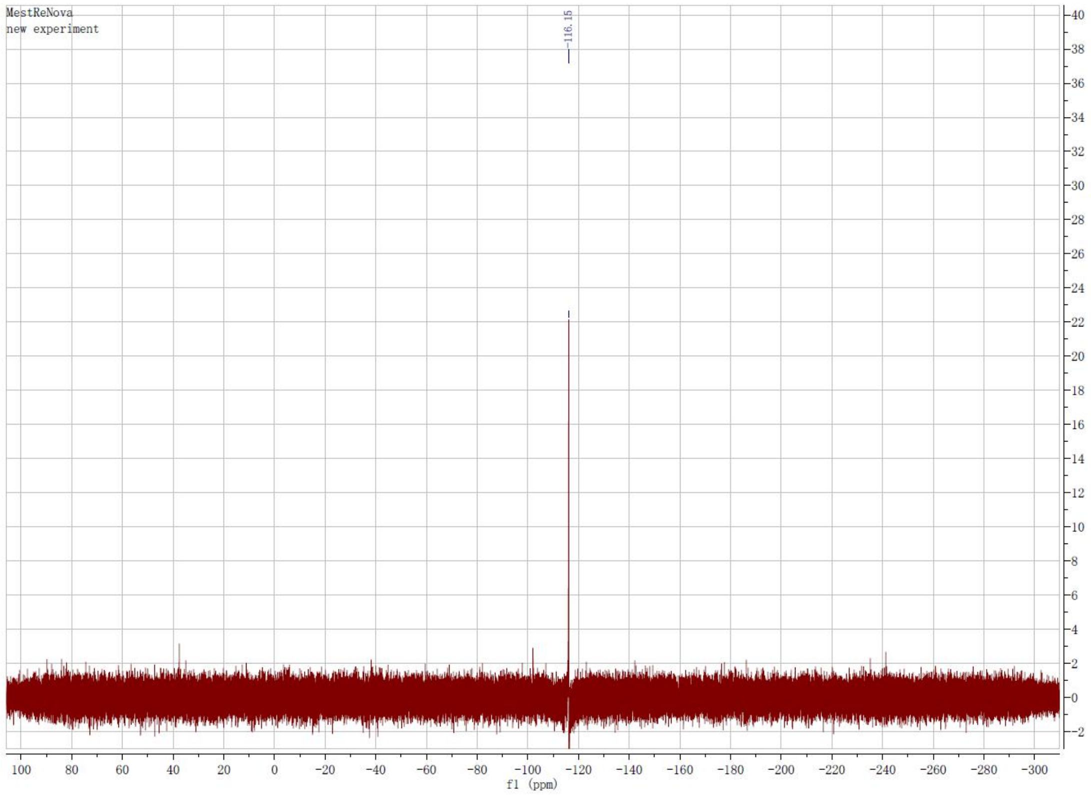

Supplementary Figure 231. ^{19}F NMR spectrum for compound **18**

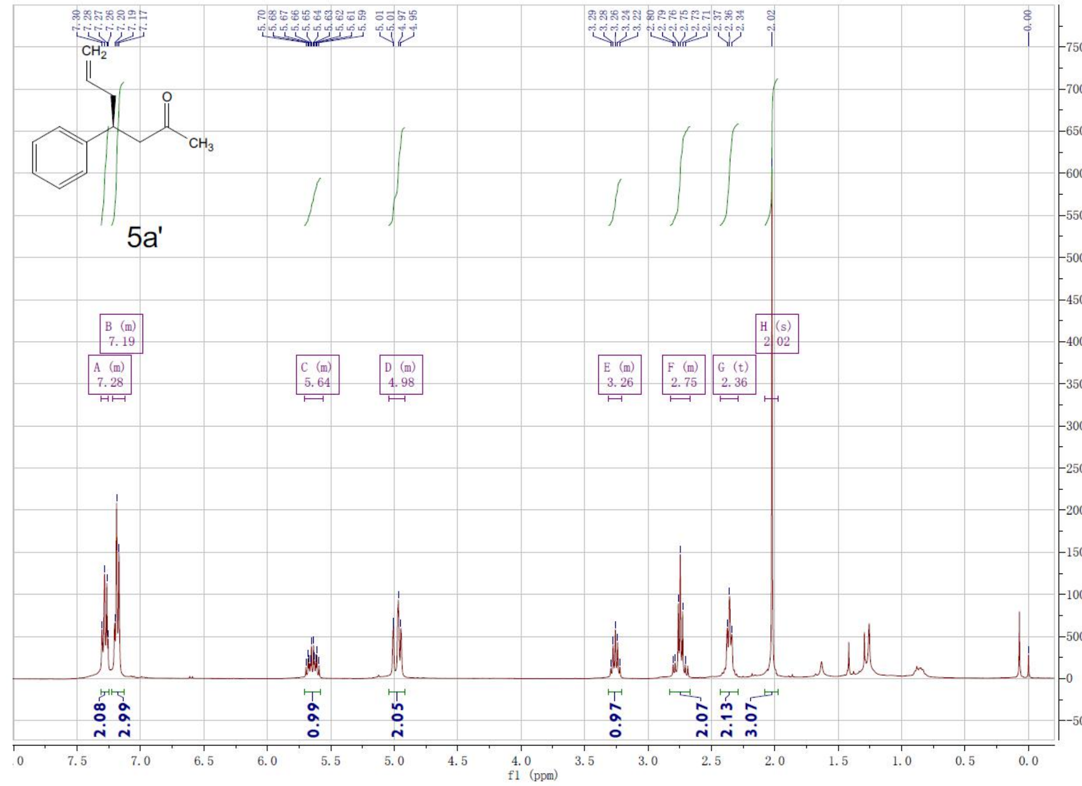

Supplementary Figure 232. ^{1}H NMR spectrum for compound **5a'**

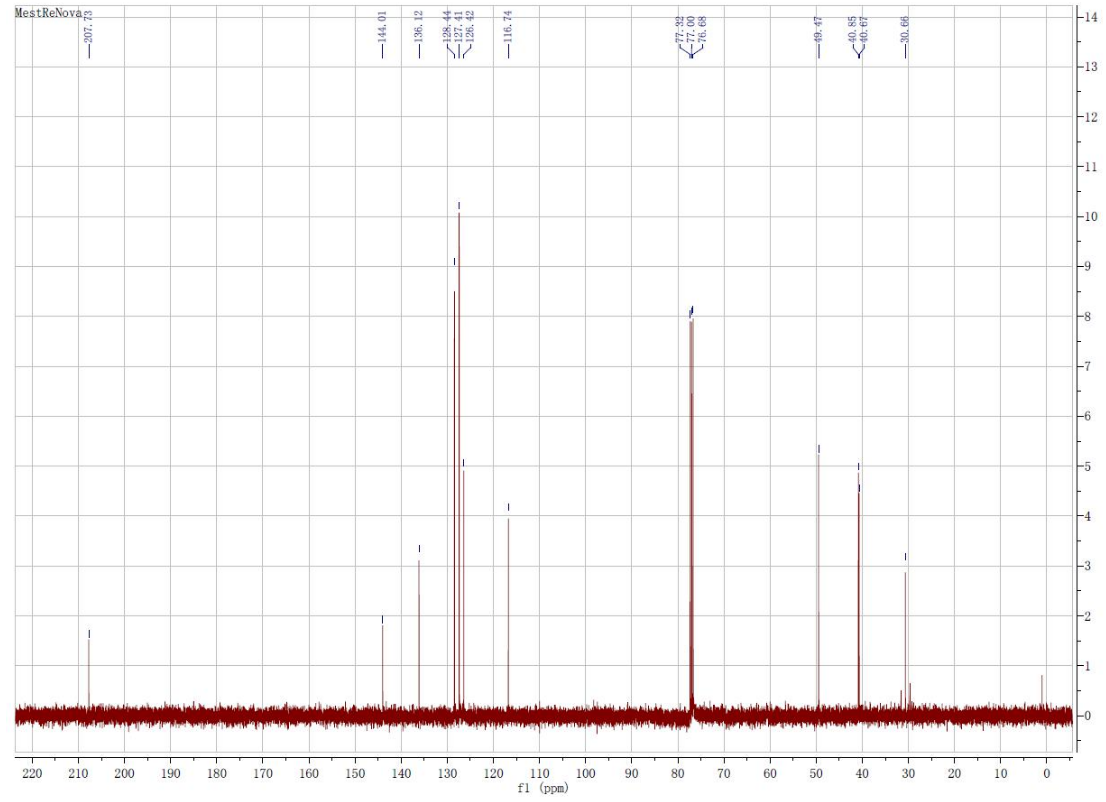

Supplementary Figure 233. ^{13}C NMR spectrum for compound **5a'**

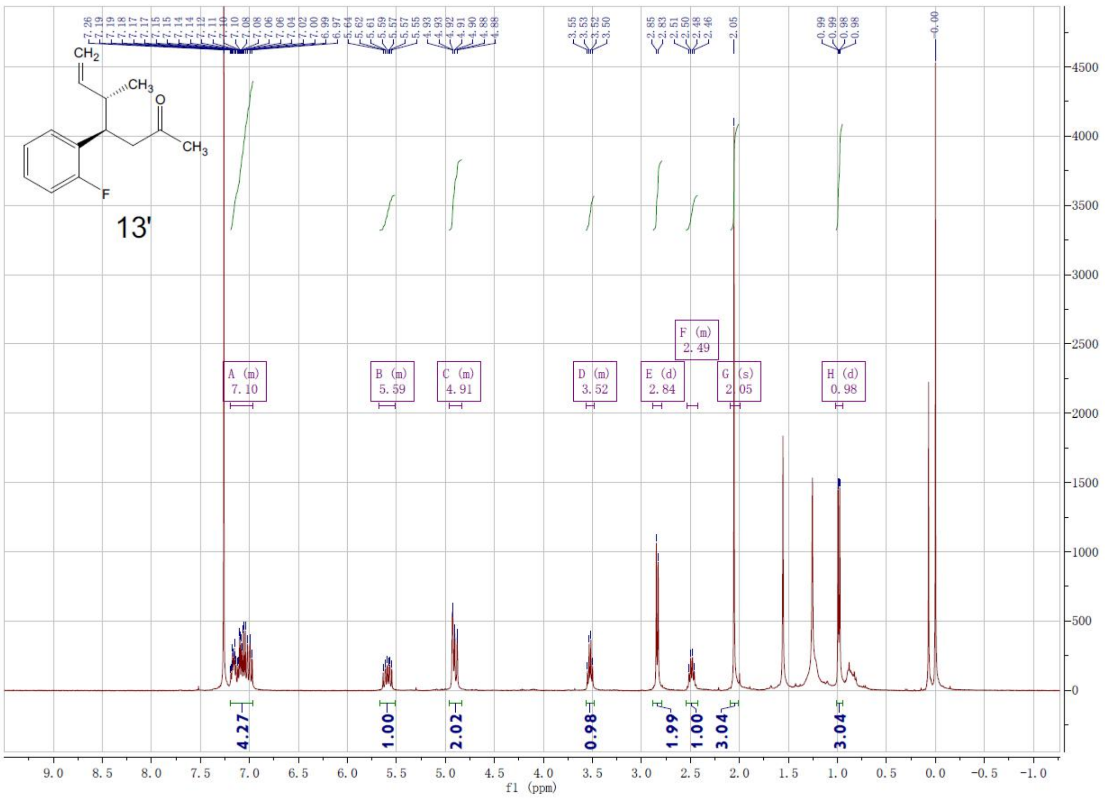

Supplementary Figure 234. ^{1}H NMR spectrum for compound **13'**

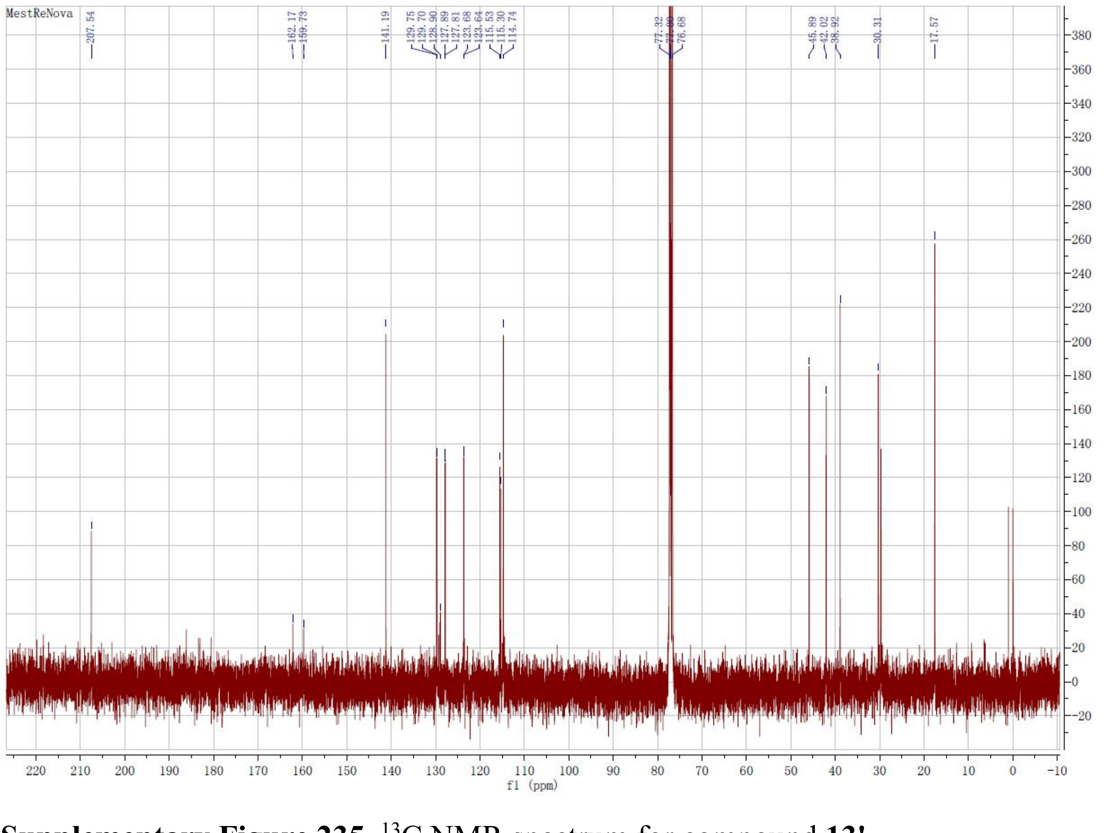

Supplementary Figure 235. ^{13}C NMR spectrum for compound **13'**

Supplementary References of Copper(I)-Catalyzed Asymmetric 1,6-Conjugate Allylation

[1] Panunzio, M.; Lentini, M. A.; Campana, E.; Martelli, G.; Tamanini, E.; Vicennati, P. Multistep microwave-assisted solvent-free organic reactions: Synthesis of 4-oxo-tetrahydro-pyridine. *Syn. Commun*. **34**, 345-359 (2004).

[2] Yamamoto, Y.; Fujikawa, R.; Umemoto, T.; Miyaura, N. Iridium-catalyzed hydroboration of alkenes with pinacolborane. *Tetrahedron* **60**, 10695-10700 (2004).

[3] Mlynarski, S. N.; Karns, A. S.; Morken, J. P. Direct stereospecific amination of alkyl and aryl pinacol boronates. *J. Am. Chem. Soc*. **134**, 16449-16451 (2012).

[4] Chen, M.; Hartwig, J. F. Iridium-catalyzed regio- and enantioselective allylic substitution of silyl dienolates derived from dioxinones. *Angew. Chem. Int. Ed*. **53**, 12172-12176 (2014).

[5] Wright, T. B.; Turnbull, B. W. H.; Evans, P. A. Enantioselective rhodium-catalyzed allylic alkylation of β,γ-unsaturated α-amino nitriles: Synthetic homoenolate equivalents. *Angew. Chem. Int. Ed*. **58**, 9886-9890 (2019).

[6] Yamamoto, Y.; Nishii, S. The anti-selective Michael addition of allylic organometals to ethylidenemalonates and related compounds. *J. Org. Chem*. **53**, 3597-3603 (1988).
